# Dynamic Adaptive Response of *Pseudomonas aeruginosa* to Clindamycin/Rifampicin-Impregnated Catheters

**DOI:** 10.3390/antibiotics10070752

**Published:** 2021-06-22

**Authors:** Kidon Sung, Jungwhan Chon, Ohgew Kweon, Seongwon Nho, Seongjae Kim, Miseon Park, Angel Paredes, Jin-Hee Lim, Saeed A. Khan, Kenneth Scott Phillips, Carl E. Cerniglia

**Affiliations:** 1 National Center for Toxicological Research, Division of Microbiology, US FDA, Jefferson, AR 72079, USA; alvarmar@naver.com (J.C.); oh-gew.kweon@fda.hhs.gov (O.K.); seongwon.nho@fda.hhs.gov (S.N.); seong-jae.kim@fda.hhs.gov (S.K.); miseon.park@fda.hhs.gov (M.P.); saeed.khan@fda.hhs.gov (S.A.K.); carl.cerniglia@fda.hhs.gov (C.E.C.); 2 Office of Scientific Coordination, National Center for Toxicological Research, US FDA, Jefferson, AR 72079, USA; angel.paredes@fda.hhs.gov; 3 NCTR-ORA Nanotechnology Core Facility, US FDA, Jefferson, AR 72079, USA; jihee3140@gmail.com; 4 Division of Biology, Chemistry, and Materials Science, Center for Devices and Radiological Health, US FDA, Silver Spring, MD 20993, USA; kenneth.phillips@fda.hhs.gov

**Keywords:** adaptive response, *Pseudomonas aeruginosa*, clindamycin/rifampicin-impregnated catheters

## Abstract

Pseudomonas aeruginosa is the most common Gram-negative pathogen causing nosocomial multidrug resistant infections. It is a good biofilm producer and has the potential for contaminating medical devices. Despite the widespread use of antibacterial-impregnated catheters, little is known about the impacts of antibacterial coating on the pathogenesis of *P. aeruginosa*. In this study, we investigated the adaptive resistance potential of *P. aeruginosa* strain PAO1 in response to continuous antibiotic exposure from clindamycin/rifampicin-impregnated catheters (CR-IC). During exposure for 144 h to clindamycin and rifampicin released from CR-IC, strain PAO1 formed biofilms featuring elongated and swollen cells. There were 545 and 372 differentially expressed proteins (DEPs) identified in the planktonic and biofilm cells, respectively, by ultra-high performance liquid chromatography-tandem mass spectrometry (UHPLC-MS/MS). Both Cluster of Orthologous Groups (COG) and Kyoto Encyclopedia of Genes and Genomes (KEGG) analyses showed that the planktonic cells responded to the released antibiotics more actively than the biofilm cells, with metabolism and ribosomal biosynthesis-associated proteins being significantly over-expressed. Exposure to CR-IC increased the invasion capability of *P. aeruginosa* for Hela cells and upregulated the expression of certain groups of virulence proteins in both planktonic and biofilm cells, including the outer membrane associated (flagella, type IV pili and type III secretion system) and extracellular (pyoverdine) virulence proteins. Continuous exposure of *P. aeruginosa* to CR-IC also induced the overexpression of antibiotic resistance proteins, including porins, efflux pumps, translation and transcription proteins. However, these upregulations did not change phenotypic minimum inhibitory concentration (MIC) during the experimental timeframe. The concerning association between CR-IC and overexpression of virulence factors in *P. aeruginosa* suggests the need for additional investigation to determine if it results in adverse clinical outcomes.

## 1. Introduction

More than 30 million urinary tract catheters and five million central venous catheters are annually used in the US [1]. Although medical devices are widely used for therapeutic treatments, infections associated with the presence of a device result in significant morbidity and mortality for patients [2]. Nosocomial bacteria can contaminate the surgical site surrounding implants and adhere to their surface to form biofilm, resulting in the evasion of the host immune response and strong resistance to many antimicrobials [3]. If antibacterial therapy is discontinued, persister cells can detach from the biofilm and cause recurrent infections [4]. Host factors and material surface characteristics influence biofilm formation [5,6]. Biofilm can also prevent devices from functioning properly [2]. Biofilms formed on intravascular catheters or central lines are recognized to be the most common cause of hospital acquired bloodstream infections [7].

*Pseudomonas aeruginosa* is responsible for more than two million nosocomial infections, especially in immunocompromised patients, and about 90,000 deaths each year [8]. *P. aeruginosa* biofilm is found in the lungs of cystic fibrosis patients, surgical sites, chronic wounds, mechanical heart valves, central venous catheters and Foley catheters [9]. *P. aeruginosa* is capable of building biofilm not only on biotic or abiotic surfaces but also on air–liquid interfaces [10]. Antibiotic treatment for *P. aeruginosa* infections is a matter of concern because it is not susceptible to many antibiotics [11]. The imprudent or excessive use of antibiotics exacerbates the emergence of multidrug resistant *P. aeruginosa* [12].

Intrinsic and acquired resistances of *P. aeruginosa* are stable and result from irreversible genetic changes [13]. By contrast, adaptive antibacterial resistance is temporary and result in gene or protein expression changes due to exposures to sub-lethal doses of antibiotics [14,15]. *P. aeruginosa* treated with sub-inhibitory concentrations of antibacterial agents for extended time periods resulted in alterations of gene expression profiles, which allowed the microorganisms to survive following exposure to normally efficacious doses of antibiotics [14]. The induction of adaptive resistance through prolonged courses with antibiotics reduced antibiotic effectiveness, resulting in the failure of antibacterial therapy [14].

In order to control biofilm colonization, the internal and external surfaces of venous catheters may be coated with antibiotics [16,17,18]. Two commercially available catheters impregnated with combinations of clindamycin/rifampicin and minocycline/rifampicin have been approved by the US Food and Drug Administration (FDA) [16,17]. It has been reported that clindamycin/rifampicin-coated and minocycline/rifampicin-coated catheters had no effect on the growth of *P. aeruginosa* [16,17,18]. These results have increased concern over the possible contamination of *P. aeruginosa* and the development of microbial resistance to the antibiotics-impregnated in the catheters. Therefore, it is important to understand how *P. aeruginosa* may overcome these antibiotic-impregnated catheters and whether the presence of the antimicrobial in devices can enable antibacterial resistance. 

To the best of our knowledge, no studies have been conducted to investigate the potential of adaptive resistance and cross-resistance to antibiotics after exposure of *P. aeruginosa* to the antibiotic-coated catheters. In this study, we investigated the response of *P. aeruginosa* PAO1 to the antibiotics eluted from clindamycin/rifampicin-impregnated catheters (CR-IC) by using genetic, minimum inhibitory concentration (MIC) and proteomic analyses to evaluate the potential development of adaptive-resistance and cross-resistance. 

## 2. Materials and Methods

### 2.1. In Vitro Continuous Culture 

Overnight-grown *P. aeruginosa* strain PAO1, *Staphylococcus aureus* strains ATCC 25923 and ATCC BAA-1717 were inoculated into fresh tryptic soy broth (TSB) (Thermo Fisher Scientific, Waltham, MA, USA) and grown at 37 °C for 7 h. The bacterial culture was centrifuged (Eppendorf, Framingham, MA, USA) at 10,000 rpm at 4 °C for 10 min and washed three times with phosphate-buffered saline (PBS) (Thermo Fisher Scientific). The bacterial cell density was adjusted to 1.3 × 10^7^ CFU/mL and inoculated into a CDC biofilm bioreactor (BioSurface Technologies, Bozeman, MT, USA) containing clindamycin/rifampicin-impregnated catheters (CR-IC, Integra LifeSciences, Plainsboro, NJ, USA) or control catheters (without antibiotic impregnation). The culture was stirred at 150 rpm at 37 °C for 144 h and the fresh medium continuously flowed through the reactor at 15 mL/min.

Following the completion of the continuous culture, cells were taken for proteomic and phenotypic analyses. For the bacterial count, 10 mm catheter segments were washed with PBS three times, sonicated for 5 min at 40 kHz and vigorously vortexed for 5 min before and after sonication. Then, the bacterial suspension was serially diluted and plated in triplicate onto tryptic soy agar (TSA) plates and the number of CFU was counted. The bacterial contamination was verified by colony morphology, VITEK 2 GN I card (bioMerieux, Durham, NC, USA) and MALDI Biotyper CA System (Bruker Daltonics, Billerica, MA, USA).

### 2.2. Measurement of Growth Curves

Strain PAO1 culture was suspended in PBS after overnight growth and adjusted to an optical density at 600 nm of 0.1. This suspension was diluted 1:100 in TSB (control) or TSB containing various mixtures of clindamycin and rifampicin (3.2 and 0.4, 6.4 and 0.8, 12.8 and 1.6 and 25.6 and 3.2 µg/mL, respectively) in a 96-well plate. The 96-well plate was incubated in a Synergy 2 Multi-Mode Microplate Reader (BIOTEK Instruments, Winooski, VT, USA) at 37 °C and then shaken continuously. Bacterial growth was monitored by reading an absorbance of 600 nm every 30 min for 24 h. All experiments were performed in triplicate.

### 2.3. Antimicrobial Susceptibility Testing

At the end of the continuous culture in the CDC biofilm bioreactor, *P. aeruginosa* strain PAO1 was harvested and inoculated on Mueller-Hinton agar (MHA, Hardy Diagnostics, Santa Maria, CA, USA) plates. After 24 h incubation at 37 °C, the colonies were suspended in 0.45% sterile sodium chloride solution and the turbidity was adjusted to a McFarland 0.5 standard by using the Densichek system (bioMérieux). VITEK 2 AST-GN69 and AST-XN06 antimicrobial susceptibility cards (bioMérieux) were placed in the VITEK 2 instrument and read by fluorescence measurement. Antimicrobial susceptibility of clindamycin and rifampicin was measured by the broth dilution method described by Shakir et al. [19].

### 2.4. Detection of Mutations

The target genes, such as *ampC* (positions and amplicon sizes: 921,961–921,147, 815 bp and 920,537–921,331, 795 bp), *gyrB* (position and amplicon size: 5321–5831, 511 bp), *oprD* (positions and amplicon sizes: 4,469,794–4,470,534, 741 bp and 4,470,514–4,471,184, 671 bp) and *rpoB* (position and amplicon size: 736,499–737,288, 790 bp), associated with avibactam, ceftazidime, ceftolozane, fluoroquinolones, imipenem, meropenem, rifampicin and tazobactam resistance were amplified by PCR and purified by the QIAquick PCR purification kit (Qiagen, Germantown, MD, USA). Both strands of the purified PCR amplicons were sequenced and the mutations were analyzed using EditSeq, MegAlign and SeqMan programs from the DNASTAR Lasergene 12 package (DNASTAR, Madison, WI, USA). Mutations of the protein sequences were compared to strain PAO1 reference protein sequences by BLASTx (http://blast.ncbi.nlm.nih.gov/Blast.cgi).

### 2.5. Determination of Efflux Pump Activity

Accumulation of ethidium bromide was assayed as described previously [20]. Strain PAO1 was grown in TSB overnight and washed with 50 mM sodium phosphate buffer (pH 7.0). In order to measure the presence of active efflux, ethidium bromide (10 µg/mL) was added to control (TSB only) and antibiotic-containing wells (6.4 µg/mL clindamycin and 0.8 µg/mL rifampicin) and fluorescence was read every 5 min at excitation 500 nm and emission 590 nm for 60 min in a fluorescence microplate reader using SoftMax Pro software (Molecular Devices, San Jose, CA, USA). All experiments were performed in triplicate. Error bars indicate standard deviation from the mean.

### 2.6. Invasion Assay

HeLa cells (ATCC CCL-2) were grown to approximately 5 × 10^5^ cells in a 24-well plate at 37 °C with 5% CO_2_. Strain PAO1 harvested from the continuous culture was inoculated into TSB and grown to mid-logarithmic phase. After centrifugation and washing, strain PAO1 was added to a monolayer of epithelial cells at a multiplicity of infection (MOI) of 100:1. Bacterial numbers spiked to HeLa cells were confirmed by a plate count method. The human cells were incubated for 2 h and washed with PBS. Thereafter, 300 μg/mL of gentamicin (Sigma-Aldrich, St. Louis, MO, USA) was added to the growth medium and incubated for another 2 h. Triton X-100 (0.25%, Sigma-Aldrich) was employed to lyse the monolayers and the released intracellular bacteria were counted. The invasion percentage was calculated by dividing the number of invading cells of strain PAO1 by the initial number of inoculated bacteria. The assays were performed in quadruplicate and data were represented as mean ± standard deviation.

### 2.7. Field Emission Scanning Electron Microscope (FESEM)

Harvested planktonic and biofilm cells were rinsed three times with PBS for 10 min and dehydrated using 15%, 50%, 90% and 95% ethanol for 10 min and 100% ethanol for 30 min. The cells were dried in an Autosamdri-815 Series A automatic critical point drier (Tousimis Research Corporation, Rockville, MD, USA) and sputter-coated using gold (Denton Vacuum, Moorestown, NJ, USA). FESEM images were obtained by a Zeiss-Merlin FESEM (Carl Zeiss Microscopy, Thornwood, NY, USA).

### 2.8. Protein Extraction and Liquid Chromatography-Tandem Mass Spectrometry

Planktonic and biofilm cells were harvested after in vitro continuous culture. Cells were placed in Lysing Matrix B tubes (MP Biomedicals, Santa Ana, CA, USA) containing 100 µL of BugBuster Plus Lysonase kit (Millipore Sigma, Burlington, MA, USA). Using an FP120 reciprocator (MP Biomedicals), cells were broken down by bead beating and boiling. The concentration of extracted protein was quantified by a Micro BCA Protein Assay Kit (Thermo Fisher Scientific). Reconstitution of the protein sample was performed in 5% SDS, 50 mM Tris-HCl (pH 8.0), 5 mM Tris (2-carboxyethyl) phosphine and 20 mM 2-chloroacetamide. Then, the sample was digested by the single-pot solid-phase-enhanced sample preparation method and analyzed by ultra-high performance liquid chromatography-tandem mass spectrometry (UHPLC-MS/MS) (Thermo Fisher Scientific) [21]. LC was carried out on an EASY-nLC 1200 (Thermo Fisher Scientific) connected to a Q-Exactive HF-X quadrupole-Orbitrap mass spectrometer (Thermo Fisher Scientific). MS was set to perform with the top 12 ions and a scan range of 350–1400 *m*/*z*. Normalized collision energy was set at 27, automatic gain control to 3 × 10^6^, maximum fill MS to 45 ms and maximum fill MS/MS to 22 ms. Data processing and library searching were performed as described by Jean-Toussaint et al. [22]. MS1-based isotopic features were detected and peptide peak areas were calculated using OpenMS (http://dx.doi.org/10.1186/1471-2105-9-163). Proteins were required to have one or more unique peptides across the analyzed samples with E-value scores of 0.0001 or less. If a fold ratio between the control and treated groups was ≥2.0 (up) and ≤0.5 (down), they were considered to be differentially expressed. The Cluster of Orthologous Groups (COG) was employed to classify the function of proteins [23] and the Kyoto Encyclopedia of Genes and Genomes (KEGG) database was used to map the potential pathway [24]. Protein interaction network analysis was performed using STRING version 11.0 (http://string-db.org/) [25]. The heatmap function in R (https://www.r-project.org/) was used to build a heatmap of the proteomic data.

## 3. Results and Discussion

### 3.1. Cell Morphology and Antimicrobial Susceptibility of P. aeruginosa PAO1

**Figure 1:**
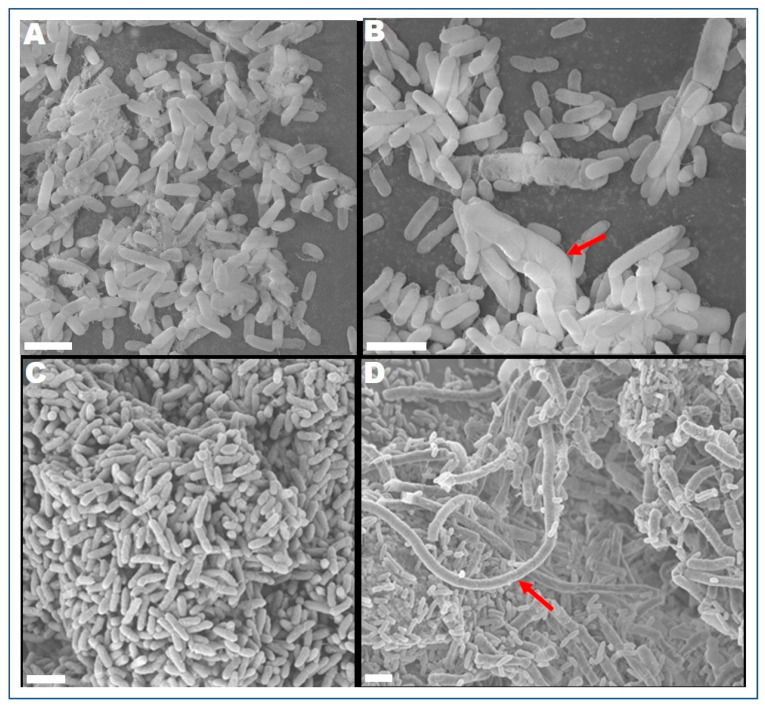
FESEM images of *P. aeruginosa* PAO1 biofilm cells after 144 h. The scale bar in all the images corresponds to 2.0 μm. (**A**) Air–liquid interface biofilm grown with control catheters, (**B**) air–liquid interface biofilm grown with antibiotic-impregnated catheters (Arrow indicates a swollen cell), (**C**) biofilm cells on control catheters and (**D**) biofilm cells on antibiotic-impregnated catheters (Arrow indicates an elongated cell).

*P. aeruginosa* PAO1 survived well when it was grown with CR-IC in the continuous culture but *S. aureus* strains ATCC 25923 and ATCC BAA-1717 did not. After 144 h of culture, the average counts of strain PAO1 biofilm cells per control segment and CR-IC segment were 7.31 ± 0.23 and 7.33 ± 0.42 log CFU, respectively. Morphologies of strain PAO1 biofilm cells grown on catheter sections and the air–liquid interface were compared (Figure 1A–D). The biofilm cells grown with CR-IC were elongated up to approximately 25 µm, showing swollen and filamentous growth. Moreover, the biofilm cells formed on CR-IC were more elongated than those on the air–liquid interface. Elongation of biofilm cells has been observed when Gram negative bacteria, including *Escherichia coli* and *P. aeruginosa,* were exposed to antibacterial agents [26,27]. By modifying its cell morphology, strain PAO1 probably responded to stress caused by the antibiotics released from CR-IC.

The MICs of aminoglycosides (amikacin, gentamicin and tobramycin), cephalosporins (cefazolin, cefepime, cefotaxime and ceftazidime), carbapenems (doripenem, imipenem and meropenem), penicillin (piperacillin), fluoroquinolones (ciprofloxacin, levofloxacin and norfloxacin) and glycylcycline (tigecycline), including clindamycin and rifampicin against strain PAO1 cultivated in the presence of control catheters and CR-IC, were not significantly different (Supplementary Table S1). Although antibiotics eluted from CR-IC could not change the MICs of *P*. *aeruginosa* strain PAO1, they exhibited an antibacterial effect with large inhibition zone diameters (10–15 mm) against *S*. *aureus* ATCC 25923 over 144 h. This indicates that antibiotics eluted from CR-IC do not possess the antibacterial potency to increase the MICs against *P*. *aeruginosa* but can sustain antibacterial activity against *S*. *aureus*. Mutations in *ampC*, *gyrB*, *oprD* and *rpoB* were not found. These genes were known to have mutations when *P. aeruginosa* was exposed to avibactam, ceftazidime, ceftolozane, fluoroquinolones, imipenem, meropenem, rifampicin and tazobactam [28]. 

### 3.2. Proteomic Insights into the Global Cellular Response to Clindamycin/Rifampicin

#### 3.2.1. Global Overview of the Proteome Data

**Figure 2:**
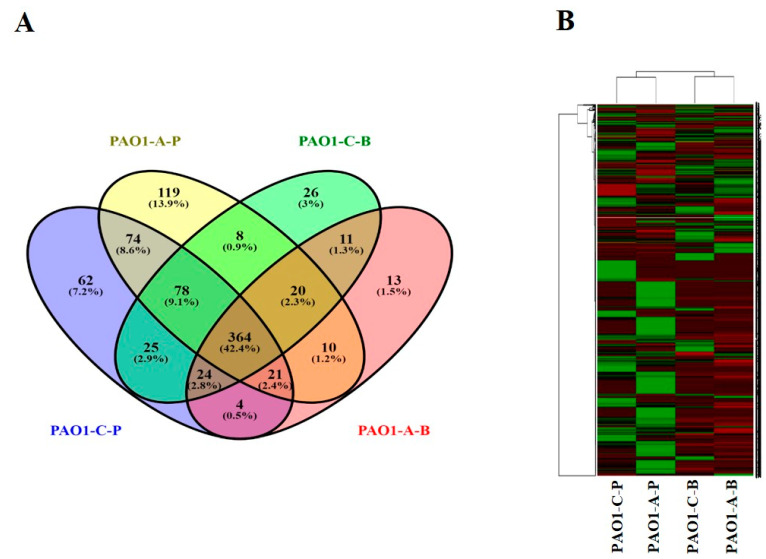
Venn diagram (**A**) and heatmap analysis (**B**) of *P. aeruginosa* PAO1 proteomic data. PAO1-C-P: planktonic cells grown with control catheters; PAO1-A-P: planktonic cells grown with antibiotic-coated catheters; PAO1-C-B: biofilm cells grown with control catheters; PAO1-A-B: biofilm cells grown with antibiotic-coated catheters.

From the proteomic data analysis, 694 and 556 proteins were identified from the planktonic (CP) and biofilm (CB) cells, respectively, grown with control catheters and 652 and 467 proteins from the planktonic (AP) and biofilm (AB) cells, respectively, grown with CR-IC (Figure 2A). A Venn diagram indicated that 364 proteins were commonly identified in all comparison groups, whereas 62, 119, 26 and 13 proteins were uniquely detected in CP, AP, CB and AB, respectively. The heatmap showed that proteins identified from the planktonic and the biofilm cells were clustered into each single branch, respectively (Figure 2B). Differentially regulated proteins of 545 in planktonic cells were identified, 326 of which displayed upregulation and 219 displayed downregulation (Table S2). On the other hand, 372 proteins were differentially expressed in biofilm cells containing 137 that were upregulated and 235 that were downregulated (Table S3).

**Figure 3:**
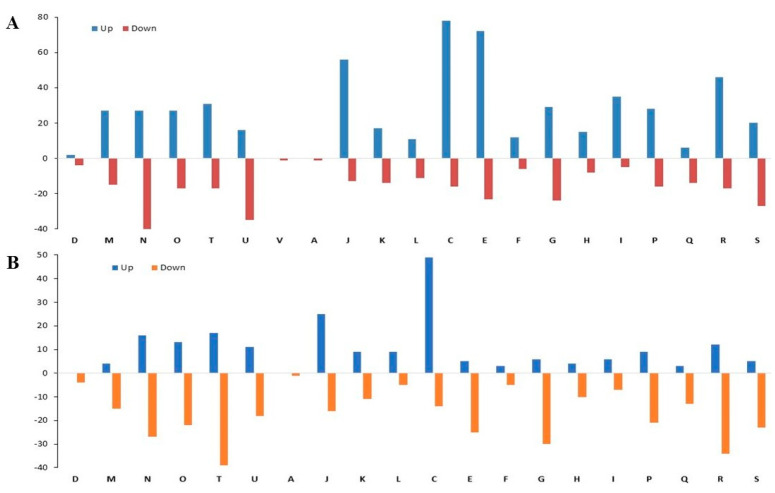
COG functional classification of the planktonic (**A**) and biofilm (**B**) cells grown with control and antibiotic-impregnated catheters. Y-axis with positive and negative values referring to upregulated and downregulated proteins, respectively. CELLULAR PROCESSES AND SIGNALING; (D) cell cycle control, cell division and chromosome partitioning; (M) cell wall/membrane/envelope biogenesis; (N) cell motility; (O) post-translational modification, protein turnover and chaperones; (T) signal transduction mechanisms; (U) intracellular trafficking, secretion and vesicular transport; (V) defense mechanisms. INFORMATION STORAGE AND PROCESSING; (A) RNA processing and modification; (J) translation, ribosomal structure and biogenesis; (K) transcription; (L) replication, recombination and repair. METABOLISM; (C) energy production and conversion, (E) amino acid transport and metabolism; (F) nucleotide transport and metabolism; (G) carbohydrate transport and metabolism; (H) coenzyme transport and metabolism; (I) lipid transport and metabolism; (P) inorganic ion transport and metabolism; (Q) secondary metabolites biosynthesis, transport and catabolism; POORLY CHARACTERIZED; (R) general function prediction only; (S) function unknown.

According to COG functional category analysis [29], the differentially expressed proteins (DEPs) were classified into four main functional categories: (1) Cellular processes and signaling; (2) Information storage and processing; (3) Metabolism; and (4) Poorly characterized (Figure 3A,B). There were more proteins in each COG category that were upregulated than downregulated in AP/CP (Figure 3A); however, in the COG analysis of AB/CB, more proteins were repressed (Figure 3B). The predominant COGs of upregulated proteins in AP/CP were energy production and conversion (C, 78); amino acid transport and metabolism (E, 72); and translation, ribosomal structure and biogenesis (J, 56), while those in under-expressed proteins were cell motility (N, 40); intracellular trafficking; secretion and vesicular transport (U, 35); and function unknown (S, 27). The main COGs of downregulated proteins in AB/CB included signal transduction mechanisms (T, 39); general function prediction only (R, 34); and carbohydrate transport and metabolism (G, 30), while the most upregulated proteins were found in energy production and conversion (C, 49).

**Figure 4:**
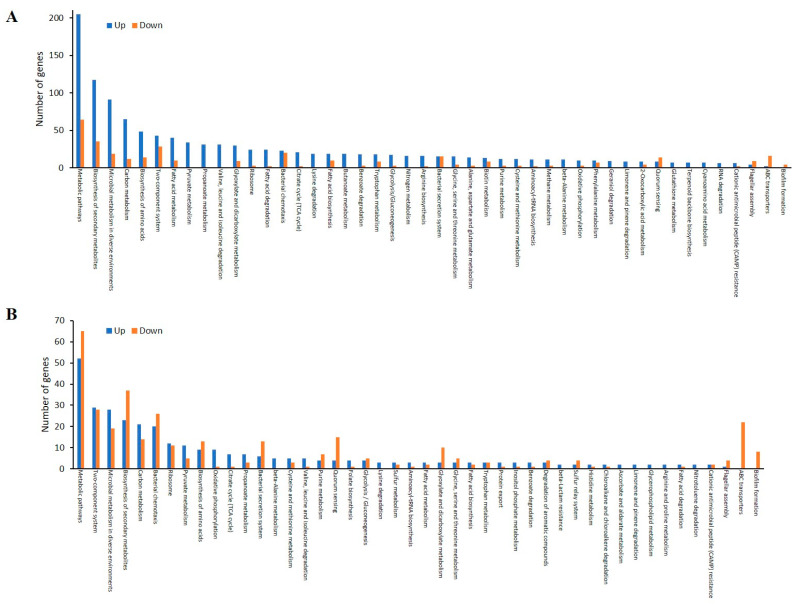
Comparison of KEGG pathways of over-expressed proteins from the planktonic (**A**) and biofilm (**B**) cells grown in control and antibiotic-impregnated catheters.

**Figure 5:**
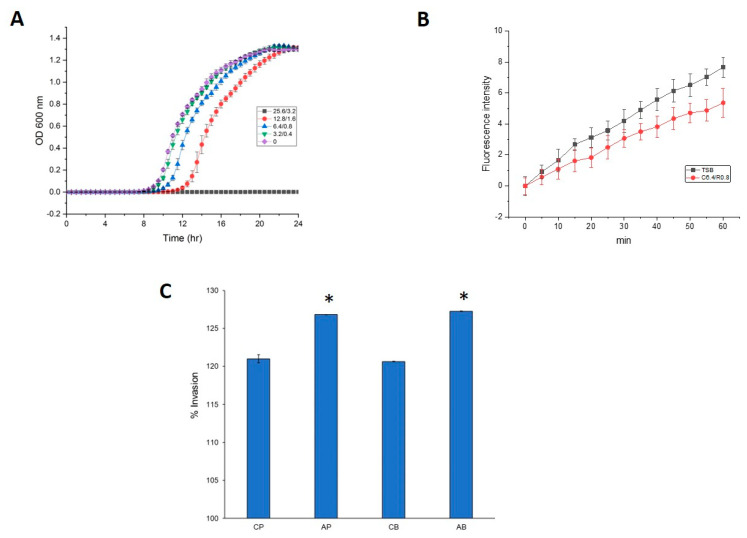
(**A**) Growth curve of strain PAO1 containing various mixtures of clindamycin and rifampicin (3.2 and 0.4, 6.4 and 0.8, 12.8 and 1.6 and 25.6 and 3.2 µg/mL, respectively) in TSB. Experiments were performed in triplicate to calculate the mean and standard deviation. (**B**) Accumulation of ethidium bromide in strain PAO1 containing a mixture of clindamycin (6.4 µg/mL) and rifampicin (0.8 µg/mL) in TSB. The natural fluorescence of the cells was subtracted and the fluorescence intensity was expressed in relative fluorescence units (RFU). All experiments were performed in triplicate. Error bars indicate standard deviation from the mean. (**C**) Invasion of *P. aeruginosa* PAO1 in the human cervical epithelial adenocarcinoma HeLa (ATCC CCL-2) cells. The % of invasion was calculated by dividing the number of invaded cells by the initial number of inoculated bacteria. The assays were performed in quadruplicate and data are represented as mean ± SD. Data were subjected to a one-way analysis of variance followed by a Tukey’s post hoc test. Differences were considered statistically significant (*) when *p* < 0.05. CP: planktonic cells grown with control catheter; CB: biofilm cells grown with control catheters; AP: planktonic cells grown with antibiotic-impregnated catheter; AB: biofilm cells grown with antibiotic-impregnated catheter.

While upregulated and downregulated proteins of AP/CP were mapped into 99 and 64 KEGG pathways, respectively, AB/CB revealed 68 upregulated and 65 downregulated pathways (Tables S4–S7, Figures S1–S4). Among five main KEGG categories including “metabolism”, “cellular processes”, “environmental information processing”, “genetic information processing” and “human diseases”, the “metabolism” pathway accounted for the majority of DEPs. Within the metabolism group, in addition to “global and overview map” category, the “carbohydrate metabolism” and “amino acid metabolism” categories were the most populated. In general, more proteins were enriched in upregulated proteins of AP/CP than AB/CB KEGG pathways, showing elevated dynamic responses of the planktonic cells than the biofilm cells to the released antibiotics from CR-IC, as shown in the COG data. Interestingly, cell motility (bacterial secretion system and flagellar assembly), membrane transport (bacterial secretion system, ATP-binding cassette (ABC) transporters and phosphotransferase system) and cellular community (quorum sensing and biofilm formation) pathways had more downregulated proteins than upregulated proteins in both AP/CP and AB/CB (Figure 4A,B). Clindamycin and rifampicin eluted from CR-IC promoted the pathways associated with metabolism and genetic information processing in the planktonic cells, but repressed the pathways associated with environmental information processing and cellular processes in the biofilm cells. A mixture of clindamycin (3.2 µg/mL) and rifampicin (0.4 µg/mL) inhibited the planktonic growth of strain PAO1 and this phenomenon was significant as the concentration increased (Figure 5A). This result supports the observation that the sublethal pressure of clindamycin and rifampicin eluted from CR-IC results in changes in the proteome profile mentioned above.

#### 3.2.2. Antibiotic Resistance Proteins of CR-IC-Treated Planktonic Cells

From the planktonic cells of strain PAO1, 21 antibacterial resistance proteins were identified, including 14 upregulated and 2 downregulated proteins (Table 1) [30]. These DEPs primarily belonged to porins, efflux pumps, translation and transcription. Porins are outer membrane proteins that mediate cellular permeability and antibiotic resistance [31,32,33]. *P. aeruginosa* has four different classes of porins: nonspecific (OprF), specific (OprB, OprD, OprE, OprO and OprP), gated (OprC, OprH and FpvA) and efflux porins (OprM, OprN and OprJ) [34]. FpvA, OprB, PA2291 (encoding OprB2) and OprH were only expressed in the planktonic cells challenged with CR-IC. OprB and PA2291 are outer membrane channel porins that play essential roles in glucose uptake and tobramycin resistance in *P. aeruginosa* [31,35]. FpvA, a ferripyoverdine receptor, and OprH, a low Mg^2+^ inducible porin, have been reported to be involved in resistance to a thiopeptide antibiotic, thiostrepton, polymyxin B and gentamicin [36]. OprD, which is a specific porin for translocating amino acids, peptides and carbapenem [32], was highly upregulated at a fold ratio of 13.2. However, OprE (OccK8), a channel-forming outer membrane porin related to cefepime, ceftazidime and chromate resistance, which is induced under oxygen-depleted conditions, was downregulated [33]. In our study, the presence of CR-IC led to the differential expression of specific and gated porin groups, such as OprB, OprB2, OprD, OprE, OprH and FpvA, which have been reported to cause resistance to a variety of antibiotics [31,32,33,35,36]. Although OprF is known as the major porin in *P. aeruginosa* [37], it was not significantly expressed (fold ratio 1.41). Treatment of *E. coli*, *Aeromonas hydrophila* and *Acinetobacter baumannii* strains with low concentrations of chloramphenicol and tetracycline to inhibit protein synthesis changed the expression of porin proteins and contributed to an increase in antibacterial resistance [38,39,40].

Multidrug efflux systems for pumping antibacterial molecules out of the bacterial cell reduce accumulation of antimicrobials within the cell [41]. MexAB-OprM and MexXY-OprM efflux pump operons of *P. aeruginosa* have been reported to export incoming antibacterial compounds across the membranes and contribute to the resistance against macrolides, β-lactams, tetracycline and chloramphenicol [42,43]. In this study, expression of OprM was significantly increased at a fold ratio of 18.4. Prior work demonstrated that *P. aeruginosa* cultivated under azithromycin, colistin and tobramycin over-expressed OprM [44,45]. However, MexA, MexB, MexX and MexY were either not differentially regulated (fold ratio 0.71) or not identified. Expression of OpmB, which is an outer membrane efflux protein that confers resistance to ampicillin, aztreonam, carbenicillin, macrolides, novobiocin and tetracycline [46], was suppressed at the highest fold ratio. Collectively, the antibiotics eluted from CR-IC, despite the low concentrations of clindamycin (0.2–24.58 µg/µL) and rifampicin (0.01–3.25 µg/µL) [47], are considered to selectively influence various porin and efflux pump proteins, which is consistent with the high efflux activities of strain PAO1 under the antibiotic pressure (Figure 5B).

Ribosomes are central components of protein biosynthesis and versatile targets for macrolides [48]. The 30S (RpsJ) and 50S (RplF) ribosomal proteins, as well as translation elongation factor proteins (TufB and FusA1), were abundantly increased under antibiotic stress, which implies the induction of the translational process by the binding of the released clindamycin of CR-IC to the ribosomal subunits. These results are in agreement with Ding et al. [49], who found that the same ribosomal proteins of strain PAO1 were up-regulated following sublethal azithromycin treatment. Moreover, many ribosomal proteins, including 30S, 50S and the translation elongation factor, were over-expressed in tetracycline-exposed *A. hydrophila* [50]. 

RpoB and RpoC are β subunits of DNA-directed RNA polymerase, which is the pivotal enzyme for transcription regulation [51]. Rifampicin binds to RpoB and hinders mRNA elongation [51]. We found that exposure of CR-IC, including clindamycin and rifampicin, to *P. aeruginosa* resulted in a significant up-expression of RpoB (fold ratio 13.15) and RpoC (fold ratio 6.81), but no mutation was detected. Similarly, when *Mycobacterium tuberculosis* or *M. smegmatis* was treated with sub-inhibitory doses of rifampicin, the expression levels of RpoB and RpoC were significantly increased [51,52]. In our results, the eluted rifampicin concentration was not high enough to cause mutations but influenced the transcription processes by targeting RpoB and RpoC proteins.

**Figure 6:**
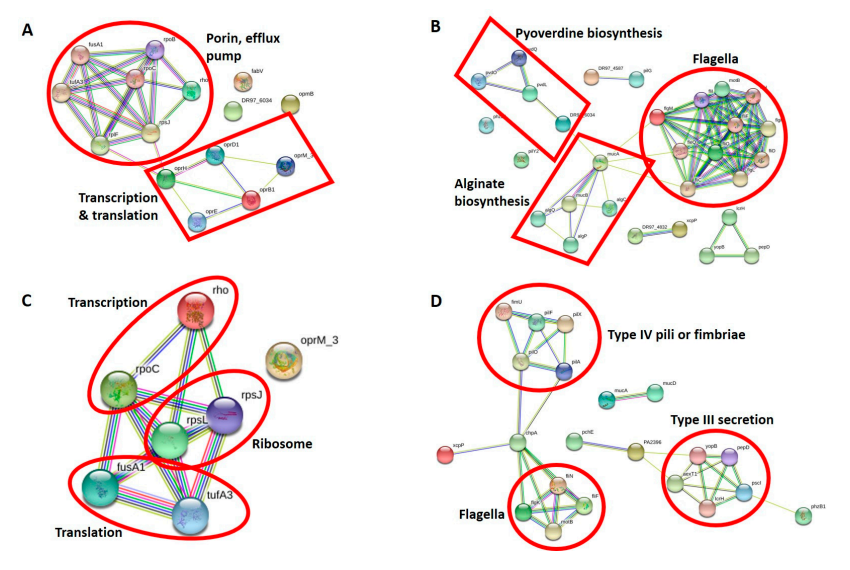
(**A**) Protein–protein interaction network analysis of DEPs associated with antibiotic resistance of planktonic cells grown with the control and antibiotic-impregnated catheters using STRING. The following proteins of *P. aeruginosa* were matched with input proteins: oprB: oprB1, oprM: oprM_3, oprD: oprD1, tufB: tufA3, fpvA: DR97-6034 and fusA2: fusB. Proteins that STRING could not find in the database: oprB2. (**B**) Protein–protein interaction network analysis of DEPs associated with virulence of planktonic cells grown with control and antibiotic-impregnated catheters using STRING. The following proteins of *P. aeruginosa* were matched with input proteins: pcrH: lcrH, popD: pepD, fpvA: DR97_6034, phzB2: phzB1, fimV: DR97_4587, xcpT: DR97_4832 and popB: yopB. Proteins STRING could not find in the database: PA1096, PA2412, PA2393, PA3352, pvdP, tagQ1 and tssB1. (**C**) Protein–protein interaction network analysis of DEPs associated with antibiotic resistance of biofilm cells grown with control and antibiotic-impregnated catheters using STRING. The following proteins of *P. aeruginosa* were matched with input proteins: tufB: tufA3 and oprM: oprM_3. (**D**) Protein–protein interaction network analysis of DEPs associated with the virulence of biofilm cells grown with control and antibiotic-impregnated catheters using STRING. The following proteins of *P. aeruginosa* were matched with input proteins: exoT: aexT1, pvdF: PA2396, pcrH: lcrH, popD: pepD and popB: yopB. Proteins that STRING could not find in the database: PA2393, PA3352 and tssB1.

Construction of a functional protein–protein network using STRING in DEPs [25] revealed two distinct clusters (Figure 6A). The proteins of the two clusters were grouped into porin and efflux pump and transcription and translation proteins, respectively. Notably, strong upregulation (fold ratio 6.6) of FabV, which is an enoyl-acyl-carrier-protein reductase that is responsible for triclosan resistance [53], was observed, although not linked to any STRING cluster.

#### 3.2.3. Virulence Proteins of CR-IC-Treated Planktonic Cells

A total of 46 virulence proteins, including 15 upregulated and 22 downregulated proteins from 244 *P. aeruginosa* PAO1 virulence protein database [30], were found from the control and treated planktonic cells (Table 2). STRING network analysis of differentially expressed virulence proteins upon the prolonged exposure of CR-IC to the planktonic cells found that three major protein clusters, containing flagella, alginate production and pyoverdine production, were connected to one another (Figure 6B). However, types II and III secretion systems and type IV pili formed independent clusters. MucA, which is an anti-sigma factor [54], was found to be the most interactive protein connecting the three clusters. It is involved in alginate biosynthesis and flagella motility in *P. aeruginosa* [54] and STRING network analysis clearly demonstrated its functional role. Flagella are pivotal virulence factors because they facilitate adherence of bacteria to host cell tissues and bacterial movement toward nutrients [55]. By the type III secretion system, flagellar proteins are pumped from the inside of the cell to the outside [56]. Early investigations showed that the exposure of bacteria to sub-inhibitory concentrations of antibacterial agents repressed flagellar expression [55,57]. Furthermore, sub-lethal concentrations of rifampicin or macrolides interfered with the production of flagellar proteins in *P. aeruginosa, Proteus mirabilis* and *Salmonella* Typhimurium [55,58]. Consistent with these previous reports, most flagellar proteins, including flagella transcriptional regulator (FleQ), flagella biosynthesis (FliO and FlgM) and flagella basal body (FliD, FliF, FliH, FliI, FlgK and FlgL) proteins, were downregulated.

The type III secretion system of *P. aeruginosa* is reported to antagonize flagellar biosynthesis [59], which is in agreement with our result that type III regulatory (PcrH) and type III translocator outer membrane (PopD) proteins were only expressed in the CR-IC-treated planktonic cells. Type I through VI secretion systems of *P. aeruginosa* play a role in delivering the proteins into the host or bacterial cells [60]. Both type II and VI secretion proteins, such as XcpP, XcpT, TagQ1 and TssB1, were suppressed when the planktonic cells were continuously adapted to the eluted antimicrobials of CR-IC. Our results are in contrast to the previous reports of other investigators who reported an over-expression of proteins related to type II and VI secretion systems in other Gram-negative bacteria after treatment with antibacterial compounds [61,62,63,64]. Although many type IV pili proteins such as ChpA, PilA, PilF, PilG, PilH, PilJ, PilO and FimV were identified, they were neither mapped in the STRING network nor differentially regulated (fold ratios 0.56–1.21). Interference with the assembly of type IV pili on the surface of *P. aeruginosa* treated with a sub-lethal concentration of macrolide has been reported and some researchers have noted an over-expression of PilA, PilH and PilY1 [65,66,67]. 

Alginate has been shown to be involved in the resistance to antibiotics, the host immune system and biofilm formation in *P. aeruginosa* [68]. Previous reports revealed that, following exposure to macrolides and epigallocatechin gallate, alginate biosynthesis genes were under-expressed [66,69]. However, our results were unique in the sense that we observed a downregulation of alginate regulatory proteins (AlgP and MucB) but an upregulation of an alginate structural protein (AlgC) and alginate regulatory proteins (AlgQ and MucA). Phenazine (PhzB1 and PhzB2) and pyoverdine (PvdL, PvdO, PvdP and PvdQ) biosynthesis proteins were differentially expressed with varied fold changes. 

Invasion into human cervical epithelial adenocarcinoma cells by strain PAO1 cells harvested from the control and CR-IC-treated cultures was compared (Figure 5C). CR-IC-treated planktonic and biofilm cells had levels of invasion significantly higher than the control (*p* < 0.05). Taken together, our results indicate that the antibiotics released from CR-IC not only altered the phenotypic invasion potential to the HeLa epithelial cell lines but also selectively influenced cell-associated and secreted virulence proteins, including type IV pili, flagella, alginate, pyoverdine, phenazine and protein secretion systems.

#### 3.2.4. Antibiotic Resistance Proteins of CR-IC-Treated Biofilm Cells

Compared to the planktonic cells, fewer antibacterial resistance proteins (11 proteins) were identified from the biofilm cells that were cultured with CR-IC (Table 3). Among seven DEPs, six were upregulated. Even though most proteins were identified in the biofilm cells, except RpsL which were commonly found in the planktonic cells, many proteins found in the planktonic cells such as FabV, FusA2, MexA, OpmB, OprB, OprB2, OprD, OprE and RpoB were not identified in the biofilm cells. These outcomes suggest that the biofilm cells responded in different manners compared to how the planktonic cells responded to the antibiotic pressure from CR-IC.

STRING network analysis of seven differentially expressed antibacterial resistance proteins in the biofilm showed a single cluster composed of six proteins associated with transcription (Rho and RpoC), translation (FusA1 and TufB) and ribosomal proteins (RpsJ and RpsL) (Figure 6C). RpsL and OprM were expressed only in the biofilm cells under the given antibiotic stress, suggesting that they are the key proteins responding to the antibiotics released from the catheters. It is interesting that RpsL, upon conferring resistance to streptomycin, was highly over-expressed under macrolide and rifampicin stresses [70]. In contrast to the planktonic cells, where many porin proteins were over-expressed, only OprM was upregulated in the biofilm cells. However, OprM did not interact with the cluster. We noticed that fold ratios of several proteins between the planktonic and the biofilm cells were generally decreased in RpoC (fold ratio from 6.8 to 2.7), RpsJ (fold ratio from 19.1 to 2.2), TufB (fold ratio from 3.5 to 2.1), RplF (fold ratio from 3.2 to 1.7) and Rho (fold ratio from 6.6 to 0.08). The reduced expression of these transcriptional and translational proteins may imply the slow adaptation of the biofilm cells with reduced protein synthesis to the antibiotic stress caused by CR-IC. 

#### 3.2.5. Virulence Proteins of CR-IC-Treated Biofilm Cells

From the strain PAO1 biofilm cells adapted to clindamycin and rifampicin released from CR-IC, 45 virulence proteins were identified and contained 9 upregulated and 15 downregulated proteins (Table 4). Although more antibiotic resistance proteins were identified in the planktonic cells than compared to the biofilm cells, there were no significant differences between the total number of identified virulence proteins in the planktonic and the biofilm cells. The most remarkable difference in virulence proteins between the planktonic and the biofilm cells was the smaller number of proteins (PvdF and PA2393) related to alginate and pyoverdine biosynthesis found in the biofilm cells. Likewise, in the proteomic study of Park et al. that compared planktonic and biofilm cells, various virulence-associated proteins were found less during biofilm growth [71].

STRING network analysis revealed three independent clusters that consisted of 21 proteins (Figure 6D). The first two clusters are primarily composed of 11 proteins linked to type IV pili or fimbriae (FimU, PilA, PilF, PilO and PilX) and flagella (FlgK, FliF, FliN and MotB) proteins. Most of them exhibited decreased expression in CR-IC-adapted biofilm cells compared to those in the unadapted cells, as the planktonic cells showed. ChpA interacted with the highest number of proteins, including FlgK, FliF, FliN, MotB, PilA, PilO and XcpP, suggesting that it may play a paramount role in virulence upon the antibiotic adaptation of the biofilm cells. The production, assembly and twitching motility in type IV pili are controlled by various signal transduction systems, including a chemosensory (Chp) system, in *P. aeruginosa* and one of the central signaling components of the Chp system is ChpA [72]. Therefore, it indicates that the downregulation of ChpA is closely associated with the suppression of type IV pili proteins. Major protein components of the second cluster in STRING network are type III secretion system (ExoT, PcrH, PopB, PopD and PscI), which are differentially expressed. Interestingly, we observed that ExoT and PscI proteins were not identified in the proteome analysis of the planktonic cells, but they were tremendously over-expressed in the biofilm cells. Moreover, the proteins PopD and PcrH that over-expressed in the planktonic cells at the highest fold ratio were also identified in the biofilm cells but with the decreased fold ratios of 0.41 and 2.5, respectively. There were conflicting reports regarding the alteration of type III secretion proteins following the treatment of antibacterial agents [44,73]. Most type III secretion proteins were repressed following the treatment of *P. aeruginosa* biofilm with coumarin [73], but exhibited the opposite result after azithromycin exposure [44].

## 4. Conclusions

Phenotypic feedback of *P. aeruginosa* PAO1 after an exposure to antibiotics included morphological changes and increased invasion potency. Virulence proteins including flagella, pili, alginate, pyoverdine, phenazine and protein secretion pathways were distinctly over-expressed in both planktonic and biofilm cells. The invasion capability for human cervical cancer cells was also increased, suggesting a potential risk in the clinical use of CR-IC. CR-IC-treated cells changed their protein expression related to antibiotic resistance but not their phenotypic MICs. This difference between gene/protein expression and antibacterial resistance phenotype should be further studied to better understand the relationship between epigenetic factors and MIC.

## Figures and Tables

**Table 1:** Identified proteins associated with antibiotic resistance of planktonic cells grown with control and antibiotic-impregnated catheters.

Locus Tag	Gene	Product	Fold Ratio
PA0156	triA	Multidrug efflux system, membrane fusion component	0.97
PA0291	oprE	Outer membrane low permeability porin, OprD family	0.00
PA0425	mexA	Multidrug efflux system, membrane fusion component	0.71
PA0427	oprM	Multidrug efflux system, outer membrane factor lipoprotein	18.38
PA0958	oprD	Outer membrane low permeability porin, OprD family	13.16
PA1178	oprH	PhoP/Q and low Mg^2+^ inducible outer membrane protein H1 precursor	1000.00
PA1777	oprF	Nonspecific porin and structural outer membrane protein OprF	1.41
PA2071	fusA2	Translation elongation factor G	1.37
PA2291	oprB2	Probable glucose-sensitive porin	1000.00
PA2398	fpvA	Ferripyoverdine receptor	1000.00
PA2525	opmB	Outer membrane factor (OMF) lipoprotein associated with MdtABC efflux system	0.00
PA2950	fabV	Enoyl-[acyl-carrier-protein] reductase [NADH]	6.63
PA3186	oprB	Glucose/carbohydrate outer membrane porin OprB precursor	1000.00
PA4248	rplF	LSU ribosomal protein L6p (L9e)	3.21
PA4264	rpsJ	SSU ribosomal protein S10p (S20e)	19.09
PA4266	fusA1	Translation elongation factor G	3.45
PA4269	rpoC	DNA-directed RNA polymerase beta subunit	6.81
PA4270	rpoB	DNA-directed RNA polymerase beta subunit	13.15
PA4277	tufB	Translation elongation factor Tu	3.46
PA4974		Outer membrane channel TolC (OpmH)	1.04
PA5239	rho	Transcription termination factor Rho	6.61

**Table 2:** Identified proteins associated with virulence of planktonic cells grown with control and antibiotic-impregnated catheters.

Locus Tag	Gene	Product	Fold Ratio
PA0070		Autotransporter adhesin	0.46
PA0083	tssB1	T6SS component TssB (ImpB/VipA)	0.28
PA0408	pilG	Twitching motility protein PilG	0.00
PA0409	pilH	Twitching motility protein PilH	0.79
PA0411	pilJ	Twitching motility protein PilJ	0.74
PA0413	chpA	Still frameshift probable component of chemotactic signal transduction system	1.00
PA0763	mucA	Sigma factor RpoE negative regulatory protein RseA	1000.00
PA0764	mucB	Sigma factor RpoE negative regulatory protein RseB precursor	0.00
PA0766	mucD	HtrA protease/chaperone protein	0.72
PA1086	flgK	Flagellar hook-associated protein FlgK	0.00
PA1087	flgL	Flagellar hook-associated protein FlgL	0.25
PA1092	fliC	Flagellin protein FlaB	7.01
PA1094	fliD	Flagellar cap protein FliD	0.13
PA1096		Two-component system sensor histidine kinase	0.00
PA1097	fleQ	Flagellar regulatory protein FleQ	0.21
PA1101	fliF	Flagellar M-ring protein FliF	0.00
PA1103	fliH	Flagellar assembly protein FliH	0.06
PA1104	fliI	Flagellum-specific ATP synthase FliI	0.00
PA1445	fliO	Flagellar biosynthesis protein FliO	0.00
PA1707	pcrH	Type III secretion chaperone protein for YopD (SycD)	1000.00
PA1708	popB	Type III secretion host injection protein (YopB)	0.00
PA1709	popD	Type III secretion host injection and negative regulator protein (YopD)	1000.00
PA1900	phzB2	Phenazine biosynthesis protein PhzB	1000.00
PA2385	pvdQ	Acyl-homoserine lactone acylase PvdQ	24.33
PA2392	pvdP	Pyoverdine biosynthesis related protein PvdP	0.00
PA2393		Putative dipeptidase, pyoverdin biosynthesis PvdM	7.82
PA2395	pvdO	PvdO, pyoverdine responsive serine/threonine kinase (predicted by OlgaV)	2.22
PA2396	pvdF	Pyoverdine synthetase PvdF, N5-hydroxyornithine formyltransferase	1.05
PA2398	fpvA	Outer membrane ferripyoverdine receptor FpvA	1000.00
PA2412		MbtH-like NRPS chaperone	1000.00
PA2424	pvdL	Pyoverdine chromophore precursor synthetase PvdL	0.00
PA3101	xcpT	General secretion pathway protein G	0.32
PA3104	xcpP	General secretion pathway protein C	0.00
PA3115	fimV	Probable type IV pilus assembly FimV-related transmembrane protein	0.43
PA3351	flgM	Negative regulator of flagellin synthesis FlgM (anti-sigma28)	0.44
PA3352		Flagellar biosynthesis protein FlgN	4.30
PA3805	pilF	Type IV pilus biogenesis protein PilF	0.56
PA4211	phzB1	Phenazine biosynthesis protein PhzB	1000.00
PA4230	pchB	Isochorismate pyruvate-lyase	0.69
PA4525	pilA	Type IV pilin PilA	1.21
PA4555	pilY2	Type IV fimbrial biogenesis protein PilY2	0.00
PA4953	motB	Flagellar motor rotation protein MotB	15.08
PA5042	pilO	Type IV pilus biogenesis protein PilO	0.90
PA5253	algP	Alginate regulatory protein AlgP	0.07
PA5255	algQ	Alginate regulatory protein AlgQ	1000.00
PA5322	algC	Phosphoglucomutase	1000.00

**Table 3:** Identified proteins associated with antibiotic resistance of biofilm cells grown with control and antibiotic-impregnated catheters.

Locus Tag	Gene	Product	Fold Ratio
PA0156		Multidrug efflux system, membrane fusion component	0.97
PA0427	oprM	Multidrug efflux system, outer membrane factor lipoprotein	1000.00
PA1777	oprF	Nonspecific porin and structural outer membrane protein OprF	1.29
PA4248	rplF	LSU ribosomal protein L6p (L9e)	1.69
PA4264	rpsJ	SSU ribosomal protein S10p (S20e)	2.17
PA4266	fusA1	Translation elongation factor G	4.60
PA4268	rpsL	SSU ribosomal protein S12p (S23e)	1000.00
PA4269	rpoC	DNA-directed RNA polymerase beta subunit	2.68
PA4277	tufB	Translation elongation factor Tu	2.06
PA4974		Outer membrane channel TolC (OpmH)	1.13
PA5239	rho	Transcription termination factor Rho	0.08

**Table 4:** Identified proteins associated with virulence of biofilm cells grown with control and antibiotic-impregnated catheters.

Locus Tag	Gene	Product	Fold Ratio
PA0044	exoT	Hypothetical protein	1000.00
PA0070		Autotransporter adhesin	0.69
PA0083		T6SS component TssB (ImpB/VipA)	0.00
PA0408	pilG	Twitching motility protein PilG	0.77
PA0409	pilH	Twitching motility protein PilH	0.66
PA0411	pilJ	Twitching motility protein PilJ	0.92
PA0413	chpA	Still frameshift probable component of chemotactic signal transduction system	0.27
PA0763	mucA	Sigma factor RpoE negative regulatory protein RseA	0.00
PA0766	mucD	HtrA protease/chaperone protein	2.88
PA1086	flgK	Flagellar hook-associated protein FlgK	0.40
PA1087	flgL	Flagellar hook-associated protein FlgL	0.95
PA1092	fliC	Flagellin protein FlaB	1.67
PA1094	fliD	Flagellar cap protein FliD	0.74
PA1096		Two-component system sensor histidine kinase	1.29
PA1097	fleQ	Flagellar regulatory protein FleQ	0.73
PA1101	fliF	Flagellar M-ring protein FliF	0.00
PA1103	fliH	Flagellar assembly protein FliH	0.77
PA1444	fliN	Flagellar motor switch protein FliN	1000.00
PA1707	pcrH	Type III secretion chaperone protein for YopD (SycD)	2.54
PA1708	popB	Type III secretion host injection protein (YopB)	0.00
PA1709	popD	Type III secretion host injection and negative regulator protein (YopD)	0.41
PA1722	pscI	Type III secretion cytoplasmic protein (YscI)	1000.00
PA2385	pvdQ	Acyl-homoserine lactone acylase PvdQ	0.96
PA2393		Putative dipeptidase	4.00
PA2395	pvdO	PvdO, pyoverdine responsive serine/threonine kinase	0.59
PA2396	pvdF	Pyoverdine synthetase PvdF, N5-hydroxyornithine formyltransferase	5.19
PA2412		MbtH-like NRPS chaperone	1.69
PA2413	pvdH	Pyoverdin biosynthesis protein PvdH	1.18
PA3101	xcpT	General secretion pathway protein G	1.31
PA3101	xcpT	General secretion pathway protein G	1.31
PA3104	xcpP	General secretion pathway protein C	0.00
PA3115	fimV	Probable type IV pilus assembly FimV-related transmembrane protein	0.69
PA3351	flgM	Negative regulator of flagellin synthesis FlgM (anti-sigma28)	0.81
PA3352		Flagellar biosynthesis protein FlgN	0.07
PA3805	pilF	Type IV pilus biogenesis protein PilF	0.18
PA4211	phzB1	Phenazine biosynthesis protein PhzB	1000.00
PA4226	pchE	Dihydroaeruginoate synthetase PchE	0.00
PA4230	pchB	Isochorismate pyruvate-lyase	0.75
PA4525	pilA	Type IV pilin PilA	21.73
PA4550	fimU	type 4 fimbrial biogenesis protein FimU	0.00
PA4553	pilX	Type IV fimbrial biogenesis protein PilX	0.00
PA4953	motB	Flagellar motor rotation protein MotB	0.00
PA5041	pilP	Type IV pilus biogenesis protein PilP	1.31
PA5042	pilO	Type IV pilus biogenesis protein PilO	0.00
PA5253	algP	Alginate regulatory protein AlgP	1.29

## Data Availability

https://www.mdpi.com/ethics.

## Supplementary Materials

The following are available online at https://www.mdpi.com/article/10.3390/antibiotics10070752/s1, Figure S1. Top 25 KEGG pathways of up-regulated proteins from the planktonic cells grown in control and antibiotic-impregnated catheters, Figure S2. Top 25 KEGG pathways of down-regulated proteins from the planktonic cells grown in control and antibiotic-impregnated catheters, Figure S3. Top 25 KEGG pathways of up-regulated proteins from the biofilm cells grown in control and antibiotic-impregnated catheters, Figure S4. Top 25 KEGG pathways of down-regulated proteins from the biofilm cells grown in control and antibiotic-impregnated catheters, Table S1. Minimum inhibitory concentration (MIC) of planktonic and biofilm cells grown with control and antibiotic-impregnated catheters in the continuous culture system, Table S2. Differentially regulated proteins of planktonic cells grown with control (CP) and antibiotic-coated (AP) catheters, Table S3. Differentially regulated proteins of biofilm cells grown with control (CB) and antibiotic-coated (AB) catheters, Table S4. KEGG pathways of up-regulated proteins from the planktonic cells grown in control and antibiotic-impregnated catheters, Table S5. KEGG pathways of down-regulated proteins from the planktonic cells grown in control and antibiotic-impregnated catheters, Table S6. KEGG pathways of up-regulated proteins from the biofilm cells grown in control and antibiotic-impregnated catheters.
