# Supplementary material for: Dynamic Adaptive Response of Pseudomonas aeruginosa to Clindamycin/Rifampicin-Impregnated Catheters
Source: Antibiotics (Basel). 2021 Jun 22;10(7):752. doi: 10.3390/antibiotics10070752 (PMC8300626; doi:10.3390/antibiotics10070752)

Table. S1. Minimum inhibitory concentration (MIC) of planktonic and biofilm cells grown with control and antibiotic-impregnated catheters in the continuous culture system

	**Control catheter**		**Antibiotic catheter**	
	**Planktonic**	**Biofilm**	**Planktonic**	**Biofilm**
Amikacin	≤2 (S)	≤2 (S)	4 (S)	≤2 (S)
Cefazolin	≥64 (R)	≥64 (R)	≥64 (R)	≥64 (R)
Cefepime	≤1 (S)	≤1 (S)	≤1 (S)	≤1 (S)
Cefotaxime	8 (S)	8 (S)	8 (S)	8 (S)
Ceftazidime	≤1 (S)	≤1 (S)	≤1 (S)	≤1 (S)
Ciprofloxacin	≤0.25	≤0.25	≤0.25	≤0.25
Clindamycin	512 (R)	512 (R)	512 (R)	512 (R)
Doripenem	0.5 (S)	0.5 (S)	0.5 (S)	0.5 (S)
Gentamicin	≤1 (S)	≤1 (S)	≤1 (S)	≤1 (S)
Imipenem	1 (S)	1 (S)	0.5 (S)	0.5 (S)
Levofloxacin	0.5 (S)	0.5 (S)	0.5 (S)	0.5 (S)
Meropenem	1 (S)	0.5 (S)	0.5 (S)	0.5 (S)
Norfloxacin	≤0.5 (S)	≤0.5 (S)	≤0.5 (S)	≤0.5 (S)
Piperacillin	≤4 (S)	≤4 (S)	≤4 (S)	≤4 (S)
Piperacillin/tazobactam	≤4 (S)	≤4 (S)	≤4 (S)	≤4 (S)
Rifampicin	32 (R)	32 (R)	32 (R)	32 (R)
Tigecycline	≥8 (R)	≥8 (R)	≥8 (R)	≥8 (R)
Tobramycin	≤1 (S)	≤1 (S)	≤1 (S)	≤1 (S)

Table S2. Differentially regulated proteins of planktonic cells grown with control (CP) and antibiotic-coated (AP) catheters

Locus Tag	Gene Name	Product Name	N_C-P	N_A-P	Fold Ratio	COG ID	CODE	COG Function	KOID	Functional Category
PA0002	dnaN	"DNA polymerase III	0	20389248	1000	COG0592	L	DNA polymerase sliding clamp subunit (PCNA homolog)	K02338	INFORMATION STORAGE AND PROCESSING
PA0002	dnaN	"DNA polymerase III	0	20389248	1000	COG0592	L	DNA polymerase sliding clamp subunit (PCNA homolog)	K04802	INFORMATION STORAGE AND PROCESSING
PA0008	glyS	glycyl-tRNA synthetase beta chain	0	60105885	1000	COG0751	J	"Glycyl-tRNA synthetase, beta subunit"	K01879	INFORMATION STORAGE AND PROCESSING
PA0019	def	polypeptide deformylase	0	50940599	1000	COG0242	J	N-formylmethionyl-tRNA deformylase	K01462	INFORMATION STORAGE AND PROCESSING
PA0039		hypothetical protein	1.32E+08	1.99E+08	1.501851					
PA0055		hypothetical protein	12859631	5.91E+08	45.93392	COG3812	S	Uncharacterized protein conserved in bacteria	K09983	POORLY CHARACTERIZED
PA0060		conserved hypothetical protein	46835654	0	0					
PA0068		hypothetical protein	87949650	0	0	COG3529	R	Predicted nucleic-acid-binding protein containing a Zn-ribbon	K07070	POORLY CHARACTERIZED
PA0070	tagQ1	TagQ1	4.51E+08	2.07E+08	0.458318					
PA0083	tssB1	TssB1	1.17E+08	32861605	0.282043	COG3516	S	Uncharacterized protein conserved in bacteria	K11901	POORLY CHARACTERIZED
PA0102		probable carbonic anhydrase	21237673	67188705	3.163657	COG0288	P	Carbonic anhydrase	K01673	METABOLISM
PA0103		probable sulfate transporter	3.85E+08	4.17E+08	1.081551	COG0659	P	Sulfate permease and related transporters (MFS superfamily)	K03321	METABOLISM
PA0125		ParD antitoxin	22238845	0	0					
PA0128		conserved hypothetical protein	6.65E+08	5.32E+08	0.800077	COG2824	P	Uncharacterized Zn-ribbon-containing protein involved in pho	K06193	METABOLISM
PA0139	ahpC	alkyl hydroperoxide reductase subunit C	4.1E+09	8.75E+09	2.132725	COG0450	O	Peroxiredoxin	K03386	CELLULAR PROCESSES AND SIGNALING
PA0141		conserved hypothetical protein	1.21E+09	6.74E+08	0.554969					
PA0141		conserved hypothetical protein	5.84E+08	1.22E+08	0.209261					
PA0143	nuh	purine nucleosidase Nuh	0	30771462	1000	COG1957	F	Inosine-uridine nucleoside N-ribohydrolase	K01239	METABOLISM
PA0143	nuh	purine nucleosidase Nuh	0	30771462	1000	COG1957	F	Inosine-uridine nucleoside N-ribohydrolase	K01250	METABOLISM
PA0156	triA	"Resistance-Nodulation-Cell Division (RND) triclosan efflux memb	1.71E+08	1.65E+08	0.96745	COG0845	M	Membrane-fusion protein	K02022	CELLULAR PROCESSES AND SIGNALING
PA0156	triA	"Resistance-Nodulation-Cell Division (RND) triclosan efflux memb	1.71E+08	1.65E+08	0.96745	COG0845	M	Membrane-fusion protein	K02005	CELLULAR PROCESSES AND SIGNALING
PA0156	triA	"Resistance-Nodulation-Cell Division (RND) triclosan efflux memb	1.71E+08	1.65E+08	0.96745	COG0845	M	Membrane-fusion protein	K01993	CELLULAR PROCESSES AND SIGNALING
PA0156	triA	"Resistance-Nodulation-Cell Division (RND) triclosan efflux memb	1.71E+08	1.65E+08	0.96745	COG0845	M	Membrane-fusion protein	K03585	CELLULAR PROCESSES AND SIGNALING
PA0169	siaD	SiaD	1.99E+09	8.27E+08	0.415427	COG3706	T	Response regulator containing a CheY-like receiver domain an	K02488	CELLULAR PROCESSES AND SIGNALING
PA0170		hypothetical protein	73438381	0	0					
PA0180	cttP	"chemotactic transducer for trichloroethylene [positive chemotax	0	6.58E+08	1000	COG0840	N	Methyl-accepting chemotaxis protein	K03406	CELLULAR PROCESSES AND SIGNALING
PA0180	cttP	"chemotactic transducer for trichloroethylene [positive chemotax	0	6.58E+08	1000	COG0840	T	Methyl-accepting chemotaxis protein	K03406	CELLULAR PROCESSES AND SIGNALING
PA0265	davD	glutaric semialdehyde dehydrogenase	0	98013613	1000	COG1012	C	NAD-dependent aldehyde dehydrogenases	K00135	METABOLISM
PA0265	davD	glutaric semialdehyde dehydrogenase	0	98013613	1000	COG1012	C	NAD-dependent aldehyde dehydrogenases	K00128	METABOLISM
PA0265	davD	glutaric semialdehyde dehydrogenase	0	98013613	1000	COG1012	C	NAD-dependent aldehyde dehydrogenases	K00130	METABOLISM
PA0265	davD	glutaric semialdehyde dehydrogenase	0	98013613	1000	COG1012	C	NAD-dependent aldehyde dehydrogenases	K00140	METABOLISM
PA0266	davT	delta-aminovalerate aminotransferase	0	1.22E+08	1000	COG0160	E	4-aminobutyrate aminotransferase and related aminotransfer	K00823	METABOLISM
PA0266	davT	delta-aminovalerate aminotransferase	0	1.22E+08	1000	COG0160	E	4-aminobutyrate aminotransferase and related aminotransfer	K07250	METABOLISM
PA0266	davT	delta-aminovalerate aminotransferase	0	1.22E+08	1000	COG0160	E	4-aminobutyrate aminotransferase and related aminotransfer	K00836	METABOLISM
PA0267		hypothetical protein	0	1.46E+08	1000	COG0784	T	FOG: CheY-like receiver	K03413	CELLULAR PROCESSES AND SIGNALING
PA0291	oprE	Anaerobically-induced outer membrane porin OprE precursor	13774199	0	0					
PA0295		probable periplasmic polyamine binding protein	17894714	0	0	COG0687	E	Spermidine/putrescine-binding periplasmic protein	K11073	METABOLISM
PA0295		probable periplasmic polyamine binding protein	17894714	0	0	COG0687	E	Spermidine/putrescine-binding periplasmic protein	K02055	METABOLISM
PA0295		probable periplasmic polyamine binding protein	17894714	0	0	COG0687	E	Spermidine/putrescine-binding periplasmic protein	K11069	METABOLISM
PA0300	spuD	polyamine transport protein	85617247	1.17E+08	1.369171	COG0687	E	Spermidine/putrescine-binding periplasmic protein	K11073	METABOLISM
PA0300	spuD	polyamine transport protein	85617247	1.17E+08	1.369171	COG0687	E	Spermidine/putrescine-binding periplasmic protein	K02055	METABOLISM
PA0300	spuD	polyamine transport protein	85617247	1.17E+08	1.369171	COG0687	E	Spermidine/putrescine-binding periplasmic protein	K11069	METABOLISM
PA0309		hypothetical protein	17717396	0	0					
PA0314		L-cysteine transporter of ABC system FliY	4.76E+08	1.64E+08	0.343572	COG0834	E	"ABC-type amino acid transport/signal transduction systems,	K02030	METABOLISM
PA0314		L-cysteine transporter of ABC system FliY	4.76E+08	1.64E+08	0.343572	COG0834	T	"ABC-type amino acid transport/signal transduction systems,	K02030	CELLULAR PROCESSES AND SIGNALING
PA0315		hypothetical protein	70521023	4.45E+08	6.310245					
PA0316	serA	PHGDH	37738148	1.27E+08	3.364903	COG0111	H	Phosphoglycerate dehydrogenase and related dehydrogenase	K12972	METABOLISM
PA0316	serA	PHGDH	37738148	1.27E+08	3.364903	COG0111	E	Phosphoglycerate dehydrogenase and related dehydrogenase	K03473	METABOLISM
PA0316	serA	PHGDH	37738148	1.27E+08	3.364903	COG0111	H	Phosphoglycerate dehydrogenase and related dehydrogenase	K00058	METABOLISM
PA0316	serA	PHGDH	37738148	1.27E+08	3.364903	COG0111	E	Phosphoglycerate dehydrogenase and related dehydrogenase	K12972	METABOLISM
PA0316	serA	PHGDH	37738148	1.27E+08	3.364903	COG0111	E	Phosphoglycerate dehydrogenase and related dehydrogenase	K00058	METABOLISM
PA0316	serA	PHGDH	37738148	1.27E+08	3.364903	COG0111	H	Phosphoglycerate dehydrogenase and related dehydrogenase	K03473	METABOLISM
PA0318		conserved hypothetical protein	2.09E+08	2.6E+08	1.243267	COG0179	Q	"2-keto-4-pentenoate hydratase/2-oxohepta-3-ene-1,7-dioic	K01555	METABOLISM
PA0318		conserved hypothetical protein	2.09E+08	2.6E+08	1.243267	COG0179	Q	"2-keto-4-pentenoate hydratase/2-oxohepta-3-ene-1,7-dioic	K05921	METABOLISM
PA0329		conserved hypothetical protein	2.75E+09	1.45E+09	0.528356	COG3422	S	Uncharacterized conserved protein	K09946	POORLY CHARACTERIZED
PA0330	rpiA	ribose 5-phosphate isomerase	1.05E+09	4.45E+08	0.421507	COG0120	G	Ribose 5-phosphate isomerase	K01807	METABOLISM
PA0355	pfpI	protease PfpI	0	46275980	1000	COG0693	R	Putative intracellular protease/amidase	K03152	POORLY CHARACTERIZED
PA0355	pfpI	protease PfpI	0	46275980	1000	COG0693	R	Putative intracellular protease/amidase	K05520	POORLY CHARACTERIZED
PA0359		hypothetical protein	21629401	81404931	3.763624					
PA0361		probable gamma-glutamyltranspeptidase precursor	23775637	26200578	1.101993	COG0405	E	Gamma-glutamyltransferase	K00681	METABOLISM
PA0363	coaD	phosphopantetheine adenylyltransferase	0	28722947	1000	COG0669	H	Phosphopantetheine adenylyltransferase	K00954	METABOLISM
PA0364	laoA	LaoA	37450094	0	0	COG2303	E	Choline dehydrogenase and related flavoproteins	K00108	METABOLISM
PA0364	laoA	LaoA	37450094	0	0	COG2303	E	Choline dehydrogenase and related flavoproteins	K03333	METABOLISM
PA0364	laoA	LaoA	37450094	0	0	COG2303	E	Choline dehydrogenase and related flavoproteins	K00119	METABOLISM
PA0371		hypothetical protein	3.27E+08	3.52E+08	1.076843	COG0612	R	Predicted Zn-dependent peptidases	K07263	POORLY CHARACTERIZED
PA0371		hypothetical protein	3.27E+08	3.52E+08	1.076843	COG0612	R	Predicted Zn-dependent peptidases	K01422	POORLY CHARACTERIZED
PA0371		hypothetical protein	3.27E+08	3.52E+08	1.076843	COG0612	R	Predicted Zn-dependent peptidases	K01412	POORLY CHARACTERIZED
PA0371		hypothetical protein	3.27E+08	3.52E+08	1.076843	COG0612	R	Predicted Zn-dependent peptidases	K01423	POORLY CHARACTERIZED
PA0372		probable zinc protease	77002810	1.2E+08	1.558446	COG0612	R	Predicted Zn-dependent peptidases	K01422	POORLY CHARACTERIZED
PA0372		probable zinc protease	77002810	1.2E+08	1.558446	COG0612	R	Predicted Zn-dependent peptidases	K01423	POORLY CHARACTERIZED
PA0372		probable zinc protease	77002810	1.2E+08	1.558446	COG0612	R	Predicted Zn-dependent peptidases	K01412	POORLY CHARACTERIZED
PA0372		probable zinc protease	77002810	1.2E+08	1.558446	COG0612	R	Predicted Zn-dependent peptidases	K07263	POORLY CHARACTERIZED
PA0373	ftsY	signal recognition particle receptor FtsY	26698447	77436625	2.900417	COG0552	U	Signal recognition particle GTPase	K03110	CELLULAR PROCESSES AND SIGNALING
PA0387		conserved hypothetical protein	92962927	0	0	COG0127	F	Xanthosine triphosphate pyrophosphatase	K01516	METABOLISM
PA0388		hypothetical protein	1.8E+08	1.44E+08	0.800565					
PA0394		conserved hypothetical protein	1.57E+08	0	0	COG0325	R	Predicted enzyme with a TIM-barrel fold	K06997	POORLY CHARACTERIZED
PA0399		cystathionine beta-synthase	0	76072303	1000	COG0517	R	FOG: CBS domain	K00088	POORLY CHARACTERIZED
PA0399		cystathionine beta-synthase	0	76072303	1000	COG0517	R	FOG: CBS domain	K06041	POORLY CHARACTERIZED
PA0399		cystathionine beta-synthase	0	76072303	1000	COG0031	E	Cysteine synthase	K12339	METABOLISM
PA0399		cystathionine beta-synthase	0	76072303	1000	COG0031	E	Cysteine synthase	K01738	METABOLISM
PA0399		cystathionine beta-synthase	0	76072303	1000	COG0031	E	Cysteine synthase	K01697	METABOLISM
PA0400		probable cystathionine gamma-lyase	0	29884999	1000	COG0626	E	Cystathionine beta-lyases/cystathionine gamma-synthases	K01760	METABOLISM
PA0400		probable cystathionine gamma-lyase	0	29884999	1000	COG0626	E	Cystathionine beta-lyases/cystathionine gamma-synthases	K10764	METABOLISM
PA0400		probable cystathionine gamma-lyase	0	29884999	1000	COG0626	E	Cystathionine beta-lyases/cystathionine gamma-synthases	K01761	METABOLISM
PA0400		probable cystathionine gamma-lyase	0	29884999	1000	COG0626	E	Cystathionine beta-lyases/cystathionine gamma-synthases	K01739	METABOLISM
PA0400		probable cystathionine gamma-lyase	0	29884999	1000	COG0626	E	Cystathionine beta-lyases/cystathionine gamma-synthases	K01758	METABOLISM
PA0403	pyrR	transcriptional regulator PyrR	91839580	1.52E+08	1.654338	COG2065	F	Pyrimidine operon attenuation protein/uracil phosphoribosylt	K02825	METABOLISM
PA0408	pilG	twitching motility protein PilG	1.23E+08	0	0	COG0745	T	Response regulators consisting of a CheY-like receiver domain	K02483	CELLULAR PROCESSES AND SIGNALING
PA0408	pilG	twitching motility protein PilG	1.23E+08	0	0	COG0745	K	Response regulators consisting of a CheY-like receiver domain	K02483	INFORMATION STORAGE AND PROCESSING
PA0409	pilH	twitching motility protein PilH	1.42E+09	1.12E+09	0.787328	COG0745	T	Response regulators consisting of a CheY-like receiver domain	K02483	CELLULAR PROCESSES AND SIGNALING
PA0409	pilH	twitching motility protein PilH	1.42E+09	1.12E+09	0.787328	COG0745	K	Response regulators consisting of a CheY-like receiver domain	K02483	INFORMATION STORAGE AND PROCESSING
PA0411	pilJ	twitching motility protein PilJ	3.88E+09	2.89E+09	0.743921	COG0840	T	Methyl-accepting chemotaxis protein	K03406	CELLULAR PROCESSES AND SIGNALING
PA0411	pilJ	twitching motility protein PilJ	3.88E+09	2.89E+09	0.743921	COG0840	N	Methyl-accepting chemotaxis protein	K03406	CELLULAR PROCESSES AND SIGNALING
PA0413	chpA	component of chemotactic signal transduction system	1.2E+08	1.2E+08	1.002058	COG2198	T	FOG: HPt domain	K07676	CELLULAR PROCESSES AND SIGNALING
PA0413	chpA	component of chemotactic signal transduction system	1.2E+08	1.2E+08	1.002058	COG2198	T	FOG: HPt domain	K07648	CELLULAR PROCESSES AND SIGNALING
PA0413	chpA	component of chemotactic signal transduction system	1.2E+08	1.2E+08	1.002058	COG2198	T	FOG: HPt domain	K07647	CELLULAR PROCESSES AND SIGNALING
PA0413	chpA	component of chemotactic signal transduction system	1.2E+08	1.2E+08	1.002058	COG2198	T	FOG: HPt domain	K07678	CELLULAR PROCESSES AND SIGNALING
PA0413	chpA	component of chemotactic signal transduction system	1.2E+08	1.2E+08	1.002058	COG0643	T	Chemotaxis protein histidine kinase and related kinases	K02487	CELLULAR PROCESSES AND SIGNALING
PA0413	chpA	component of chemotactic signal transduction system	1.2E+08	1.2E+08	1.002058	COG0643	T	Chemotaxis protein histidine kinase and related kinases	K03407	CELLULAR PROCESSES AND SIGNALING
PA0413	chpA	component of chemotactic signal transduction system	1.2E+08	1.2E+08	1.002058	COG2198	T	FOG: HPt domain	K07679	CELLULAR PROCESSES AND SIGNALING
PA0413	chpA	component of chemotactic signal transduction system	1.2E+08	1.2E+08	1.002058	COG0784	T	FOG: CheY-like receiver	K03413	CELLULAR PROCESSES AND SIGNALING
PA0413	chpA	component of chemotactic signal transduction system	1.2E+08	1.2E+08	1.002058	COG0643	N	Chemotaxis protein histidine kinase and related kinases	K06596	CELLULAR PROCESSES AND SIGNALING
PA0413	chpA	component of chemotactic signal transduction system	1.2E+08	1.2E+08	1.002058	COG0643	T	Chemotaxis protein histidine kinase and related kinases	K06596	CELLULAR PROCESSES AND SIGNALING
PA0413	chpA	component of chemotactic signal transduction system	1.2E+08	1.2E+08	1.002058	COG0643	N	Chemotaxis protein histidine kinase and related kinases	K02487	CELLULAR PROCESSES AND SIGNALING
PA0413	chpA	component of chemotactic signal transduction system	1.2E+08	1.2E+08	1.002058	COG0643	N	Chemotaxis protein histidine kinase and related kinases	K03407	CELLULAR PROCESSES AND SIGNALING
PA0419		conserved hypothetical protein	0	52785791	1000	COG1385	S	Uncharacterized protein conserved in bacteria	K09761	POORLY CHARACTERIZED
PA0423	pasP	PasP	1.42E+09	3.5E+09	2.470571					
PA0425	mexA	Resistance-Nodulation-Cell Division (RND) multidrug efflux memb	3.12E+08	2.22E+08	0.711067	COG0845	M	Membrane-fusion protein	K02022	CELLULAR PROCESSES AND SIGNALING
PA0425	mexA	Resistance-Nodulation-Cell Division (RND) multidrug efflux memb	3.12E+08	2.22E+08	0.711067	COG0845	M	Membrane-fusion protein	K03585	CELLULAR PROCESSES AND SIGNALING
PA0425	mexA	Resistance-Nodulation-Cell Division (RND) multidrug efflux memb	3.12E+08	2.22E+08	0.711067	COG0845	M	Membrane-fusion protein	K01993	CELLULAR PROCESSES AND SIGNALING
PA0425	mexA	Resistance-Nodulation-Cell Division (RND) multidrug efflux memb	3.12E+08	2.22E+08	0.711067	COG0845	M	Membrane-fusion protein	K02005	CELLULAR PROCESSES AND SIGNALING
PA0427	oprM	Major intrinsic multiple antibiotic resistance efflux outer membra	1.28E+08	2.35E+09	18.38197	COG1538	M	Outer membrane protein	K12340	CELLULAR PROCESSES AND SIGNALING
PA0427	oprM	Major intrinsic multiple antibiotic resistance efflux outer membra	1.28E+08	2.35E+09	18.38197	COG1538	U	Outer membrane protein	K12340	CELLULAR PROCESSES AND SIGNALING
PA0427	oprM	Major intrinsic multiple antibiotic resistance efflux outer membra	1.28E+08	2.35E+09	18.38197	COG1538	M	Outer membrane protein	K03287	CELLULAR PROCESSES AND SIGNALING
PA0427	oprM	Major intrinsic multiple antibiotic resistance efflux outer membra	1.28E+08	2.35E+09	18.38197	COG1538	U	Outer membrane protein	K03287	CELLULAR PROCESSES AND SIGNALING
PA0432	sahH	S-adenosyl-L-homocysteine hydrolase	33905060	3.99E+08	11.77791	COG0499	H	S-adenosylhomocysteine hydrolase	K01251	METABOLISM
PA0449		hypothetical protein	6.72E+08	3.45E+08	0.513335	COG1607	I	Acyl-CoA hydrolase	K10806	METABOLISM
PA0449		hypothetical protein	6.72E+08	3.45E+08	0.513335	COG1607	I	Acyl-CoA hydrolase	K01076	METABOLISM
PA0456		probable cold-shock protein	3.03E+10	1.56E+10	0.514484	COG1278	K	Cold shock proteins	K03704	INFORMATION STORAGE AND PROCESSING

PA0459		probable ClpA/B protease ATP binding subunit	6.9E+08	5.32E+08	0.771326	COG0542	O	"ATPases with chaperone activity, ATP-binding subunit"	K11907	CELLULAR PROCESSES AND SIGNALING
PA0459		probable ClpA/B protease ATP binding subunit	6.9E+08	5.32E+08	0.771326	COG0542	O	"ATPases with chaperone activity, ATP-binding subunit"	K03696	CELLULAR PROCESSES AND SIGNALING
PA0459		probable ClpA/B protease ATP binding subunit	6.9E+08	5.32E+08	0.771326	COG0542	O	"ATPases with chaperone activity, ATP-binding subunit"	K03695	CELLULAR PROCESSES AND SIGNALING
PA0459		probable ClpA/B protease ATP binding subunit	6.9E+08	5.32E+08	0.771326	COG0542	O	"ATPases with chaperone activity, ATP-binding subunit"	K03694	CELLULAR PROCESSES AND SIGNALING
PA0460		hypothetical protein	3.62E+08	3.97E+08	1.096053					
PA0468		hypothetical protein	27589152	4.43E+08	16.04688					
PA0482	glcB	malate synthase G	0	34328605	1000	COG2225	C	Malate synthase	K01638	METABOLISM
PA0500	bioB	biotin synthase	0	15571364	1000	COG0502	H	Biotin synthase and related enzymes	K01012	METABOLISM
PA0506		probable acyl-CoA dehydrogenase	0	18133796	1000	COG1960	I	Acyl-CoA dehydrogenases	K00249	METABOLISM
PA0506		probable acyl-CoA dehydrogenase	0	18133796	1000	COG1960	I	Acyl-CoA dehydrogenases	K00257	METABOLISM
PA0518	nirM	cytochrome c-551 precursor	1.02E+08	0	0					
PA0519	nirS	nitrite reductase precursor	0	2.32E+08	1000	COG2010	C	"Cytochrome c, mono- and diheme variants"	K02275	METABOLISM
PA0519	nirS	nitrite reductase precursor	0	2.32E+08	1000	COG2010	C	"Cytochrome c, mono- and diheme variants"	K00406	METABOLISM
PA0541		hypothetical protein	1.28E+09	8.36E+08	0.655002					
PA0546	metK	methionine adenosyltransferase	2.4E+08	3.71E+08	1.546574	COG0192	H	S-adenosylmethionine synthetase	K00789	METABOLISM
PA0548	tktA	transketolase	0	70911909	1000	COG0021	G	Transketolase	K00615	METABOLISM
PA0552	pgk	phosphoglycerate kinase	94645399	1.81E+08	1.909958	COG0126	G	3-phosphoglycerate kinase	K00927	METABOLISM
PA0555	fda	"fructose-1	3.69E+08	2.92E+09	7.913151	COG0191	G	Fructose/tagatose bisphosphate aldolase	K01624	METABOLISM
PA0555	fda	"fructose-1	3.69E+08	2.92E+09	7.913151	COG0191	G	Fructose/tagatose bisphosphate aldolase	K08302	METABOLISM
PA0576	rpoD	sigma factor RpoD	8.25E+08	4.61E+08	0.558827	COG0568	K	"DNA-directed RNA polymerase, sigma subunit (sigma70/sigm	K03086	INFORMATION STORAGE AND PROCESSING
PA0576	rpoD	sigma factor RpoD	8.25E+08	4.61E+08	0.558827	COG0568	K	"DNA-directed RNA polymerase, sigma subunit (sigma70/sigm	K03087	INFORMATION STORAGE AND PROCESSING
PA0576	rpoD	sigma factor RpoD	8.25E+08	4.61E+08	0.558827	COG0568	K	"DNA-directed RNA polymerase, sigma subunit (sigma70/sigm	K03089	INFORMATION STORAGE AND PROCESSING
PA0577	dnaG	DNA primase	51501443	41766281	0.810973	COG0358	L	DNA primase (bacterial type)	K02316	INFORMATION STORAGE AND PROCESSING
PA0578		conserved hypothetical protein	17981050	0	0	COG1610	S	Uncharacterized conserved protein	K09117	POORLY CHARACTERIZED
PA0579	rpsU	30S ribosomal protein S21	1.38E+09	2.73E+09	1.982596	COG0828	J	Ribosomal protein S21	K02970	INFORMATION STORAGE AND PROCESSING
PA0587		conserved hypothetical protein	9083028	29365286	3.232984	COG2718	S	Uncharacterized conserved protein	K09786	POORLY CHARACTERIZED
PA0588		conserved hypothetical protein	1.95E+08	2.62E+08	1.343821	COG2766	T	Putative Ser protein kinase	K07180	CELLULAR PROCESSES AND SIGNALING
PA0591		conserved hypothetical protein	46039596	0	0	COG2967	P	Uncharacterized protein affecting Mg2? transport	K06195	METABOLISM
PA0594	surA	peptidyl-prolyl cis-trans isomerase SurA	6.37E+08	3.97E+08	0.623496	COG0760	O	Parvulin-like peptidyl-prolyl isomerase	K03769	CELLULAR PROCESSES AND SIGNALING
PA0594	surA	peptidyl-prolyl cis-trans isomerase SurA	6.37E+08	3.97E+08	0.623496	COG0760	O	Parvulin-like peptidyl-prolyl isomerase	K03769	CELLULAR PROCESSES AND SIGNALING
PA0594	surA	peptidyl-prolyl cis-trans isomerase SurA	6.37E+08	3.97E+08	0.623496	COG0760	O	Parvulin-like peptidyl-prolyl isomerase	K07533	CELLULAR PROCESSES AND SIGNALING
PA0594	surA	peptidyl-prolyl cis-trans isomerase SurA	6.37E+08	3.97E+08	0.623496	COG0760	O	Parvulin-like peptidyl-prolyl isomerase	K03771	CELLULAR PROCESSES AND SIGNALING
PA0594	surA	peptidyl-prolyl cis-trans isomerase SurA	6.37E+08	3.97E+08	0.623496	COG0760	O	Parvulin-like peptidyl-prolyl isomerase	K07533	CELLULAR PROCESSES AND SIGNALING
PA0594	surA	peptidyl-prolyl cis-trans isomerase SurA	6.37E+08	3.97E+08	0.623496	COG0760	O	Parvulin-like peptidyl-prolyl isomerase	K03769	CELLULAR PROCESSES AND SIGNALING
PA0594	surA	peptidyl-prolyl cis-trans isomerase SurA	6.37E+08	3.97E+08	0.623496	COG0760	O	Parvulin-like peptidyl-prolyl isomerase	K03770	CELLULAR PROCESSES AND SIGNALING
PA0594	surA	peptidyl-prolyl cis-trans isomerase SurA	6.37E+08	3.97E+08	0.623496	COG0760	O	Parvulin-like peptidyl-prolyl isomerase	K03769	CELLULAR PROCESSES AND SIGNALING
PA0594	surA	peptidyl-prolyl cis-trans isomerase SurA	6.37E+08	3.97E+08	0.623496	COG0760	O	Parvulin-like peptidyl-prolyl isomerase	K03770	CELLULAR PROCESSES AND SIGNALING
PA0594	surA	peptidyl-prolyl cis-trans isomerase SurA	6.37E+08	3.97E+08	0.623496	COG0760	O	Parvulin-like peptidyl-prolyl isomerase	K03771	CELLULAR PROCESSES AND SIGNALING
PA0594	surA	peptidyl-prolyl cis-trans isomerase SurA	6.37E+08	3.97E+08	0.623496	COG0760	O	Parvulin-like peptidyl-prolyl isomerase	K03770	CELLULAR PROCESSES AND SIGNALING
PA0594	surA	peptidyl-prolyl cis-trans isomerase SurA	6.37E+08	3.97E+08	0.623496	COG0760	O	Parvulin-like peptidyl-prolyl isomerase	K03770	CELLULAR PROCESSES AND SIGNALING
PA0594	surA	peptidyl-prolyl cis-trans isomerase SurA	6.37E+08	3.97E+08	0.623496	COG0760	O	Parvulin-like peptidyl-prolyl isomerase	K07533	CELLULAR PROCESSES AND SIGNALING
PA0594	surA	peptidyl-prolyl cis-trans isomerase SurA	6.37E+08	3.97E+08	0.623496	COG0760	O	Parvulin-like peptidyl-prolyl isomerase	K03771	CELLULAR PROCESSES AND SIGNALING
PA0594	surA	peptidyl-prolyl cis-trans isomerase SurA	6.37E+08	3.97E+08	0.623496	COG0760	O	Parvulin-like peptidyl-prolyl isomerase	K07533	CELLULAR PROCESSES AND SIGNALING
PA0594	surA	peptidyl-prolyl cis-trans isomerase SurA	6.37E+08	3.97E+08	0.623496	COG0760	O	Parvulin-like peptidyl-prolyl isomerase	K03771	CELLULAR PROCESSES AND SIGNALING
PA0599		hypothetical protein	16335489	20413788	1.249659					
PA0654	speD	S-adenosylmethionine decarboxylase proenzyme	25883174	40189979	1.552745	COG1586	E	S-adenosylmethionine decarboxylase	K01611	METABOLISM
PA0655		hypothetical protein	8.41E+08	5.31E+08	0.631147	COG2941	H	Ubiquinone biosynthesis protein COQ7	K06134	METABOLISM
PA0659		hypothetical protein	22540394	0	0	COG4969	U	"Tfp pilus assembly protein, major pilin PilA"	K02682	CELLULAR PROCESSES AND SIGNALING
PA0659		hypothetical protein	22540394	0	0	COG4969	N	"Tfp pilus assembly protein, major pilin PilA"	K02650	CELLULAR PROCESSES AND SIGNALING
PA0659		hypothetical protein	22540394	0	0	COG4969	N	"Tfp pilus assembly protein, major pilin PilA"	K02655	CELLULAR PROCESSES AND SIGNALING
PA0659		hypothetical protein	22540394	0	0	COG4969	U	"Tfp pilus assembly protein, major pilin PilA"	K02655	CELLULAR PROCESSES AND SIGNALING
PA0659		hypothetical protein	22540394	0	0	COG4969	N	"Tfp pilus assembly protein, major pilin PilA"	K02682	CELLULAR PROCESSES AND SIGNALING
PA0659		hypothetical protein	22540394	0	0	COG4969	U	"Tfp pilus assembly protein, major pilin PilA"	K02650	CELLULAR PROCESSES AND SIGNALING
PA0660		NAD(P)H:quinone reductase	0	31816910	1000	COG2070	R	Dioxygenases related to 2-nitropropane dioxygenase	K00459	POORLY CHARACTERIZED
PA0660		NAD(P)H:quinone reductase	0	31816910	1000	COG2070	R	Dioxygenases related to 2-nitropropane dioxygenase	K02371	POORLY CHARACTERIZED
PA0664		hypothetical protein	1.68E+08	3.63E+08	2.162318					
PA0665		conserved hypothetical protein	4.52E+08	79342640	0.175348	COG0316	S	Uncharacterized conserved protein	K13628	POORLY CHARACTERIZED
PA0665		conserved hypothetical protein	4.52E+08	79342640	0.175348	COG0316	S	Uncharacterized conserved protein	K07400	POORLY CHARACTERIZED
PA0668	tyrZ	tyrosyl-tRNA synthetase 2	8.59E+08	1.14E+09	1.332015	COG0162	J	Tyrosyl-tRNA synthetase	K01866	INFORMATION STORAGE AND PROCESSING
PA0672	hemO	heme oxygenase	5.52E+08	1.31E+08	0.236952	COG3230	P	Heme oxygenase	K07215	METABOLISM
PA0720		helix destabilizing protein of bacteriophage Pf1	7.91E+08	1.03E+10	13.01363					
PA0724		probable coat protein A of bacteriophage Pf1	0	5.85E+08	1000					
PA0727		Pf replication initiator protein	1.88E+08	4.27E+08	2.264923					
PA0758		hypothetical protein	1.66E+08	77112783	0.46548					
PA0763	mucA	anti-sigma factor MucA	0	45205540	1000	COG3073	T	Negative regulator of sigma E activity	K03597	CELLULAR PROCESSES AND SIGNALING
PA0764	mucB	negative regulator for alginate biosynthesis MucB	29078665	0	0	COG3026	T	Negative regulator of sigma E activity	K03598	CELLULAR PROCESSES AND SIGNALING
PA0766	mucD	serine protease MucD precursor	4.77E+08	3.43E+08	0.718976	COG0265	O	"Trypsin-like serine proteases, typically periplasmic, contain C	K04691	CELLULAR PROCESSES AND SIGNALING
PA0766	mucD	serine protease MucD precursor	4.77E+08	3.43E+08	0.718976	COG0265	O	"Trypsin-like serine proteases, typically periplasmic, contain C	K01362	CELLULAR PROCESSES AND SIGNALING
PA0773	pdxJ	pyridoxal phosphate biosynthetic protein PdxJ	0	30233984	1000	COG0854	H	Pyridoxal phosphate biosynthesis protein	K03474	METABOLISM
PA0782	putA	proline dehydrogenase PutA	20958915	0	0	COG0506	E	Proline dehydrogenase	K13821	METABOLISM
PA0782	putA	proline dehydrogenase PutA	20958915	0	0	COG0506	E	Proline dehydrogenase	K00318	METABOLISM
PA0782	putA	proline dehydrogenase PutA	20958915	0	0	COG4230	C	Delta 1-pyrroline-5-carboxylate dehydrogenase	K13821	METABOLISM
PA0797		probable transcriptional regulator	25432012	14741705	0.579652					
PA0830		hypothetical protein	0	5.75E+08	1000	COG3687	R	Predicted metal-dependent hydrolase	K07044	POORLY CHARACTERIZED
PA0833		hypothetical protein	1.34E+09	1.81E+09	1.352414	COG2885	M	Outer membrane protein and related peptidoglycan-associate	K03286	CELLULAR PROCESSES AND SIGNALING
PA0833		hypothetical protein	1.34E+09	1.81E+09	1.352414	COG2885	M	Outer membrane protein and related peptidoglycan-associate	K03640	CELLULAR PROCESSES AND SIGNALING
PA0837	slyD	peptidyl-prolyl cis-trans isomerase SlyD	1.97E+09	3.56E+09	1.810593	COG1047	O	FKBP-type peptidyl-prolyl cis-trans isomerases 2	K03774	CELLULAR PROCESSES AND SIGNALING
PA0837	slyD	peptidyl-prolyl cis-trans isomerase SlyD	1.97E+09	3.56E+09	1.810593	COG1047	O	FKBP-type peptidyl-prolyl cis-trans isomerases 2	K03775	CELLULAR PROCESSES AND SIGNALING
PA0837	slyD	peptidyl-prolyl cis-trans isomerase SlyD	1.97E+09	3.56E+09	1.810593	COG1047	O	FKBP-type peptidyl-prolyl cis-trans isomerases 2	K01802	CELLULAR PROCESSES AND SIGNALING
PA0838		probable glutathione peroxidase	48266359	44466319	0.921269	COG0386	O	Glutathione peroxidase	K00432	CELLULAR PROCESSES AND SIGNALING
PA0854	fumC2	fumarate hydratase	4.78E+08	2.73E+08	0.570225	COG0114	C	Fumarase	K01679	METABOLISM
PA0856		hypothetical protein	2.89E+09	1.91E+09	0.659764	COG3184	S	Uncharacterized protein conserved in bacteria	K09924	POORLY CHARACTERIZED
PA0865	hpd	4-hydroxyphenylpyruvate dioxygenase	0	4.48E+08	1000	COG3185	R	4-hydroxyphenylpyruvate dioxygenase and related hemolysin	K00457	POORLY CHARACTERIZED
PA0865	hpd	4-hydroxyphenylpyruvate dioxygenase	0	4.48E+08	1000	COG3185	E	4-hydroxyphenylpyruvate dioxygenase and related hemolysin	K00457	METABOLISM
PA0867	mliC	membrane-bound lysozyme inhibitor of c-type lysozyme MliC	81047357	1.76E+08	2.170536					
PA0870	phhC	aromatic amino acid aminotransferase	0	14098689	1000	COG1448	E	Aspartate/tyrosine/aromatic aminotransferase	K00832	METABOLISM
PA0870	phhC	aromatic amino acid aminotransferase	0	14098689	1000	COG1448	E	Aspartate/tyrosine/aromatic aminotransferase	K00813	METABOLISM
PA0871	phhB	pterin-4-alpha-carbinolamine dehydratase	36923536	4.54E+08	12.30245	COG2154	H	Pterin-4a-carbinolamine dehydratase	K01724	METABOLISM
PA0887	acsA	acetyl-coenzyme A synthetase	0	40170708	1000	COG0365	I	Acyl-coenzyme A synthetases/AMP-(fatty) acid ligases	K01907	METABOLISM
PA0887	acsA	acetyl-coenzyme A synthetase	0	40170708	1000	COG0365	I	Acyl-coenzyme A synthetases/AMP-(fatty) acid ligases	K01908	METABOLISM
PA0887	acsA	acetyl-coenzyme A synthetase	0	40170708	1000	COG0365	I	Acyl-coenzyme A synthetases/AMP-(fatty) acid ligases	K01895	METABOLISM
PA0888	aotJ	arginine/ornithine binding protein AotJ	9.48E+08	5.72E+08	0.603688	COG0834	T	"ABC-type amino acid transport/signal transduction systems,	K02030	CELLULAR PROCESSES AND SIGNALING
PA0888	aotJ	arginine/ornithine binding protein AotJ	9.48E+08	5.72E+08	0.603688	COG0834	E	"ABC-type amino acid transport/signal transduction systems,	K02030	METABOLISM
PA0895	aruC	N2-Succinylornithine 5-aminotransferase (SOAT) = N2-acetylornit	0	1.4E+08	1000	COG4992	E	Ornithine/acetylornithine aminotransferase	K00821	METABOLISM
PA0895	aruC	N2-Succinylornithine 5-aminotransferase (SOAT) = N2-acetylornit	0	1.4E+08	1000	COG4992	E	Ornithine/acetylornithine aminotransferase	K00818	METABOLISM
PA0895	aruC	N2-Succinylornithine 5-aminotransferase (SOAT) = N2-acetylornit	0	1.4E+08	1000	COG4992	E	Ornithine/acetylornithine aminotransferase	K00819	METABOLISM
PA0899	aruB	N2-Succinylarginine dihydrolase	0	31477862	1000	COG3724	E	Succinylarginine dihydrolase	K01484	METABOLISM
PA0900		hypothetical protein	17060762	0	0					
PA0903	alaS	alanyl-tRNA synthetase	22414644	1.74E+08	7.749393	COG0013	J	Alanyl-tRNA synthetase	K01872	INFORMATION STORAGE AND PROCESSING
PA0905	rsmA	RsmA	3.97E+09	4.92E+09	1.238003	COG1551	T	Carbon storage regulator (could also regulate swarming and q	K03563	CELLULAR PROCESSES AND SIGNALING
PA0915		conserved hypothetical protein	53905077	0	0					
PA0919		alanyl-phosphatidylglycerol hydrolase	64866664	37689576	0.581032					
PA0932	cysM	cysteine synthase B	0	20722876	1000	COG0031	E	Cysteine synthase	K12339	METABOLISM
PA0932	cysM	cysteine synthase B	0	20722876	1000	COG0031	E	Cysteine synthase	K01738	METABOLISM
PA0932	cysM	cysteine synthase B	0	20722876	1000	COG0031	E	Cysteine synthase	K01697	METABOLISM
PA0937		conserved hypothetical protein	1.36E+09	2.9E+09	2.131733	COG3122	S	Uncharacterized protein conserved in bacteria	K09912	POORLY CHARACTERIZED
PA0938	wzz2	Wzz2	56778463	0	0	COG3765	M	Chain length determinant protein	K05789	CELLULAR PROCESSES AND SIGNALING
PA0938	wzz2	Wzz2	56778463	0	0	COG3765	M	Chain length determinant protein	K05790	CELLULAR PROCESSES AND SIGNALING
PA0941		hypothetical protein	28697305	1.66E+08	5.772694	COG0695	O	Glutaredoxin and related proteins	K03676	CELLULAR PROCESSES AND SIGNALING
PA0941		hypothetical protein	28697305	1.66E+08	5.772694	COG0695	O	Glutaredoxin and related proteins	K03674	CELLULAR PROCESSES AND SIGNALING
PA0941		hypothetical protein	28697305	1.66E+08	5.772694	COG0695	O	Glutaredoxin and related proteins	K06191	CELLULAR PROCESSES AND SIGNALING
PA0945	purM	phosphoribosylaminoimidazole synthetase	2.14E+08	1.22E+08	0.56878	COG0150	F	Phosphoribosylaminoimidazole (AIR) synthetase	K01933	METABOLISM
PA0946		hypothetical protein	7.21E+08	5.39E+08	0.747082	COG3249	S	Uncharacterized protein conserved in bacteria	K09938	POORLY CHARACTERIZED
PA0950		probable arsenate reductase	2.18E+09	7.54E+08	0.345596	COG1393	P	"Arsenate reductase and related proteins, glutaredoxin family	K00537	METABOLISM
PA0953		probable thioredoxin	1.68E+08	1.12E+08	0.666805	COG0526	O	Thiol-disulfide isomerase and thioredoxins	K03671	CELLULAR PROCESSES AND SIGNALING
PA0953		probable thioredoxin	1.68E+08	1.12E+08	0.666805	COG0526	C	Thiol-disulfide isomerase and thioredoxins	K03673	METABOLISM
PA0953		probable thioredoxin	1.68E+08	1.12E+08	0.666805	COG0526	C	Thiol-disulfide isomerase and thioredoxins	K03671	METABOLISM
PA0953		probable thioredoxin	1.68E+08	1.12E+08	0.666805	COG0526	O	Thiol-disulfide isomerase and thioredoxins	K03673	CELLULAR PROCESSES AND SIGNALING
PA0956	proS	prolyl-tRNA synthetase	0	52190952	1000	COG0442	J	Prolyl-tRNA synthetase	K01881	INFORMATION STORAGE AND PROCESSING
PA0958	oprD	"Basic amino acid	59789753	7.87E+08	13.15548					
PA0960		hypothetical protein	1.32E+08	80158343	0.606412	COG2900	S	Uncharacterized protein conserved in bacteria	K03745	POORLY CHARACTERIZED

PA0962	dps	"DNA-binding protein from starved cells	1.02E+09	1.67E+09	1.641916	COG0783	P	DNA-binding ferritin-like protein (oxidative damage protectan	K04047	METABOLISM
PA0964	pmpR	"pqsR-mediated PQS regulator	1.18E+08	5.6E+08	4.739545					
PA0969	tolQ	TolQ protein	4.46E+08	3.67E+08	0.823542	COG0811	U	Biopolymer transport proteins	K03562	CELLULAR PROCESSES AND SIGNALING
PA0969	tolQ	TolQ protein	4.46E+08	3.67E+08	0.823542	COG0811	U	Biopolymer transport proteins	K03561	CELLULAR PROCESSES AND SIGNALING
PA0970	tolR	TolR protein	6.19E+08	5.77E+08	0.932034	COG0848	U	Biopolymer transport protein	K03560	CELLULAR PROCESSES AND SIGNALING
PA0970	tolR	TolR protein	6.19E+08	5.77E+08	0.932034	COG0848	U	Biopolymer transport protein	K03559	CELLULAR PROCESSES AND SIGNALING
PA0971	tolA	TolA protein	27232012	0	0	COG0810	M	"Periplasmic protein TonB, links inner and outer membranes"	K03832	CELLULAR PROCESSES AND SIGNALING
PA0973	oprL	Peptidoglycan associated lipoprotein OprL precursor	2.26E+10	2.29E+10	1.014878	COG2885	M	Outer membrane protein and related peptidoglycan-associate	K03640	CELLULAR PROCESSES AND SIGNALING
PA0973	oprL	Peptidoglycan associated lipoprotein OprL precursor	2.26E+10	2.29E+10	1.014878	COG2885	M	Outer membrane protein and related peptidoglycan-associate	K03286	CELLULAR PROCESSES AND SIGNALING
PA0974		conserved hypothetical protein	9.9E+08	7.5E+08	0.757178					
PA0991	hptA	Histidine phosphotransfer protein HptA	0	90308703	1000					
PA0997	pqsB	PqsB	1.09E+08	2.51E+08	2.293008	COG0332	I	3-oxoacyl-[acyl-carrier-protein] synthase III	K00648	METABOLISM
PA1006		Protein PA1006	3.95E+08	2.97E+08	0.750135	COG0425	O	"Predicted redox protein, regulator of disulfide bond formatio	K04085	CELLULAR PROCESSES AND SIGNALING
PA1008	bcp	bacterioferritin comigratory protein	6.94E+08	1.46E+08	0.210822	COG1225	O	Peroxiredoxin	K03564	CELLULAR PROCESSES AND SIGNALING
PA1010	dapA	dihydrodipicolinate synthase	5.27E+08	8.13E+08	1.540852	COG0329	E	Dihydrodipicolinate synthase/N-acetylneuraminate lyase	K01714	METABOLISM
PA1010	dapA	dihydrodipicolinate synthase	5.27E+08	8.13E+08	1.540852	COG0329	E	Dihydrodipicolinate synthase/N-acetylneuraminate lyase	K01639	METABOLISM
PA1010	dapA	dihydrodipicolinate synthase	5.27E+08	8.13E+08	1.540852	COG0329	M	Dihydrodipicolinate synthase/N-acetylneuraminate lyase	K01714	CELLULAR PROCESSES AND SIGNALING
PA1010	dapA	dihydrodipicolinate synthase	5.27E+08	8.13E+08	1.540852	COG0329	M	Dihydrodipicolinate synthase/N-acetylneuraminate lyase	K01639	CELLULAR PROCESSES AND SIGNALING
PA1011		hypothetical protein	8.51E+09	7.8E+09	0.91615	COG3317	M	Uncharacterized lipoprotein	K07287	CELLULAR PROCESSES AND SIGNALING
PA1013	purC	phosphoribosylaminoimidazole-succinocarboxamide synthase	2.38E+08	7.85E+08	3.293792	COG0152	F	Phosphoribosylaminoimidazolesuccinocarboxamide (SAICAR)	K01923	METABOLISM
PA1031		conserved hypothetical protein	0	76136891	1000	COG1322	S	Uncharacterized protein conserved in bacteria	K09760	POORLY CHARACTERIZED
PA1036		hypothetical protein	0	26315360	1000					
PA1041		probable outer membrane protein precursor	5.35E+08	8.52E+08	1.593352	COG2885	M	Outer membrane protein and related peptidoglycan-associate	K03640	CELLULAR PROCESSES AND SIGNALING
PA1041		probable outer membrane protein precursor	5.35E+08	8.52E+08	1.593352	COG2885	M	Outer membrane protein and related peptidoglycan-associate	K03286	CELLULAR PROCESSES AND SIGNALING
PA1049	pdxH	pyridoxine 5'-phosphate oxidase	31707612	3.25E+08	10.26288	COG0259	H	Pyridoxamine-phosphate oxidase	K00275	METABOLISM
PA1053		conserved hypothetical protein	1.02E+09	7.23E+08	0.70647	COG3133	M	Outer membrane lipoprotein	K06077	CELLULAR PROCESSES AND SIGNALING
PA1054	shaA	ShaA(Probable NADH dehydrogenase)	0	41885219	1000	COG1009	C	NADH:ubiquinone oxidoreductase subunit 5 (chain L)/Multisu	K05577	METABOLISM
PA1054	shaA	ShaA(Probable NADH dehydrogenase)	0	41885219	1000	COG1009	P	NADH:ubiquinone oxidoreductase subunit 5 (chain L)/Multisu	K05565	METABOLISM
PA1054	shaA	ShaA(Probable NADH dehydrogenase)	0	41885219	1000	COG2111	P	"Multisubunit Na? antiporter, MnhB subunit"	K05565	METABOLISM
PA1054	shaA	ShaA(Probable NADH dehydrogenase)	0	41885219	1000	COG1009	P	NADH:ubiquinone oxidoreductase subunit 5 (chain L)/Multisu	K05559	METABOLISM
PA1054	shaA	ShaA(Probable NADH dehydrogenase)	0	41885219	1000	COG2111	P	"Multisubunit Na? antiporter, MnhB subunit"	K05559	METABOLISM
PA1054	shaA	ShaA(Probable NADH dehydrogenase)	0	41885219	1000	COG1009	C	NADH:ubiquinone oxidoreductase subunit 5 (chain L)/Multisu	K00341	METABOLISM
PA1054	shaA	ShaA(Probable NADH dehydrogenase)	0	41885219	1000	COG1009	C	NADH:ubiquinone oxidoreductase subunit 5 (chain L)/Multisu	K05565	METABOLISM
PA1054	shaA	ShaA(Probable NADH dehydrogenase)	0	41885219	1000	COG2111	P	"Multisubunit Na? antiporter, MnhB subunit"	K05566	METABOLISM
PA1054	shaA	ShaA(Probable NADH dehydrogenase)	0	41885219	1000	COG1009	P	NADH:ubiquinone oxidoreductase subunit 5 (chain L)/Multisu	K05577	METABOLISM
PA1054	shaA	ShaA(Probable NADH dehydrogenase)	0	41885219	1000	COG1009	P	NADH:ubiquinone oxidoreductase subunit 5 (chain L)/Multisu	K00341	METABOLISM
PA1054	shaA	ShaA(Probable NADH dehydrogenase)	0	41885219	1000	COG1009	C	NADH:ubiquinone oxidoreductase subunit 5 (chain L)/Multisu	K05559	METABOLISM
PA1064		hypothetical protein	3.88E+08	2.22E+08	0.573276	COG3650	S	Predicted membrane protein	K08985	POORLY CHARACTERIZED
PA1074	braC	branched-chain amino acid transport protein BraC	1.07E+09	5.51E+08	0.513928	COG0683	E	"ABC-type branched-chain amino acid transport systems, peri	K01999	METABOLISM
PA1076		hypothetical protein	2.3E+09	2.74E+09	1.193768					
PA1086	flgK	flagellar hook-associated protein 1 FlgK	2.67E+08	0	0	COG1256	N	Flagellar hook-associated protein	K02396	CELLULAR PROCESSES AND SIGNALING
PA1087	flgL	flagellar hook-associated protein type 3 FlgL	68489365	17123427	0.250016	COG1344	N	Flagellin and related hook-associated proteins	K02406	CELLULAR PROCESSES AND SIGNALING
PA1087	flgL	flagellar hook-associated protein type 3 FlgL	68489365	17123427	0.250016	COG1344	N	Flagellin and related hook-associated proteins	K02397	CELLULAR PROCESSES AND SIGNALING
PA1092	fliC	flagellin type B	2.9E+09	2.03E+10	7.005868	COG1344	N	Flagellin and related hook-associated proteins	K02406	CELLULAR PROCESSES AND SIGNALING
PA1092	fliC	flagellin type B	2.9E+09	2.03E+10	7.005868	COG1344	N	Flagellin and related hook-associated proteins	K02397	CELLULAR PROCESSES AND SIGNALING
PA1094	fliD	flagellar capping protein FliD	8.65E+08	1.12E+08	0.129389	COG1345	N	Flagellar capping protein	K02407	CELLULAR PROCESSES AND SIGNALING
PA1096		hypothetical protein	2.14E+08	0	0					
PA1097	fleQ	transcriptional regulator FleQ	1.36E+08	28666369	0.210398	COG2204	T	"Response regulator containing CheY-like receiver, AAA-type /	K07712	CELLULAR PROCESSES AND SIGNALING
PA1097	fleQ	transcriptional regulator FleQ	1.36E+08	28666369	0.210398	COG2204	T	"Response regulator containing CheY-like receiver, AAA-type /	K02481	CELLULAR PROCESSES AND SIGNALING
PA1097	fleQ	transcriptional regulator FleQ	1.36E+08	28666369	0.210398	COG2204	T	"Response regulator containing CheY-like receiver, AAA-type /	K10126	CELLULAR PROCESSES AND SIGNALING
PA1101	fliF	Flagella M-ring outer membrane protein precursor	22290950	0	0	COG1766	N	Flagellar biosynthesis/type III secretory pathway lipoprotein	K02409	CELLULAR PROCESSES AND SIGNALING
PA1101	fliF	Flagella M-ring outer membrane protein precursor	22290950	0	0	COG1766	U	Flagellar biosynthesis/type III secretory pathway lipoprotein	K02409	CELLULAR PROCESSES AND SIGNALING
PA1103		probable flagellar assembly protein	6.47E+08	39800345	6.15E-02	COG1317	U	Flagellar biosynthesis/type III secretory pathway protein	K03223	CELLULAR PROCESSES AND SIGNALING
PA1103		probable flagellar assembly protein	6.47E+08	39800345	6.15E-02	COG1317	U	Flagellar biosynthesis/type III secretory pathway protein	K02411	CELLULAR PROCESSES AND SIGNALING
PA1103		probable flagellar assembly protein	6.47E+08	39800345	6.15E-02	COG1317	N	Flagellar biosynthesis/type III secretory pathway protein	K02411	CELLULAR PROCESSES AND SIGNALING
PA1103		probable flagellar assembly protein	6.47E+08	39800345	6.15E-02	COG1317	N	Flagellar biosynthesis/type III secretory pathway protein	K03223	CELLULAR PROCESSES AND SIGNALING
PA1104	fliI	flagellum-specific ATP synthase FliI	35239777	0	0	COG1157	U	Flagellar biosynthesis/type III secretory pathway ATPase	K03224	CELLULAR PROCESSES AND SIGNALING
PA1104	fliI	flagellum-specific ATP synthase FliI	35239777	0	0	COG1157	N	Flagellar biosynthesis/type III secretory pathway ATPase	K02412	CELLULAR PROCESSES AND SIGNALING
PA1104	fliI	flagellum-specific ATP synthase FliI	35239777	0	0	COG1157	U	Flagellar biosynthesis/type III secretory pathway ATPase	K02412	CELLULAR PROCESSES AND SIGNALING
PA1104	fliI	flagellum-specific ATP synthase FliI	35239777	0	0	COG1157	N	Flagellar biosynthesis/type III secretory pathway ATPase	K03224	CELLULAR PROCESSES AND SIGNALING
PA1119	yfiB	YfiB	59742564	4.38E+08	7.33421	COG2885	M	Outer membrane protein and related peptidoglycan-associate	K03640	CELLULAR PROCESSES AND SIGNALING
PA1119	yfiB	YfiB	59742564	4.38E+08	7.33421	COG2885	M	Outer membrane protein and related peptidoglycan-associate	K03286	CELLULAR PROCESSES AND SIGNALING
PA1123		hypothetical protein	2.31E+08	1.72E+08	0.745049					
PA1127		probable oxidoreductase	99885150	62740281	0.628124					
PA1151	imm2	pyocin S2 immunity protein	1.45E+09	1.18E+09	0.818264					
PA1154		conserved hypothetical protein	0	2.48E+08	1000	COG1943	L	Transposase and inactivated derivatives	K07491	INFORMATION STORAGE AND PROCESSING
PA1155	nrdB	"NrdB	6.22E+08	7.25E+08	1.165097	COG0208	F	"Ribonucleotide reductase, beta subunit"	K10808	METABOLISM
PA1155	nrdB	"NrdB	6.22E+08	7.25E+08	1.165097	COG0208	F	"Ribonucleotide reductase, beta subunit"	K00526	METABOLISM
PA1156	nrdA	"NrdA	3.07E+08	5.58E+08	1.814538	COG0209	F	"Ribonucleotide reductase, alpha subunit"	K00525	METABOLISM
PA1159		probable cold-shock protein	1.27E+10	1.46E+10	1.147181	COG1278	K	Cold shock proteins	K03704	INFORMATION STORAGE AND PROCESSING
PA1160		hypothetical protein	17271686	0	0					
PA1163	ndvB	NdvB(encodes a glucosyltransferase enzyme that catalyzes the syr	0	1.21E+08	1000	COG1215	M	"Glycosyltransferases, probably involved in cell wall biogenesi	K00694	CELLULAR PROCESSES AND SIGNALING
PA1163	ndvB	NdvB(encodes a glucosyltransferase enzyme that catalyzes the syr	0	1.21E+08	1000	COG1215	M	"Glycosyltransferases, probably involved in cell wall biogenesi	K00720	CELLULAR PROCESSES AND SIGNALING
PA1163	ndvB	NdvB(encodes a glucosyltransferase enzyme that catalyzes the syr	0	1.21E+08	1000	COG5309	G	"Exo-beta-1,3-glucanase"	K01210	METABOLISM
PA1178	oprH	PhoP/Q and low Mg2+ inducible outer membrane protein H1 pre	0	2.34E+08	1000	COG3637	M	Opacity protein and related surface antigens	K11934	CELLULAR PROCESSES AND SIGNALING
PA1203		hypothetical protein	2.18E+08	27366680	0.125694	COG1765	O	"Predicted redox protein, regulator of disulfide bond formatio	K07397	CELLULAR PROCESSES AND SIGNALING
PA1203		hypothetical protein	2.18E+08	27366680	0.125694	COG1765	O	"Predicted redox protein, regulator of disulfide bond formatio	K06889	CELLULAR PROCESSES AND SIGNALING
PA1244	qslA	QslA	3.04E+08	0	0					
PA1250	aprI	alkaline proteinase inhibitor AprI	63675483	30803831	0.483763					
PA1272	cobO	cob(I)alamin adenosyltransferase	38197800	13715664	0.359069	COG2109	H	ATP:corrinoid adenosyltransferase	K00798	METABOLISM
PA1295		conserved hypothetical protein	53598523	0	0	COG3100	S	Uncharacterized protein conserved in bacteria	K09902	POORLY CHARACTERIZED
PA1323		hypothetical protein	4.48E+09	1.65E+09	0.367553	COG4575	S	Uncharacterized conserved protein	K05594	POORLY CHARACTERIZED
PA1324		hypothetical protein	1.73E+08	1.63E+08	0.941313					
PA1337	ansB	glutaminase-asparaginase	94239729	2.37E+08	2.511435	COG0252	E	L-asparaginase/archaeal Glu-tRNAGln amidotransferase subur	K09482	METABOLISM
PA1337	ansB	glutaminase-asparaginase	94239729	2.37E+08	2.511435	COG0252	J	L-asparaginase/archaeal Glu-tRNAGln amidotransferase subur	K09482	INFORMATION STORAGE AND PROCESSING
PA1337	ansB	glutaminase-asparaginase	94239729	2.37E+08	2.511435	COG0252	E	L-asparaginase/archaeal Glu-tRNAGln amidotransferase subur	K01424	METABOLISM
PA1337	ansB	glutaminase-asparaginase	94239729	2.37E+08	2.511435	COG0252	J	L-asparaginase/archaeal Glu-tRNAGln amidotransferase subur	K01424	INFORMATION STORAGE AND PROCESSING
PA1338	ggt	gamma-glutamyltranspeptidase precursor	79643375	3.11E+08	3.907197	COG0405	E	Gamma-glutamyltransferase	K00681	METABOLISM
PA1342	aatJ	AatJ	1.48E+09	4.44E+08	0.298788	COG0834	T	"ABC-type amino acid transport/signal transduction systems,	K02030	CELLULAR PROCESSES AND SIGNALING
PA1342	aatJ	AatJ	1.48E+09	4.44E+08	0.298788	COG0834	E	"ABC-type amino acid transport/signal transduction systems,	K02030	METABOLISM
PA1367		hypothetical protein	0	24500279	1000	COG0384	R	"Predicted epimerase, PhzC/PhzF homolog"	K06998	POORLY CHARACTERIZED
PA1369		hypothetical protein	1.87E+10	2.67E+10	1.428502					
PA1404		hypothetical protein	2.29E+08	0	0					
PA1423	bdlA	BdlA	36477344	7.2E+08	19.74484	COG0840	N	Methyl-accepting chemotaxis protein	K03406	CELLULAR PROCESSES AND SIGNALING
PA1423	bdlA	BdlA	36477344	7.2E+08	19.74484	COG0840	T	Methyl-accepting chemotaxis protein	K03406	CELLULAR PROCESSES AND SIGNALING
PA1425		probable ATP-binding component of ABC transporter	40766241	0	0	COG0488	R	ATPase components of ABC transporters with duplicated ATPa	K06158	POORLY CHARACTERIZED
PA1429		probable cation-transporting P-type ATPase	0	36972336	1000	COG0474	P	Cation transport ATPase	K01530	METABOLISM
PA1429		probable cation-transporting P-type ATPase	0	36972336	1000	COG0474	P	Cation transport ATPase	K01552	METABOLISM
PA1429		probable cation-transporting P-type ATPase	0	36972336	1000	COG0474	P	Cation transport ATPase	K01537	METABOLISM
PA1430	lasR	transcriptional regulator LasR	19605966	6176439	0.315029	COG2771	K	DNA-binding HTH domain-containing proteins	K07782	INFORMATION STORAGE AND PROCESSING
PA1445	fliO	flagellar protein FliO	1.04E+08	0	0	COG3190	N	Flagellar biogenesis protein	K02418	CELLULAR PROCESSES AND SIGNALING
PA1456	cheY	two-component response regulator CheY	2.15E+08	2.64E+08	1.224633	COG0745	T	Response regulators consisting of a CheY-like receiver domain	K02483	CELLULAR PROCESSES AND SIGNALING
PA1456	cheY	two-component response regulator CheY	2.15E+08	2.64E+08	1.224633	COG0745	K	Response regulators consisting of a CheY-like receiver domain	K02483	INFORMATION STORAGE AND PROCESSING
PA1457	cheZ	chemotaxis protein CheZ	1.16E+09	4.63E+08	0.397888	COG3143	N	Chemotaxis protein	K03414	CELLULAR PROCESSES AND SIGNALING
PA1457	cheZ	chemotaxis protein CheZ	1.16E+09	4.63E+08	0.397888	COG3143	T	Chemotaxis protein	K03414	CELLULAR PROCESSES AND SIGNALING
PA1457	cheZ	chemotaxis protein CheZ	1.16E+09	4.63E+08	0.397888	COG3143	T	Chemotaxis protein	K03414	CELLULAR PROCESSES AND SIGNALING
PA1457	cheZ	chemotaxis protein CheZ	1.16E+09	4.63E+08	0.397888	COG3143	N	Chemotaxis protein	K03414	CELLULAR PROCESSES AND SIGNALING
PA1457	cheZ	chemotaxis protein CheZ	2.71E+09	8.66E+08	0.319565	COG3143	N	Chemotaxis protein	K03414	CELLULAR PROCESSES AND SIGNALING
PA1457	cheZ	chemotaxis protein CheZ	2.71E+09	8.66E+08	0.319565	COG3143	T	Chemotaxis protein	K03414	CELLULAR PROCESSES AND SIGNALING
PA1457	cheZ	chemotaxis protein CheZ	2.71E+09	8.66E+08	0.319565	COG3143	N	Chemotaxis protein	K03414	CELLULAR PROCESSES AND SIGNALING
PA1457	cheZ	chemotaxis protein CheZ	2.71E+09	8.66E+08	0.319565	COG3143	T	Chemotaxis protein	K03414	CELLULAR PROCESSES AND SIGNALING
PA1458		probable two-component sensor	74821539	81713567	1.092113	COG0643	N	Chemotaxis protein histidine kinase and related kinases	K03407	CELLULAR PROCESSES AND SIGNALING
PA1458		probable two-component sensor	74821539	81713567	1.092113	COG0643	T	Chemotaxis protein histidine kinase and related kinases	K02487	CELLULAR PROCESSES AND SIGNALING
PA1458		probable two-component sensor	74821539	81713567	1.092113	COG2198	T	FOG: HPt domain	K07676	CELLULAR PROCESSES AND SIGNALING
PA1458		probable two-component sensor	74821539	81713567	1.092113	COG2198	T	FOG: HPt domain	K07678	CELLULAR PROCESSES AND SIGNALING
PA1458		probable two-component sensor	74821539	81713567	1.092113	COG2198	T	FOG: HPt domain	K07679	CELLULAR PROCESSES AND SIGNALING
PA1458		probable two-component sensor	74821539	81713567	1.092113	COG0643	T	Chemotaxis protein histidine kinase and related kinases	K03407	CELLULAR PROCESSES AND SIGNALING
PA1458		probable two-component sensor	74821539	81713567	1.092113	COG2198	T	FOG: HPt domain	K07647	CELLULAR PROCESSES AND SIGNALING
PA1458		probable two-component sensor	74821539	81713567	1.092113	COG0643	T	Chemotaxis protein histidine kinase and related kinases	K06596	CELLULAR PROCESSES AND SIGNALING
PA1458		probable two-component sensor	74821539	81713567	1.092113	COG2198	T	FOG: HPt domain	K07648	CELLULAR PROCESSES AND SIGNALING
PA1458		probable two-component sensor	74821539	81713567	1.092113	COG0643	N	Chemotaxis protein histidine kinase and related kinases	K06596	CELLULAR PROCESSES AND SIGNALING

PA1458		probable two-component sensor	74821539	81713567	1.092113	COG0643	N	Chemotaxis protein histidine kinase and related kinases	K02487	CELLULAR PROCESSES AND SIGNALING
PA1465		hypothetical protein	1.1E+08	0	0					
PA1479	ccmE	cytochrome C-type biogenesis protein CcmE	1E+09	5.32E+08	0.530313	COG2332	O	Cytochrome c-type biogenesis protein CcmE	K02197	CELLULAR PROCESSES AND SIGNALING
PA1482	ccmH	cytochrome C-type biogenesis protein CcmH	54848781	86542892	1.577845	COG3088	O	Uncharacterized protein involved in biosynthesis of c-type cyt	K04017	CELLULAR PROCESSES AND SIGNALING
PA1482	ccmH	cytochrome C-type biogenesis protein CcmH	54848781	86542892	1.577845	COG3088	O	Uncharacterized protein involved in biosynthesis of c-type cyt	K02200	CELLULAR PROCESSES AND SIGNALING
PA1483	cycH	cytochrome c-type biogenesis protein	58893060	48525560	0.823961	COG4235	O	Cytochrome c biogenesis factor	K04018	CELLULAR PROCESSES AND SIGNALING
PA1483	cycH	cytochrome c-type biogenesis protein	58893060	48525560	0.823961	COG4235	O	Cytochrome c biogenesis factor	K02200	CELLULAR PROCESSES AND SIGNALING
PA1493	cysP	sulfate-binding protein of ABC transporter	25921516	0	0	COG1613	P	"ABC-type sulfate transport system, periplasmic component"	K02048	METABOLISM
PA1494	muiA	mucoidy inhibitor gene A	0	12943532	1000					
PA1516		hypothetical protein	36440611	1.03E+08	2.838832	COG3195	S	Uncharacterized protein conserved in bacteria	K06016	POORLY CHARACTERIZED
PA1516		hypothetical protein	36440611	1.03E+08	2.838832	COG3195	S	Uncharacterized protein conserved in bacteria	K13485	POORLY CHARACTERIZED
PA1528	zipA	cell division protein ZipA	2.09E+09	2.21E+08	0.105689	COG3115	D	Cell division protein	K03528	CELLULAR PROCESSES AND SIGNALING
PA1532	dnaX	DNA polymerase subunits gamma and tau	77887795	55288152	0.709844	COG2812	L	"DNA polymerase III, gamma/tau subunits"	K02343	INFORMATION STORAGE AND PROCESSING
PA1532	dnaX	DNA polymerase subunits gamma and tau	77887795	55288152	0.709844	COG2812	L	"DNA polymerase III, gamma/tau subunits"	K02341	INFORMATION STORAGE AND PROCESSING
PA1533		conserved hypothetical protein	1.57E+08	4.84E+08	3.089335	COG0718	S	Uncharacterized protein conserved in bacteria	K09747	POORLY CHARACTERIZED
PA1550		hypothetical protein	4.11E+08	5.15E+08	1.252029	COG3198	S	Uncharacterized protein conserved in bacteria	K09926	POORLY CHARACTERIZED
PA1551		probable ferredoxin	2.01E+08	27155753	0.134804	COG0348	C	Polyferredoxin	K02574	METABOLISM
PA1552	ccoP1	"Cytochrome c oxidase	1.16E+08	3.92E+08	3.365611	COG2010	C	"Cytochrome c, mono- and diheme variants"	K02275	METABOLISM
PA1552	ccoP1	"Cytochrome c oxidase	1.16E+08	3.92E+08	3.365611	COG2010	C	"Cytochrome c, mono- and diheme variants"	K02275	METABOLISM
PA1552	ccoP1	"Cytochrome c oxidase	1.16E+08	3.92E+08	3.365611	COG2010	C	"Cytochrome c, mono- and diheme variants"	K00406	METABOLISM
PA1552	ccoP1	"Cytochrome c oxidase	1.16E+08	3.92E+08	3.365611	COG2010	C	"Cytochrome c, mono- and diheme variants"	K00406	METABOLISM
PA1553	ccoO1	"Cytochrome c oxidase	9.4E+08	7E+08	0.744461	COG2993	C	"Cbb3-type cytochrome oxidase, cytochrome c subunit"	K00405	METABOLISM
PA1555	ccoP2	"Cytochrome c oxidase	41132765	1.39E+08	3.379298	COG2010	C	"Cytochrome c, mono- and diheme variants"	K02275	METABOLISM
PA1555	ccoP2	"Cytochrome c oxidase	41132765	1.39E+08	3.379298	COG2010	C	"Cytochrome c, mono- and diheme variants"	K00406	METABOLISM
PA1555	ccoP2	"Cytochrome c oxidase	41132765	1.39E+08	3.379298	COG2010	C	"Cytochrome c, mono- and diheme variants"	K00406	METABOLISM
PA1555	ccoP2	"Cytochrome c oxidase	41132765	1.39E+08	3.379298	COG2010	C	"Cytochrome c, mono- and diheme variants"	K02275	METABOLISM
PA1556	ccoO2	"Cytochrome c oxidase	6.35E+08	4.44E+08	0.699727	COG2993	C	"Cbb3-type cytochrome oxidase, cytochrome c subunit"	K00405	METABOLISM
PA1561	aer	aerotaxis receptor Aer	2.32E+08	1.13E+09	4.861806	COG0840	T	Methyl-accepting chemotaxis protein	K03406	CELLULAR PROCESSES AND SIGNALING
PA1561	aer	aerotaxis receptor Aer	2.32E+08	1.13E+09	4.861806	COG0840	N	Methyl-accepting chemotaxis protein	K03406	CELLULAR PROCESSES AND SIGNALING
PA1562	acnA	aconitate hydratase 1	9887754	0	0	COG1048	C	Aconitase A	K01681	METABOLISM
PA1571		hypothetical protein	0	25465331	1000					
PA1579		hypothetical protein	32430204	0	0					
PA1580	gltA	citrate synthase	0	1.16E+08	1000	COG0372	C	Citrate synthase	K01659	METABOLISM
PA1580	gltA	citrate synthase	0	1.16E+08	1000	COG0372	C	Citrate synthase	K01647	METABOLISM
PA1583	sdhA	succinate dehydrogenase (A subunit)	4E+08	6.07E+08	1.518347	COG1053	C	"Succinate dehydrogenase/fumarate reductase, flavoprotein s	K00239	METABOLISM
PA1583	sdhA	succinate dehydrogenase (A subunit)	4E+08	6.07E+08	1.518347	COG1053	C	"Succinate dehydrogenase/fumarate reductase, flavoprotein s	K00244	METABOLISM
PA1584	sdhB	succinate dehydrogenase (B subunit)	0	87457358	1000	COG0479	C	"Succinate dehydrogenase/fumarate reductase, Fe-S protein s	K00245	METABOLISM
PA1584	sdhB	succinate dehydrogenase (B subunit)	0	87457358	1000	COG0479	C	"Succinate dehydrogenase/fumarate reductase, Fe-S protein s	K00240	METABOLISM
PA1585	sucA	2-oxoglutarate dehydrogenase (E1 subunit)	83629561	67210987	0.803675	COG0567	C	"2-oxoglutarate dehydrogenase complex, dehydrogenase (E1)	K01616	METABOLISM
PA1585	sucA	2-oxoglutarate dehydrogenase (E1 subunit)	83629561	67210987	0.803675	COG0567	C	"2-oxoglutarate dehydrogenase complex, dehydrogenase (E1)	K00164	METABOLISM
PA1586	sucB	dihydrolipoamide succinyltransferase (E2 subunit)	7.19E+08	1.52E+09	2.110144	COG0508	C	"Pyruvate/2-oxoglutarate dehydrogenase complex, dihydrolip	K00627	METABOLISM
PA1586	sucB	dihydrolipoamide succinyltransferase (E2 subunit)	7.19E+08	1.52E+09	2.110144	COG0508	C	"Pyruvate/2-oxoglutarate dehydrogenase complex, dihydrolip	K00658	METABOLISM
PA1586	sucB	dihydrolipoamide succinyltransferase (E2 subunit)	7.19E+08	1.52E+09	2.110144	COG0508	C	"Pyruvate/2-oxoglutarate dehydrogenase complex, dihydrolip	K09699	METABOLISM
PA1587	lpd	dihydrolipoamide dehydrogenase Lpd	2.63E+08	1.65E+09	6.281281	COG1249	C	"Pyruvate/2-oxoglutarate dehydrogenase complex, dihydrolip	K00520	METABOLISM
PA1587	lpd	dihydrolipoamide dehydrogenase Lpd	2.63E+08	1.65E+09	6.281281	COG1249	C	"Pyruvate/2-oxoglutarate dehydrogenase complex, dihydrolip	K00383	METABOLISM
PA1587	lpd	dihydrolipoamide dehydrogenase Lpd	2.63E+08	1.65E+09	6.281281	COG1249	C	"Pyruvate/2-oxoglutarate dehydrogenase complex, dihydrolip	K00322	METABOLISM
PA1587	lpd	dihydrolipoamide dehydrogenase Lpd	2.63E+08	1.65E+09	6.281281	COG1249	C	"Pyruvate/2-oxoglutarate dehydrogenase complex, dihydrolip	K00382	METABOLISM
PA1588	sucC	succinyl-CoA synthetase beta chain	8.36E+08	6.28E+09	7.517167	COG0045	C	"Succinyl-CoA synthetase, beta subunit"	K01903	METABOLISM
PA1588	sucC	succinyl-CoA synthetase beta chain	8.36E+08	6.28E+09	7.517167	COG0045	C	"Succinyl-CoA synthetase, beta subunit"	K01900	METABOLISM
PA1589	sucD	succinyl-CoA synthetase alpha chain	1.62E+09	4.18E+09	2.584875	COG0074	C	"Succinyl-CoA synthetase, alpha subunit"	K01902	METABOLISM
PA1596	htpG	heat shock protein HtpG	3.96E+08	5.93E+08	1.496488	COG0326	O	"Molecular chaperone, HSP90 family"	K04079	CELLULAR PROCESSES AND SIGNALING
PA1601		probable aldehyde dehydrogenase	13161359	1.57E+08	11.96061	COG1529	C	"Aerobic-type carbon monoxide dehydrogenase, large subuni	K11177	METABOLISM
PA1601		probable aldehyde dehydrogenase	13161359	1.57E+08	11.96061	COG1529	C	"Aerobic-type carbon monoxide dehydrogenase, large subuni	K07303	METABOLISM
PA1601		probable aldehyde dehydrogenase	13161359	1.57E+08	11.96061	COG1529	C	"Aerobic-type carbon monoxide dehydrogenase, large subuni	K03520	METABOLISM
PA1608		probable chemotaxis transducer	0	6.58E+08	1000	COG0840	N	Methyl-accepting chemotaxis protein	K03406	CELLULAR PROCESSES AND SIGNALING
PA1608		probable chemotaxis transducer	0	6.58E+08	1000	COG0840	T	Methyl-accepting chemotaxis protein	K03406	CELLULAR PROCESSES AND SIGNALING
PA1609	fabB	beta-ketoacyl-ACP synthase I	10966235	82808848	7.551256	COG0304	I	3-oxoacyl-(acyl-carrier-protein) synthase	K00647	METABOLISM
PA1609	fabB	beta-ketoacyl-ACP synthase I	10966235	82808848	7.551256	COG0304	I	3-oxoacyl-(acyl-carrier-protein) synthase	K09458	METABOLISM
PA1609	fabB	beta-ketoacyl-ACP synthase I	10966235	82808848	7.551256	COG0304	Q	3-oxoacyl-(acyl-carrier-protein) synthase	K09458	METABOLISM
PA1609	fabB	beta-ketoacyl-ACP synthase I	10966235	82808848	7.551256	COG0304	Q	3-oxoacyl-(acyl-carrier-protein) synthase	K00647	METABOLISM
PA1615		probable lipase	4.3E+08	3.34E+08	0.777904					
PA1641		hypothetical protein	22639600	0	0					
PA1646		probable chemotaxis transducer	36477344	7.2E+08	19.74484	COG0840	T	Methyl-accepting chemotaxis protein	K03406	CELLULAR PROCESSES AND SIGNALING
PA1646		probable chemotaxis transducer	36477344	7.2E+08	19.74484	COG0840	N	Methyl-accepting chemotaxis protein	K03406	CELLULAR PROCESSES AND SIGNALING
PA1657	hsiB2	HsiB2	1.21E+08	0	0	COG3516	S	Uncharacterized protein conserved in bacteria	K11901	POORLY CHARACTERIZED
PA1707	pcrH	regulatory protein PcrH	0	1.73E+08	1000					
PA1708	popB	translocator protein PopB	8807933	0	0					
PA1709	popD	Translocator outer membrane protein PopD precursor	0	62768134	1000					
PA1746		hypothetical protein	1.13E+09	1.08E+09	0.949674					
PA1749		hypothetical protein	6.16E+08	4.26E+08	0.69172	COG2388	R	Predicted acetyltransferase	K06975	POORLY CHARACTERIZED
PA1750		phospho-2-dehydro-3-deoxyheptonate aldolase	72586107	0	0	COG0722	E	3-deoxy-D-arabino-heptulosonate 7-phosphate (DAHP) syntha	K01626	METABOLISM
PA1754	cysB	transcriptional regulator CysB	28770771	0	0					
PA1770	ppsA	phosphoenolpyruvate synthase	1.87E+08	1.15E+09	6.147403	COG0574	G	Phosphoenolpyruvate synthase/pyruvate phosphate dikinase	K01007	METABOLISM
PA1770	ppsA	phosphoenolpyruvate synthase	1.87E+08	1.15E+09	6.147403	COG1080	G	Phosphoenolpyruvate-protein kinase (PTS system EI compone	K08483	METABOLISM
PA1770	ppsA	phosphoenolpyruvate synthase	1.87E+08	1.15E+09	6.147403	COG0574	G	Phosphoenolpyruvate synthase/pyruvate phosphate dikinase	K01006	METABOLISM
PA1770	ppsA	phosphoenolpyruvate synthase	1.87E+08	1.15E+09	6.147403	COG1080	G	Phosphoenolpyruvate-protein kinase (PTS system EI compone	K02768	METABOLISM
PA1770	ppsA	phosphoenolpyruvate synthase	1.87E+08	1.15E+09	6.147403	COG1080	G	Phosphoenolpyruvate-protein kinase (PTS system EI compone	K11201	METABOLISM
PA1770	ppsA	phosphoenolpyruvate synthase	1.87E+08	1.15E+09	6.147403	COG1080	G	Phosphoenolpyruvate-protein kinase (PTS system EI compone	K11183	METABOLISM
PA1770	ppsA	phosphoenolpyruvate synthase	1.87E+08	1.15E+09	6.147403	COG1080	G	Phosphoenolpyruvate-protein kinase (PTS system EI compone	K11189	METABOLISM
PA1772		probable methyltransferase	3.16E+08	6.62E+08	2.093863	COG0684	H	Demethylmenaquinone methyltransferase	K02553	METABOLISM
PA1777	oprF	Major porin and structural outer membrane porin OprF precursor	7.39E+10	1.04E+11	1.409752	COG2885	M	Outer membrane protein and related peptidoglycan-associate	K03286	CELLULAR PROCESSES AND SIGNALING
PA1777	oprF	Major porin and structural outer membrane porin OprF precursor	7.39E+10	1.04E+11	1.409752	COG3047	M	Outer membrane protein W	K07275	CELLULAR PROCESSES AND SIGNALING
PA1777	oprF	Major porin and structural outer membrane porin OprF precursor	7.39E+10	1.04E+11	1.409752	COG2885	M	Outer membrane protein and related peptidoglycan-associate	K03640	CELLULAR PROCESSES AND SIGNALING
PA1787	acnB	aconitate hydratase 2	1.46E+09	1.8E+09	1.232215	COG1049	C	Aconitase B	K01682	METABOLISM
PA1793	ppiB	peptidyl-prolyl cis-trans isomerase B	1.25E+10	1.33E+10	1.067032	COG0652	O	Peptidyl-prolyl cis-trans isomerase (rotamase) - cyclophilin far	K03767	CELLULAR PROCESSES AND SIGNALING
PA1793	ppiB	peptidyl-prolyl cis-trans isomerase B	1.25E+10	1.33E+10	1.067032	COG0652	O	Peptidyl-prolyl cis-trans isomerase (rotamase) - cyclophilin far	K01802	CELLULAR PROCESSES AND SIGNALING
PA1793	ppiB	peptidyl-prolyl cis-trans isomerase B	1.25E+10	1.33E+10	1.067032	COG0652	O	Peptidyl-prolyl cis-trans isomerase (rotamase) - cyclophilin far	K03768	CELLULAR PROCESSES AND SIGNALING
PA1800	tig	trigger factor	1.07E+10	8.65E+09	0.810073	COG0544	O	FKBP-type peptidyl-prolyl cis-trans isomerase (trigger factor)	K03545	CELLULAR PROCESSES AND SIGNALING
PA1801	clpP	ClpP	2.75E+08	1.01E+09	3.670035	COG0740	U	Protease subunit of ATP-dependent Clp proteases	K01358	CELLULAR PROCESSES AND SIGNALING
PA1801	clpP	ClpP	2.75E+08	1.01E+09	3.670035	COG0740	O	Protease subunit of ATP-dependent Clp proteases	K01358	CELLULAR PROCESSES AND SIGNALING
PA1802	clpX	ClpX	1.61E+10	1.1E+09	6.82E-02	COG1219	O	"ATP-dependent protease Clp, ATPase subunit"	K03544	CELLULAR PROCESSES AND SIGNALING
PA1803	lon	Lon protease	48701880	1.01E+08	2.082254	COG0466	O	"ATP-dependent Lon protease, bacterial type"	K01338	CELLULAR PROCESSES AND SIGNALING
PA1804	hupB	DNA-binding protein HU	2.71E+09	1.02E+10	3.764223	COG0776	L	Bacterial nucleoid DNA-binding protein	K03530	INFORMATION STORAGE AND PROCESSING
PA1804	hupB	DNA-binding protein HU	2.71E+09	1.02E+10	3.764223	COG0776	L	Bacterial nucleoid DNA-binding protein	K05787	INFORMATION STORAGE AND PROCESSING
PA1804	hupB	DNA-binding protein HU	2.71E+09	1.02E+10	3.764223	COG0776	L	Bacterial nucleoid DNA-binding protein	K04764	INFORMATION STORAGE AND PROCESSING
PA1804	hupB	DNA-binding protein HU	2.71E+09	1.02E+10	3.764223	COG0776	L	Bacterial nucleoid DNA-binding protein	K05788	INFORMATION STORAGE AND PROCESSING
PA1805	ppiD	peptidyl-prolyl cis-trans isomerase D	5.52E+09	1.93E+09	0.35066	COG0760	O	Parvulin-like peptidyl-prolyl isomerase	K03771	CELLULAR PROCESSES AND SIGNALING
PA1805	ppiD	peptidyl-prolyl cis-trans isomerase D	5.52E+09	1.93E+09	0.35066	COG0760	O	Parvulin-like peptidyl-prolyl isomerase	K03770	CELLULAR PROCESSES AND SIGNALING
PA1805	ppiD	peptidyl-prolyl cis-trans isomerase D	5.52E+09	1.93E+09	0.35066	COG0760	O	Parvulin-like peptidyl-prolyl isomerase	K07533	CELLULAR PROCESSES AND SIGNALING
PA1805	ppiD	peptidyl-prolyl cis-trans isomerase D	5.52E+09	1.93E+09	0.35066	COG0760	O	Parvulin-like peptidyl-prolyl isomerase	K03769	CELLULAR PROCESSES AND SIGNALING
PA1815	rnhA	ribonuclease H	1.48E+08	0	0	COG0328	L	Ribonuclease HI	K03469	INFORMATION STORAGE AND PROCESSING
PA1830		hypothetical protein	2.05E+08	90065558	0.439464					
PA1832		probable protease	21925677	88653812	4.043379	COG0616	O	Periplasmic serine proteases (ClpP class)	K04773	CELLULAR PROCESSES AND SIGNALING
PA1832		probable protease	21925677	88653812	4.043379	COG0616	O	Periplasmic serine proteases (ClpP class)	K04774	CELLULAR PROCESSES AND SIGNALING
PA1832		probable protease	21925677	88653812	4.043379	COG0616	U	Periplasmic serine proteases (ClpP class)	K04774	CELLULAR PROCESSES AND SIGNALING
PA1832		probable protease	21925677	88653812	4.043379	COG0616	U	Periplasmic serine proteases (ClpP class)	K04773	CELLULAR PROCESSES AND SIGNALING
PA1847	nfuA	NfuA	9.32E+08	6.68E+08	0.716538	COG0694	O	Thioredoxin-like proteins and domains	K13819	CELLULAR PROCESSES AND SIGNALING
PA1847	nfuA	NfuA	9.32E+08	6.68E+08	0.716538	COG0316	S	Uncharacterized conserved protein	K13628	POORLY CHARACTERIZED
PA1847	nfuA	NfuA	9.32E+08	6.68E+08	0.716538	COG0316	S	Uncharacterized conserved protein	K07400	POORLY CHARACTERIZED
PA1847	nfuA	NfuA	9.32E+08	6.68E+08	0.716538	COG0694	O	Thioredoxin-like proteins and domains	K07400	CELLULAR PROCESSES AND SIGNALING
PA1852		hypothetical protein	1.12E+08	55346115	0.495588					
PA1868	xqhA	secretion protein XqhA	0	23738626	1000	COG1450	N	"Type II secretory pathway, component PulD"	K12282	CELLULAR PROCESSES AND SIGNALING
PA1868	xqhA	secretion protein XqhA	0	23738626	1000	COG1450	N	"Type II secretory pathway, component PulD"	K03219	CELLULAR PROCESSES AND SIGNALING
PA1868	xqhA	secretion protein XqhA	0	23738626	1000	COG1450	N	"Type II secretory pathway, component PulD"	K02453	CELLULAR PROCESSES AND SIGNALING
PA1868	xqhA	secretion protein XqhA	0	23738626	1000	COG1450	U	"Type II secretory pathway, component PulD"	K12282	CELLULAR PROCESSES AND SIGNALING
PA1868	xqhA	secretion protein XqhA	0	23738626	1000	COG1450	U	"Type II secretory pathway, component PulD"	K02453	CELLULAR PROCESSES AND SIGNALING
PA1868	xqhA	secretion protein XqhA	0	23738626	1000	COG3266	S	Uncharacterized protein conserved in bacteria	K03112	POORLY CHARACTERIZED
PA1868	xqhA	secretion protein XqhA	0	23738626	1000	COG1450	U	"Type II secretory pathway, component PulD"	K03219	CELLULAR PROCESSES AND SIGNALING
PA1870		hypothetical protein	73983565	12674341	0.171313					
PA1900	phzB2	probable phenazine biosynthesis protein	0	15591689	1000	COG3631	R	Ketosteroid isomerase-related protein	K06893	POORLY CHARACTERIZED
PA1907		hypothetical protein	0	40629296	1000					

PA1926		Uncharacterized protein	0	59766234	1000	COG1944	S	Uncharacterized conserved protein	K09136	POORLY CHARACTERIZED
PA1930		probable chemotaxis transducer	0	6.58E+08	1000	COG0840	N	Methyl-accepting chemotaxis protein	K03406	CELLULAR PROCESSES AND SIGNALING
PA1930		probable chemotaxis transducer	0	6.58E+08	1000	COG2202	T	FOG: PAS/PAC domain	K00936	CELLULAR PROCESSES AND SIGNALING
PA1930		probable chemotaxis transducer	0	6.58E+08	1000	COG0840	T	Methyl-accepting chemotaxis protein	K03406	CELLULAR PROCESSES AND SIGNALING
PA1969		hypothetical protein	4.2E+09	3.77E+09	0.896596					
PA2021		hypothetical protein	4.27E+08	9.34E+08	2.189186					
PA2071	fusA2	elongation factor G	88381954	1.21E+08	1.367377	COG0480	J	Translation elongation factors (GTPases)	K02355	INFORMATION STORAGE AND PROCESSING
PA2071	fusA2	elongation factor G	88381954	1.21E+08	1.367377	COG0480	J	Translation elongation factors (GTPases)	K03234	INFORMATION STORAGE AND PROCESSING
PA2081	kynB	"kynurenine formamidase	66069375	1.31E+08	1.982797	COG1878	R	Predicted metal-dependent hydrolase	K07130	POORLY CHARACTERIZED
PA2126	cgrC	"cupA gene regulator C	0	36930331	1000	COG1475	K	Predicted transcriptional regulators	K03497	INFORMATION STORAGE AND PROCESSING
PA2169		hypothetical protein	4.51E+08	3.46E+08	0.76812					
PA2190		conserved hypothetical protein	21807882	0	0	COG3729	R	General stress protein	K06884	POORLY CHARACTERIZED
PA2193	hcnA	hydrogen cyanide synthase HcnA	12248757	0	0	COG3383	R	Uncharacterized anaerobic dehydrogenase	K00336	POORLY CHARACTERIZED
PA2193	hcnA	hydrogen cyanide synthase HcnA	12248757	0	0	COG3383	R	Uncharacterized anaerobic dehydrogenase	K00123	POORLY CHARACTERIZED
PA2247	bkdA1	2-oxoisovalerate dehydrogenase (alpha subunit)	20778110	0	0	COG1071	C	"Pyruvate/2-oxoglutarate dehydrogenase complex, dehydroge	K00161	METABOLISM
PA2247	bkdA1	2-oxoisovalerate dehydrogenase (alpha subunit)	20778110	0	0	COG1071	C	"Pyruvate/2-oxoglutarate dehydrogenase complex, dehydroge	K11381	METABOLISM
PA2247	bkdA1	2-oxoisovalerate dehydrogenase (alpha subunit)	20778110	0	0	COG1071	C	"Pyruvate/2-oxoglutarate dehydrogenase complex, dehydroge	K00166	METABOLISM
PA2249	bkdB	branched-chain alpha-keto acid dehydrogenase (lipoamide compo	0	1.08E+08	1000	COG0508	C	"Pyruvate/2-oxoglutarate dehydrogenase complex, dihydrolip	K09699	METABOLISM
PA2249	bkdB	branched-chain alpha-keto acid dehydrogenase (lipoamide compo	0	1.08E+08	1000	COG0508	C	"Pyruvate/2-oxoglutarate dehydrogenase complex, dihydrolip	K00658	METABOLISM
PA2249	bkdB	branched-chain alpha-keto acid dehydrogenase (lipoamide compo	0	1.08E+08	1000	COG0508	C	"Pyruvate/2-oxoglutarate dehydrogenase complex, dihydrolip	K00627	METABOLISM
PA2264		conserved hypothetical protein	59696268	77740593	1.302269					
PA2291		probable glucose-sensitive porin	0	1.07E+08	1000	COG3659	M	Carbohydrate-selective porin	K07267	CELLULAR PROCESSES AND SIGNALING
PA2331		hypothetical protein	2.97E+09	1.78E+09	0.598278	COG2128	S	Uncharacterized conserved protein	K04756	POORLY CHARACTERIZED
PA2349		conserved hypothetical protein	20675151	0	0	COG1464	P	"ABC-type metal ion transport system, periplasmic componen	K02073	METABOLISM
PA2381		hypothetical protein	25514147	0	0					
PA2385	pvdQ	3-oxo-C12-homoserine lactone acylase PvdQ	13898608	3.38E+08	24.32869	COG2366	R	Protein related to penicillin acylase	K01434	POORLY CHARACTERIZED
PA2385	pvdQ	3-oxo-C12-homoserine lactone acylase PvdQ	13898608	3.38E+08	24.32869	COG2366	R	Protein related to penicillin acylase	K07116	POORLY CHARACTERIZED
PA2392	pvdP	PvdP	94354307	0	0					
PA2393		putative dipeptidase	59609396	4.66E+08	7.822192	COG2355	E	"Zn-dependent dipeptidase, microsomal dipeptidase homolog	K01273	METABOLISM
PA2395	pvdO	PvdO	3.09E+08	6.87E+08	2.221798					
PA2396	pvdF	pyoverdine synthetase F	3.51E+08	3.69E+08	1.051129	COG0299	F	Folate-dependent phosphoribosylglycinamide formyltransfera	K11175	METABOLISM
PA2398	fpvA	ferripyoverdine receptor	0	14353276	1000	COG4773	P	Outer membrane receptor for ferric coprogen and ferric-rhod	K02014	METABOLISM
PA2410	fpvF	FpvF	2.77E+08	91002457	0.328639	COG0803	P	"ABC-type metal ion transport system, periplasmic componen	K09818	METABOLISM
PA2410	fpvF	FpvF	2.77E+08	91002457	0.328639	COG0803	P	"ABC-type metal ion transport system, periplasmic componen	K02077	METABOLISM
PA2410	fpvF	FpvF	2.77E+08	91002457	0.328639	COG0803	P	"ABC-type metal ion transport system, periplasmic componen	K09815	METABOLISM
PA2412		conserved hypothetical protein	0	7.33E+08	1000	COG3251	S	Uncharacterized protein conserved in bacteria	K05375	POORLY CHARACTERIZED
PA2424	pvdL	PvdL	50919973	0	0	COG3433	Q	Aryl carrier domain	K04784	METABOLISM
PA2424	pvdL	PvdL	50919973	0	0	COG0318	I	Acyl-CoA synthetases (AMP-forming)/AMP-acid ligases II	K00666	METABOLISM
PA2424	pvdL	PvdL	50919973	0	0	COG3433	Q	Aryl carrier domain	K12239	METABOLISM
PA2424	pvdL	PvdL	50919973	0	0	COG3433	Q	Aryl carrier domain	K01252	METABOLISM
PA2424	pvdL	PvdL	50919973	0	0	COG0318	I	Acyl-CoA synthetases (AMP-forming)/AMP-acid ligases II	K01911	METABOLISM
PA2424	pvdL	PvdL	50919973	0	0	COG0318	Q	Acyl-CoA synthetases (AMP-forming)/AMP-acid ligases II	K01897	METABOLISM
PA2424	pvdL	PvdL	50919973	0	0	COG0318	Q	Acyl-CoA synthetases (AMP-forming)/AMP-acid ligases II	K01911	METABOLISM
PA2424	pvdL	PvdL	50919973	0	0	COG3433	Q	Aryl carrier domain	K04788	METABOLISM
PA2424	pvdL	PvdL	50919973	0	0	COG0318	Q	Acyl-CoA synthetases (AMP-forming)/AMP-acid ligases II	K00666	METABOLISM
PA2424	pvdL	PvdL	50919973	0	0	COG0318	I	Acyl-CoA synthetases (AMP-forming)/AMP-acid ligases II	K01897	METABOLISM
PA2424	pvdL	PvdL	50919973	0	0	COG3433	Q	Aryl carrier domain	K04779	METABOLISM
PA2433		hypothetical protein	1.31E+08	1.5E+08	1.146884					
PA2446	gcvH2	glycine cleavage system protein H2	52540330	1.13E+08	2.144673	COG0509	E	Glycine cleavage system H protein (lipoate-binding)	K02437	METABOLISM
PA2453		hypothetical protein	2.73E+08	0	0					
PA2476	dsbG	thiol:disulfide interchange protein DsbG	36514881	29810023	0.81638	COG1651	O	Protein-disulfide isomerase	K03981	CELLULAR PROCESSES AND SIGNALING
PA2476	dsbG	thiol:disulfide interchange protein DsbG	36514881	29810023	0.81638	COG1651	O	Protein-disulfide isomerase	K03805	CELLULAR PROCESSES AND SIGNALING
PA2507	catA	"catechol 1	3.12E+09	7.2E+08	0.230923	COG3485	Q	"Protocatechuate 3,4-dioxygenase beta subunit"	K03381	METABOLISM
PA2507	catA	"catechol 1	3.12E+09	7.2E+08	0.230923	COG3485	Q	"Protocatechuate 3,4-dioxygenase beta subunit"	K00449	METABOLISM
PA2507	catA	"catechol 1	3.12E+09	7.2E+08	0.230923	COG3485	Q	"Protocatechuate 3,4-dioxygenase beta subunit"	K00448	METABOLISM
PA2508	catC	muconolactone delta-isomerase	56314074	1.58E+08	2.802931	COG4829	Q	Muconolactone delta-isomerase	K01828	METABOLISM
PA2508	catC	muconolactone delta-isomerase	56314074	1.58E+08	2.802931	COG4829	Q	Muconolactone delta-isomerase	K03464	METABOLISM
PA2525	opmB	OpmB	63110458	0	0	COG1538	M	Outer membrane protein	K03287	CELLULAR PROCESSES AND SIGNALING
PA2525	opmB	OpmB	63110458	0	0	COG1538	U	Outer membrane protein	K12340	CELLULAR PROCESSES AND SIGNALING
PA2525	opmB	OpmB	63110458	0	0	COG1538	U	Outer membrane protein	K03287	CELLULAR PROCESSES AND SIGNALING
PA2525	opmB	OpmB	63110458	0	0	COG1538	M	Outer membrane protein	K12340	CELLULAR PROCESSES AND SIGNALING
PA2532	tpx	thiol peroxidase	1.44E+08	3.37E+08	2.341829	COG2077	O	Peroxiredoxin	K11065	CELLULAR PROCESSES AND SIGNALING
PA2561	ctpH	CtpH	0	6.89E+08	1000	COG0840	T	Methyl-accepting chemotaxis protein	K03406	CELLULAR PROCESSES AND SIGNALING
PA2561	ctpH	CtpH	0	6.89E+08	1000	COG0840	N	Methyl-accepting chemotaxis protein	K03406	CELLULAR PROCESSES AND SIGNALING
PA2573		probable chemotaxis transducer	1.03E+08	1.04E+09	10.06826	COG0840	T	Methyl-accepting chemotaxis protein	K03406	CELLULAR PROCESSES AND SIGNALING
PA2573		probable chemotaxis transducer	1.03E+08	1.04E+09	10.06826	COG0840	N	Methyl-accepting chemotaxis protein	K03406	CELLULAR PROCESSES AND SIGNALING
PA2575		hypothetical protein	1.27E+08	1.56E+08	1.221266	COG3560	R	Predicted oxidoreductase related to nitroreductase	K07078	POORLY CHARACTERIZED
PA2582		hypothetical protein	7.84E+08	5.6E+08	0.714719	COG3109	T	Activator of osmoprotectant transporter ProP	K03607	CELLULAR PROCESSES AND SIGNALING
PA2606		conserved hypothetical protein	0	8433441	1000	COG2923	P	Uncharacterized protein involved in the oxidation of intracellu	K07236	METABOLISM
PA2612	serS	seryl-tRNA synthetase	26266054	0	0	COG0172	J	Seryl-tRNA synthetase	K01875	INFORMATION STORAGE AND PROCESSING
PA2619	infA	initiation factor	1.03E+08	59632693	0.581773	COG0361	J	Translation initiation factor 1 (IF-1)	K03236	INFORMATION STORAGE AND PROCESSING
PA2619	infA	initiation factor	1.03E+08	59632693	0.581773	COG0361	J	Translation initiation factor 1 (IF-1)	K02518	INFORMATION STORAGE AND PROCESSING
PA2620	clpA	ATP-binding protease component ClpA	2.18E+08	1.14E+08	0.520334	COG0542	O	"ATPases with chaperone activity, ATP-binding subunit"	K11907	CELLULAR PROCESSES AND SIGNALING
PA2620	clpA	ATP-binding protease component ClpA	2.18E+08	1.14E+08	0.520334	COG0542	O	"ATPases with chaperone activity, ATP-binding subunit"	K03696	CELLULAR PROCESSES AND SIGNALING
PA2620	clpA	ATP-binding protease component ClpA	2.18E+08	1.14E+08	0.520334	COG0542	O	"ATPases with chaperone activity, ATP-binding subunit"	K03695	CELLULAR PROCESSES AND SIGNALING
PA2620	clpA	ATP-binding protease component ClpA	2.18E+08	1.14E+08	0.520334	COG0542	O	"ATPases with chaperone activity, ATP-binding subunit"	K03694	CELLULAR PROCESSES AND SIGNALING
PA2622	cspD	cold-shock protein CspD	2.3E+08	1.24E+08	0.538447	COG1278	K	Cold shock proteins	K03704	INFORMATION STORAGE AND PROCESSING
PA2623	icd	isocitrate dehydrogenase	2.11E+08	1.27E+09	6.008676	COG0538	C	Isocitrate dehydrogenases	K00030	METABOLISM
PA2623	icd	isocitrate dehydrogenase	2.11E+08	1.27E+09	6.008676	COG0538	C	Isocitrate dehydrogenases	K00031	METABOLISM
PA2624	idh	isocitrate dehydrogenase	3.01E+08	2.08E+09	6.935855	COG2838	C	Monomeric isocitrate dehydrogenase	K00031	METABOLISM
PA2626	trmU	tRNA methyltransferase	0	62963101	1000	COG0482	J	"Predicted tRNA(5-methylaminomethyl-2-thiouridylate) meth	K00566	INFORMATION STORAGE AND PROCESSING
PA2638	nuoB	NADH dehydrogenase I chain B	0	69624370	1000	COG0377	C	NADH:ubiquinone oxidoreductase 20 kD subunit and related F	K05582	METABOLISM
PA2638	nuoB	NADH dehydrogenase I chain B	0	69624370	1000	COG0377	C	NADH:ubiquinone oxidoreductase 20 kD subunit and related F	K00331	METABOLISM
PA2640	nuoE	NADH dehydrogenase I chain E	4.61E+08	3.74E+08	0.81103	COG1905	C	NADH:ubiquinone oxidoreductase 24 kD subunit	K00334	METABOLISM
PA2640	nuoE	NADH dehydrogenase I chain E	4.61E+08	3.74E+08	0.81103	COG1905	C	NADH:ubiquinone oxidoreductase 24 kD subunit	K00127	METABOLISM
PA2641	nuoF	NADH dehydrogenase I chain F	32569986	68367995	2.09911	COG1894	C	"NADH:ubiquinone oxidoreductase, NADH-binding (51 kD) sub	K00335	METABOLISM
PA2641	nuoF	NADH dehydrogenase I chain F	32569986	68367995	2.09911	COG1894	C	"NADH:ubiquinone oxidoreductase, NADH-binding (51 kD) sub	K00124	METABOLISM
PA2641	nuoF	NADH dehydrogenase I chain F	32569986	68367995	2.09911	COG1894	C	"NADH:ubiquinone oxidoreductase, NADH-binding (51 kD) sub	K05903	METABOLISM
PA2642	nuoG	NADH dehydrogenase I chain G	64566992	1.49E+08	2.300599	COG1034	C	NADH dehydrogenase/NADH:ubiquinone oxidoreductase 75 k	K00336	METABOLISM
PA2644	nuoI	NADH Dehydrogenase I chain I	1.8E+08	2.85E+08	1.5838	COG1143	C	Formate hydrogenlyase subunit 6/NADH:ubiquinone oxidored	K05580	METABOLISM
PA2644	nuoI	NADH Dehydrogenase I chain I	1.8E+08	2.85E+08	1.5838	COG1143	C	Formate hydrogenlyase subunit 6/NADH:ubiquinone oxidored	K00338	METABOLISM
PA2652		methyl-accepting chemotaxis protein	2.3E+08	1.56E+09	6.781302	COG4564	T	Signal transduction histidine kinase	K02480	CELLULAR PROCESSES AND SIGNALING
PA2652		methyl-accepting chemotaxis protein	2.3E+08	1.56E+09	6.781302	COG0840	N	Methyl-accepting chemotaxis protein	K03406	CELLULAR PROCESSES AND SIGNALING
PA2652		methyl-accepting chemotaxis protein	2.3E+08	1.56E+09	6.781302	COG0840	T	Methyl-accepting chemotaxis protein	K03406	CELLULAR PROCESSES AND SIGNALING
PA2654	tlpQ	TlpQ	4.98E+08	7.14E+08	1.432656	COG0840	T	Methyl-accepting chemotaxis protein	K03406	CELLULAR PROCESSES AND SIGNALING
PA2654	tlpQ	TlpQ	4.98E+08	7.14E+08	1.432656	COG0840	N	Methyl-accepting chemotaxis protein	K03406	CELLULAR PROCESSES AND SIGNALING
PA2659		hypothetical protein	2.14E+09	2.2E+09	1.025548					
PA2667	mvaU	MvaU	2.65E+09	1.98E+09	0.748117					
PA2709	cysK	cysteine synthase A	0	97127150	1000	COG0031	E	Cysteine synthase	K12339	METABOLISM
PA2709	cysK	cysteine synthase A	0	97127150	1000	COG0031	E	Cysteine synthase	K01697	METABOLISM
PA2709	cysK	cysteine synthase A	0	97127150	1000	COG0031	E	Cysteine synthase	K01738	METABOLISM
PA2735		type I HsdM methyltransferase	50453081	0	0	COG0286	V	Type I restriction-modification system methyltransferase subu	K03427	CELLULAR PROCESSES AND SIGNALING
PA2738	himA	"integration host factor	1.04E+09	1.56E+09	1.495737	COG0776	L	Bacterial nucleoid DNA-binding protein	K04764	INFORMATION STORAGE AND PROCESSING
PA2738	himA	"integration host factor	1.04E+09	1.56E+09	1.495737	COG0776	L	Bacterial nucleoid DNA-binding protein	K05787	INFORMATION STORAGE AND PROCESSING
PA2738	himA	"integration host factor	1.04E+09	1.56E+09	1.495737	COG0776	L	Bacterial nucleoid DNA-binding protein	K03530	INFORMATION STORAGE AND PROCESSING
PA2738	himA	"integration host factor	1.04E+09	1.56E+09	1.495737	COG0776	L	Bacterial nucleoid DNA-binding protein	K05788	INFORMATION STORAGE AND PROCESSING
PA2739	pheT	"phenylalanyl-tRNA synthetase	0	7233645	1000	COG0073	R	EMAP domain	K01874	POORLY CHARACTERIZED
PA2739	pheT	"phenylalanyl-tRNA synthetase	0	7233645	1000	COG0073	R	EMAP domain	K06878	POORLY CHARACTERIZED
PA2739	pheT	"phenylalanyl-tRNA synthetase	0	7233645	1000	COG0072	J	Phenylalanyl-tRNA synthetase beta subunit	K01890	INFORMATION STORAGE AND PROCESSING
PA2739	pheT	"phenylalanyl-tRNA synthetase	0	7233645	1000	COG0073	R	EMAP domain	K01890	POORLY CHARACTERIZED
PA2740	pheS	"phenylalanyl-tRNA synthetase	38812159	1.63E+08	4.196589	COG0016	J	Phenylalanyl-tRNA synthetase alpha subunit	K01889	INFORMATION STORAGE AND PROCESSING
PA2742	rpmI	50S ribosomal protein L35	0	1.16E+08	1000	COG0291	J	Ribosomal protein L35	K02916	INFORMATION STORAGE AND PROCESSING
PA2743	infC	translation initiation factor IF-3	5.53E+08	8.25E+08	1.491994	COG0290	J	Translation initiation factor 3 (IF-3)	K02520	INFORMATION STORAGE AND PROCESSING
PA2744	thrS	threonyl-tRNA synthetase	0	66350419	1000	COG0441	J	Threonyl-tRNA synthetase	K01868	INFORMATION STORAGE AND PROCESSING
PA2755	eco	ecotin precursor	4.09E+08	1.67E+08	0.407356	COG4574	R	Serine protease inhibitor ecotin	K08276	POORLY CHARACTERIZED
PA2779		hypothetical protein	0	2.26E+08	1000					
PA2788		probable chemotaxis transducer	5.05E+09	9.09E+09	1.800074	COG3850	T	"Signal transduction histidine kinase, nitrate/nitrite-specific"	K07674	CELLULAR PROCESSES AND SIGNALING
PA2788		probable chemotaxis transducer	5.05E+09	9.09E+09	1.800074	COG0840	N	Methyl-accepting chemotaxis protein	K03406	CELLULAR PROCESSES AND SIGNALING
PA2788		probable chemotaxis transducer	5.05E+09	9.09E+09	1.800074	COG0840	T	Methyl-accepting chemotaxis protein	K03406	CELLULAR PROCESSES AND SIGNALING
PA2788		probable chemotaxis transducer	5.05E+09	9.09E+09	1.800074	COG3850	T	"Signal transduction histidine kinase, nitrate/nitrite-specific"	K07673	CELLULAR PROCESSES AND SIGNALING
PA2796	tal	transaldolase	35208496	2.58E+08	7.325053	COG0176	G	Transaldolase	K00616	METABOLISM

PA2800	vacJ	VacJ	1.59E+09	9.02E+08	0.568801	COG2853	M	Surface lipoprotein	K04754	CELLULAR PROCESSES AND SIGNALING
PA2805		hypothetical protein	8.14E+08	6.23E+08	0.765309					
PA2817		hypothetical protein	6.06E+08	2.69E+08	0.444123					
PA2823		conserved hypothetical protein	1.8E+08	1.09E+08	0.607366	COG2607	R	Predicted ATPase (AAA superfamily)	K06923	POORLY CHARACTERIZED
PA2827		conserved hypothetical protein	17254526	0	0	COG0229	O	Conserved domain frequently associated with peptide methio	K12267	CELLULAR PROCESSES AND SIGNALING
PA2827		conserved hypothetical protein	17254526	0	0	COG0229	O	Conserved domain frequently associated with peptide methio	K07305	CELLULAR PROCESSES AND SIGNALING
PA2851	efp	translation elongation factor P	8.58E+08	1.13E+09	1.314927	COG0231	J	Translation elongation factor P (EF-P)/translation initiation fac	K02356	INFORMATION STORAGE AND PROCESSING
PA2851	efp	translation elongation factor P	8.58E+08	1.13E+09	1.314927	COG0231	J	Translation elongation factor P (EF-P)/translation initiation fac	K03263	INFORMATION STORAGE AND PROCESSING
PA2853	oprI	Outer membrane lipoprotein OprI precursor	2.86E+09	3.64E+09	1.270996					
PA2867		probable chemotaxis transducer	59727191	7.86E+08	13.16402	COG0840	T	Methyl-accepting chemotaxis protein	K03406	CELLULAR PROCESSES AND SIGNALING
PA2867		probable chemotaxis transducer	59727191	7.86E+08	13.16402	COG0840	N	Methyl-accepting chemotaxis protein	K03406	CELLULAR PROCESSES AND SIGNALING
PA2883		hypothetical protein	2.9E+08	1.41E+08	0.487374					
PA2894		hypothetical protein	35239777	0	0					
PA2900		probable outer membrane protein precursor	0	11238943	1000	COG2885	M	Outer membrane protein and related peptidoglycan-associate	K03640	CELLULAR PROCESSES AND SIGNALING
PA2900		probable outer membrane protein precursor	0	11238943	1000	COG2885	M	Outer membrane protein and related peptidoglycan-associate	K03286	CELLULAR PROCESSES AND SIGNALING
PA2901		hypothetical protein	1.64E+08	2.24E+08	1.363111					
PA2920		probable chemotaxis transducer	88586532	6.58E+08	7.425097	COG0840	T	Methyl-accepting chemotaxis protein	K03406	CELLULAR PROCESSES AND SIGNALING
PA2920		probable chemotaxis transducer	88586532	6.58E+08	7.425097	COG0840	N	Methyl-accepting chemotaxis protein	K03406	CELLULAR PROCESSES AND SIGNALING
PA2945		conserved hypothetical protein	1.97E+08	2.58E+08	1.307847	COG0523	R	Putative GTPases (G3E family)	K02234	POORLY CHARACTERIZED
PA2950	fabV	FabV	32442538	2.15E+08	6.632426	COG3007	S	Uncharacterized paraquat-inducible protein B	K10783	POORLY CHARACTERIZED
PA2951	etfA	electron transfer flavoprotein alpha-subunit	3.99E+08	9.34E+08	2.340312	COG2025	C	"Electron transfer flavoprotein, alpha subunit"	K03522	METABOLISM
PA2952	etfB	electron transfer flavoprotein beta-subunit	3.1E+08	2.02E+09	6.506742	COG2086	C	"Electron transfer flavoprotein, beta subunit"	K03521	METABOLISM
PA2953		electron transfer flavoprotein-ubiquinone oxidoreductase	0	1.16E+08	1000	COG0644	C	Dehydrogenases (flavoproteins)	K00311	METABOLISM
PA2953		electron transfer flavoprotein-ubiquinone oxidoreductase	0	1.16E+08	1000	COG0644	C	Dehydrogenases (flavoproteins)	K10960	METABOLISM
PA2953		electron transfer flavoprotein-ubiquinone oxidoreductase	0	1.16E+08	1000	COG0644	C	Dehydrogenases (flavoproteins)	K00313	METABOLISM
PA2953		electron transfer flavoprotein-ubiquinone oxidoreductase	0	1.16E+08	1000	COG2440	C	Ferredoxin-like protein	K03855	METABOLISM
PA2957		probable transcriptional regulator	1.67E+08	1.62E+08	0.97071					
PA2962	tmk	thymidylate kinase	32320274	24769320	0.766371	COG0125	F	Thymidylate kinase	K00943	METABOLISM
PA2966	acpP	acyl carrier protein	2.63E+08	2.16E+08	0.822717	COG0236	Q	Acyl carrier protein	K02078	METABOLISM
PA2966	acpP	acyl carrier protein	2.63E+08	2.16E+08	0.822717	COG0236	Q	Acyl carrier protein	K14188	METABOLISM
PA2966	acpP	acyl carrier protein	2.63E+08	2.16E+08	0.822717	COG0236	I	Acyl carrier protein	K02078	METABOLISM
PA2966	acpP	acyl carrier protein	2.63E+08	2.16E+08	0.822717	COG0236	I	Acyl carrier protein	K14188	METABOLISM
PA2967	fabG	3-oxoacyl-[acyl-carrier-protein] reductase	4.59E+09	1.26E+09	0.275097	COG1028	Q	Dehydrogenases with different specificities (related to short-c	K00059	METABOLISM
PA2967	fabG	3-oxoacyl-[acyl-carrier-protein] reductase	4.59E+09	1.26E+09	0.275097	COG1028	I	Dehydrogenases with different specificities (related to short-c	K00059	METABOLISM
PA2967	fabG	3-oxoacyl-[acyl-carrier-protein] reductase	4.59E+09	1.26E+09	0.275097	COG1028	R	Dehydrogenases with different specificities (related to short-c	K00059	POORLY CHARACTERIZED
PA2968	fabD	malonyl-CoA-[acyl-carrier-protein] transacylase	1.33E+08	6.88E+08	5.171332	COG0331	I	(acyl-carrier-protein) S-malonyltransferase	K00645	METABOLISM
PA2968	fabD	malonyl-CoA-[acyl-carrier-protein] transacylase	1.33E+08	6.88E+08	5.171332	COG0331	I	(acyl-carrier-protein) S-malonyltransferase	K11533	METABOLISM
PA2970	rpmF	50S ribosomal protein L32	3.12E+10	2.96E+10	0.946703	COG0333	J	Ribosomal protein L32	K02911	INFORMATION STORAGE AND PROCESSING
PA2976	rne	ribonuclease E	2.05E+08	1.21E+09	5.913099	COG1530	J	Ribonucleases G and E	K08300	INFORMATION STORAGE AND PROCESSING
PA2976	rne	ribonuclease E	2.05E+08	1.21E+09	5.913099	COG1530	J	Ribonucleases G and E	K08301	INFORMATION STORAGE AND PROCESSING
PA2980		conserved hypothetical protein	4.33E+09	2.82E+09	0.650631	COG2835	S	Uncharacterized conserved protein	K09791	POORLY CHARACTERIZED
PA3001		probable glyceraldehyde-3-phosphate dehydrogenase	1.05E+08	2.92E+09	27.7279	COG0057	G	Glyceraldehyde-3-phosphate dehydrogenase/erythrose-4-phc	K03472	METABOLISM
PA3001		probable glyceraldehyde-3-phosphate dehydrogenase	1.05E+08	2.92E+09	27.7279	COG0057	G	Glyceraldehyde-3-phosphate dehydrogenase/erythrose-4-phc	K00150	METABOLISM
PA3001		probable glyceraldehyde-3-phosphate dehydrogenase	1.05E+08	2.92E+09	27.7279	COG0057	G	Glyceraldehyde-3-phosphate dehydrogenase/erythrose-4-phc	K00134	METABOLISM
PA3003		hypothetical protein	1.87E+08	2.35E+08	1.258991					
PA3009		hypothetical protein	64967836	87854069	1.35227					
PA3013	faoB	fatty-acid oxidation complex beta-subunit	0	2.06E+08	1000	COG0183	I	Acetyl-CoA acetyltransferase	K00632	METABOLISM
PA3013	faoB	fatty-acid oxidation complex beta-subunit	0	2.06E+08	1000	COG0183	I	Acetyl-CoA acetyltransferase	K00626	METABOLISM
PA3014	faoA	fatty-acid oxidation complex alpha-subunit	0	1.66E+08	1000	COG1024	I	Enoyl-CoA hydratase/carnithine racemase	K01692	METABOLISM
PA3014	faoA	fatty-acid oxidation complex alpha-subunit	0	1.66E+08	1000	COG1250	I	3-hydroxyacyl-CoA dehydrogenase	K00074	METABOLISM
PA3014	faoA	fatty-acid oxidation complex alpha-subunit	0	1.66E+08	1000	COG1250	I	3-hydroxyacyl-CoA dehydrogenase	K01782	METABOLISM
PA3014	faoA	fatty-acid oxidation complex alpha-subunit	0	1.66E+08	1000	COG1250	I	3-hydroxyacyl-CoA dehydrogenase	K01825	METABOLISM
PA3014	faoA	fatty-acid oxidation complex alpha-subunit	0	1.66E+08	1000	COG1250	I	3-hydroxyacyl-CoA dehydrogenase	K07516	METABOLISM
PA3022		hypothetical protein	0	67341969	1000	COG3217	R	Uncharacterized Fe-S protein	K07140	POORLY CHARACTERIZED
PA3024		probable carbohydrate kinase	0	60210670	1000	COG1070	G	Sugar (pentulose and hexulose) kinases	K00851	METABOLISM
PA3024		probable carbohydrate kinase	0	60210670	1000	COG1070	G	Sugar (pentulose and hexulose) kinases	K00854	METABOLISM
PA3024		probable carbohydrate kinase	0	60210670	1000	COG1070	G	Sugar (pentulose and hexulose) kinases	K00880	METABOLISM
PA3024		probable carbohydrate kinase	0	60210670	1000	COG1070	G	Sugar (pentulose and hexulose) kinases	K00848	METABOLISM
PA3031		hypothetical protein	0	3.41E+08	1000					
PA3040		conserved hypothetical protein	1.09E+08	1.09E+08	1.000734	COG4575	S	Uncharacterized conserved protein	K05594	POORLY CHARACTERIZED
PA3049	rmf	ribosome modulation factor	1.48E+08	55822468	0.376551	COG3130	J	Ribosome modulation factor	K03812	INFORMATION STORAGE AND PROCESSING
PA3058	pelG	PelG	8931413	0	0					
PA3061	pelD	PelD(cyclic diguanylate effector involved in pellicle formation)	0	1.08E+08	1000	COG2203	T	FOG: GAF domain	K00936	CELLULAR PROCESSES AND SIGNALING
PA3062	pelC	PelC	27330860	4.08E+08	14.9424					
PA3074		hypothetical protein	35028585	0	0	COG2304	R	Uncharacterized protein containing a von Willebrand factor ty	K07114	POORLY CHARACTERIZED
PA3085		hypothetical protein	19591397	0	0	COG3139	S	Uncharacterized protein conserved in bacteria	K09916	POORLY CHARACTERIZED
PA3101	xcpT	general secretion pathway protein G	7.64E+08	2.42E+08	0.317276	COG2165	U	"Type II secretory pathway, pseudopilin PulG"	K02457	CELLULAR PROCESSES AND SIGNALING
PA3101	xcpT	general secretion pathway protein G	7.64E+08	2.42E+08	0.317276	COG2165	U	"Type II secretory pathway, pseudopilin PulG"	K02679	CELLULAR PROCESSES AND SIGNALING
PA3101	xcpT	general secretion pathway protein G	7.64E+08	2.42E+08	0.317276	COG2165	N	"Type II secretory pathway, pseudopilin PulG"	K02458	CELLULAR PROCESSES AND SIGNALING
PA3101	xcpT	general secretion pathway protein G	7.64E+08	2.42E+08	0.317276	COG2165	U	"Type II secretory pathway, pseudopilin PulG"	K02246	CELLULAR PROCESSES AND SIGNALING
PA3101	xcpT	general secretion pathway protein G	7.64E+08	2.42E+08	0.317276	COG2165	U	"Type II secretory pathway, pseudopilin PulG"	K02456	CELLULAR PROCESSES AND SIGNALING
PA3101	xcpT	general secretion pathway protein G	7.64E+08	2.42E+08	0.317276	COG2165	U	"Type II secretory pathway, pseudopilin PulG"	K02457	CELLULAR PROCESSES AND SIGNALING
PA3101	xcpT	general secretion pathway protein G	7.64E+08	2.42E+08	0.317276	COG2165	N	"Type II secretory pathway, pseudopilin PulG"	K02246	CELLULAR PROCESSES AND SIGNALING
PA3101	xcpT	general secretion pathway protein G	7.64E+08	2.42E+08	0.317276	COG2165	N	"Type II secretory pathway, pseudopilin PulG"	K02456	CELLULAR PROCESSES AND SIGNALING
PA3101	xcpT	general secretion pathway protein G	7.64E+08	2.42E+08	0.317276	COG2165	N	"Type II secretory pathway, pseudopilin PulG"	K02246	CELLULAR PROCESSES AND SIGNALING
PA3101	xcpT	general secretion pathway protein G	7.64E+08	2.42E+08	0.317276	COG2165	N	"Type II secretory pathway, pseudopilin PulG"	K02679	CELLULAR PROCESSES AND SIGNALING
PA3101	xcpT	general secretion pathway protein G	7.64E+08	2.42E+08	0.317276	COG2165	U	"Type II secretory pathway, pseudopilin PulG"	K02458	CELLULAR PROCESSES AND SIGNALING
PA3101	xcpT	general secretion pathway protein G	7.64E+08	2.42E+08	0.317276	COG2165	U	"Type II secretory pathway, pseudopilin PulG"	K02679	CELLULAR PROCESSES AND SIGNALING
PA3101	xcpT	general secretion pathway protein G	7.64E+08	2.42E+08	0.317276	COG2165	U	"Type II secretory pathway, pseudopilin PulG"	K02456	CELLULAR PROCESSES AND SIGNALING
PA3101	xcpT	general secretion pathway protein G	7.64E+08	2.42E+08	0.317276	COG2165	U	"Type II secretory pathway, pseudopilin PulG"	K02246	CELLULAR PROCESSES AND SIGNALING
PA3101	xcpT	general secretion pathway protein G	7.64E+08	2.42E+08	0.317276	COG2165	U	"Type II secretory pathway, pseudopilin PulG"	K02458	CELLULAR PROCESSES AND SIGNALING
PA3101	xcpT	general secretion pathway protein G	7.64E+08	2.42E+08	0.317276	COG2165	N	"Type II secretory pathway, pseudopilin PulG"	K02679	CELLULAR PROCESSES AND SIGNALING
PA3101	xcpT	general secretion pathway protein G	7.64E+08	2.42E+08	0.317276	COG2165	N	"Type II secretory pathway, pseudopilin PulG"	K02458	CELLULAR PROCESSES AND SIGNALING
PA3101	xcpT	general secretion pathway protein G	7.64E+08	2.42E+08	0.317276	COG2165	N	"Type II secretory pathway, pseudopilin PulG"	K02457	CELLULAR PROCESSES AND SIGNALING
PA3101	xcpT	general secretion pathway protein G	7.64E+08	2.42E+08	0.317276	COG2165	U	"Type II secretory pathway, pseudopilin PulG"	K02679	CELLULAR PROCESSES AND SIGNALING
PA3101	xcpT	general secretion pathway protein G	7.64E+08	2.42E+08	0.317276	COG2165	U	"Type II secretory pathway, pseudopilin PulG"	K02246	CELLULAR PROCESSES AND SIGNALING
PA3101	xcpT	general secretion pathway protein G	7.64E+08	2.42E+08	0.317276	COG2165	U	"Type II secretory pathway, pseudopilin PulG"	K02458	CELLULAR PROCESSES AND SIGNALING
PA3101	xcpT	general secretion pathway protein G	7.64E+08	2.42E+08	0.317276	COG2165	U	"Type II secretory pathway, pseudopilin PulG"	K02456	CELLULAR PROCESSES AND SIGNALING
PA3101	xcpT	general secretion pathway protein G	7.64E+08	2.42E+08	0.317276	COG2165	N	"Type II secretory pathway, pseudopilin PulG"	K02679	CELLULAR PROCESSES AND SIGNALING
PA3101	xcpT	general secretion pathway protein G	7.64E+08	2.42E+08	0.317276	COG2165	N	"Type II secretory pathway, pseudopilin PulG"	K02679	CELLULAR PROCESSES AND SIGNALING
PA3101	xcpT	general secretion pathway protein G	7.64E+08	2.42E+08	0.317276	COG2165	N	"Type II secretory pathway, pseudopilin PulG"	K02246	CELLULAR PROCESSES AND SIGNALING
PA3101	xcpT	general secretion pathway protein G	7.64E+08	2.42E+08	0.317276	COG2165	N	"Type II secretory pathway, pseudopilin PulG"	K02456	CELLULAR PROCESSES AND SIGNALING
PA3101	xcpT	general secretion pathway protein G	7.64E+08	2.42E+08	0.317276	COG2165	N	"Type II secretory pathway, pseudopilin PulG"	K02246	CELLULAR PROCESSES AND SIGNALING
PA3101	xcpT	general secretion pathway protein G	7.64E+08	2.42E+08	0.317276	COG2165	U	"Type II secretory pathway, pseudopilin PulG"	K02679	CELLULAR PROCESSES AND SIGNALING
PA3101	xcpT	general secretion pathway protein G	7.64E+08	2.42E+08	0.317276	COG2165	N	"Type II secretory pathway, pseudopilin PulG"	K02458	CELLULAR PROCESSES AND SIGNALING
PA3101	xcpT	general secretion pathway protein G	7.64E+08	2.42E+08	0.317276	COG2165	N	"Type II secretory pathway, pseudopilin PulG"	K02457	CELLULAR PROCESSES AND SIGNALING
PA3101	xcpT	general secretion pathway protein G	7.64E+08	2.42E+08	0.317276	COG2165	N	"Type II secretory pathway, pseudopilin PulG"	K02456	CELLULAR PROCESSES AND SIGNALING
PA3101	xcpT	general secretion pathway protein G	7.64E+08	2.42E+08	0.317276	COG2165	N	"Type II secretory pathway, pseudopilin PulG"	K02456	CELLULAR PROCESSES AND SIGNALING
PA3101	xcpT	general secretion pathway protein G	7.64E+08	2.42E+08	0.317276	COG2165	N	"Type II secretory pathway, pseudopilin PulG"	K02457	CELLULAR PROCESSES AND SIGNALING
PA3101	xcpT	general secretion pathway protein G	7.64E+08	2.42E+08	0.317276	COG2165	U	"Type II secretory pathway, pseudopilin PulG"	K02457	CELLULAR PROCESSES AND SIGNALING
PA3101	xcpT	general secretion pathway protein G	7.64E+08	2.42E+08	0.317276	COG2165	U	"Type II secretory pathway, pseudopilin PulG"	K02246	CELLULAR PROCESSES AND SIGNALING
PA3101	xcpT	general secretion pathway protein G	7.64E+08	2.42E+08	0.317276	COG2165	U	"Type II secretory pathway, pseudopilin PulG"	K02456	CELLULAR PROCESSES AND SIGNALING
PA3101	xcpT	general secretion pathway protein G	7.64E+08	2.42E+08	0.317276	COG2165	N	"Type II secretory pathway, pseudopilin PulG"	K02458	CELLULAR PROCESSES AND SIGNALING
PA3101	xcpT	general secretion pathway protein G	7.64E+08	2.42E+08	0.317276	COG2165	U	"Type II secretory pathway, pseudopilin PulG"	K02458	CELLULAR PROCESSES AND SIGNALING
PA3101	xcpT	general secretion pathway protein G	7.64E+08	2.42E+08	0.317276	COG2165	N	"Type II secretory pathway, pseudopilin PulG"	K02457	CELLULAR PROCESSES AND SIGNALING
PA3101	xcpT	general secretion pathway protein G	7.64E+08	2.42E+08	0.317276	COG2165	U	"Type II secretory pathway, pseudopilin PulG"	K02457	CELLULAR PROCESSES AND SIGNALING
PA3104	xcpP	secretion protein XcpP	25088994	0	0					
PA3110		hypothetical protein	0	26566183	1000	COG3147	S	Uncharacterized protein conserved in bacteria	K03749	POORLY CHARACTERIZED
PA3115	fimV	Motility protein FimV	3.92E+09	1.68E+09	0.428129	COG3170	N	Tfp pilus assembly protein FimV	K07288	CELLULAR PROCESSES AND SIGNALING
PA3115	fimV	Motility protein FimV	3.92E+09	1.68E+09	0.428129	COG3170	U	Tfp pilus assembly protein FimV	K07288	CELLULAR PROCESSES AND SIGNALING
PA3117	asd	aspartate semialdehyde dehydrogenase	99798993	0	0	COG0136	E	Aspartate-semialdehyde dehydrogenase	K00133	METABOLISM
PA3120	leuD	3-isopropylmalate dehydratase small subunit	7983785	1.73E+09	216.1628	COG0066	E	3-isopropylmalate dehydratase small subunit	K01704	METABOLISM
PA3126	ibpA	heat-shock protein IbpA	77454597	9.18E+08	11.84847	COG0071	O	Molecular chaperone (small heat shock protein)	K04080	CELLULAR PROCESSES AND SIGNALING
PA3126	ibpA	heat-shock protein IbpA	77454597	9.18E+08	11.84847	COG0071	O	Molecular chaperone (small heat shock protein)	K13993	CELLULAR PROCESSES AND SIGNALING
PA3148	wbpI	UDP-N-acetylglucosamine 2-epimerase WbpI	0	37075616	1000	COG0381	M	UDP-N-acetylglucosamine 2-epimerase	K01791	CELLULAR PROCESSES AND SIGNALING
PA3158	wbpB	"UDP-2-acetamido-2-deoxy-d-glucuronic acid 3-dehydrogenase	0	1.38E+08	1000	COG0673	R	Predicted dehydrogenases and related proteins	K00010	POORLY CHARACTERIZED
PA3159	wbpA	UDP-N-acetyl-d-glucosamine 6-Dehydrogenase	0	56044235	1000	COG0677	M	UDP-N-acetyl-D-mannosaminuronate dehydrogenase	K13015	CELLULAR PROCESSES AND SIGNALING
PA3159	wbpA	UDP-N-acetyl-d-glucosamine 6-Dehydrogenase	0	56044235	1000	COG0677	M	UDP-N-acetyl-D-mannosaminuronate dehydrogenase	K02474	CELLULAR PROCESSES AND SIGNALING
PA3159	wbpA	UDP-N-acetyl-d-glucosamine 6-Dehydrogenase	0	56044235	1000	COG0677	M	UDP-N-acetyl-D-mannosaminuronate dehydrogenase	K02472	CELLULAR PROCESSES AND SIGNALING
PA3161	himD	integration host factor beta subunit	99418437	0	0	COG0776	L	Bacterial nucleoid DNA-binding protein	K05788	INFORMATION STORAGE AND PROCESSING
PA3161	himD	integration host factor beta subunit	99418437	0	0	COG0776	L	Bacterial nucleoid DNA-binding protein	K03530	INFORMATION STORAGE AND PROCESSING

PA3161	himD	integration host factor beta subunit	99418437	0	0	COG0776	L	Bacterial nucleoid DNA-binding protein	K05787	INFORMATION STORAGE AND PROCESSING
PA3161	himD	integration host factor beta subunit	99418437	0	0	COG0776	L	Bacterial nucleoid DNA-binding protein	K04764	INFORMATION STORAGE AND PROCESSING
PA3162	rpsA	30S ribosomal protein S1	7.48E+09	9.73E+09	1.300511	COG0539	J	Ribosomal protein S1	K02945	INFORMATION STORAGE AND PROCESSING
PA3162	rpsA	30S ribosomal protein S1	7.48E+09	9.73E+09	1.300511	COG1093	J	"Translation initiation factor 2, alpha subunit (eIF-2alpha)"	K03237	INFORMATION STORAGE AND PROCESSING
PA3163	cmk	cytidylate kinase	17195360	35565106	2.068297	COG0283	F	Cytidylate kinase	K00945	METABOLISM
PA3163	cmk	cytidylate kinase	17195360	35565106	2.068297	COG0283	F	Cytidylate kinase	K13799	METABOLISM
PA3166	pheA	chorismate mutase	0	21172882	1000	COG1605	E	Chorismate mutase	K14170	METABOLISM
PA3166	pheA	chorismate mutase	0	21172882	1000	COG1605	E	Chorismate mutase	K04516	METABOLISM
PA3166	pheA	chorismate mutase	0	21172882	1000	COG1605	E	Chorismate mutase	K04782	METABOLISM
PA3166	pheA	chorismate mutase	0	21172882	1000	COG0077	E	Prephenate dehydratase	K14170	METABOLISM
PA3166	pheA	chorismate mutase	0	21172882	1000	COG0077	E	Prephenate dehydratase	K04518	METABOLISM
PA3166	pheA	chorismate mutase	0	21172882	1000	COG1605	E	Chorismate mutase	K04093	METABOLISM
PA3166	pheA	chorismate mutase	0	21172882	1000	COG1605	E	Chorismate mutase	K14187	METABOLISM
PA3166	pheA	chorismate mutase	0	21172882	1000	COG1605	E	Chorismate mutase	K01850	METABOLISM
PA3166	pheA	chorismate mutase	0	21172882	1000	COG1605	E	Chorismate mutase	K04092	METABOLISM
PA3166	pheA	chorismate mutase	0	21172882	1000	COG1605	E	Chorismate mutase	K13853	METABOLISM
PA3171	ubiG	3-demethylubiquinone-9 3-methyltransferase	71352383	59238994	0.830231	COG2227	H	"2-polyprenyl-3-methyl-5-hydroxy-6-metoxy-1,4-benzoquinol	K00568	METABOLISM
PA3171	ubiG	3-demethylubiquinone-9 3-methyltransferase	71352383	59238994	0.830231	COG2227	H	"2-polyprenyl-3-methyl-5-hydroxy-6-metoxy-1,4-benzoquinol	K03428	METABOLISM
PA3172		probable hydrolase	55431859	0	0	COG0546	R	Predicted phosphatases	K01091	POORLY CHARACTERIZED
PA3186	oprB	Glucose/carbohydrate outer membrane porin OprB precursor	0	1.07E+08	1000	COG3659	M	Carbohydrate-selective porin	K07267	CELLULAR PROCESSES AND SIGNALING
PA3190		probable binding protein component of ABC sugar transporter	5.92E+08	2.78E+08	0.468793	COG1653	G	"ABC-type sugar transport system, periplasmic component"	K02027	METABOLISM
PA3190		probable binding protein component of ABC sugar transporter	5.92E+08	2.78E+08	0.468793	COG1653	G	"ABC-type sugar transport system, periplasmic component"	K05813	METABOLISM
PA3194	edd	phosphogluconate dehydratase	24552837	93528455	3.809273	COG0129	G	Dihydroxyacid dehydratase/phosphogluconate dehydratase	K01687	METABOLISM
PA3194	edd	phosphogluconate dehydratase	24552837	93528455	3.809273	COG0129	G	Dihydroxyacid dehydratase/phosphogluconate dehydratase	K01690	METABOLISM
PA3194	edd	phosphogluconate dehydratase	24552837	93528455	3.809273	COG0129	E	Dihydroxyacid dehydratase/phosphogluconate dehydratase	K01690	METABOLISM
PA3194	edd	phosphogluconate dehydratase	24552837	93528455	3.809273	COG0129	E	Dihydroxyacid dehydratase/phosphogluconate dehydratase	K01687	METABOLISM
PA3202		conserved hypothetical protein	59972792	1.55E+08	2.588321	COG2350	S	Uncharacterized protein conserved in bacteria	K09780	POORLY CHARACTERIZED
PA3205		hypothetical protein	0	1.61E+08	1000	COG3678	P	"Pilus assembly/Cpx signaling pathway, periplasmic inhibitor/	K07803	METABOLISM
PA3205		hypothetical protein	0	1.61E+08	1000	COG3678	N	"Pilus assembly/Cpx signaling pathway, periplasmic inhibitor/	K06006	CELLULAR PROCESSES AND SIGNALING
PA3205		hypothetical protein	0	1.61E+08	1000	COG3678	U	"Pilus assembly/Cpx signaling pathway, periplasmic inhibitor/	K07803	CELLULAR PROCESSES AND SIGNALING
PA3205		hypothetical protein	0	1.61E+08	1000	COG3678	N	"Pilus assembly/Cpx signaling pathway, periplasmic inhibitor/	K07803	CELLULAR PROCESSES AND SIGNALING
PA3205		hypothetical protein	0	1.61E+08	1000	COG3678	U	"Pilus assembly/Cpx signaling pathway, periplasmic inhibitor/	K06006	CELLULAR PROCESSES AND SIGNALING
PA3205		hypothetical protein	0	1.61E+08	1000	COG3678	P	"Pilus assembly/Cpx signaling pathway, periplasmic inhibitor/	K06006	METABOLISM
PA3205		hypothetical protein	0	1.61E+08	1000	COG3678	T	"Pilus assembly/Cpx signaling pathway, periplasmic inhibitor/	K06006	CELLULAR PROCESSES AND SIGNALING
PA3205		hypothetical protein	0	1.61E+08	1000	COG3678	T	"Pilus assembly/Cpx signaling pathway, periplasmic inhibitor/	K07803	CELLULAR PROCESSES AND SIGNALING
PA3213		hypothetical protein	12850246	0	0	COG1463	Q	"ABC-type transport system involved in resistance to organic s	K02067	METABOLISM
PA3214		hypothetical protein	96749987	56244321	0.581337					
PA3227	ppiA	peptidyl-prolyl cis-trans isomerase A	3.11E+08	2.59E+08	0.833482	COG0652	O	Peptidyl-prolyl cis-trans isomerase (rotamase) - cyclophilin fam	K03768	CELLULAR PROCESSES AND SIGNALING
PA3227	ppiA	peptidyl-prolyl cis-trans isomerase A	3.11E+08	2.59E+08	0.833482	COG0652	O	Peptidyl-prolyl cis-trans isomerase (rotamase) - cyclophilin fam	K03767	CELLULAR PROCESSES AND SIGNALING
PA3227	ppiA	peptidyl-prolyl cis-trans isomerase A	3.11E+08	2.59E+08	0.833482	COG0652	O	Peptidyl-prolyl cis-trans isomerase (rotamase) - cyclophilin fam	K01802	CELLULAR PROCESSES AND SIGNALING
PA3244	minD	cell division inhibitor MinD	36262041	4.58E+08	12.62021	COG2894	D	Septum formation inhibitor-activating ATPase	K03609	CELLULAR PROCESSES AND SIGNALING
PA3245	minE	cell division topological specificity factor MinE	2.04E+09	4.11E+08	0.201214	COG0851	D	Septum formation topological specificity factor	K03608	CELLULAR PROCESSES AND SIGNALING
PA3262		"probable peptidyl-prolyl cis-trans isomerase	1.67E+10	1.11E+10	0.663046	COG0545	O	FKBP-type peptidyl-prolyl cis-trans isomerases 1	K03772	CELLULAR PROCESSES AND SIGNALING
PA3262		"probable peptidyl-prolyl cis-trans isomerase	1.67E+10	1.11E+10	0.663046	COG0545	O	FKBP-type peptidyl-prolyl cis-trans isomerases 1	K03773	CELLULAR PROCESSES AND SIGNALING
PA3262		"probable peptidyl-prolyl cis-trans isomerase	1.67E+10	1.11E+10	0.663046	COG0545	O	FKBP-type peptidyl-prolyl cis-trans isomerases 1	K09575	CELLULAR PROCESSES AND SIGNALING
PA3262		"probable peptidyl-prolyl cis-trans isomerase	1.67E+10	1.11E+10	0.663046	COG0545	O	FKBP-type peptidyl-prolyl cis-trans isomerases 1	K01802	CELLULAR PROCESSES AND SIGNALING
PA3263		conserved hypothetical protein	0	51112833	1000	COG2974	L	DNA recombination-dependent growth factor C	K03554	INFORMATION STORAGE AND PROCESSING
PA3264		probable transporter	0	86277767	1000	COG0385	R	Predicted Na+dependent transporter	K03453	POORLY CHARACTERIZED
PA3266	capB	cold acclimation protein B	1.12E+10	8.98E+09	0.803197	COG1278	K	Cold shock proteins	K03704	INFORMATION STORAGE AND PROCESSING
PA3302		conserved hypothetical protein	1.74E+08	0	0					
PA3309		conserved hypothetical protein	1.47E+10	6.08E+09	0.413043	COG0589	T	Universal stress protein UspA and related nucleotide-binding p	K06149	CELLULAR PROCESSES AND SIGNALING
PA3313		hypothetical protein	26062906	41124770	1.577904	COG3221	P	"ABC-type phosphate/phosphonate transport system, peripla	K02044	METABOLISM
PA3326	clpP2	ClpP2	4.38E+08	2.47E+08	0.565238	COG0740	O	Protease subunit of ATP-dependent Clp proteases	K01358	CELLULAR PROCESSES AND SIGNALING
PA3326	clpP2	ClpP2	4.38E+08	2.47E+08	0.565238	COG0740	U	Protease subunit of ATP-dependent Clp proteases	K01358	CELLULAR PROCESSES AND SIGNALING
PA3330		probable short chain dehydrogenase	14706552	0	0	COG1028	R	Dehydrogenases with different specificities (related to short-c	K00059	POORLY CHARACTERIZED
PA3330		probable short chain dehydrogenase	14706552	0	0	COG1028	I	Dehydrogenases with different specificities (related to short-c	K00059	METABOLISM
PA3330		probable short chain dehydrogenase	14706552	0	0	COG1028	Q	Dehydrogenases with different specificities (related to short-c	K00059	METABOLISM
PA3341		probable transcriptional regulator	62821958	1.4E+08	2.226162	COG1846	K	Transcriptional regulators	K03712	INFORMATION STORAGE AND PROCESSING
PA3347	hsbA	HptB-dependent secretion and biofilm anti anti-sigma factor HsbA	1.59E+08	1.85E+08	1.166461	COG1366	T	Anti-anti-sigma regulatory factor (antagonist of anti-sigma fac	K06378	CELLULAR PROCESSES AND SIGNALING
PA3347	hsbA	HptB-dependent secretion and biofilm anti anti-sigma factor HsbA	1.59E+08	1.85E+08	1.166461	COG1366	T	Anti-anti-sigma regulatory factor (antagonist of anti-sigma fac	K04749	CELLULAR PROCESSES AND SIGNALING
PA3351	flgM	FlgM	6.02E+08	2.65E+08	0.440457	COG2747	K	Negative regulator of flagellin synthesis (anti-sigma28 factor)	K02398	INFORMATION STORAGE AND PROCESSING
PA3351	flgM	FlgM	6.02E+08	2.65E+08	0.440457	COG2747	U	Negative regulator of flagellin synthesis (anti-sigma28 factor)	K02398	CELLULAR PROCESSES AND SIGNALING
PA3351	flgM	FlgM	6.02E+08	2.65E+08	0.440457	COG2747	N	Negative regulator of flagellin synthesis (anti-sigma28 factor)	K02398	CELLULAR PROCESSES AND SIGNALING
PA3352		hypothetical protein	5.05E+08	2.17E+09	4.297886					
PA3378		conserved hypothetical protein	0	47502093	1000	COG3626	P	Uncharacterized enzyme of phosphonate metabolism	K06164	METABOLISM
PA3385	amrZ	alginate and motility regulator Z	6.76E+08	1.99E+08	0.294851					
PA3392	nosZ	nitrous-oxide reductase precursor	0	3E+08	1000	COG4263	C	Nitrous oxide reductase	K00376	METABOLISM
PA3413		conserved hypothetical protein	3.37E+08	1.19E+08	0.35245	COG3141	S	Uncharacterized protein conserved in bacteria	K09918	POORLY CHARACTERIZED
PA3424		hypothetical protein	63567340	0	0	COG0665	E	Glycine/D-amino acid oxidases (deaminating)	K00303	METABOLISM
PA3424		hypothetical protein	63567340	0	0	COG0665	E	Glycine/D-amino acid oxidases (deaminating)	K00285	METABOLISM
PA3424		hypothetical protein	63567340	0	0	COG0665	E	Glycine/D-amino acid oxidases (deaminating)	K03153	METABOLISM
PA3435		conserved hypothetical protein	2.3E+08	1.48E+08	0.642493	COG0716	C	Flavodoxins	K03839	METABOLISM
PA3435		conserved hypothetical protein	2.3E+08	1.48E+08	0.642493	COG0716	C	Flavodoxins	K03840	METABOLISM
PA3435		conserved hypothetical protein	2.3E+08	1.48E+08	0.642493	COG0716	C	Flavodoxins	K06205	METABOLISM
PA3440		conserved hypothetical protein	62703626	0	0	COG3492	S	Uncharacterized protein conserved in bacteria	K09948	POORLY CHARACTERIZED
PA3441		probable molybdopterin-binding protein	0	76128761	1000					
PA3452	mqoA	malate:quinone oxidoreductase	33561594	1.5E+08	4.465369	COG0579	R	Predicted dehydrogenase	K00111	POORLY CHARACTERIZED
PA3452	mqoA	malate:quinone oxidoreductase	33561594	1.5E+08	4.465369	COG0579	R	Predicted dehydrogenase	K00116	POORLY CHARACTERIZED
PA3453		conserved hypothetical protein	2.24E+08	39697968	0.176908	COG3132	S	Uncharacterized protein conserved in bacteria	K03810	POORLY CHARACTERIZED
PA3453		conserved hypothetical protein	2.24E+08	39697968	0.176908	COG3132	S	Uncharacterized protein conserved in bacteria	K09915	POORLY CHARACTERIZED
PA3458		probable transcriptional regulator	39246430	0	0	COG1846	K	Transcriptional regulators	K03712	INFORMATION STORAGE AND PROCESSING
PA3470		hypothetical protein	0	50476592	1000	COG0494	R	NTP pyrophosphohydrolases including oxidative damage repa	K03574	POORLY CHARACTERIZED
PA3470		hypothetical protein	0	50476592	1000	COG0494	L	NTP pyrophosphohydrolases including oxidative damage repa	K01515	INFORMATION STORAGE AND PROCESSING
PA3470		hypothetical protein	0	50476592	1000	COG0494	R	NTP pyrophosphohydrolases including oxidative damage repa	K01515	POORLY CHARACTERIZED
PA3470		hypothetical protein	0	50476592	1000	COG0494	L	NTP pyrophosphohydrolases including oxidative damage repa	K08311	INFORMATION STORAGE AND PROCESSING
PA3470		hypothetical protein	0	50476592	1000	COG0494	L	NTP pyrophosphohydrolases including oxidative damage repa	K03574	INFORMATION STORAGE AND PROCESSING
PA3470		hypothetical protein	0	50476592	1000	COG0494	R	NTP pyrophosphohydrolases including oxidative damage repa	K08311	POORLY CHARACTERIZED
PA3482	metG	methionyl-tRNA synthetase	0	15240747	1000	COG0073	R	EMAP domain	K01890	POORLY CHARACTERIZED
PA3482	metG	methionyl-tRNA synthetase	0	15240747	1000	COG0143	J	Methionyl-tRNA synthetase	K01874	INFORMATION STORAGE AND PROCESSING
PA3482	metG	methionyl-tRNA synthetase	0	15240747	1000	COG0073	R	EMAP domain	K06878	POORLY CHARACTERIZED
PA3482	metG	methionyl-tRNA synthetase	0	15240747	1000	COG0073	R	EMAP domain	K01874	POORLY CHARACTERIZED
PA3496		hypothetical protein	1.91E+08	1.34E+08	0.700394					
PA3520		hypothetical protein	59609396	1.27E+08	2.125457	COG2608	P	Copper chaperone	K08364	METABOLISM
PA3520		hypothetical protein	59609396	1.27E+08	2.125457	COG2608	P	Copper chaperone	K07213	METABOLISM
PA3525	argG	argininosuccinate synthase	22160553	2.51E+08	11.34411	COG0137	E	Argininosuccinate synthase	K01940	METABOLISM
PA3529		alkylhydroperoxide reductase C	1.27E+09	5.08E+09	3.994497	COG0450	O	Peroxiredoxin	K03386	CELLULAR PROCESSES AND SIGNALING
PA3531	bfrB	bacterioferritin	2.91E+09	2.77E+09	0.953468	COG2193	P	Bacterioferritin (cytochrome b1)	K03594	METABOLISM
PA3533	grxD	GrxD	1.21E+09	5.08E+08	0.420679	COG0278	O	Glutaredoxin-related protein	K07390	CELLULAR PROCESSES AND SIGNALING
PA3535		probable serine protease	0	1.03E+08	1000	COG1404	O	Subtilisin-like serine proteases	K01362	CELLULAR PROCESSES AND SIGNALING
PA3535		probable serine protease	0	1.03E+08	1000	COG4625	S	Uncharacterized protein with a C-terminal OMP (outer memb	K12685	POORLY CHARACTERIZED
PA3561	fruK	1-phosphofructokinase	0	58266564	1000	COG1105	G	Fructose-1-phosphate kinase and related fructose-6-phosphat	K00917	METABOLISM
PA3561	fruK	1-phosphofructokinase	0	58266564	1000	COG1105	G	Fructose-1-phosphate kinase and related fructose-6-phosphat	K00882	METABOLISM
PA3561	fruK	1-phosphofructokinase	0	58266564	1000	COG1105	G	Fructose-1-phosphate kinase and related fructose-6-phosphat	K00850	METABOLISM
PA3562	fruI	"phosphotransferase system transporter enzyme I	2.5E+08	73648682	0.294729	COG1925	G	"Phosphotransferase system, HPr-related proteins"	K02784	METABOLISM
PA3562	fruI	"phosphotransferase system transporter enzyme I	2.5E+08	73648682	0.294729	COG1080	G	Phosphoenolpyruvate-protein kinase (PTS system EI compone	K11189	METABOLISM
PA3562	fruI	"phosphotransferase system transporter enzyme I	2.5E+08	73648682	0.294729	COG1080	G	Phosphoenolpyruvate-protein kinase (PTS system EI compone	K02768	METABOLISM
PA3562	fruI	"phosphotransferase system transporter enzyme I	2.5E+08	73648682	0.294729	COG1925	G	"Phosphotransferase system, HPr-related proteins"	K11189	METABOLISM
PA3562	fruI	"phosphotransferase system transporter enzyme I	2.5E+08	73648682	0.294729	COG1925	G	"Phosphotransferase system, HPr-related proteins"	K08483	METABOLISM
PA3562	fruI	"phosphotransferase system transporter enzyme I	2.5E+08	73648682	0.294729	COG4668	G	"Mannitol/fructose-specific phosphotransferase system, IIA do	K02799	METABOLISM
PA3562	fruI	"phosphotransferase system transporter enzyme I	2.5E+08	73648682	0.294729	COG1925	G	"Phosphotransferase system, HPr-related proteins"	K11183	METABOLISM
PA3562	fruI	"phosphotransferase system transporter enzyme I	2.5E+08	73648682	0.294729	COG1925	G	"Phosphotransferase system, HPr-related proteins"	K02768	METABOLISM
PA3562	fruI	"phosphotransferase system transporter enzyme I	2.5E+08	73648682	0.294729	COG1080	G	Phosphoenolpyruvate-protein kinase (PTS system EI compone	K11201	METABOLISM
PA3562	fruI	"phosphotransferase system transporter enzyme I	2.5E+08	73648682	0.294729	COG1080	G	Phosphoenolpyruvate-protein kinase (PTS system EI compone	K11183	METABOLISM
PA3562	fruI	"phosphotransferase system transporter enzyme I	2.5E+08	73648682	0.294729	COG4668	G	"Mannitol/fructose-specific phosphotransferase system, IIA do	K08483	METABOLISM
PA3562	fruI	"phosphotransferase system transporter enzyme I	2.5E+08	73648682	0.294729	COG1080	G	Phosphoenolpyruvate-protein kinase (PTS system EI compone	K08483	METABOLISM
PA3562	fruI	"phosphotransferase system transporter enzyme I	2.5E+08	73648682	0.294729	COG4668	G	"Mannitol/fructose-specific phosphotransferase system, IIA do	K02800	METABOLISM
PA3562	fruI	"phosphotransferase system transporter enzyme I	2.5E+08	73648682	0.294729	COG4668	G	"Mannitol/fructose-specific phosphotransferase system, IIA do	K02768	METABOLISM
PA3562	fruI	"phosphotransferase system transporter enzyme I	2.5E+08	73648682	0.294729	COG4668	G	"Mannitol/fructose-specific phosphotransferase system, IIA do	K11183	METABOLISM
PA3562	fruI	"phosphotransferase system transporter enzyme I	2.5E+08	73648682	0.294729	COG4668	G	"Mannitol/fructose-specific phosphotransferase system, IIA do	K02798	METABOLISM
PA3562	fruI	"phosphotransferase system transporter enzyme I	2.5E+08	73648682	0.294729	COG1925	G	"Phosphotransferase system, HPr-related proteins"	K08485	METABOLISM
PA3566		conserved hypothetical protein	4.92E+08	99349932	0.202015	COG1359	S	Uncharacterized conserved protein	K11530	POORLY CHARACTERIZED

PA3580		conserved hypothetical protein	0	22480144	1000	COG2606	S	Uncharacterized conserved protein	K03976	POORLY CHARACTERIZED
PA3592		conserved hypothetical protein	24681893	0	0	COG1804	C	Predicted acyl-CoA transferases/carnitine dehydratase	K01796	METABOLISM
PA3592		conserved hypothetical protein	24681893	0	0	COG1804	C	Predicted acyl-CoA transferases/carnitine dehydratase	K07749	METABOLISM
PA3601		conserved hypothetical protein	57306665	1.33E+08	2.31374	COG0254	J	Ribosomal protein L31	K02909	INFORMATION STORAGE AND PROCESSING
PA3602		conserved hypothetical protein	0	10273770	1000	COG0069	E	Glutamate synthase domain 2	K00265	METABOLISM
PA3602		conserved hypothetical protein	0	10273770	1000	COG0069	E	Glutamate synthase domain 2	K00284	METABOLISM
PA3611		hypothetical protein	74803396	87957800	1.175853					
PA3617	recA	RecA protein	1.15E+08	19263705	0.166855	COG0468	L	RecA/RadA recombinase	K03553	INFORMATION STORAGE AND PROCESSING
PA3617	recA	RecA protein	1.15E+08	19263705	0.166855	COG0468	L	RecA/RadA recombinase	K04483	INFORMATION STORAGE AND PROCESSING
PA3621	fdxA	ferredoxin I	1.54E+09	4.74E+08	0.307174	COG1146	C	Ferredoxin	K00176	METABOLISM
PA3621	fdxA	ferredoxin I	1.54E+09	4.74E+08	0.307174	COG1146	C	Ferredoxin	K05524	METABOLISM
PA3623		conserved hypothetical protein	0	54631284	1000	COG1388	M	FOG: LysM repeat	K01448	CELLULAR PROCESSES AND SIGNALING
PA3632		conserved hypothetical protein	28495140	24727315	0.867773	COG0425	O	"Predicted redox protein, regulator of disulfide bond formatio	K04085	CELLULAR PROCESSES AND SIGNALING
PA3635	eno	enolase	1.73E+10	8.08E+09	0.467164	COG0148	G	Enolase	K01689	METABOLISM
PA3636	kdsA	2-dehydro-3-deoxyphosphooctonate aldolase	0	9713513	1000	COG2877	M	3-deoxy-D-manno-octulosonic acid (KDO) 8-phosphate syntha	K01627	CELLULAR PROCESSES AND SIGNALING
PA3639	accA	acetyl-coenzyme A carboxylase carboxyl transferase (alpha subun	0	52949293	1000	COG0825	I	Acetyl-CoA carboxylase alpha subunit	K01963	METABOLISM
PA3639	accA	acetyl-coenzyme A carboxylase carboxyl transferase (alpha subun	0	52949293	1000	COG0825	I	Acetyl-CoA carboxylase alpha subunit	K01962	METABOLISM
PA3647		probable outer membrane protein precursor	6.36E+09	7.31E+09	1.150069	COG2825	M	Outer membrane protein	K06142	CELLULAR PROCESSES AND SIGNALING
PA3653	frr	ribosome recycling factor	1.12E+10	7.68E+09	0.683803	COG0233	J	Ribosome recycling factor	K02838	INFORMATION STORAGE AND PROCESSING
PA3654	pyrH	uridylate kinase	0	26470581	1000	COG0528	F	Uridylate kinase	K09903	METABOLISM
PA3655	tsf	elongation factor Ts	2.72E+09	4.68E+09	1.720306	COG0264	J	Translation elongation factor Ts	K02357	INFORMATION STORAGE AND PROCESSING
PA3656	rpsB	30S ribosomal protein S2	3.84E+09	4.86E+09	1.264095	COG0052	J	Ribosomal protein S2	K02967	INFORMATION STORAGE AND PROCESSING
PA3657	map	methionine aminopeptidase	59624232	2.4E+08	4.025924	COG0024	J	Methionine aminopeptidase	K01265	INFORMATION STORAGE AND PROCESSING
PA3664		conserved hypothetical protein	1.91E+08	0	0	COG1393	P	"Arsenate reductase and related proteins, glutaredoxin family	K00537	METABOLISM
PA3675		hypothetical protein	46621960	1.22E+08	2.610322					
PA3678	mexL	MexL	1.36E+08	1.01E+08	0.744297					
PA3686	adk	adenylate kinase	8.28E+09	3.7E+09	0.446546	COG0563	F	Adenylate kinase and related kinases	K00939	METABOLISM
PA3688		hypothetical protein	8.03E+08	1.43E+08	0.177959					
PA3691		hypothetical protein	1.35E+10	7.68E+09	0.567961					
PA3692	lptF	"Lipotoxon F	2.08E+10	7.16E+09	0.343562	COG2885	M	Outer membrane protein and related peptidoglycan-associate	K03286	CELLULAR PROCESSES AND SIGNALING
PA3692	lptF	"Lipotoxon F	2.08E+10	7.16E+09	0.343562	COG2885	M	Outer membrane protein and related peptidoglycan-associate	K03640	CELLULAR PROCESSES AND SIGNALING
PA3694		hypothetical protein	4.59E+08	3.69E+08	0.802985					
PA3700	lysS	lysyl-tRNA synthetase	0	1.27E+08	1000	COG1190	J	Lysyl-tRNA synthetase (class II)	K04567	INFORMATION STORAGE AND PROCESSING
PA3701	peptide chain release factor 2(prfB)		6.64E+08	0	0					
PA3701	peptide chain release factor 2(prfB)		31343769	0	0					
PA3708	wspA	probable chemotaxis transducer	1.01E+09	4.52E+08	0.448482	COG0840	N	Methyl-accepting chemotaxis protein	K03406	CELLULAR PROCESSES AND SIGNALING
PA3708	wspA	probable chemotaxis transducer	1.01E+09	4.52E+08	0.448482	COG0840	T	Methyl-accepting chemotaxis protein	K03406	CELLULAR PROCESSES AND SIGNALING
PA3712		hypothetical protein	1.27E+08	4.26E+08	3.350091					
PA3716		hypothetical protein	1.39E+08	1.96E+08	1.408854					
PA3729		conserved hypothetical protein	7.13E+08	6.51E+08	0.91363	COG2268	S	Uncharacterized protein conserved in bacteria	K07192	POORLY CHARACTERIZED
PA3731		conserved hypothetical protein	1.48E+09	3.91E+08	0.263703	COG1842	K	"Phage shock protein A (IM30), suppresses sigma54-depender	K03969	INFORMATION STORAGE AND PROCESSING
PA3731		conserved hypothetical protein	1.48E+09	3.91E+08	0.263703	COG1842	T	"Phage shock protein A (IM30), suppresses sigma54-depender	K03969	CELLULAR PROCESSES AND SIGNALING
PA3735	thrC	threonine synthase	2.65E+08	4.44E+08	1.674996	COG0498	E	Threonine synthase	K01733	METABOLISM
PA3736	hom	homoserine dehydrogenase	9774517	23722366	2.42696	COG0317	K	Guanosine polyphosphate pyrophosphohydrolases/synthetas	K01139	INFORMATION STORAGE AND PROCESSING
PA3736	hom	homoserine dehydrogenase	9774517	23722366	2.42696	COG0317	T	Guanosine polyphosphate pyrophosphohydrolases/synthetas	K01139	CELLULAR PROCESSES AND SIGNALING
PA3736	hom	homoserine dehydrogenase	9774517	23722366	2.42696	COG0460	E	Homoserine dehydrogenase	K12525	METABOLISM
PA3736	hom	homoserine dehydrogenase	9774517	23722366	2.42696	COG0460	E	Homoserine dehydrogenase	K00003	METABOLISM
PA3736	hom	homoserine dehydrogenase	9774517	23722366	2.42696	COG0317	T	Guanosine polyphosphate pyrophosphohydrolases/synthetas	K00951	CELLULAR PROCESSES AND SIGNALING
PA3736	hom	homoserine dehydrogenase	9774517	23722366	2.42696	COG0317	K	Guanosine polyphosphate pyrophosphohydrolases/synthetas	K00951	INFORMATION STORAGE AND PROCESSING
PA3736	hom	homoserine dehydrogenase	9774517	23722366	2.42696	COG0460	E	Homoserine dehydrogenase	K12524	METABOLISM
PA3742	rplS	50S ribosomal protein L19	0	36603930	1000	COG0335	J	Ribosomal protein L19	K02884	INFORMATION STORAGE AND PROCESSING
PA3745	rpsP	30S ribosomal protein S16	7.63E+08	1.18E+09	1.548114	COG0228	J	Ribosomal protein S16	K02959	INFORMATION STORAGE AND PROCESSING
PA3746	ffh	signal recognition particle protein Ffh	1.1E+09	4.23E+08	0.383312	COG0541	U	Signal recognition particle GTPase	K03106	CELLULAR PROCESSES AND SIGNALING
PA3753		conserved hypothetical protein	6.33E+08	5.44E+08	0.859274	COG0663	R	"Carbonic anhydrases/acetyltransferases, isoleucine patch su	K08279	POORLY CHARACTERIZED
PA3765		hypothetical protein	52010787	0	0	COG3644	S	Uncharacterized protein conserved in bacteria	K09965	POORLY CHARACTERIZED
PA3770	guaB	inosine-5'-monophosphate dehydrogenase	0	3.52E+08	1000	COG2070	R	Dioxygenases related to 2-nitropropane dioxygenase	K02371	POORLY CHARACTERIZED
PA3770	guaB	inosine-5'-monophosphate dehydrogenase	0	3.52E+08	1000	COG0517	R	FOG: CBS domain	K00088	POORLY CHARACTERIZED
PA3770	guaB	inosine-5'-monophosphate dehydrogenase	0	3.52E+08	1000	COG0516	F	IMP dehydrogenase/GMP reductase	K00364	METABOLISM
PA3770	guaB	inosine-5'-monophosphate dehydrogenase	0	3.52E+08	1000	COG2070	R	Dioxygenases related to 2-nitropropane dioxygenase	K00459	POORLY CHARACTERIZED
PA3770	guaB	inosine-5'-monophosphate dehydrogenase	0	3.52E+08	1000	COG0516	F	IMP dehydrogenase/GMP reductase	K00088	METABOLISM
PA3770	guaB	inosine-5'-monophosphate dehydrogenase	0	3.52E+08	1000	COG0517	R	FOG: CBS domain	K06041	POORLY CHARACTERIZED
PA3785		conserved hypothetical protein	3.26E+09	4.64E+09	1.421367	COG2847	S	Uncharacterized protein conserved in bacteria	K09796	POORLY CHARACTERIZED
PA3790	oprC	Putative copper transport outer membrane porin OprC precursor	4.05E+08	5.1E+08	1.257714	COG1629	P	"Outer membrane receptor proteins, mostly Fe transport"	K02014	METABOLISM
PA3790	oprC	Putative copper transport outer membrane porin OprC precursor	4.05E+08	5.1E+08	1.257714	COG4774	P	Outer membrane receptor for monomeric catechols	K02014	METABOLISM
PA3801		conserved hypothetical protein	1.08E+09	1.49E+09	1.381999					
PA3803	gcpE	probable isoprenoid biosynthetic protein GcpE	0	52338495	1000	COG0821	I	Enzyme involved in the deoxyxylulose pathway of isoprenoid I	K03526	METABOLISM
PA3804		hypothetical protein	1.78E+09	2.01E+08	0.113134					
PA3805	pilF	type 4 fimbrial biogenesis protein PilF	3.07E+08	1.71E+08	0.557028	COG3063	U	Tfp pilus assembly protein PilF	K02656	CELLULAR PROCESSES AND SIGNALING
PA3805	pilF	type 4 fimbrial biogenesis protein PilF	3.07E+08	1.71E+08	0.557028	COG3063	N	Tfp pilus assembly protein PilF	K02656	CELLULAR PROCESSES AND SIGNALING
PA3807	ndk	nucleoside diphosphate kinase	1.1E+10	1.11E+10	1.009651	COG0105	F	Nucleoside diphosphate kinase	K00940	METABOLISM
PA3809	fdx2	ferredoxin [2Fe-2S]	1.27E+08	1.34E+08	1.049646	COG0633	C	Ferredoxin	K11107	METABOLISM
PA3809	fdx2	ferredoxin [2Fe-2S]	1.27E+08	1.34E+08	1.049646	COG0633	C	Ferredoxin	K04755	METABOLISM
PA3809	fdx2	ferredoxin [2Fe-2S]	1.27E+08	1.34E+08	1.049646	COG0633	C	Ferredoxin	K02639	METABOLISM
PA3812	iscA	probable iron-binding protein IscA	55582902	0	0	COG0316	S	Uncharacterized conserved protein	K07400	POORLY CHARACTERIZED
PA3812	iscA	probable iron-binding protein IscA	55582902	0	0	COG0316	S	Uncharacterized conserved protein	K13628	POORLY CHARACTERIZED
PA3813	iscU	probable iron-binding protein IscU	3.03E+09	2.19E+09	0.72219	COG0822	C	NifU homolog involved in Fe-S cluster formation	K13819	METABOLISM
PA3813	iscU	probable iron-binding protein IscU	3.03E+09	2.19E+09	0.72219	COG0822	C	NifU homolog involved in Fe-S cluster formation	K04488	METABOLISM
PA3814	iscS	L-cysteine desulfurase (pyridoxal phosphate-dependent)	0	42560153	1000	COG1104	E	Cysteine sulfinate desulfinase/cysteine desulfurase and relate	K04487	METABOLISM
PA3816	cysE	O-acetylserine synthase	4.35E+08	82499158	0.189766	COG1045	E	Serine acetyltransferase	K00640	METABOLISM
PA3819		conserved hypothetical protein	17836174	29740166	1.667407	COG3134	S	Predicted outer membrane lipoprotein	K06077	POORLY CHARACTERIZED
PA3821	secD	secretion protein SecD	11313275	0	0	COG0342	U	Preprotein translocase subunit SecD	K03072	CELLULAR PROCESSES AND SIGNALING
PA3821	secD	secretion protein SecD	11313275	0	0	COG0342	U	Preprotein translocase subunit SecD	K12257	CELLULAR PROCESSES AND SIGNALING
PA3822		conserved hypothetical protein	1.77E+08	2.92E+08	1.649228	COG1862	U	Preprotein translocase subunit YajC	K03210	CELLULAR PROCESSES AND SIGNALING
PA3831	pepA	leucine aminopeptidase	12598658	0	0	COG0260	E	Leucyl aminopeptidase	K01255	METABOLISM
PA3831	pepA	leucine aminopeptidase	12598658	0	0	COG0260	E	Leucyl aminopeptidase	K07751	METABOLISM
PA3834	valS	valyl-tRNA synthetase	50132049	74806745	1.492194	COG0525	J	Valyl-tRNA synthetase	K01873	INFORMATION STORAGE AND PROCESSING
PA3836		hypothetical protein	3.01E+08	3.4E+08	1.130446	COG2984	R	"ABC-type uncharacterized transport system, periplasmic com	K01989	POORLY CHARACTERIZED
PA3842	spcS	"specific Pseudomonas chaperone for ExoS	0	59204411	1000					
PA3866		Pyocin S4	0	13679456	1000					
PA3901	fecA	Fe(III) dicitrate transport protein FecA	22137316	0	0	COG4772	P	Outer membrane receptor for Fe3+dicitrate	K02014	METABOLISM
PA3902		hypothetical protein	0	30684743	1000					
PA3925		probable acyl-CoA thiolase	0	24286794	1000	COG0183	I	Acetyl-CoA acetyltransferase	K00632	METABOLISM
PA3925		probable acyl-CoA thiolase	0	24286794	1000	COG0183	I	Acetyl-CoA acetyltransferase	K00626	METABOLISM
PA3931		conserved hypothetical protein	2.68E+08	19985612	7.46E-02	COG1464	P	"ABC-type metal ion transport system, periplasmic componen	K02073	METABOLISM
PA3940		probable DNA binding protein	1.93E+10	1.3E+10	0.674546	COG0776	L	Bacterial nucleoid DNA-binding protein	K05788	INFORMATION STORAGE AND PROCESSING
PA3940		probable DNA binding protein	1.93E+10	1.3E+10	0.674546	COG0776	L	Bacterial nucleoid DNA-binding protein	K05787	INFORMATION STORAGE AND PROCESSING
PA3940		probable DNA binding protein	1.93E+10	1.3E+10	0.674546	COG0776	L	Bacterial nucleoid DNA-binding protein	K03530	INFORMATION STORAGE AND PROCESSING
PA3940		probable DNA binding protein	1.93E+10	1.3E+10	0.674546	COG0776	L	Bacterial nucleoid DNA-binding protein	K04764	INFORMATION STORAGE AND PROCESSING
PA3942	tesB	acyl-CoA thioesterase II	0	71840527	1000	COG1946	I	Acyl-CoA thioesterase	K10805	METABOLISM
PA3975	thiD	phosphomethylpyrimidine kinase	45393420	38710634	0.852781	COG0351	H	Hydroxymethylpyrimidine/phosphomethylpyrimidine kinase	K00941	METABOLISM
PA3975	thiD	phosphomethylpyrimidine kinase	45393420	38710634	0.852781	COG0351	H	Hydroxymethylpyrimidine/phosphomethylpyrimidine kinase	K14153	METABOLISM
PA3975	thiD	phosphomethylpyrimidine kinase	45393420	38710634	0.852781	COG0351	H	Hydroxymethylpyrimidine/phosphomethylpyrimidine kinase	K00868	METABOLISM
PA3988	lptE	LPS-assembly lipoprotein LptE	5.76E+09	5.06E+09	0.877938	COG2980	M	Rare lipoprotein B	K03643	CELLULAR PROCESSES AND SIGNALING
PA4005		conserved hypothetical protein	7.29E+08	5.62E+08	0.770763	COG0799	S	Uncharacterized homolog of plant Iojap protein	K09710	POORLY CHARACTERIZED
PA4007	proA	gamma-glutamyl phosphate reductase	11903772	34676084	2.913033	COG0014	E	Gamma-glutamyl phosphate reductase	K00147	METABOLISM
PA4017		conserved hypothetical protein	12447794	0	0	COG0702	G	Predicted nucleoside-diphosphate-sugar epimerases	K00329	METABOLISM
PA4017		conserved hypothetical protein	12447794	0	0	COG0702	G	Predicted nucleoside-diphosphate-sugar epimerases	K00356	METABOLISM
PA4017		conserved hypothetical protein	12447794	0	0	COG0702	M	Predicted nucleoside-diphosphate-sugar epimerases	K00329	CELLULAR PROCESSES AND SIGNALING
PA4017		conserved hypothetical protein	12447794	0	0	COG0702	M	Predicted nucleoside-diphosphate-sugar epimerases	K00356	CELLULAR PROCESSES AND SIGNALING
PA4031	ppa	inorganic pyrophosphatase	7.7E+09	6.36E+09	0.825814	COG0221	C	Inorganic pyrophosphatase	K01507	METABOLISM
PA4042	xseB	exodeoxyribonuclease VII small subunit	2.3E+08	1.79E+08	0.778075	COG1722	L	Exonuclease VII small subunit	K03602	INFORMATION STORAGE AND PROCESSING
PA4052	nusB	NusB protein	2.18E+08	1.88E+08	0.863148	COG0781	K	Transcription termination factor	K03625	INFORMATION STORAGE AND PROCESSING
PA4054	ribB	"GTP cyclohydrolase II / 3	0	1.9E+08	1000	COG0108	H	"3,4-dihydroxy-2-butanone 4-phosphate synthase"	K01497	METABOLISM
PA4054	ribB	"GTP cyclohydrolase II / 3	0	1.9E+08	1000	COG0807	H	GTP cyclohydrolase II	K02858	METABOLISM
PA4054	ribB	"GTP cyclohydrolase II / 3	0	1.9E+08	1000	COG0807	H	GTP cyclohydrolase II	K01497	METABOLISM
PA4054	ribB	"GTP cyclohydrolase II / 3	0	1.9E+08	1000	COG0108	H	"3,4-dihydroxy-2-butanone 4-phosphate synthase"	K02858	METABOLISM
PA4057	nrdR	NrdR	70903456	0	0	COG1327	K	"Predicted transcriptional regulator, consists of a Zn-ribbon ar	K07738	INFORMATION STORAGE AND PROCESSING
PA4061		probable thioredoxin	0	62874726	1000	COG3118	O	Thioredoxin domain-containing protein	K05838	CELLULAR PROCESSES AND SIGNALING
PA4063		hypothetical protein	2.15E+08	1.59E+08	0.737773					
PA4067	oprG	Outer membrane protein OprG precursor	3.5E+09	5.03E+09	1.438535	COG3047	M	Outer membrane protein W	K07275	CELLULAR PROCESSES AND SIGNALING
PA4076		hypothetical protein	9222005	0	0					

PA4097		probable alcohol dehydrogenase (Zn-dependent)	0	14890980	1000	COG1063	E	Threonine dehydrogenase and related Zn-dependent dehydro	K00060	METABOLISM
PA4097		probable alcohol dehydrogenase (Zn-dependent)	0	14890980	1000	COG1063	E	Threonine dehydrogenase and related Zn-dependent dehydro	K00001	METABOLISM
PA4097		probable alcohol dehydrogenase (Zn-dependent)	0	14890980	1000	COG1063	R	Threonine dehydrogenase and related Zn-dependent dehydro	K00001	POORLY CHARACTERIZED
PA4097		probable alcohol dehydrogenase (Zn-dependent)	0	14890980	1000	COG1063	R	Threonine dehydrogenase and related Zn-dependent dehydro	K00008	POORLY CHARACTERIZED
PA4097		probable alcohol dehydrogenase (Zn-dependent)	0	14890980	1000	COG1063	R	Threonine dehydrogenase and related Zn-dependent dehydro	K00060	POORLY CHARACTERIZED
PA4097		probable alcohol dehydrogenase (Zn-dependent)	0	14890980	1000	COG1063	E	Threonine dehydrogenase and related Zn-dependent dehydro	K00008	METABOLISM
PA4135		probable transcriptional regulator	28594792	0	0	COG1846	K	Transcriptional regulators	K03712	INFORMATION STORAGE AND PROCESSING
PA4139		hypothetical protein	0	2.26E+08	1000					
PA4164		hypothetical protein	5824948	0	0					
PA4175	piv	protease IV	1.98E+08	6.35E+08	3.20808					
PA4176	ppiC2	peptidyl-prolyl cis-trans isomerase C2	1.99E+09	1.34E+09	0.673936	COG0760	O	Parvulin-like peptidyl-prolyl isomerase	K03771	CELLULAR PROCESSES AND SIGNALING
PA4176	ppiC2	peptidyl-prolyl cis-trans isomerase C2	1.99E+09	1.34E+09	0.673936	COG0760	O	Parvulin-like peptidyl-prolyl isomerase	K07533	CELLULAR PROCESSES AND SIGNALING
PA4176	ppiC2	peptidyl-prolyl cis-trans isomerase C2	1.99E+09	1.34E+09	0.673936	COG0760	O	Parvulin-like peptidyl-prolyl isomerase	K03770	CELLULAR PROCESSES AND SIGNALING
PA4176	ppiC2	peptidyl-prolyl cis-trans isomerase C2	1.99E+09	1.34E+09	0.673936	COG0760	O	Parvulin-like peptidyl-prolyl isomerase	K03769	CELLULAR PROCESSES AND SIGNALING
PA4211	phzB1	probable phenazine biosynthesis protein	0	15591689	1000	COG3631	R	Ketosteroid isomerase-related protein	K06893	POORLY CHARACTERIZED
PA4230	pchB	salicylate biosynthesis protein PchB	8.42E+08	5.82E+08	0.691196	COG1605	E	Chorismate mutase	K04782	METABOLISM
PA4230	pchB	salicylate biosynthesis protein PchB	8.42E+08	5.82E+08	0.691196	COG1605	E	Chorismate mutase	K14170	METABOLISM
PA4230	pchB	salicylate biosynthesis protein PchB	8.42E+08	5.82E+08	0.691196	COG1605	E	Chorismate mutase	K14187	METABOLISM
PA4230	pchB	salicylate biosynthesis protein PchB	8.42E+08	5.82E+08	0.691196	COG1605	E	Chorismate mutase	K04093	METABOLISM
PA4230	pchB	salicylate biosynthesis protein PchB	8.42E+08	5.82E+08	0.691196	COG1605	E	Chorismate mutase	K01850	METABOLISM
PA4230	pchB	salicylate biosynthesis protein PchB	8.42E+08	5.82E+08	0.691196	COG1605	E	Chorismate mutase	K13853	METABOLISM
PA4230	pchB	salicylate biosynthesis protein PchB	8.42E+08	5.82E+08	0.691196	COG1605	E	Chorismate mutase	K04092	METABOLISM
PA4230	pchB	salicylate biosynthesis protein PchB	8.42E+08	5.82E+08	0.691196	COG1605	E	Chorismate mutase	K04516	METABOLISM
PA4232	ssb	single-stranded DNA-binding protein	1.57E+08	2.1E+08	1.339922	COG0629	L	Single-stranded DNA-binding protein	K03111	INFORMATION STORAGE AND PROCESSING
PA4235	ftnA	bacterial ferritin	6.71E+09	5.45E+09	0.813125	COG2193	P	Bacterioferritin (cytochrome b1)	K03594	METABOLISM
PA4236	katA	catalase	5.92E+08	2.93E+08	0.494419	COG0753	P	Catalase	K00429	METABOLISM
PA4236	katA	catalase	5.92E+08	2.93E+08	0.494419	COG0753	P	Catalase	K03781	METABOLISM
PA4237	rplQ	50S ribosomal protein L17	1.01E+10	6.02E+09	0.594527	COG0203	J	Ribosomal protein L17	K02879	INFORMATION STORAGE AND PROCESSING
PA4238	rpoA	DNA-directed RNA polymerase alpha chain	1.99E+09	4.13E+09	2.074744	COG0202	K	"DNA-directed RNA polymerase, alpha subunit/40 kD subunit"	K03047	INFORMATION STORAGE AND PROCESSING
PA4238	rpoA	DNA-directed RNA polymerase alpha chain	1.99E+09	4.13E+09	2.074744	COG0202	K	"DNA-directed RNA polymerase, alpha subunit/40 kD subunit"	K03040	INFORMATION STORAGE AND PROCESSING
PA4239	rpsD	30S ribosomal protein S4	48572108	2.92E+08	6.021009	COG0522	J	Ribosomal protein S4 and related proteins	K02986	INFORMATION STORAGE AND PROCESSING
PA4240	rpsK	30S ribosomal protein S11	1.76E+08	7.23E+08	4.102362	COG0100	J	Ribosomal protein S11	K02948	INFORMATION STORAGE AND PROCESSING
PA4244	rplO	50S ribosomal protein L15	3.89E+09	5.1E+09	1.312233	COG0200	J	Ribosomal protein L15	K02876	INFORMATION STORAGE AND PROCESSING
PA4245	rpmD	50S ribosomal protein L30	91279203	5.27E+08	5.769197	COG1841	J	Ribosomal protein L30/L7E	K02937	INFORMATION STORAGE AND PROCESSING
PA4245	rpmD	50S ribosomal protein L30	91279203	5.27E+08	5.769197	COG1841	J	Ribosomal protein L30/L7E	K02907	INFORMATION STORAGE AND PROCESSING
PA4246	rpsE	30S ribosomal protein S5	4.54E+08	3.07E+09	6.750367	COG0098	J	Ribosomal protein S5	K02981	INFORMATION STORAGE AND PROCESSING
PA4246	rpsE	30S ribosomal protein S5	4.54E+08	3.07E+09	6.750367	COG0098	J	Ribosomal protein S5	K02988	INFORMATION STORAGE AND PROCESSING
PA4247	rplR	50S ribosomal protein L18	2.61E+08	8.49E+08	3.253879	COG0256	J	Ribosomal protein L18	K02881	INFORMATION STORAGE AND PROCESSING
PA4248	rplF	50S ribosomal protein L6	3.36E+08	1.08E+09	3.213564	COG0097	J	Ribosomal protein L6P/L9E	K02933	INFORMATION STORAGE AND PROCESSING
PA4248	rplF	50S ribosomal protein L6	3.36E+08	1.08E+09	3.213564	COG0097	J	Ribosomal protein L6P/L9E	K02940	INFORMATION STORAGE AND PROCESSING
PA4249	rpsH	30S ribosomal protein S8	3.08E+09	1.75E+09	0.566008	COG0096	J	Ribosomal protein S8	K02994	INFORMATION STORAGE AND PROCESSING
PA4250	rpsN	30S ribosomal protein S14	2.6E+08	6.96E+08	2.671497	COG0199	J	Ribosomal protein S14	K02954	INFORMATION STORAGE AND PROCESSING
PA4251	rplE	50S ribosomal protein L5	3.1E+09	4E+09	1.289802	COG0094	J	Ribosomal protein L5	K02931	INFORMATION STORAGE AND PROCESSING
PA4252	rplX	50S ribosomal protein L24	8.53E+09	1.21E+10	1.422891	COG0198	J	Ribosomal protein L24	K02895	INFORMATION STORAGE AND PROCESSING
PA4254	rpsQ	30S ribosomal protein S17	91690951	4.72E+08	5.150554	COG0186	J	Ribosomal protein S17	K02961	INFORMATION STORAGE AND PROCESSING
PA4255	rpmC	50S ribosomal protein L29	2.07E+10	2.24E+10	1.083765	COG0255	J	Ribosomal protein L29	K02918	INFORMATION STORAGE AND PROCESSING
PA4255	rpmC	50S ribosomal protein L29	2.07E+10	2.24E+10	1.083765	COG0255	J	Ribosomal protein L29	K02904	INFORMATION STORAGE AND PROCESSING
PA4257	rpsC	30S ribosomal protein S3	99643482	1.7E+08	1.708558	COG0092	J	Ribosomal protein S3	K02982	INFORMATION STORAGE AND PROCESSING
PA4258	rplV	50S ribosomal protein L22	1.65E+09	2.23E+09	1.346674	COG0091	J	Ribosomal protein L22	K02890	INFORMATION STORAGE AND PROCESSING
PA4259	rpsS	30S ribosomal protein S19	4.51E+08	1.45E+09	3.209029	COG0185	J	Ribosomal protein S19	K02965	INFORMATION STORAGE AND PROCESSING
PA4260	rplB	50S ribosomal protein L2	21748358	1.53E+08	7.019821	COG0090	J	Ribosomal protein L2	K02886	INFORMATION STORAGE AND PROCESSING
PA4261	rplW	50S ribosomal protein L23	3.18E+08	1.9E+09	5.969437	COG0089	J	Ribosomal protein L23	K02892	INFORMATION STORAGE AND PROCESSING
PA4261	rplW	50S ribosomal protein L23	3.18E+08	1.9E+09	5.969437	COG0089	J	Ribosomal protein L23	K02893	INFORMATION STORAGE AND PROCESSING
PA4262	rplD	50S ribosomal protein L4	34948059	38245121	1.094342	COG0088	J	Ribosomal protein L4	K02926	INFORMATION STORAGE AND PROCESSING
PA4262	rplD	50S ribosomal protein L4	34948059	38245121	1.094342	COG0088	J	Ribosomal protein L4	K02930	INFORMATION STORAGE AND PROCESSING
PA4263	rplC	50S ribosomal protein L3	31857404	1.66E+08	5.210799	COG0087	J	Ribosomal protein L3	K02925	INFORMATION STORAGE AND PROCESSING
PA4263	rplC	50S ribosomal protein L3	31857404	1.66E+08	5.210799	COG0087	J	Ribosomal protein L3	K02906	INFORMATION STORAGE AND PROCESSING
PA4264	rpsJ	30S ribosomal protein S10	98428884	1.88E+09	19.09406	COG0051	J	Ribosomal protein S10	K02946	INFORMATION STORAGE AND PROCESSING
PA4266	fusA1	elongation factor G	3.54E+08	1.22E+09	3.449641	COG0480	J	Translation elongation factors (GTPases)	K03234	INFORMATION STORAGE AND PROCESSING
PA4266	fusA1	elongation factor G	3.54E+08	1.22E+09	3.449641	COG0480	J	Translation elongation factors (GTPases)	K02355	INFORMATION STORAGE AND PROCESSING
PA4267	rpsG	30S ribosomal protein S7	1.02E+10	8.04E+09	0.787247	COG0049	J	Ribosomal protein S7	K02992	INFORMATION STORAGE AND PROCESSING
PA4269	rpoC	DNA-directed RNA polymerase beta* chain	20455648	1.39E+08	6.807375	COG0086	K	"DNA-directed RNA polymerase, beta' subunit/160 kD subunit	K03042	INFORMATION STORAGE AND PROCESSING
PA4269	rpoC	DNA-directed RNA polymerase beta* chain	20455648	1.39E+08	6.807375	COG0086	K	"DNA-directed RNA polymerase, beta' subunit/160 kD subunit	K03046	INFORMATION STORAGE AND PROCESSING
PA4269	rpoC	DNA-directed RNA polymerase beta* chain	20455648	1.39E+08	6.807375	COG0086	K	"DNA-directed RNA polymerase, beta' subunit/160 kD subunit	K03041	INFORMATION STORAGE AND PROCESSING
PA4270	rpoB	DNA-directed RNA polymerase beta chain	10712322	1.41E+08	13.15328	COG0085	K	"DNA-directed RNA polymerase, beta subunit/140 kD subunit	K03043	INFORMATION STORAGE AND PROCESSING
PA4271	rplL	50S ribosomal protein L7 / L12	3.74E+10	7.44E+10	1.991144	COG0222	J	Ribosomal protein L7/L12	K02935	INFORMATION STORAGE AND PROCESSING
PA4272	rplJ	50S ribosomal protein L10	4.82E+09	5.1E+09	1.058794	COG0244	J	Ribosomal protein L10	K02864	INFORMATION STORAGE AND PROCESSING
PA4273	rplA	50S ribosomal protein L1	4.97E+09	5.2E+09	1.045926	COG0081	J	Ribosomal protein L1	K02863	INFORMATION STORAGE AND PROCESSING
PA4274	rplK	50S ribosomal protein L11	2.27E+08	1.01E+09	4.446979	COG0080	J	Ribosomal protein L11	K02867	INFORMATION STORAGE AND PROCESSING
PA4275	nusG	transcription antitermination protein NusG	15254149	0	0	COG0250	K	Transcription antiterminator	K02601	INFORMATION STORAGE AND PROCESSING
PA4275	nusG	transcription antitermination protein NusG	15254149	0	0	COG0250	K	Transcription antiterminator	K05785	INFORMATION STORAGE AND PROCESSING
PA4277	tufB	elongation factor Tu	6.97E+09	2.41E+10	3.458687	COG0050	J	GTPases - translation elongation factors	K02358	INFORMATION STORAGE AND PROCESSING
PA4307	pctC	chemotactic transducer PctC	1.03E+08	1.1E+09	10.70025	COG0840	T	Methyl-accepting chemotaxis protein	K03406	CELLULAR PROCESSES AND SIGNALING
PA4307	pctC	chemotactic transducer PctC	1.03E+08	1.1E+09	10.70025	COG0840	N	Methyl-accepting chemotaxis protein	K03406	CELLULAR PROCESSES AND SIGNALING
PA4309	pctA	chemotactic transducer PctA	1.38E+08	1.22E+09	8.88316	COG0840	N	Methyl-accepting chemotaxis protein	K03406	CELLULAR PROCESSES AND SIGNALING
PA4309	pctA	chemotactic transducer PctA	1.38E+08	1.22E+09	8.88316	COG0840	T	Methyl-accepting chemotaxis protein	K03406	CELLULAR PROCESSES AND SIGNALING
PA4310	pctB	chemotactic transducer PctB	1.03E+08	1.07E+09	10.37231	COG0840	N	Methyl-accepting chemotaxis protein	K03406	CELLULAR PROCESSES AND SIGNALING
PA4310	pctB	chemotactic transducer PctB	1.03E+08	1.07E+09	10.37231	COG0840	T	Methyl-accepting chemotaxis protein	K03406	CELLULAR PROCESSES AND SIGNALING
PA4315	mvaT	"transcriptional regulator MvaT	4.9E+10	2.09E+10	0.426297					
PA4318		hypothetical protein	0	57286156	1000					
PA4329	pykA	pyruvate kinase II	35114831	50668548	1.442939	COG0469	G	Pyruvate kinase	K00873	METABOLISM
PA4333		probable fumarase	49442883	1.69E+08	3.412611	COG1838	C	"Tartrate dehydratase beta subunit/Fumarate hydratase class	K01678	METABOLISM
PA4333		probable fumarase	49442883	1.69E+08	3.412611	COG1838	C	"Tartrate dehydratase beta subunit/Fumarate hydratase class	K01676	METABOLISM
PA4333		probable fumarase	49442883	1.69E+08	3.412611	COG1838	C	"Tartrate dehydratase beta subunit/Fumarate hydratase class	K03780	METABOLISM
PA4333		probable fumarase	49442883	1.69E+08	3.412611	COG1951	C	"Tartrate dehydratase alpha subunit/Fumarate hydratase clas	K01677	METABOLISM
PA4333		probable fumarase	49442883	1.69E+08	3.412611	COG1951	C	"Tartrate dehydratase alpha subunit/Fumarate hydratase clas	K01676	METABOLISM
PA4333		probable fumarase	49442883	1.69E+08	3.412611	COG1951	C	"Tartrate dehydratase alpha subunit/Fumarate hydratase clas	K03779	METABOLISM
PA4336		conserved hypothetical protein	4.01E+08	3.78E+08	0.942669	COG0693	R	Putative intracellular protease/amidase	K05520	POORLY CHARACTERIZED
PA4336		conserved hypothetical protein	4.01E+08	3.78E+08	0.942669	COG0693	R	Putative intracellular protease/amidase	K03152	POORLY CHARACTERIZED
PA4349		hypothetical protein	0	1.79E+08	1000	COG1960	I	Acyl-CoA dehydrogenases	K00257	METABOLISM
PA4349		hypothetical protein	0	1.79E+08	1000	COG1960	I	Acyl-CoA dehydrogenases	K00249	METABOLISM
PA4352		conserved hypothetical protein	1.3E+09	9.63E+08	0.740998	COG0589	T	Universal stress protein UspA and related nucleotide-binding	K06149	CELLULAR PROCESSES AND SIGNALING
PA4366	sodB	superoxide dismutase	1.96E+09	1.52E+09	0.775348	COG0605	P	Superoxide dismutase	K04564	METABOLISM
PA4369		hypothetical protein	1.21E+09	63539121	5.27E-02	COG1704	S	Uncharacterized conserved protein	K03744	POORLY CHARACTERIZED
PA4370	icmP	Insulin-cleaving metalloproteinase outer membrane protein prec	4.23E+10	4.78E+10	1.129842	COG3487	P	Uncharacterized iron-regulated protein	K07231	METABOLISM
PA4372		hypothetical protein	5.14E+08	1.57E+08	0.304663	COG3489	R	Predicted periplasmic lipoprotein	K07338	POORLY CHARACTERIZED
PA4385	groEL	GroEL protein	2.35E+10	2.46E+10	1.047743	COG0459	O	Chaperonin GroEL (HSP60 family)	K04077	CELLULAR PROCESSES AND SIGNALING
PA4386	groES	GroES protein	3.61E+10	3.44E+10	0.953209	COG0234	O	Co-chaperonin GroES (HSP10)	K04078	CELLULAR PROCESSES AND SIGNALING
PA4389		3-oxacyl-acyl carrier protein reductase	0	28671984	1000	COG1028	I	Dehydrogenases with different specificities (related to short-c	K00059	METABOLISM
PA4389		3-oxacyl-acyl carrier protein reductase	0	28671984	1000	COG1028	Q	Dehydrogenases with different specificities (related to short-c	K00059	METABOLISM
PA4389		3-oxacyl-acyl carrier protein reductase	0	28671984	1000	COG1028	R	Dehydrogenases with different specificities (related to short-c	K00059	POORLY CHARACTERIZED
PA4394		conserved hypothetical protein	0	48024817	1000	COG0668	M	Small-conductance mechanosensitive channel	K03442	CELLULAR PROCESSES AND SIGNALING
PA4395		conserved hypothetical protein	5.39E+09	1.43E+09	0.264478	COG1666	S	Uncharacterized protein conserved in bacteria	K09767	POORLY CHARACTERIZED
PA4399		conserved hypothetical protein	1.65E+08	0	0	COG2096	S	Uncharacterized conserved protein	K00798	POORLY CHARACTERIZED
PA4402	argJ	glutamate N-acetyltransferase	25028576	0	0	COG1364	E	N-acetylglutamate synthase (N-acetylornithine aminotransfer	K00620	METABOLISM
PA4403	secA	secretion protein SecA	2.06E+08	5.93E+08	2.886603	COG0653	U	"Preprotein translocase subunit SecA (ATPase, RNA helicase)"	K03070	CELLULAR PROCESSES AND SIGNALING
PA4406	lpxC	UDP-3-O-acyl-N-acetylglucosamine deacetylase	4.77E+08	4.46E+08	0.933415	COG0774	M	UDP-3-O-acyl-N-acetylglucosamine deacetylase	K02535	CELLULAR PROCESSES AND SIGNALING
PA4407	ftsZ	cell division protein FtsZ	9.46E+08	4.5E+08	0.47604	COG0206	D	Cell division GTPase	K03531	CELLULAR PROCESSES AND SIGNALING
PA4408	ftsA	cell division protein FtsA	68051520	0	0	COG0849	D	Actin-like ATPase involved in cell division	K03590	CELLULAR PROCESSES AND SIGNALING
PA4409	ftsQ	cell division protein FtsQ	4.06E+08	0	0	COG1589	M	Cell division septal protein	K03589	CELLULAR PROCESSES AND SIGNALING
PA4417	murE	"UDP-N-acetylmuramoylalanyl-D-glutamate-2	0	28333087	1000	COG0769	M	UDP-N-acetylmuramyl tripeptide synthase	K01928	CELLULAR PROCESSES AND SIGNALING
PA4417	murE	"UDP-N-acetylmuramoylalanyl-D-glutamate-2	0	28333087	1000	COG0769	M	UDP-N-acetylmuramyl tripeptide synthase	K05362	CELLULAR PROCESSES AND SIGNALING
PA4417	murE	"UDP-N-acetylmuramoylalanyl-D-glutamate-2	0	28333087	1000	COG0769	M	UDP-N-acetylmuramyl tripeptide synthase	K03802	CELLULAR PROCESSES AND SIGNALING
PA4420		conserved hypothetical protein	29489340	0	0	COG0275	M	Predicted S-adenosylmethionine-dependent methyltransferas	K03438	CELLULAR PROCESSES AND SIGNALING
PA4423		conserved hypothetical protein	1.92E+09	2.2E+09	1.14629	COG3107	R	Putative lipoprotein	K07121	POORLY CHARACTERIZED
PA4426		conserved hypothetical protein	26025011	68184018	2.619942	COG2823	R	Predicted periplasmic or secreted lipoprotein	K04065	POORLY CHARACTERIZED
PA4429		probable cytochrome c1 precursor	2.91E+09	1.4E+09	0.4814	COG2857	C	Cytochrome c1	K02275	METABOLISM
PA4429		probable cytochrome c1 precursor	2.91E+09	1.4E+09	0.4814	COG2857	C	Cytochrome c1	K00413	METABOLISM
PA4431		probable iron-sulfur protein	3.17E+09	2.29E+09	0.722797	COG0723	C	Rieske Fe-S protein	K03890	METABOLISM
PA4431		probable iron-sulfur protein	3.17E+09	2.29E+09	0.722797	COG0723	C	Rieske Fe-S protein	K02636	METABOLISM
PA4431		probable iron-sulfur protein	3.17E+09	2.29E+09	0.722797	COG0723	C	Rieske Fe-S protein	K00411	METABOLISM

PA4432	rpsI	30S ribosomal protein S9	0	24530842	1000	COG0103	J	Ribosomal protein S9	K02996	INFORMATION STORAGE AND PROCESSING
PA4433	rplM	50S ribosomal protein L13	1.02E+09	3.39E+09	3.314908	COG0102	J	Ribosomal protein L13	K02871	INFORMATION STORAGE AND PROCESSING
PA4438		conserved hypothetical protein	31472200	0	0	COG1485	R	Predicted ATPase	K06916	POORLY CHARACTERIZED
PA4439	trpS	tryptophanyl-tRNA synthetase	17603443	48921066	2.779062	COG0180	J	Tryptophanyl-tRNA synthetase	K01867	INFORMATION STORAGE AND PROCESSING
PA4441		hypothetical protein	6.14E+09	6.44E+09	1.047534	COG3105	S	Uncharacterized protein conserved in bacteria	K09908	POORLY CHARACTERIZED
PA4454		conserved hypothetical protein	0	36505618	1000	COG1463	Q	"ABC-type transport system involved in resistance to organic s	K02067	METABOLISM
PA4457		arabinose-5-phosphate isomerase KdsD	27242201	47403781	1.740086	COG0794	M	Predicted sugar phosphate isomerase involved in capsule forn	K02467	CELLULAR PROCESSES AND SIGNALING
PA4457		arabinose-5-phosphate isomerase KdsD	27242201	47403781	1.740086	COG0517	R	FOG: CBS domain	K00088	POORLY CHARACTERIZED
PA4457		arabinose-5-phosphate isomerase KdsD	27242201	47403781	1.740086	COG0794	M	Predicted sugar phosphate isomerase involved in capsule forn	K06041	CELLULAR PROCESSES AND SIGNALING
PA4457		arabinose-5-phosphate isomerase KdsD	27242201	47403781	1.740086	COG0794	M	Predicted sugar phosphate isomerase involved in capsule forn	K08094	CELLULAR PROCESSES AND SIGNALING
PA4457		arabinose-5-phosphate isomerase KdsD	27242201	47403781	1.740086	COG0517	R	FOG: CBS domain	K06041	POORLY CHARACTERIZED
PA4459	lptC	Lipopolysaccharide export system protein LptC	7.69E+08	3E+08	0.390399	COG3117	S	Uncharacterized protein conserved in bacteria	K11719	POORLY CHARACTERIZED
PA4460	lptH	LptH	1.18E+08	2.42E+08	2.038829	COG1934	S	Uncharacterized protein conserved in bacteria	K09774	POORLY CHARACTERIZED
PA4463		conserved hypothetical protein	1.2E+08	15133101	0.125771	COG1544	J	Ribosome-associated protein Y (PSrp-1)	K05808	INFORMATION STORAGE AND PROCESSING
PA4463		conserved hypothetical protein	1.2E+08	15133101	0.125771	COG1544	J	Ribosome-associated protein Y (PSrp-1)	K05809	INFORMATION STORAGE AND PROCESSING
PA4464	ptsN	nitrogen regulatory IIA protein	3.56E+08	2.09E+08	0.586104	COG1762	T	Phosphotransferase system mannitol/fructose-specific IIA dor	K02770	CELLULAR PROCESSES AND SIGNALING
PA4464	ptsN	nitrogen regulatory IIA protein	3.56E+08	2.09E+08	0.586104	COG1762	G	Phosphotransferase system mannitol/fructose-specific IIA dor	K02821	METABOLISM
PA4464	ptsN	nitrogen regulatory IIA protein	3.56E+08	2.09E+08	0.586104	COG1762	T	Phosphotransferase system mannitol/fructose-specific IIA dor	K02821	CELLULAR PROCESSES AND SIGNALING
PA4464	ptsN	nitrogen regulatory IIA protein	3.56E+08	2.09E+08	0.586104	COG1762	G	Phosphotransferase system mannitol/fructose-specific IIA dor	K02806	METABOLISM
PA4464	ptsN	nitrogen regulatory IIA protein	3.56E+08	2.09E+08	0.586104	COG1762	G	Phosphotransferase system mannitol/fructose-specific IIA dor	K02770	METABOLISM
PA4464	ptsN	nitrogen regulatory IIA protein	3.56E+08	2.09E+08	0.586104	COG1762	G	Phosphotransferase system mannitol/fructose-specific IIA dor	K02769	METABOLISM
PA4464	ptsN	nitrogen regulatory IIA protein	3.56E+08	2.09E+08	0.586104	COG1762	G	Phosphotransferase system mannitol/fructose-specific IIA dor	K02773	METABOLISM
PA4464	ptsN	nitrogen regulatory IIA protein	3.56E+08	2.09E+08	0.586104	COG1762	T	Phosphotransferase system mannitol/fructose-specific IIA dor	K02768	CELLULAR PROCESSES AND SIGNALING
PA4464	ptsN	nitrogen regulatory IIA protein	3.56E+08	2.09E+08	0.586104	COG1762	G	Phosphotransferase system mannitol/fructose-specific IIA dor	K02768	METABOLISM
PA4464	ptsN	nitrogen regulatory IIA protein	3.56E+08	2.09E+08	0.586104	COG1762	T	Phosphotransferase system mannitol/fructose-specific IIA dor	K02769	CELLULAR PROCESSES AND SIGNALING
PA4464	ptsN	nitrogen regulatory IIA protein	3.56E+08	2.09E+08	0.586104	COG1762	T	Phosphotransferase system mannitol/fructose-specific IIA dor	K02806	CELLULAR PROCESSES AND SIGNALING
PA4464	ptsN	nitrogen regulatory IIA protein	3.56E+08	2.09E+08	0.586104	COG1762	T	Phosphotransferase system mannitol/fructose-specific IIA dor	K02773	CELLULAR PROCESSES AND SIGNALING
PA4468	sodM	superoxide dismutase	0	48967286	1000	COG0605	P	Superoxide dismutase	K04564	METABOLISM
PA4470	fumC1	fumarate hydratase	70276584	6.09E+08	8.664241	COG0114	C	Fumarase	K01679	METABOLISM
PA4473		hypothetical protein	2.35E+09	2.99E+08	0.12748	COG3028	S	Uncharacterized protein conserved in bacteria	K09889	POORLY CHARACTERIZED
PA4473		hypothetical protein	2.35E+09	2.99E+08	0.12748	COG3028	S	Uncharacterized protein conserved in bacteria	K09889	POORLY CHARACTERIZED
PA4473		hypothetical protein	2.35E+09	2.99E+08	0.12748	COG3028	S	Uncharacterized protein conserved in bacteria	K09889	POORLY CHARACTERIZED
PA4473		hypothetical protein	2.35E+09	2.99E+08	0.12748	COG3028	S	Uncharacterized protein conserved in bacteria	K09889	POORLY CHARACTERIZED
PA4480	mreC	rod shape-determining protein MreC	29619827	0	0	COG1792	M	Cell shape-determining protein	K03570	CELLULAR PROCESSES AND SIGNALING
PA4481	mreB	rod shape-determining protein MreB	79412164	1.46E+08	1.841378	COG1077	D	Actin-like ATPase involved in cell morphogenesis	K03569	CELLULAR PROCESSES AND SIGNALING
PA4482	gatC	Glu-tRNA(Gln) amidotransferase subunit C	2.54E+09	2.1E+09	0.825038	COG0721	J	Asp-tRNAAsn/Glu-tRNAGln amidotransferase C subunit	K02435	INFORMATION STORAGE AND PROCESSING
PA4483	gatA	Glu-tRNA(Gln) amidotransferase subunit A	11349115	0	0	COG0154	J	Asp-tRNAAsn/Glu-tRNAGln amidotransferase A subunit and re	K01426	INFORMATION STORAGE AND PROCESSING
PA4483	gatA	Glu-tRNA(Gln) amidotransferase subunit A	11349115	0	0	COG0154	J	Asp-tRNAAsn/Glu-tRNAGln amidotransferase A subunit and re	K02433	INFORMATION STORAGE AND PROCESSING
PA4484	gatB	Glu-tRNA(Gln) amidotransferase subunit B	1.06E+08	5.46E+08	5.169849	COG0064	J	Asp-tRNAAsn/Glu-tRNAGln amidotransferase B subunit (PET1	K02434	INFORMATION STORAGE AND PROCESSING
PA4493	roxR	RoxR	20543592	0	0					
PA4495		hypothetical protein	3.16E+09	2.35E+09	0.742015					
PA4520		probable chemotaxis transducer	0	6.58E+08	1000	COG0840	T	Methyl-accepting chemotaxis protein	K03406	CELLULAR PROCESSES AND SIGNALING
PA4520		probable chemotaxis transducer	0	6.58E+08	1000	COG0840	N	Methyl-accepting chemotaxis protein	K03406	CELLULAR PROCESSES AND SIGNALING
PA4524	nadC	nicotinate-nucleotide pyrophosphorylase	11353851	0	0	COG0157	H	Nicotinate-nucleotide pyrophosphorylase	K00767	METABOLISM
PA4524	nadC	nicotinate-nucleotide pyrophosphorylase	11353851	0	0	COG0157	H	Nicotinate-nucleotide pyrophosphorylase	K03813	METABOLISM
PA4525	pilA	type 4 fimbrial precursor PilA	8.12E+09	9.85E+09	1.213607	COG4969	N	"Tfp pilus assembly protein, major pilin PilA"	K02682	CELLULAR PROCESSES AND SIGNALING
PA4525	pilA	type 4 fimbrial precursor PilA	8.12E+09	9.85E+09	1.213607	COG4969	U	"Tfp pilus assembly protein, major pilin PilA"	K02655	CELLULAR PROCESSES AND SIGNALING
PA4525	pilA	type 4 fimbrial precursor PilA	8.12E+09	9.85E+09	1.213607	COG4969	U	"Tfp pilus assembly protein, major pilin PilA"	K02682	CELLULAR PROCESSES AND SIGNALING
PA4525	pilA	type 4 fimbrial precursor PilA	8.12E+09	9.85E+09	1.213607	COG4969	N	"Tfp pilus assembly protein, major pilin PilA"	K02650	CELLULAR PROCESSES AND SIGNALING
PA4525	pilA	type 4 fimbrial precursor PilA	8.12E+09	9.85E+09	1.213607	COG4969	N	"Tfp pilus assembly protein, major pilin PilA"	K02655	CELLULAR PROCESSES AND SIGNALING
PA4525	pilA	type 4 fimbrial precursor PilA	8.12E+09	9.85E+09	1.213607	COG4969	U	"Tfp pilus assembly protein, major pilin PilA"	K02650	CELLULAR PROCESSES AND SIGNALING
PA4529	coaE	dephosphocoenzyme A kinase	1.4E+08	43140238	0.309054	COG0237	H	Dephospho-CoA kinase	K00859	METABOLISM
PA4530		conserved hypothetical protein	51494239	1.38E+08	2.67622	COG3024	S	Uncharacterized protein conserved in bacteria	K09862	POORLY CHARACTERIZED
PA4536		hypothetical protein	0	1.54E+08	1000					
PA4542	clpB	ClpB protein	4.51E+08	6.91E+08	1.531048	COG0542	O	"ATPases with chaperone activity, ATP-binding subunit"	K03695	CELLULAR PROCESSES AND SIGNALING
PA4542	clpB	ClpB protein	4.51E+08	6.91E+08	1.531048	COG0542	O	"ATPases with chaperone activity, ATP-binding subunit"	K11907	CELLULAR PROCESSES AND SIGNALING
PA4542	clpB	ClpB protein	4.51E+08	6.91E+08	1.531048	COG0542	O	"ATPases with chaperone activity, ATP-binding subunit"	K03696	CELLULAR PROCESSES AND SIGNALING
PA4542	clpB	ClpB protein	4.51E+08	6.91E+08	1.531048	COG0542	O	"ATPases with chaperone activity, ATP-binding subunit"	K03694	CELLULAR PROCESSES AND SIGNALING
PA4545	comL	competence protein ComL	20732083	46274173	2.232008	COG4105	R	DNA uptake lipoprotein	K05807	POORLY CHARACTERIZED
PA4555	pilY2	type 4 fimbrial biogenesis protein PilY2	1.07E+08	0	0					
PA4558		"probable peptidyl-prolyl cis-trans isomerase	7.11E+08	5.94E+08	0.83565	COG1047	O	FKBP-type peptidyl-prolyl cis-trans isomerases 2	K03774	CELLULAR PROCESSES AND SIGNALING
PA4558		"probable peptidyl-prolyl cis-trans isomerase	7.11E+08	5.94E+08	0.83565	COG1047	O	FKBP-type peptidyl-prolyl cis-trans isomerases 2	K03775	CELLULAR PROCESSES AND SIGNALING
PA4558		"probable peptidyl-prolyl cis-trans isomerase	7.11E+08	5.94E+08	0.83565	COG1047	O	FKBP-type peptidyl-prolyl cis-trans isomerases 2	K01802	CELLULAR PROCESSES AND SIGNALING
PA4563	rpsT	30S ribosomal protein S20	6.78E+09	6.13E+09	0.9034	COG0268	J	Ribosomal protein S20	K02968	INFORMATION STORAGE AND PROCESSING
PA4566	obg	GTP-binding protein Obg	45523638	0	0	COG0536	R	Predicted GTPase	K03979	POORLY CHARACTERIZED
PA4568	rplU	50S ribosomal protein L21	0	70213639	1000	COG0261	J	Ribosomal protein L21	K02888	INFORMATION STORAGE AND PROCESSING
PA4572	fklB	peptidyl-prolyl cis-trans isomerase FklB	6.49E+08	1.78E+09	2.745913	COG0545	O	FKBP-type peptidyl-prolyl cis-trans isomerases 1	K01802	CELLULAR PROCESSES AND SIGNALING
PA4572	fklB	peptidyl-prolyl cis-trans isomerase FklB	6.49E+08	1.78E+09	2.745913	COG0545	O	FKBP-type peptidyl-prolyl cis-trans isomerases 1	K03772	CELLULAR PROCESSES AND SIGNALING
PA4572	fklB	peptidyl-prolyl cis-trans isomerase FklB	6.49E+08	1.78E+09	2.745913	COG0545	O	FKBP-type peptidyl-prolyl cis-trans isomerases 1	K03773	CELLULAR PROCESSES AND SIGNALING
PA4572	fklB	peptidyl-prolyl cis-trans isomerase FklB	6.49E+08	1.78E+09	2.745913	COG0545	O	FKBP-type peptidyl-prolyl cis-trans isomerases 1	K09575	CELLULAR PROCESSES AND SIGNALING
PA4576		probable ATP-dependent protease	0	8360814	1000	COG1067	O	Predicted ATP-dependent protease	K04770	CELLULAR PROCESSES AND SIGNALING
PA4576		probable ATP-dependent protease	0	8360814	1000	COG1067	O	Predicted ATP-dependent protease	K04076	CELLULAR PROCESSES AND SIGNALING
PA4576		probable ATP-dependent protease	0	8360814	1000	COG1067	O	Predicted ATP-dependent protease	K01338	CELLULAR PROCESSES AND SIGNALING
PA4577		hypothetical protein	1.28E+08	9.77E+08	7.613762	COG1734	T	DnaK suppressor protein	K06204	CELLULAR PROCESSES AND SIGNALING
PA4587	ccpR	cytochrome c551 peroxidase precursor	1.21E+09	1.11E+08	9.15E-02	COG1858	P	Cytochrome c peroxidase	K00428	METABOLISM
PA4595		probable ATP-binding component of ABC transporter	2.67E+08	2.47E+09	9.2377	COG0488	R	ATPase components of ABC transporters with duplicated ATPa	K06158	POORLY CHARACTERIZED
PA4602	glyA3	serine hydroxymethyltransferase	0	1.1E+08	1000	COG0112	E	Glycine/serine hydroxymethyltransferase	K00600	METABOLISM
PA4611		hypothetical protein	1.11E+10	7.79E+09	0.699866	COG2841	S	Uncharacterized protein conserved in bacteria	K09794	POORLY CHARACTERIZED
PA4632		hypothetical protein	2.58E+08	16171473	6.26E-02	COG0501	O	Zn-dependent protease with chaperone function	K07387	CELLULAR PROCESSES AND SIGNALING
PA4632		hypothetical protein	2.58E+08	16171473	6.26E-02	COG0501	O	Zn-dependent protease with chaperone function	K06013	CELLULAR PROCESSES AND SIGNALING
PA4632		hypothetical protein	2.58E+08	16171473	6.26E-02	COG0501	O	Zn-dependent protease with chaperone function	K03799	CELLULAR PROCESSES AND SIGNALING
PA4633		probable chemotaxis transducer	0	7.3E+08	1000	COG0840	T	Methyl-accepting chemotaxis protein	K03406	CELLULAR PROCESSES AND SIGNALING
PA4633		probable chemotaxis transducer	0	7.3E+08	1000	COG0840	N	Methyl-accepting chemotaxis protein	K03406	CELLULAR PROCESSES AND SIGNALING
PA4639		hypothetical protein	29179569	73265823	2.51086					
PA4640	mqoB	malate:quinone oxidoreductase	30914057	72144646	2.333717	COG0579	R	Predicted dehydrogenase	K00116	POORLY CHARACTERIZED
PA4640	mqoB	malate:quinone oxidoreductase	30914057	72144646	2.333717	COG0579	R	Predicted dehydrogenase	K00111	POORLY CHARACTERIZED
PA4642		hypothetical protein	9.22E+08	9.64E+08	1.04482					
PA4646	upp	uracil phosphoribosyltransferase	40475059	39227185	0.969169	COG0035	F	Uracil phosphoribosyltransferase	K00761	METABOLISM
PA4659		probable transcriptional regulator	9.22E+08	0	0					
PA4661	pagL	Lipid A 3-O-deacylase	3.98E+08	2.46E+08	0.61781					
PA4665	prfA	peptide chain release factor 1	15121249	19493149	1.289123	COG0216	J	Protein chain release factor A	K02835	INFORMATION STORAGE AND PROCESSING
PA4670	prs	ribose-phosphate pyrophosphokinase	11156155	12142554	1.088417	COG0462	E	Phosphoribosylpyrophosphate synthetase	K00948	METABOLISM
PA4670	prs	ribose-phosphate pyrophosphokinase	11156155	12142554	1.088417	COG0462	F	Phosphoribosylpyrophosphate synthetase	K00948	METABOLISM
PA4671		probable ribosomal protein L25	4.62E+09	9.35E+09	2.024594	COG1825	J	Ribosomal protein L25 (general stress protein Ctc)	K02897	INFORMATION STORAGE AND PROCESSING
PA4673		conserved hypothetical protein	0	66266109	1000	COG0012	J	"Predicted GTPase, probable translation factor"	K06942	INFORMATION STORAGE AND PROCESSING
PA4674		Antitoxin HigA	7195030	0	0					
PA4686		hypothetical protein	0	20027165	1000	COG1196	D	Chromosome segregation ATPases	K03529	CELLULAR PROCESSES AND SIGNALING
PA4687	hitA	ferric iron-binding periplasmic protein HitA	86862974	60050029	0.691319	COG1840	P	"ABC-type Fe3 transport system, periplasmic component"	K02055	METABOLISM
PA4687	hitA	ferric iron-binding periplasmic protein HitA	86862974	60050029	0.691319	COG1840	P	"ABC-type Fe3 transport system, periplasmic component"	K02012	METABOLISM
PA4694	ilvC	ketol-acid reductoisomerase	9.76E+08	2.12E+09	2.175506	COG0059	E	Ketol-acid reductoisomerase	K00053	METABOLISM
PA4694	ilvC	ketol-acid reductoisomerase	9.76E+08	2.12E+09	2.175506	COG0059	H	Ketol-acid reductoisomerase	K00053	METABOLISM
PA4699		hypothetical protein	8.67E+08	7.01E+08	0.80847	COG3071	H	Uncharacterized enzyme of heme biosynthesis	K02498	METABOLISM
PA4701		conserved hypothetical protein	0	2758965	1000	COG0645	R	Predicted kinase	K07028	POORLY CHARACTERIZED
PA4701		conserved hypothetical protein	0	2758965	1000	COG2187	S	Uncharacterized protein conserved in bacteria	K07028	POORLY CHARACTERIZED
PA4702		hypothetical protein	33038128	1.23E+08	3.721956					
PA4708	phuT	"Heme-transport protein	51814521	0	0	COG4558	P	"ABC-type hemin transport system, periplasmic component"	K02016	METABOLISM
PA4723	dksA	suppressor protein DksA	3.61E+09	4.12E+09	1.141064	COG1734	T	DnaK suppressor protein	K06204	CELLULAR PROCESSES AND SIGNALING
PA4731	panD	aspartate 1-decarboxylase precursor	1.91E+08	0	0	COG0853	H	Aspartate 1-decarboxylase	K01579	METABOLISM
PA4738		conserved hypothetical protein	1.4E+09	1.73E+09	1.241167					
PA4739		conserved hypothetical protein	1.04E+10	1.01E+10	0.968848	COG2823	R	Predicted periplasmic or secreted lipoprotein	K04065	POORLY CHARACTERIZED
PA4740	pnp	polyribonucleotide nucleotidyltransferase	72171857	3.86E+08	5.343534	COG1185	J	Polyribonucleotide nucleotidyltransferase (polynucleotide pho	K00962	INFORMATION STORAGE AND PROCESSING
PA4740	pnp	polyribonucleotide nucleotidyltransferase	72171857	3.86E+08	5.343534	COG1185	J	Polyribonucleotide nucleotidyltransferase (polynucleotide pho	K12818	INFORMATION STORAGE AND PROCESSING
PA4741	rpsO	30S ribosomal protein S15	6.23E+09	2.73E+09	0.438765	COG0184	J	Ribosomal protein S15P/S13E	K02956	INFORMATION STORAGE AND PROCESSING
PA4744	infB	translation initiation factor IF-2	1.37E+08	6.6E+08	4.804771	COG0532	J	Translation initiation factor 2 (IF-2; GTPase)	K02519	INFORMATION STORAGE AND PROCESSING
PA4744	infB	translation initiation factor IF-2	1.37E+08	6.6E+08	4.804771	COG0532	J	Translation initiation factor 2 (IF-2; GTPase)	K03243	INFORMATION STORAGE AND PROCESSING
PA4745	nusA	N utilization substance protein A	3.4E+08	4.9E+08	1.439864	COG0195	K	Transcription elongation factor	K02600	INFORMATION STORAGE AND PROCESSING
PA4747	secG	secretion protein SecG	71306534	2.5E+08	3.508072	COG1314	U	Preprotein translocase subunit SecG	K03075	CELLULAR PROCESSES AND SIGNALING
PA4748	tpiA	triosephosphate isomerase	22454952	0	0	COG0149	G	Triosephosphate isomerase	K01803	METABOLISM
PA4751	ftsH	cell division protein FtsH	2.71E+09	1.63E+09	0.599435	COG0465	O	ATP-dependent Zn proteases	K03798	CELLULAR PROCESSES AND SIGNALING
PA4753		conserved hypothetical protein	3.12E+08	7.58E+08	2.433629	COG1534	J	"Predicted RNA-binding protein containing KH domain, possib	K07574	INFORMATION STORAGE AND PROCESSING
PA4755	greA	transcription elongation factor GreA	9.39E+09	9.38E+09	0.998738	COG0782	K	Transcription elongation factor	K04760	INFORMATION STORAGE AND PROCESSING

PA4755	greA	transcription elongation factor GreA	9.39E+09	9.38E+09	0.998738	COG0782	K	Transcription elongation factor	K06140	INFORMATION STORAGE AND PROCESSING
PA4755	greA	transcription elongation factor GreA	9.39E+09	9.38E+09	0.998738	COG0782	K	Transcription elongation factor	K03624	INFORMATION STORAGE AND PROCESSING
PA4756	carB	carbamoylphosphate synthetase large subunit	0	50156815	1000	COG0458	E	Carbamoylphosphate synthase large subunit (split gene in MJ)	K01955	METABOLISM
PA4756	carB	carbamoylphosphate synthetase large subunit	0	50156815	1000	COG0458	F	Carbamoylphosphate synthase large subunit (split gene in MJ)	K01955	METABOLISM
PA4758	carA	carbamoyl-phosphate synthase small chain	3.55E+08	2.56E+08	0.723208	COG0505	E	Carbamoylphosphate synthase small subunit	K01956	METABOLISM
PA4758	carA	carbamoyl-phosphate synthase small chain	3.55E+08	2.56E+08	0.723208	COG0505	F	Carbamoylphosphate synthase small subunit	K01956	METABOLISM
PA4759	dapB	dihydrodipicolinate reductase	0	21384862	1000	COG0289	E	Dihydrodipicolinate reductase	K00215	METABOLISM
PA4760	dnaJ	DnaJ protein	63214329	5.09E+08	8.045541	COG0484	O	DnaJ-class molecular chaperone with C-terminal Zn finger don	K03686	CELLULAR PROCESSES AND SIGNALING
PA4761	dnaK	DnaK protein	2.57E+10	1.43E+10	0.556526	COG0443	O	Molecular chaperone	K04044	CELLULAR PROCESSES AND SIGNALING
PA4761	dnaK	DnaK protein	2.57E+10	1.43E+10	0.556526	COG0443	O	Molecular chaperone	K04043	CELLULAR PROCESSES AND SIGNALING
PA4761	dnaK	DnaK protein	2.57E+10	1.43E+10	0.556526	COG0443	O	Molecular chaperone	K03283	CELLULAR PROCESSES AND SIGNALING
PA4761	dnaK	DnaK protein	2.57E+10	1.43E+10	0.556526	COG0443	O	Molecular chaperone	K04046	CELLULAR PROCESSES AND SIGNALING
PA4762	grpE	heat shock protein GrpE	2.35E+10	1.34E+10	0.570748	COG0576	O	Molecular chaperone GrpE (heat shock protein)	K03687	CELLULAR PROCESSES AND SIGNALING
PA4764	fur	ferric uptake regulation protein	5320599	29872955	5.614585	COG0735	P	Fe2? uptake regulation proteins	K09825	METABOLISM
PA4764	fur	ferric uptake regulation protein	5320599	29872955	5.614585	COG0735	P	Fe2? uptake regulation proteins	K09823	METABOLISM
PA4764	fur	ferric uptake regulation protein	5320599	29872955	5.614585	COG0735	P	Fe2? uptake regulation proteins	K03711	METABOLISM
PA4765	omlA	Outer membrane lipoprotein OmlA precursor	23884048	77588383	3.248544	COG2913	J	Small protein A (tmRNA-binding)	K06186	INFORMATION STORAGE AND PROCESSING
PA4778	cueR	CueR	2.86E+08	2.8E+08	0.977857					
PA4811	fdnH	"nitrate-inducible formate dehydrogenase	15700216	0	0	COG0437	C	Fe-S-cluster-containing hydrogenase components 1	K00184	METABOLISM
PA4811	fdnH	"nitrate-inducible formate dehydrogenase	15700216	0	0	COG0437	C	Fe-S-cluster-containing hydrogenase components 1	K04014	METABOLISM
PA4811	fdnH	"nitrate-inducible formate dehydrogenase	15700216	0	0	COG0437	C	Fe-S-cluster-containing hydrogenase components 1	K07307	METABOLISM
PA4811	fdnH	"nitrate-inducible formate dehydrogenase	15700216	0	0	COG0437	C	Fe-S-cluster-containing hydrogenase components 1	K00124	METABOLISM
PA4842		hypothetical protein	2.3E+08	82450680	0.357788					
PA4845	dipZ	thiol:disulfide interchange protein DipZ	75578093	65650342	0.868642	COG4232	O	Thiol:disulfide interchange protein	K04084	CELLULAR PROCESSES AND SIGNALING
PA4845	dipZ	thiol:disulfide interchange protein DipZ	75578093	65650342	0.868642	COG4232	C	Thiol:disulfide interchange protein	K04084	METABOLISM
PA4847	accB	biotin carboxyl carrier protein (BCCP)	1.53E+08	2.33E+08	1.521237	COG0511	I	Biotin carboxyl carrier protein	K01960	METABOLISM
PA4847	accB	biotin carboxyl carrier protein (BCCP)	1.53E+08	2.33E+08	1.521237	COG0511	I	Biotin carboxyl carrier protein	K02160	METABOLISM
PA4847	accB	biotin carboxyl carrier protein (BCCP)	1.53E+08	2.33E+08	1.521237	COG0511	I	Biotin carboxyl carrier protein	K01571	METABOLISM
PA4848	accC	biotin carboxylase	54620340	2.57E+08	4.704749	COG0439	I	Biotin carboxylase	K01961	METABOLISM
PA4848	accC	biotin carboxylase	54620340	2.57E+08	4.704749	COG0439	I	Biotin carboxylase	K01959	METABOLISM
PA4851		hypothetical protein	59176288	2.45E+08	4.137446					
PA4853	fis	DNA-binding protein Fis	8.62E+08	4.79E+08	0.555318	COG2901	K	"Factor for inversion stimulation Fis, transcriptional activator"	K03557	INFORMATION STORAGE AND PROCESSING
PA4853	fis	DNA-binding protein Fis	8.62E+08	4.79E+08	0.555318	COG2901	K	"Factor for inversion stimulation Fis, transcriptional activator"	K03557	INFORMATION STORAGE AND PROCESSING
PA4853	fis	DNA-binding protein Fis	8.62E+08	4.79E+08	0.555318	COG2901	L	"Factor for inversion stimulation Fis, transcriptional activator"	K03557	INFORMATION STORAGE AND PROCESSING
PA4853	fis	DNA-binding protein Fis	8.62E+08	4.79E+08	0.555318	COG2901	K	"Factor for inversion stimulation Fis, transcriptional activator"	K03557	INFORMATION STORAGE AND PROCESSING
PA4853	fis	DNA-binding protein Fis	8.62E+08	4.79E+08	0.555318	COG2901	L	"Factor for inversion stimulation Fis, transcriptional activator"	K03557	INFORMATION STORAGE AND PROCESSING
PA4853	fis	DNA-binding protein Fis	8.62E+08	4.79E+08	0.555318	COG2901	L	"Factor for inversion stimulation Fis, transcriptional activator"	K03557	INFORMATION STORAGE AND PROCESSING
PA4853	fis	DNA-binding protein Fis	8.62E+08	4.79E+08	0.555318	COG2901	L	"Factor for inversion stimulation Fis, transcriptional activator"	K03557	INFORMATION STORAGE AND PROCESSING
PA4853	fis	DNA-binding protein Fis	8.62E+08	4.79E+08	0.555318	COG2901	K	"Factor for inversion stimulation Fis, transcriptional activator"	K03557	INFORMATION STORAGE AND PROCESSING
PA4855	purD	phosphoribosylamine--glycine ligase	0	14713446	1000	COG0151	F	Phosphoribosylamine-glycine ligase	K01945	METABOLISM
PA4865	ureA	urease gamma subunit	1.07E+08	2.15E+08	2.003667	COG0831	E	Urea amidohydrolase (urease) gamma subunit	K14048	METABOLISM
PA4865	ureA	urease gamma subunit	1.07E+08	2.15E+08	2.003667	COG0831	E	Urea amidohydrolase (urease) gamma subunit	K01430	METABOLISM
PA4867	ureB	urease beta subunit	1.77E+08	2.03E+08	1.146966	COG0832	E	Urea amidohydrolase (urease) beta subunit	K01429	METABOLISM
PA4867	ureB	urease beta subunit	1.77E+08	2.03E+08	1.146966	COG0832	E	Urea amidohydrolase (urease) beta subunit	K14048	METABOLISM
PA4876	osmE	osmotically inducible lipoprotein OsmE	1.31E+09	2E+09	1.52307	COG2913	J	Small protein A (tmRNA-binding)	K06186	INFORMATION STORAGE AND PROCESSING
PA4880		probable bacterioferritin	1.68E+09	1.18E+09	0.703884	COG2193	P	Bacterioferritin (cytochrome b1)	K03594	METABOLISM
PA4885	irlR	two-component response regulator	0	96099769	1000	COG0745	K	Response regulators consisting of a CheY-like receiver domain	K02483	INFORMATION STORAGE AND PROCESSING
PA4885	irlR	two-component response regulator	0	96099769	1000	COG0745	T	Response regulators consisting of a CheY-like receiver domain	K02483	CELLULAR PROCESSES AND SIGNALING
PA4907		probable short-chain dehydrogenase	0	11643346	1000	COG4221	R	Short-chain alcohol dehydrogenase of unknown specificity	K00540	POORLY CHARACTERIZED
PA4915		probable chemotaxis transducer	1.46E+08	1.04E+09	7.094225	COG0840	N	Methyl-accepting chemotaxis protein	K03406	CELLULAR PROCESSES AND SIGNALING
PA4915		probable chemotaxis transducer	1.46E+08	1.04E+09	7.094225	COG0840	T	Methyl-accepting chemotaxis protein	K03406	CELLULAR PROCESSES AND SIGNALING
PA4920	nadE	NH3-dependent NAD synthetase	1.63E+09	83573061	0.051281	COG0171	H	NAD synthase	K01916	METABOLISM
PA4920	nadE	NH3-dependent NAD synthetase	1.63E+09	83573061	0.051281	COG0171	H	NAD synthase	K01950	METABOLISM
PA4922	azu	azurin precursor	2.45E+10	1.78E+10	0.724925					
PA4932	rplI	50S ribosomal protein L9	4.88E+09	9.65E+09	1.977174	COG0359	J	Ribosomal protein L9	K02939	INFORMATION STORAGE AND PROCESSING
PA4934	rpsR	30S ribosomal protein S18	3.75E+08	1.15E+09	3.068901	COG0238	J	Ribosomal protein S18	K02963	INFORMATION STORAGE AND PROCESSING
PA4935	rpsF	30S ribosomal protein S6	2.28E+10	2.29E+10	1.004138	COG0360	J	Ribosomal protein S6	K02990	INFORMATION STORAGE AND PROCESSING
PA4936		probable rRNA methylase	7435867	0	0	COG0566	J	rRNA methylases	K00556	INFORMATION STORAGE AND PROCESSING
PA4936		probable rRNA methylase	7435867	0	0	COG0566	J	rRNA methylases	K03437	INFORMATION STORAGE AND PROCESSING
PA4936		probable rRNA methylase	7435867	0	0	COG0566	J	rRNA methylases	K03218	INFORMATION STORAGE AND PROCESSING
PA4936		probable rRNA methylase	7435867	0	0	COG0566	J	rRNA methylases	K00599	INFORMATION STORAGE AND PROCESSING
PA4937	rnr	exoribonuclease RNase R	9.27E+08	2.09E+09	2.252897	COG0557	K	Exoribonuclease R	K01147	INFORMATION STORAGE AND PROCESSING
PA4937	rnr	exoribonuclease RNase R	9.27E+08	2.09E+09	2.252897	COG0557	K	Exoribonuclease R	K12573	INFORMATION STORAGE AND PROCESSING
PA4938	purA	adenylosuccinate synthetase	90573771	1.12E+08	1.234677	COG0104	F	Adenylosuccinate synthase	K01939	METABOLISM
PA4941	hflC	protease subunit HflC	0	96029761	1000	COG0330	O	"Membrane protease subunits, stomatin/prohibitin homologs	K04088	CELLULAR PROCESSES AND SIGNALING
PA4941	hflC	protease subunit HflC	0	96029761	1000	COG0330	O	"Membrane protease subunits, stomatin/prohibitin homologs	K04087	CELLULAR PROCESSES AND SIGNALING
PA4942	hflK	protease subunit HflK	8.99E+09	6.46E+09	0.718009	COG0330	O	"Membrane protease subunits, stomatin/prohibitin homologs	K04088	CELLULAR PROCESSES AND SIGNALING
PA4942	hflK	protease subunit HflK	8.99E+09	6.46E+09	0.718009	COG0330	O	"Membrane protease subunits, stomatin/prohibitin homologs	K04087	CELLULAR PROCESSES AND SIGNALING
PA4944	hfq	Hfq	1.17E+09	3.99E+09	3.423517	COG1923	R	Uncharacterized host factor I protein	K03666	POORLY CHARACTERIZED
PA4951	orn	oligoribonuclease	84823603	37158571	0.438069	COG1949	A	Oligoribonuclease (3'->5' exoribonuclease)	K13288	INFORMATION STORAGE AND PROCESSING
PA4953	motB	chemotaxis protein MotB	21503383	3.24E+08	15.07846	COG1360	N	Flagellar motor protein	K02557	CELLULAR PROCESSES AND SIGNALING
PA4953	motB	chemotaxis protein MotB	21503383	3.24E+08	15.07846	COG1360	N	Flagellar motor protein	K11892	CELLULAR PROCESSES AND SIGNALING
PA4956	rhdA	thiosulfate:cyanide sulfurtransferase	78361211	41723825	0.532455	COG2897	P	Rhodanese-related sulfurtransferase	K01011	METABOLISM
PA4963		hypothetical protein	40909597	3.23E+08	7.885884	COG3009	S	Uncharacterized protein conserved in bacteria	K09857	POORLY CHARACTERIZED
PA4974		probable outer membrane protein precursor	2.51E+09	2.61E+09	1.040435	COG1538	U	Outer membrane protein	K12340	CELLULAR PROCESSES AND SIGNALING
PA4974		probable outer membrane protein precursor	2.51E+09	2.61E+09	1.040435	COG1538	M	Outer membrane protein	K12340	CELLULAR PROCESSES AND SIGNALING
PA4974		probable outer membrane protein precursor	2.51E+09	2.61E+09	1.040435	COG1538	U	Outer membrane protein	K03287	CELLULAR PROCESSES AND SIGNALING
PA4974		probable outer membrane protein precursor	2.51E+09	2.61E+09	1.040435	COG1538	M	Outer membrane protein	K03287	CELLULAR PROCESSES AND SIGNALING
PA4976	aruH	"Arginine:Pyruvate Transaminas	1.2E+08	0	0	COG0436	E	Aspartate/tyrosine/aromatic aminotransferase	K00812	METABOLISM
PA4976	aruH	"Arginine:Pyruvate Transaminas	1.2E+08	0	0	COG0436	E	Aspartate/tyrosine/aromatic aminotransferase	K14260	METABOLISM
PA4979		probable acyl-CoA dehydrogenase	0	2.21E+08	1000	COG1960	I	Acyl-CoA dehydrogenases	K00249	METABOLISM
PA4979		probable acyl-CoA dehydrogenase	0	2.21E+08	1000	COG1960	I	Acyl-CoA dehydrogenases	K00257	METABOLISM
PA5015	aceE	pyruvate dehydrogenase	1.82E+08	2.59E+08	1.420444	COG2609	C	"Pyruvate dehydrogenase complex, dehydrogenase (E1) comp	K00163	METABOLISM
PA5016	aceF	dihydrolipoamide acetyltransferase	3.74E+09	2.29E+09	0.614161	COG0508	C	"Pyruvate/2-oxoglutarate dehydrogenase complex, dihydrolip	K00627	METABOLISM
PA5016	aceF	dihydrolipoamide acetyltransferase	3.74E+09	2.29E+09	0.614161	COG0508	C	"Pyruvate/2-oxoglutarate dehydrogenase complex, dihydrolip	K00658	METABOLISM
PA5016	aceF	dihydrolipoamide acetyltransferase	3.74E+09	2.29E+09	0.614161	COG0508	C	"Pyruvate/2-oxoglutarate dehydrogenase complex, dihydrolip	K09699	METABOLISM
PA5019		conserved hypothetical protein	45431225	0	0	COG2961	R	Protein involved in catabolism of external DNA	K07115	POORLY CHARACTERIZED
PA5027		hypothetical protein	5.08E+08	5.11E+08	1.004232	COG0589	T	Universal stress protein UspA and related nucleotide-binding	K06149	CELLULAR PROCESSES AND SIGNALING
PA5042	pilO	type 4 fimbrial biogenesis protein PilO	31906649	28842412	0.903962	COG3167	N	Tfp pilus assembly protein PilO	K02664	CELLULAR PROCESSES AND SIGNALING
PA5042	pilO	type 4 fimbrial biogenesis protein PilO	31906649	28842412	0.903962	COG3167	U	Tfp pilus assembly protein PilO	K02664	CELLULAR PROCESSES AND SIGNALING
PA5046		malic enzyme	3.6E+08	1.43E+09	3.980084	COG0281	C	Malic enzyme	K00029	METABOLISM
PA5046		malic enzyme	3.6E+08	1.43E+09	3.980084	COG0281	C	Malic enzyme	K00027	METABOLISM
PA5049	rpmE	50S ribosomal protein L31	3.54E+09	6.01E+08	0.169918	COG0254	J	Ribosomal protein L31	K02909	INFORMATION STORAGE AND PROCESSING
PA5051	argS	arginyl-tRNA synthetase	28010731	89181655	3.183839	COG0018	J	Arginyl-tRNA synthetase	K01887	INFORMATION STORAGE AND PROCESSING
PA5054	hslU	heat shock protein HslU	1.63E+08	3.51E+08	2.159422	COG1220	O	"ATP-dependent protease HslVU (ClpYQ), ATPase subunit"	K03667	CELLULAR PROCESSES AND SIGNALING
PA5058	phaC2	poly(3-hydroxyalkanoic acid) synthase 2	0	7.11E+08	1000	COG3243	I	Poly(3-hydroxyalkanoate) synthetase	K03821	METABOLISM
PA5058	phaC2	poly(3-hydroxyalkanoic acid) synthase 2	0	7.11E+08	1000	COG3243	I	Poly(3-hydroxyalkanoate) synthetase	K03822	METABOLISM
PA5060	phaF	polyhydroxyalkanoate synthesis protein PhaF	4.82E+09	3.38E+09	0.702177					
PA5061		conserved hypothetical protein	57491760	29415722	0.511651					
PA5062		conserved hypothetical protein	0	36130889	1000					
PA5063	ubiE	ubiquinone biosynthesis methyltransferase UbiE	2.28E+08	2.66E+08	1.166716	COG2226	H	Methylase involved in ubiquinone/menaquinone biosynthesis	K03183	METABOLISM
PA5064		hypothetical protein	1.66E+08	0	0	COG3165	S	Uncharacterized protein conserved in bacteria	K03690	POORLY CHARACTERIZED
PA5065	ubiB	ubiquinone biosynthetic protein UbiB	3.47E+08	1.61E+08	0.464939	COG0661	R	Predicted unusual protein kinase	K08869	POORLY CHARACTERIZED
PA5065	ubiB	ubiquinone biosynthetic protein UbiB	3.47E+08	1.61E+08	0.464939	COG0661	R	Predicted unusual protein kinase	K03688	POORLY CHARACTERIZED
PA5067	hisE	phosphoribosyl-ATP pyrophosphohydrolase	3.39E+08	2.05E+08	0.606148	COG0140	E	Phosphoribosyl-ATP pyrophosphohydrolase	K11755	METABOLISM
PA5067	hisE	phosphoribosyl-ATP pyrophosphohydrolase	3.39E+08	2.05E+08	0.606148	COG0140	E	Phosphoribosyl-ATP pyrophosphohydrolase	K01523	METABOLISM
PA5068	tatA	translocation protein TatA	1.82E+08	4.37E+08	2.40154	COG1826	U	Sec-independent protein secretion pathway components	K03425	CELLULAR PROCESSES AND SIGNALING
PA5068	tatA	translocation protein TatA	1.82E+08	4.37E+08	2.40154	COG1826	U	Sec-independent protein secretion pathway components	K03116	CELLULAR PROCESSES AND SIGNALING
PA5068	tatA	translocation protein TatA	1.82E+08	4.37E+08	2.40154	COG1826	U	Sec-independent protein secretion pathway components	K03117	CELLULAR PROCESSES AND SIGNALING
PA5069	tatB	translocation protein TatB	26706044	0	0					
PA5072	mcpK	McpK (Probable chemotaxis transducer)	0	6.97E+08	1000	COG0840	N	Methyl-accepting chemotaxis protein	K03406	CELLULAR PROCESSES AND SIGNALING
PA5072	mcpK	McpK (Probable chemotaxis transducer)	0	6.97E+08	1000	COG0840	T	Methyl-accepting chemotaxis protein	K03406	CELLULAR PROCESSES AND SIGNALING
PA5077	opgH	OpgH	19446700	0	0	COG2943	M	Membrane glycosyltransferase	K03669	CELLULAR PROCESSES AND SIGNALING
PA5078	opgG	OpgG(Glucans biosynthesis protein G)	0	2.71E+08	1000	COG3131	P	Periplasmic glucans biosynthesis protein	K03670	METABOLISM
PA5082	dguC	DguC	45587362	31230953	0.685079	COG0834	T	"ABC-type amino acid transport/signal transduction systems,	K02030	CELLULAR PROCESSES AND SIGNALING
PA5082	dguC	DguC	45587362	31230953	0.685079	COG0834	E	"ABC-type amino acid transport/signal transduction systems,	K02030	METABOLISM
PA5108		hypothetical protein	2.64E+08	3.18E+08	1.20565					
PA5110	fbp	"fructose-1	80846980	1.68E+08	2.077501	COG0158	G	"Fructose-1,6-bisphosphatase"	K03841	METABOLISM
PA5117	typA	regulatory protein TypA	0	1.66E+08	1000	COG1217	T	Predicted membrane GTPase involved in stress response	K06207	CELLULAR PROCESSES AND SIGNALING
PA5119	glnA	glutamine synthetase	0	1.09E+08	1000	COG0174	E	Glutamine synthetase	K01915	METABOLISM
PA5128	secB	secretion protein SecB	52625325	63333464	1.203479	COG1952	U	Preprotein translocase subunit SecB	K03071	CELLULAR PROCESSES AND SIGNALING

PA5129	grxC	GrxC	10253117	0	0	COG0695	O	Glutaredoxin and related proteins	K06191	CELLULAR PROCESSES AND SIGNALING
PA5129	grxC	GrxC	10253117	0	0	COG0695	O	Glutaredoxin and related proteins	K03674	CELLULAR PROCESSES AND SIGNALING
PA5129	grxC	GrxC	10253117	0	0	COG0695	O	Glutaredoxin and related proteins	K03676	CELLULAR PROCESSES AND SIGNALING
PA5130		conserved hypothetical protein	2.62E+09	2.01E+09	0.767173	COG0607	P	Rhodanese-related sulfurtransferase	K03151	METABOLISM
PA5131	pgm	phosphoglycerate mutase	0	47150248	1000	COG0696	G	Phosphoglyceromutase	K01834	METABOLISM
PA5133		conserved hypothetical protein	1.84E+08	91685672	0.498448					
PA5134	ctpA	"carboxyl-terminal processing protease	31858923	86020620	2.700048	COG0793	M	Periplasmic protease	K03797	CELLULAR PROCESSES AND SIGNALING
PA5135		conserved hypothetical protein	16504049	0	0	COG2861	S	Uncharacterized protein conserved in bacteria	K09798	POORLY CHARACTERIZED
PA5141	hisA	phosphoribosylformimino-5-aminoimidazole carboxamide	1.16E+08	2.68E+08	2.308401	COG0106	E	Phosphoribosylformimino-5-aminoimidazole carboxamide rib	K01817	METABOLISM
PA5141	hisA	phosphoribosylformimino-5-aminoimidazole carboxamide	1.16E+08	2.68E+08	2.308401	COG0106	E	Phosphoribosylformimino-5-aminoimidazole carboxamide rib	K01814	METABOLISM
PA5146		hypothetical protein	56265454	0	0	COG2982	M	Uncharacterized protein involved in outer membrane biogene	K07289	CELLULAR PROCESSES AND SIGNALING
PA5146		hypothetical protein	56265454	0	0	COG2982	M	Uncharacterized protein involved in outer membrane biogene	K07290	CELLULAR PROCESSES AND SIGNALING
PA5148		conserved hypothetical protein	1.82E+09	1.38E+09	0.75572					
PA5161	rmlB	"dTDP-D-glucose 4	0	19243681	1000	COG1088	M	"dTDP-D-glucose 4,6-dehydratase"	K01710	CELLULAR PROCESSES AND SIGNALING
PA5171	arcA	arginine deiminase	96118557	1.14E+09	11.88473	COG2235	E	Arginine deiminase	K01478	METABOLISM
PA5172	arcB	"ornithine carbamoyltransferase	3.35E+08	4.53E+09	13.51179	COG0078	E	Ornithine carbamoyltransferase	K00611	METABOLISM
PA5173	arcC	carbamate kinase	4.23E+08	2.63E+09	6.210332	COG0549	E	Carbamate kinase	K00926	METABOLISM
PA5178		conserved hypothetical protein	1.63E+10	1.16E+10	0.711563					
PA5192	pckA	phosphoenolpyruvate carboxykinase	0	27157861	1000	COG1866	C	Phosphoenolpyruvate carboxykinase (ATP)	K01610	METABOLISM
PA5200	amgR	AmgR	1.71E+08	76408189	0.44604	COG0745	T	Response regulators consisting of a CheY-like receiver domain	K02483	CELLULAR PROCESSES AND SIGNALING
PA5200	amgR	AmgR	1.71E+08	76408189	0.44604	COG0745	K	Response regulators consisting of a CheY-like receiver domain	K02483	INFORMATION STORAGE AND PROCESSING
PA5201		conserved hypothetical protein	69782612	1.51E+08	2.165771	COG2183	K	Transcriptional accessory protein	K11292	INFORMATION STORAGE AND PROCESSING
PA5201		conserved hypothetical protein	69782612	1.51E+08	2.165771	COG2183	K	Transcriptional accessory protein	K06959	INFORMATION STORAGE AND PROCESSING
PA5207		probable phosphate transporter	0	6637345	1000	COG0306	P	Phosphate/sulphate permeases	K03306	METABOLISM
PA5214	gcvH1	glycine cleavage system protein H1	6.03E+08	6.25E+08	1.036619	COG0509	E	Glycine cleavage system H protein (lipoate-binding)	K02437	METABOLISM
PA5225		hypothetical protein	76478271	38620000	0.50498	COG3079	S	Uncharacterized protein conserved in bacteria	K09895	POORLY CHARACTERIZED
PA5226		hypothetical protein	68984141	5.05E+08	7.323623					
PA5227		conserved hypothetical protein	7.31E+08	9.5E+08	1.299332	COG3027	S	Uncharacterized protein conserved in bacteria	K09888	POORLY CHARACTERIZED
PA5232		conserved hypothetical protein(Biotin_lipoyl_2 domain-containin	0	1.36E+08	1000	COG0845	M	Membrane-fusion protein	K02005	CELLULAR PROCESSES AND SIGNALING
PA5232		conserved hypothetical protein	0	1.36E+08	1000	COG0845	M	Membrane-fusion protein	K03585	CELLULAR PROCESSES AND SIGNALING
PA5232		conserved hypothetical protein	0	1.36E+08	1000	COG0845	M	Membrane-fusion protein	K01993	CELLULAR PROCESSES AND SIGNALING
PA5232		conserved hypothetical protein	0	1.36E+08	1000	COG0845	M	Membrane-fusion protein	K02022	CELLULAR PROCESSES AND SIGNALING
PA5233		hypothetical protein	5.39E+08	3.57E+08	0.66203	COG1580	N	Flagellar basal body-associated protein	K02415	CELLULAR PROCESSES AND SIGNALING
PA5239	rho	transcription termination factor Rho	2.31E+08	1.53E+09	6.614102	COG1158	K	Transcription termination factor	K03628	INFORMATION STORAGE AND PROCESSING
PA5240	trxA	thioredoxin	1.9E+10	1.51E+10	0.793673	COG3118	O	Thioredoxin domain-containing protein	K05838	CELLULAR PROCESSES AND SIGNALING
PA5243	hemB	delta-aminolevulinic acid dehydratase	4.34E+09	18582447	4.28E-03	COG0113	H	Delta-aminolevulinic acid dehydratase	K01698	METABOLISM
PA5245		conserved hypothetical protein	1.52E+08	30050609	0.197181					
PA5253	algP	alginate regulatory protein AlgP	4.8E+09	3.46E+08	7.20E-02					
PA5255	algQ	Alginate regulatory protein AlgQ	0	73528540	1000	COG3160	K	Regulator of sigma D	K07740	INFORMATION STORAGE AND PROCESSING
PA5258		hypothetical protein	9.63E+08	6.18E+08	0.64124	COG2959	H	Uncharacterized enzyme of heme biosynthesis	K13543	METABOLISM
PA5258		hypothetical protein	9.63E+08	6.18E+08	0.64124	COG2959	H	Uncharacterized enzyme of heme biosynthesis	K02496	METABOLISM
PA5263	argH	argininosuccinate lyase	0	3.22E+08	1000	COG0165	E	Argininosuccinate lyase	K01755	METABOLISM
PA5269		hypothetical protein	2.48E+08	3.68E+08	1.482267					
PA5271		hypothetical protein	81261677	28793481	0.35433					
PA5274	rnk	nucleoside diphosphate kinase regulator	34566966	0	0	COG0782	K	Transcription elongation factor	K04760	INFORMATION STORAGE AND PROCESSING
PA5274	rnk	nucleoside diphosphate kinase regulator	34566966	0	0	COG0782	K	Transcription elongation factor	K03624	INFORMATION STORAGE AND PROCESSING
PA5274	rnk	nucleoside diphosphate kinase regulator	34566966	0	0	COG0782	K	Transcription elongation factor	K06140	INFORMATION STORAGE AND PROCESSING
PA5275		conserved hypothetical protein	0	53151337	1000	COG1965	P	"Protein implicated in iron transport, frataxin homolog"	K06202	METABOLISM
PA5275		conserved hypothetical protein	0	53151337	1000	COG1965	P	"Protein implicated in iron transport, frataxin homolog"	K06202	METABOLISM
PA5275		conserved hypothetical protein	0	53151337	1000	COG1965	P	"Protein implicated in iron transport, frataxin homolog"	K06202	METABOLISM
PA5275		conserved hypothetical protein	0	53151337	1000	COG1965	P	"Protein implicated in iron transport, frataxin homolog"	K06202	METABOLISM
PA5277	lysA	diaminopimelate decarboxylase	0	18061379	1000	COG0019	E	Diaminopimelate decarboxylase	K13747	METABOLISM
PA5277	lysA	diaminopimelate decarboxylase	0	18061379	1000	COG0019	E	Diaminopimelate decarboxylase	K01581	METABOLISM
PA5277	lysA	diaminopimelate decarboxylase	0	18061379	1000	COG0019	E	Diaminopimelate decarboxylase	K01586	METABOLISM
PA5285	sutA	SutA	3.01E+09	3.06E+09	1.017789					
PA5288	glnK	nitrogen regulatory protein P-II 2	3.63E+09	2.39E+09	0.658651	COG0347	E	Nitrogen regulatory protein PII	K04751	METABOLISM
PA5288	glnK	nitrogen regulatory protein P-II 2	3.63E+09	2.39E+09	0.658651	COG0347	E	Nitrogen regulatory protein PII	K04752	METABOLISM
PA5289		hypothetical protein	1.16E+08	75468596	0.650873	COG2960	S	Uncharacterized protein conserved in bacteria	K09806	POORLY CHARACTERIZED
PA5298		xanthine phosphoribosyltransferase	37986072	16021822	0.421781	COG0503	F	Adenine/guanine phosphoribosyltransferases and related PRP	K00759	METABOLISM
PA5298		xanthine phosphoribosyltransferase	37986072	16021822	0.421781	COG0503	F	Adenine/guanine phosphoribosyltransferases and related PRP	K03816	METABOLISM
PA5298		xanthine phosphoribosyltransferase	37986072	16021822	0.421781	COG0503	F	Adenine/guanine phosphoribosyltransferases and related PRP	K00769	METABOLISM
PA5298		xanthine phosphoribosyltransferase	37986072	16021822	0.421781	COG0503	F	Adenine/guanine phosphoribosyltransferases and related PRP	K09685	METABOLISM
PA5300	cycB	cytochrome c5	9.5E+09	8.29E+09	0.87209					
PA5303		conserved hypothetical protein	55218970	54696926	0.990546	COG0251	J	"Putative translation initiation inhibitor, yjgF family"	K07567	INFORMATION STORAGE AND PROCESSING
PA5305		conserved hypothetical protein	2.78E+08	13029077	4.69E-02	COG3784	S	Uncharacterized protein conserved in bacteria	K09978	POORLY CHARACTERIZED
PA5312	pauC	Aldehyde dehydrogenase	0	8114101	1000	COG1012	C	NAD-dependent aldehyde dehydrogenases	K00128	METABOLISM
PA5312	pauC	Aldehyde dehydrogenase	0	8114101	1000	COG1012	C	NAD-dependent aldehyde dehydrogenases	K00135	METABOLISM
PA5312	pauC	Aldehyde dehydrogenase	0	8114101	1000	COG1012	C	NAD-dependent aldehyde dehydrogenases	K00140	METABOLISM
PA5312	pauC	Aldehyde dehydrogenase	0	8114101	1000	COG1012	C	NAD-dependent aldehyde dehydrogenases	K00130	METABOLISM
PA5315	rpmG	50S ribosomal protein L33	7.92E+09	5.4E+09	0.681843	COG0267	J	Ribosomal protein L33	K02913	INFORMATION STORAGE AND PROCESSING
PA5316	rpmB	50S ribosomal protein L28	0	56720674	1000	COG0227	J	Ribosomal protein L28	K02902	INFORMATION STORAGE AND PROCESSING
PA5322	algC	phosphomannomutase AlgC	0	64005388	1000	COG1109	G	Phosphomannomutase	K01840	METABOLISM
PA5322	algC	phosphomannomutase AlgC	0	64005388	1000	COG1109	G	Phosphomannomutase	K03431	METABOLISM
PA5322	algC	phosphomannomutase AlgC	0	64005388	1000	COG1109	G	Phosphomannomutase	K01835	METABOLISM
PA5322	algC	phosphomannomutase AlgC	0	64005388	1000	COG4191	T	Signal transduction histidine kinase regulating C4-dicarboxylat	K10125	CELLULAR PROCESSES AND SIGNALING
PA5323	argB	acetylglutamate kinase	3.57E+08	5.42E+08	1.516337	COG0548	E	Acetylglutamate kinase	K00619	METABOLISM
PA5323	argB	acetylglutamate kinase	3.57E+08	5.42E+08	1.516337	COG0548	E	Acetylglutamate kinase	K00930	METABOLISM
PA5330		hypothetical protein	2.64E+08	32019556	0.121139					
PA5336	gmk	guanylate kinase	0	2.47E+08	1000	COG0194	F	Guanylate kinase	K00942	METABOLISM
PA5337	rpoZ	RNA polymerase omega subunit	3.79E+09	3.1E+09	0.818469	COG1758	K	"DNA-directed RNA polymerase, subunit K/omega"	K03060	INFORMATION STORAGE AND PROCESSING
PA5337	rpoZ	RNA polymerase omega subunit	3.79E+09	3.1E+09	0.818469	COG1758	K	"DNA-directed RNA polymerase, subunit K/omega"	K03014	INFORMATION STORAGE AND PROCESSING
PA5337	rpoZ	RNA polymerase omega subunit	3.79E+09	3.1E+09	0.818469	COG1758	K	"DNA-directed RNA polymerase, subunit K/omega"	K03055	INFORMATION STORAGE AND PROCESSING
PA5339		conserved hypothetical protein	1.05E+10	1.98E+09	0.188597	COG0251	J	"Putative translation initiation inhibitor, yjgF family"	K07567	INFORMATION STORAGE AND PROCESSING
PA5340		hypothetical protein	1.2E+08	1.88E+08	1.568686					
PA5348		probable DNA-binding protein	2.36E+09	8.26E+08	0.34945	COG0776	L	Bacterial nucleoid DNA-binding protein	K05788	INFORMATION STORAGE AND PROCESSING
PA5348		probable DNA-binding protein	2.36E+09	8.26E+08	0.34945	COG0776	L	Bacterial nucleoid DNA-binding protein	K04764	INFORMATION STORAGE AND PROCESSING
PA5348		probable DNA-binding protein	2.36E+09	8.26E+08	0.34945	COG0776	L	Bacterial nucleoid DNA-binding protein	K05787	INFORMATION STORAGE AND PROCESSING
PA5348		probable DNA-binding protein	2.36E+09	8.26E+08	0.34945	COG0776	L	Bacterial nucleoid DNA-binding protein	K03530	INFORMATION STORAGE AND PROCESSING
PA5355	glcD	glycolate oxidase subunit GlcD	3.05E+08	6.2E+08	2.032656	COG0277	C	FAD/FMN-containing dehydrogenases	K11472	METABOLISM
PA5355	glcD	glycolate oxidase subunit GlcD	3.05E+08	6.2E+08	2.032656	COG0277	C	FAD/FMN-containing dehydrogenases	K00102	METABOLISM
PA5355	glcD	glycolate oxidase subunit GlcD	3.05E+08	6.2E+08	2.032656	COG0277	C	FAD/FMN-containing dehydrogenases	K00104	METABOLISM
PA5355	glcD	glycolate oxidase subunit GlcD	3.05E+08	6.2E+08	2.032656	COG0277	C	FAD/FMN-containing dehydrogenases	K06911	METABOLISM
PA5359		hypothetical protein	11171170	20512100	1.836164					
PA5381		hypothetical protein	1.04E+08	0	0					
PA5406		hypothetical protein	1.91E+08	1.74E+08	0.911167					
PA5414		hypothetical protein	1.19E+08	2.21E+08	1.85829					
PA5426	purE	"phosphoribosylaminoimidazole carboxylase	0	28610859	1000	COG0041	F	Phosphoribosylcarboxyaminoimidazole (NCAIR) mutase	K01588	METABOLISM
PA5427	adhA	alcohol dehydrogenase	0	90585723	1000	COG1064	R	Zn-dependent alcohol dehydrogenases	K13979	POORLY CHARACTERIZED
PA5427	adhA	alcohol dehydrogenase	0	90585723	1000	COG1064	R	Zn-dependent alcohol dehydrogenases	K13953	POORLY CHARACTERIZED
PA5427	adhA	alcohol dehydrogenase	0	90585723	1000	COG1064	R	Zn-dependent alcohol dehydrogenases	K00001	POORLY CHARACTERIZED
PA5429	aspA	aspartate ammonia-lyase	7051843	0	0	COG1027	E	Aspartate ammonia-lyase	K01744	METABOLISM
PA5435		probable transcarboxylase subunit	0	46509866	1000	COG0511	I	Biotin carboxyl carrier protein	K01960	METABOLISM
PA5435		probable transcarboxylase subunit	0	46509866	1000	COG5016	C	Pyruvate/oxaloacetate carboxyltransferase	K01571	METABOLISM
PA5435		probable transcarboxylase subunit	0	46509866	1000	COG0511	I	Biotin carboxyl carrier protein	K02160	METABOLISM
PA5435		probable transcarboxylase subunit	0	46509866	1000	COG0511	I	Biotin carboxyl carrier protein	K01571	METABOLISM
PA5435		probable transcarboxylase subunit	0	46509866	1000	COG5016	C	Pyruvate/oxaloacetate carboxyltransferase	K01960	METABOLISM
PA5441		hypothetical protein	94230702	0	0					
PA5472		hypothetical protein	2.02E+08	33263886	0.164912	COG0834	E	"ABC-type amino acid transport/signal transduction systems,	K02030	METABOLISM
PA5472		hypothetical protein	2.02E+08	33263886	0.164912	COG0834	T	"ABC-type amino acid transport/signal transduction systems,	K02030	CELLULAR PROCESSES AND SIGNALING
PA5481		hypothetical protein	1.07E+08	0	0					
PA5487		hypothetical protein	56225235	70676291	1.257021	COG3706	T	Response regulator containing a CheY-like receiver domain an	K02488	CELLULAR PROCESSES AND SIGNALING
PA5489	dsbA	thiol:disulfide interchange protein DsbA	0	35978076	1000	COG1651	O	Protein-disulfide isomerase	K03981	CELLULAR PROCESSES AND SIGNALING
PA5489	dsbA	thiol:disulfide interchange protein DsbA	0	35978076	1000	COG1651	O	Protein-disulfide isomerase	K03805	CELLULAR PROCESSES AND SIGNALING
PA5490	cc4	cytochrome c4 precursor	2.7E+09	1.23E+09	0.453575					
PA5494		hypothetical protein	15905241	0	0					
PA5496	nrdJb	"class II (cobalamin-dependent) ribonucleotide-diphosphate redu	1.14E+08	0	0					
PA5505		probable TonB-dependent receptor	4.09E+09	1.39E+09	0.339164	COG1464	P	"ABC-type metal ion transport system, periplasmic componen	K02073	METABOLISM
PA5528		hypothetical protein	58441362	1.22E+08	2.09066					
PA5553	atpC	ATP synthase epsilon chain	2.04E+08	2.66E+08	1.304465	COG0355	C	"F0F1-type ATP synthase, epsilon subunit (mitochondrial delta	K02114	METABOLISM
PA5554	atpD	ATP synthase beta chain	2.78E+08	2.85E+09	10.24906	COG0055	C	"F0F1-type ATP synthase, beta subunit"	K02112	METABOLISM
PA5554	atpD	ATP synthase beta chain	2.78E+08	2.85E+09	10.24906	COG0055	C	"F0F1-type ATP synthase, beta subunit"	K02133	METABOLISM

PA5555	atpG	ATP synthase gamma chain	57376199	74934264	1.306017	COG0224	C	"F0F1-type ATP synthase, gamma subunit"	K02115	METABOLISM
PA5556	atpA	ATP synthase alpha chain	9.36E+08	2.72E+09	2.904279	COG0056	C	"F0F1-type ATP synthase, alpha subunit"	K02111	METABOLISM
PA5557	atpH	ATP synthase delta chain	5.95E+09	4.14E+09	0.696811	COG0712	C	"F0F1-type ATP synthase, delta subunit (mitochondrial oligom	K02113	METABOLISM
PA5558	atpF	ATP synthase B chain	2.84E+10	2.24E+10	0.786812	COG0711	C	"F0F1-type ATP synthase, subunit b"	K02109	METABOLISM
PA5562	spoOJ	chromosome partitioning protein Spo0J	2.13E+08	98362748	0.462161	COG1475	K	Predicted transcriptional regulators	K03497	INFORMATION STORAGE AND PROCESSING
PA5568		conserved hypothetical protein	46712407	85006788	1.81979	COG0706	U	Preprotein translocase subunit YidC	K03217	CELLULAR PROCESSES AND SIGNALING
PA5570	rpmH	50S ribosomal protein L34	3.22E+09	2.96E+09	0.920184	COG0230	J	Ribosomal protein L34	K02914	INFORMATION STORAGE AND PROCESSING

Table S3. Differentially regulated proteins of biofilm cells grown with control (CB) and antibiotic-coated (AB) catheters

Locus Tag	Gene Name	Product Name	N_C-B	N_A-B	Fold Ratio	COG ID	CODE	COG Function	KOID	Functional Category
PA0015		hypothetical protein	70835633.9	65494903.27	0.924603899	COG3063	U	Tfp pilus assembly protein PilF	K02656	CELLULAR PROCESSES AND SIGNALING
PA0015		hypothetical protein	70835633.9	65494903.27	0.924603899	COG3063	N	Tfp pilus assembly protein PilF	K02656	CELLULAR PROCESSES AND SIGNALING
PA0039		hypothetical protein	419117259.3	981040359.6	2.340730041					
PA0044	exoT	exoenzyme T	0	58208225.1	1000					
PA0068		hypothetical protein	127040911.9	124516690.4	0.980130641	COG3529	R	Predicted nucleic-acid-binding protein containing a Zn-ribbon domain	K07070	POORLY CHARACTERIZED
PA0070	tagQ1	TagQ1	125004838.4	86603817.63	0.692803725					
PA0083	tssB1	TssB1	16375939.94	0	0	COG3516	S	Uncharacterized protein conserved in bacteria	K11901	POORLY CHARACTERIZED
PA0103		probable sulfate transporter	526721845	635674266.7	1.206850015	COG0659	P	Sulfate permease and related transporters (MFS superfamily)	K03321	METABOLISM
PA0128		conserved hypothetical protein	0	52707309.96	1000	COG2824	P	Uncharacterized Zn-ribbon-containing protein involved in phosphonate metaboli	K06193	METABOLISM
PA0139	ahpC	alkyl hydroperoxide reductase subunit C	3388114287	6743261155	1.990269685	COG0450	O	Peroxiredoxin	K03386	CELLULAR PROCESSES AND SIGNALING
PA0141		conserved hypothetical protein	1227474395	1635507451	1.332416756					
PA0141		conserved hypothetical protein	468973779.1	682089762.1	1.454430487					
PA0156	triA	"Resistance-Nodulation-Cell Division (RND) triclosan efflux membrane fusion protein	217121176.3	211368650.3	0.973505459	COG0845	M	Membrane-fusion protein	K02005	CELLULAR PROCESSES AND SIGNALING
PA0156	triA	"Resistance-Nodulation-Cell Division (RND) triclosan efflux membrane fusion protein	217121176.3	211368650.3	0.973505459	COG0845	M	Membrane-fusion protein	K01993	CELLULAR PROCESSES AND SIGNALING
PA0156	triA	"Resistance-Nodulation-Cell Division (RND) triclosan efflux membrane fusion protein	217121176.3	211368650.3	0.973505459	COG0845	M	Membrane-fusion protein	K02022	CELLULAR PROCESSES AND SIGNALING
PA0156	triA	"Resistance-Nodulation-Cell Division (RND) triclosan efflux membrane fusion protein	217121176.3	211368650.3	0.973505459	COG0845	M	Membrane-fusion protein	K03585	CELLULAR PROCESSES AND SIGNALING
PA0169	siaD	SiaD	1812886732	2168054978	1.195913093	COG3706	T	Response regulator containing a CheY-like receiver domain and a GGDEF domain	K02488	CELLULAR PROCESSES AND SIGNALING
PA0180	cttP	"chemotactic transducer for trichloroethylene [positive chemotaxis]	410781354.9	899531154.1	2.189805217	COG0840	T	Methyl-accepting chemotaxis protein	K03406	CELLULAR PROCESSES AND SIGNALING
PA0180	cttP	"chemotactic transducer for trichloroethylene [positive chemotaxis]	410781354.9	899531154.1	2.189805217	COG0840	N	Methyl-accepting chemotaxis protein	K03406	CELLULAR PROCESSES AND SIGNALING
PA0300	spuD	polyamine transport protein	72269979.17	0	0	COG0687	E	Spermidine/putrescine-binding periplasmic protein	K11069	METABOLISM
PA0300	spuD	polyamine transport protein	72269979.17	0	0	COG0687	E	Spermidine/putrescine-binding periplasmic protein	K02055	METABOLISM
PA0300	spuD	polyamine transport protein	72269979.17	0	0	COG0687	E	Spermidine/putrescine-binding periplasmic protein	K11073	METABOLISM
PA0301	spuE	polyamine transport protein	157780541.4	0	0	COG0687	E	Spermidine/putrescine-binding periplasmic protein	K11069	METABOLISM
PA0301	spuE	polyamine transport protein	157780541.4	0	0	COG0687	E	Spermidine/putrescine-binding periplasmic protein	K02055	METABOLISM
PA0301	spuE	polyamine transport protein	157780541.4	0	0	COG0687	E	Spermidine/putrescine-binding periplasmic protein	K11073	METABOLISM
PA0309		hypothetical protein	290627400.9	49617820.38	0.170726574					
PA0314		L-cysteine transporter of ABC system FliY	509111538.1	20652313.64	4.06E-02	COG0834	T	"ABC-type amino acid transport/signal transduction systems, periplasmic compon"	K02030	CELLULAR PROCESSES AND SIGNALING
PA0314		L-cysteine transporter of ABC system FliY	509111538.1	20652313.64	4.06E-02	COG0834	E	"ABC-type amino acid transport/signal transduction systems, periplasmic compon"	K02030	METABOLISM
PA0315		hypothetical protein	459817041.2	609666385.1	1.325889061					
PA0318		conserved hypothetical protein	27481655.78	0	0	COG0179	Q	"2-keto-4-pentenoate hydratase/2-oxohepta-3-ene-1,7-dioic acid hydratase (cate"	K05921	METABOLISM
PA0318		conserved hypothetical protein	27481655.78	0	0	COG0179	Q	"2-keto-4-pentenoate hydratase/2-oxohepta-3-ene-1,7-dioic acid hydratase (cate"	K01555	METABOLISM
PA0329		conserved hypothetical protein	3631135821	3738456426	1.029555657	COG3422	S	Uncharacterized conserved protein	K09946	POORLY CHARACTERIZED
PA0330	rpiA	ribose 5-phosphate isomerase	73732502.43	0	0	COG0120	G	Ribose 5-phosphate isomerase	K01807	METABOLISM
PA0355	pfpI	protease PfpI	14094120.16	0	0	COG0693	R	Putative intracellular protease/amidase	K05520	POORLY CHARACTERIZED
PA0355	pfpI	protease PfpI	14094120.16	0	0	COG0693	R	Putative intracellular protease/amidase	K03152	POORLY CHARACTERIZED
PA0359		hypothetical protein	62998650.72	0	0					
PA0371		hypothetical protein	267696234.9	68238141.28	0.254908857	COG0612	R	Predicted Zn-dependent peptidases	K07263	POORLY CHARACTERIZED
PA0371		hypothetical protein	267696234.9	68238141.28	0.254908857	COG0612	R	Predicted Zn-dependent peptidases	K01422	POORLY CHARACTERIZED
PA0371		hypothetical protein	267696234.9	68238141.28	0.254908857	COG0612	R	Predicted Zn-dependent peptidases	K01412	POORLY CHARACTERIZED
PA0371		hypothetical protein	267696234.9	68238141.28	0.254908857	COG0612	R	Predicted Zn-dependent peptidases	K01423	POORLY CHARACTERIZED
PA0372		probable zinc protease	44101805	0	0	COG0612	R	Predicted Zn-dependent peptidases	K01422	POORLY CHARACTERIZED
PA0372		probable zinc protease	44101805	0	0	COG0612	R	Predicted Zn-dependent peptidases	K01412	POORLY CHARACTERIZED
PA0372		probable zinc protease	44101805	0	0	COG0612	R	Predicted Zn-dependent peptidases	K01423	POORLY CHARACTERIZED
PA0372		probable zinc protease	44101805	0	0	COG0612	R	Predicted Zn-dependent peptidases	K07263	POORLY CHARACTERIZED
PA0373	ftsY	signal recognition particle receptor FtsY	17847770.48	0	0	COG0552	U	Signal recognition particle GTPase	K03110	CELLULAR PROCESSES AND SIGNALING
PA0375	ftsX	cell division protein FtsX	294609463.7	0	0	COG2177	D	Cell division protein	K09811	CELLULAR PROCESSES AND SIGNALING
PA0387		conserved hypothetical protein	153184966.8	0	0	COG0127	F	Xanthosine triphosphate pyrophosphatase	K01516	METABOLISM
PA0388		hypothetical protein	241382685.1	0	0					
PA0394		conserved hypothetical protein	13907206.13	39139431.44	2.814327412	COG0325	R	Predicted enzyme with a TIM-barrel fold	K06997	POORLY CHARACTERIZED
PA0399		cystathionine beta-synthase	0	14040161.44	1000	COG0517	R	FOG: CBS domain	K06041	POORLY CHARACTERIZED
PA0399		cystathionine beta-synthase	0	14040161.44	1000	COG0031	E	Cysteine synthase	K01697	METABOLISM
PA0399		cystathionine beta-synthase	0	14040161.44	1000	COG0031	E	Cysteine synthase	K12339	METABOLISM
PA0399		cystathionine beta-synthase	0	14040161.44	1000	COG0517	R	FOG: CBS domain	K00088	POORLY CHARACTERIZED
PA0399		cystathionine beta-synthase	0	14040161.44	1000	COG0031	E	Cysteine synthase	K01738	METABOLISM
PA0403	pyrR	transcriptional regulator PyrR	67239694.95	52021404.83	0.773671042	COG2065	F	Pyrimidine operon attenuation protein/uracil phosphoribosyltransferase	K02825	METABOLISM
PA0408	pilG	twitching motility protein PilG	345996731.7	267481962.5	0.773076558	COG0745	K	Response regulators consisting of a CheY-like receiver domain and a winged-he	K02483	INFORMATION STORAGE AND PROCESSING
PA0408	pilG	twitching motility protein PilG	345996731.7	267481962.5	0.773076558	COG0745	T	Response regulators consisting of a CheY-like receiver domain and a winged-he	K02483	CELLULAR PROCESSES AND SIGNALING
PA0409	pilH	twitching motility protein PilH	2574139142	1687949120	0.655733442	COG0745	T	Response regulators consisting of a CheY-like receiver domain and a winged-he	K02483	CELLULAR PROCESSES AND SIGNALING
PA0409	pilH	twitching motility protein PilH	2574139142	1687949120	0.655733442	COG0745	K	Response regulators consisting of a CheY-like receiver domain and a winged-he	K02483	INFORMATION STORAGE AND PROCESSING
PA0411	pilJ	twitching motility protein PilJ	3104945942	2856650942	0.920032424	COG0840	N	Methyl-accepting chemotaxis protein	K03406	CELLULAR PROCESSES AND SIGNALING
PA0411	pilJ	twitching motility protein PilJ	3104945942	2856650942	0.920032424	COG0840	T	Methyl-accepting chemotaxis protein	K03406	CELLULAR PROCESSES AND SIGNALING
PA0413	chpA	component of chemotactic signal transduction system	35193588.43	9563944.391	0.271752465	COG2198	T	FOG: HPt domain	K07676	CELLULAR PROCESSES AND SIGNALING
PA0413	chpA	component of chemotactic signal transduction system	35193588.43	9563944.391	0.271752465	COG0643	T	Chemotaxis protein histidine kinase and related kinases	K03407	CELLULAR PROCESSES AND SIGNALING
PA0413	chpA	component of chemotactic signal transduction system	35193588.43	9563944.391	0.271752465	COG0784	T	FOG: CheY-like receiver	K03413	CELLULAR PROCESSES AND SIGNALING
PA0413	chpA	component of chemotactic signal transduction system	35193588.43	9563944.391	0.271752465	COG0643	T	Chemotaxis protein histidine kinase and related kinases	K02487	CELLULAR PROCESSES AND SIGNALING
PA0413	chpA	component of chemotactic signal transduction system	35193588.43	9563944.391	0.271752465	COG0643	T	Chemotaxis protein histidine kinase and related kinases	K06596	CELLULAR PROCESSES AND SIGNALING
PA0413	chpA	component of chemotactic signal transduction system	35193588.43	9563944.391	0.271752465	COG0643	N	Chemotaxis protein histidine kinase and related kinases	K02487	CELLULAR PROCESSES AND SIGNALING
PA0413	chpA	component of chemotactic signal transduction system	35193588.43	9563944.391	0.271752465	COG0643	N	Chemotaxis protein histidine kinase and related kinases	K03407	CELLULAR PROCESSES AND SIGNALING
PA0413	chpA	component of chemotactic signal transduction system	35193588.43	9563944.391	0.271752465	COG2198	T	FOG: HPt domain	K07678	CELLULAR PROCESSES AND SIGNALING
PA0413	chpA	component of chemotactic signal transduction system	35193588.43	9563944.391	0.271752465	COG2198	T	FOG: HPt domain	K07679	CELLULAR PROCESSES AND SIGNALING
PA0413	chpA	component of chemotactic signal transduction system	35193588.43	9563944.391	0.271752465	COG2198	T	FOG: HPt domain	K07647	CELLULAR PROCESSES AND SIGNALING
PA0413	chpA	component of chemotactic signal transduction system	35193588.43	9563944.391	0.271752465	COG2198	T	FOG: HPt domain	K07648	CELLULAR PROCESSES AND SIGNALING
PA0413	chpA	component of chemotactic signal transduction system	35193588.43	9563944.391	0.271752465	COG0643	N	Chemotaxis protein histidine kinase and related kinases	K06596	CELLULAR PROCESSES AND SIGNALING
PA0423	pasP	PasP	4749966972	5497898223	1.157460305					
PA0427	oprM	Major intrinsic multiple antibiotic resistance efflux outer membrane protein OprM precursor	0	11134937.75	1000	COG1538	M	Outer membrane protein	K03287	CELLULAR PROCESSES AND SIGNALING
PA0427	oprM	Major intrinsic multiple antibiotic resistance efflux outer membrane protein OprM precursor	0	11134937.75	1000	COG1538	M	Outer membrane protein	K12340	CELLULAR PROCESSES AND SIGNALING
PA0427	oprM	Major intrinsic multiple antibiotic resistance efflux outer membrane protein OprM precursor	0	11134937.75	1000	COG1538	U	Outer membrane protein	K12340	CELLULAR PROCESSES AND SIGNALING
PA0427	oprM	Major intrinsic multiple antibiotic resistance efflux outer membrane protein OprM precursor	0	11134937.75	1000	COG1538	U	Outer membrane protein	K03287	CELLULAR PROCESSES AND SIGNALING
PA0429		hypothetical protein	0	261823938.5	1000					
PA0449		hypothetical protein	1722116875	769783110.3	0.446998181	COG1607	I	Acyl-CoA hydrolase	K01076	METABOLISM
PA0449		hypothetical protein	1722116875	769783110.3	0.446998181	COG1607	I	Acyl-CoA hydrolase	K10806	METABOLISM
PA0456		probable cold-shock protein	30940350815	19067531426	0.616267461	COG1278	K	Cold shock proteins	K03704	INFORMATION STORAGE AND PROCESSING
PA0459		probable ClpA/B protease ATP binding subunit	708897783.4	509231725.2	0.71834295	COG0542	O	"ATPases with chaperone activity, ATP-binding subunit"	K03694	CELLULAR PROCESSES AND SIGNALING
PA0459		probable ClpA/B protease ATP binding subunit	708897783.4	509231725.2	0.71834295	COG0542	O	"ATPases with chaperone activity, ATP-binding subunit"	K11907	CELLULAR PROCESSES AND SIGNALING
PA0459		probable ClpA/B protease ATP binding subunit	708897783.4	509231725.2	0.71834295	COG0542	O	"ATPases with chaperone activity, ATP-binding subunit"	K03696	CELLULAR PROCESSES AND SIGNALING
PA0459		probable ClpA/B protease ATP binding subunit	708897783.4	509231725.2	0.71834295	COG0542	O	"ATPases with chaperone activity, ATP-binding subunit"	K03695	CELLULAR PROCESSES AND SIGNALING
PA0460		hypothetical protein	306163295	253256865.9	0.827195389					
PA0468		hypothetical protein	60888972.87	56578997.22	0.929215826					
PA0541		hypothetical protein	669954856.7	1175636416	1.754799453					
PA0546	metK	methionine adenosyltransferase	20829087.61	0	0	COG0192	H	S-adenosylmethionine synthetase	K00789	METABOLISM
PA0552	pgk	phosphoglycerate kinase	304295008.7	0	0	COG0126	G	3-phosphoglycerate kinase	K00927	METABOLISM
PA0555	fda	"fructose-1	123325515.3	3168445646	25.69172842	COG0191	G	Fructose/tagatose bisphosphate aldolase	K01624	METABOLISM
PA0555	fda	"fructose-1	123325515.3	3168445646	25.69172842	COG0191	G	Fructose/tagatose bisphosphate aldolase	K08302	METABOLISM
PA0576	rpoD	sigma factor RpoD	952268468.6	319840056.6	0.335871729	COG0568	K	"DNA-directed RNA polymerase, sigma subunit (sigma70/sigma32)"	K03087	INFORMATION STORAGE AND PROCESSING
PA0576	rpoD	sigma factor RpoD	952268468.6	319840056.6	0.335871729	COG0568	K	"DNA-directed RNA polymerase, sigma subunit (sigma70/sigma32)"	K03089	INFORMATION STORAGE AND PROCESSING
PA0576	rpoD	sigma factor RpoD	952268468.6	319840056.6	0.335871729	COG0568	K	"DNA-directed RNA polymerase, sigma subunit (sigma70/sigma32)"	K03086	INFORMATION STORAGE AND PROCESSING
PA0579	rpsU	30S ribosomal protein S21	1902376624	0	0	COG0828	J	Ribosomal protein S21	K02970	INFORMATION STORAGE AND PROCESSING
PA0588		conserved hypothetical protein	15151819.58	0	0	COG2766	T	Putative Ser protein kinase	K07180	CELLULAR PROCESSES AND SIGNALING
PA0591		conserved hypothetical protein	50249303.97	0	0	COG2967	P	Uncharacterized protein affecting Mg2? transport	K06195	METABOLISM
PA0594	surA	peptidyl-prolyl cis-trans isomerase SurA	314725050.4	254149317.1	0.807528085	COG0760	O	Parvulin-like peptidyl-prolyl isomerase	K03771	CELLULAR PROCESSES AND SIGNALING
PA0594	surA	peptidyl-prolyl cis-trans isomerase SurA	314725050.4	254149317.1	0.807528085	COG0760	O	Parvulin-like peptidyl-prolyl isomerase	K07533	CELLULAR PROCESSES AND SIGNALING
PA0594	surA	peptidyl-prolyl cis-trans isomerase SurA	314725050.4	254149317.1	0.807528085	COG0760	O	Parvulin-like peptidyl-prolyl isomerase	K03770	CELLULAR PROCESSES AND SIGNALING
PA0594	surA	peptidyl-prolyl cis-trans isomerase SurA	314725050.4	254149317.1	0.807528085	COG0760	O	Parvulin-like peptidyl-prolyl isomerase	K03769	CELLULAR PROCESSES AND SIGNALING
PA0594	surA	peptidyl-prolyl cis-trans isomerase SurA	314725050.4	254149317.1	0.807528085	COG0760	O	Parvulin-like peptidyl-prolyl isomerase	K03771	CELLULAR PROCESSES AND SIGNALING
PA0594	surA	peptidyl-prolyl cis-trans isomerase SurA	314725050.4	254149317.1	0.807528085	COG0760	O	Parvulin-like peptidyl-prolyl isomerase	K03770	CELLULAR PROCESSES AND SIGNALING
PA0594	surA	peptidyl-prolyl cis-trans isomerase SurA	314725050.4	254149317.1	0.807528085	COG0760	O	Parvulin-like peptidyl-prolyl isomerase	K03769	CELLULAR PROCESSES AND SIGNALING
PA0594	surA	peptidyl-prolyl cis-trans isomerase SurA	314725050.4	254149317.1	0.807528085	COG0760	O	Parvulin-like peptidyl-prolyl isomerase	K07533	CELLULAR PROCESSES AND SIGNALING
PA0594	surA	peptidyl-prolyl cis-trans isomerase SurA	314725050.4	254149317.1	0.807528085	COG0760	O	Parvulin-like peptidyl-prolyl isomerase	K03769	CELLULAR PROCESSES AND SIGNALING
PA0594	surA	peptidyl-prolyl cis-trans isomerase SurA	314725050.4	254149317.1	0.807528085	COG0760	O	Parvulin-like peptidyl-prolyl isomerase	K03770	CELLULAR PROCESSES AND SIGNALING
PA0594	surA	peptidyl-prolyl cis-trans isomerase SurA	314725050.4	254149317.1	0.807528085	COG0760	O	Parvulin-like peptidyl-prolyl isomerase	K03771	CELLULAR PROCESSES AND SIGNALING
PA0594	surA	peptidyl-prolyl cis-trans isomerase SurA	314725050.4	254149317.1	0.807528085	COG0760	O	Parvulin-like peptidyl-prolyl isomerase	K03770	CELLULAR PROCESSES AND SIGNALING
PA0594	surA	peptidyl-prolyl cis-trans isomerase SurA	314725050.4	254149317.1	0.807528085	COG0760	O	Parvulin-like peptidyl-prolyl isomerase	K03769	CELLULAR PROCESSES AND SIGNALING
PA0594	surA	peptidyl-prolyl cis-trans isomerase SurA	314725050.4	254149317.1	0.807528085	COG0760	O	Parvulin-like peptidyl-prolyl isomerase	K03771	CELLULAR PROCESSES AND SIGNALING
PA0594	surA	peptidyl-prolyl cis-trans isomerase SurA	314725050.4	254149317.1	0.807528085	COG0760	O	Parvulin-like peptidyl-prolyl isomerase	K07533	CELLULAR PROCESSES AND SIGNALING
PA0594	surA	peptidyl-prolyl cis-trans isomerase SurA	314725050.4	254149317.1	0.807528085	COG0760	O	Parvulin-like peptidyl-prolyl isomerase	K07533	CELLULAR PROCESSES AND SIGNALING
PA0655		hypothetical protein	264209848.6	175250720.3	0.66330124	COG2941	H	Ubiquinone biosynthesis protein COQ7	K06134	METABOLISM
PA0659		hypothetical protein	91768894.7	0	0	COG4969	N	"Tfp pilus assembly protein, major pilin PilA"	K02655	CELLULAR PROCESSES AND SIGNALING
PA0659		hypothetical protein	91768894.7	0	0	COG4969	U	"Tfp pilus assembly protein, major pilin PilA"	K02655	CELLULAR PROCESSES AND SIGNALING
PA0659		hypothetical protein	91768894.7	0	0	COG4969	N	"Tfp pilus assembly protein, major pilin PilA"	K02650	CELLULAR PROCESSES AND SIGNALING
PA0659		hypothetical protein	91768894.7	0	0	COG4969	U	"Tfp pilus assembly protein, major pilin PilA"	K02682	CELLULAR PROCESSES AND SIGNALING
PA0659		hypothetical protein	91768894.7	0	0	COG4969	U	"Tfp pilus assembly protein, major pilin PilA"	K02650	CELLULAR PROCESSES AND SIGNALING
PA0659		hypothetical protein	91768894.7	0	0	COG4969	N	"Tfp pilus assembly protein, major pilin PilA"	K02682	CELLULAR PROCESSES AND SIGNALING
PA0664		hypothetical protein	428296539.7	252504789.5	0.589555988					
PA0665		conserved hypothetical protein	64869840.29	34136043.76	0.526223644	COG0316	S	Uncharacterized conserved protein	K13628	POORLY CHARACTERIZED
PA0665		conserved hypothetical protein	64869840.29	34136043.76	0.526223644	COG0316	S	Uncharacterized conserved protein	K07400	POORLY CHARACTERIZED
PA0668	tyrZ	tyrosyl-tRNA synthetase 2	804687507	1109936592	1.379338665	COG0162	J	Tyrosyl-tRNA synthetase	K01866	INFORMATION STORAGE AND PROCESSING
PA0672	hemO	heme oxygenase	761895295.6	804383022.2	1.055765834	COG3230	P	Heme oxygenase	K07215	METABOLISM
PA0720		helix destabilizing protein of bacteriophage Pf1	261473253.1	97745066.51	0.373824341					
PA0724		probable coat protein A of bacteriophage Pf1	0	27159949.7	1000					
PA0727		Pf replication initiator protein	0	979669123.1	1000					
PA0758		hypothetical protein	420711279.9	0	0					
PA0763	mucA	anti-sigma factor MucA	13078260.79	0	0	COG3073	T	Negative regulator of sigma E activity	K03597	CELLULAR PROCESSES AND SIGNALING
PA0766	mucD	serine protease MucD precursor	75599849.54	218022781	2.88390496	COG0265	O	"Trypsin-like serine proteases, typically periplasmic, contain C-terminal PDZ"	K04691	CELLULAR PROCESSES AND SIGNALING
PA0766	mucD	serine protease MucD precursor	75599849.54	218022781	2.88390496	COG0265	O	"Trypsin-like serine proteases, typically periplasmic, contain C-terminal PDZ"	K01362	CELLULAR PROCESSES AND SIGNALING
PA0778	icp	inhibitor of cysteine peptidase	76430161.09	34436013.7	0.450555294					
PA0797		probable transcriptional regulator	36871331.79	45827317.21	1.242898344					
PA0833		hypothetical protein	1866107277	2550486532	1.366741646	COG2885	M	Outer membrane protein and related peptidoglycan-associated (lipo)proteins	K03286	CELLULAR PROCESSES AND SIGNALING
PA0833		hypothetical protein	1866107277	2550486532	1.366741646	COG2885	M	Outer membrane protein and related peptidoglycan-associated (lipo)proteins	K03640	CELLULAR PROCESSES AND SIGNALING
PA0837	slyD	peptidyl-prolyl cis-trans isomerase SlyD	1512696317	2009478265	1.328408248	COG1047	O	FKBP-type peptidyl-prolyl cis-trans isomerases 2	K01802	CELLULAR PROCESSES AND SIGNALING
PA0837	slyD	peptidyl-prolyl cis-trans isomerase SlyD	1512696317	2009478265	1.328408248	COG1047	O	FKBP-type peptidyl-prolyl cis-trans isomerases 2	K03775	CELLULAR PROCESSES AND SIGNALING
PA0837	slyD	peptidyl-prolyl cis-trans isomerase SlyD	1512696317	2009478265	1.328408248	COG1047	O	FKBP-type peptidyl-prolyl cis-trans isomerases 2	K03774	CELLULAR PROCESSES AND SIGNALING
PA0854	fumC2	fumarate hydratase	211750877.6	173612790.7	0.819891718	COG0114	C	Fumarase	K01679	METABOLISM
PA0856		hypothetical protein	949487386	650428924.6	0.685031665	COG3184	S	Uncharacterized protein conserved in bacteria	K09924	POORLY CHARACTERIZED
PA0867	mliC	membrane-bound lysozyme inhibitor of c-type lysozyme MliC	53159663.79	218041905.7	4.101641924					

PA0871	phhB	pterin-4-alpha-carbinolamine dehydratase	257686045.3	0	0	COG2154	H	Pterin-4a-carbinolamine dehydratase	K01724	METABOLISM
PA0888	aotJ	arginine/ornithine binding protein AotJ	838527228.2	153987757	0.183640736	COG0834	T	"ABC-type amino acid transport/signal transduction systems, periplasmic compon"	K02030	CELLULAR PROCESSES AND SIGNALING
PA0888	aotJ	arginine/ornithine binding protein AotJ	838527228.2	153987757	0.183640736	COG0834	E	"ABC-type amino acid transport/signal transduction systems, periplasmic compon"	K02030	METABOLISM
PA0900		hypothetical protein	18364879.36	0	0					
PA0905	rsmA	RsmA	4748669931	3375936489	0.71092254	COG1551	T	Carbon storage regulator (could also regulate swarming and quorum sensing)	K03563	CELLULAR PROCESSES AND SIGNALING
PA0913	mgtE	MgtE	15791630.81	0	0	COG2239	P	Mg/Co/Ni transporter MgtE (contains CBS domain)	K06213	METABOLISM
PA0919		alanyl-phosphatidylglycerol hydrolase	22770551.38	0	0					
PA0937		conserved hypothetical protein	3665436124	2339752150	0.638328447	COG3122	S	Uncharacterized protein conserved in bacteria	K09912	POORLY CHARACTERIZED
PA0938	wzz2	Wzz2	199594122.6	0	0	COG3765	M	Chain length determinant protein	K05789	CELLULAR PROCESSES AND SIGNALING
PA0938	wzz2	Wzz2	199594122.6	0	0	COG3765	M	Chain length determinant protein	K05790	CELLULAR PROCESSES AND SIGNALING
PA0945	purM	phosphoribosylaminoimidazole synthetase	56484752.44	25497253.71	0.451400645	COG0150	F	Phosphoribosylaminoimidazole (AIR) synthetase	K01933	METABOLISM
PA0946		hypothetical protein	949045076.9	864039651.7	0.910430571	COG3249	S	Uncharacterized protein conserved in bacteria	K09938	POORLY CHARACTERIZED
PA0950		probable arsenate reductase	2104155822	877472797.1	0.417018924	COG1393	P	"Arsenate reductase and related proteins, glutaredoxin family"	K00537	METABOLISM
PA0953		probable thioredoxin	143178022	0	0	COG0526	O	Thiol-disulfide isomerase and thioredoxins	K03671	CELLULAR PROCESSES AND SIGNALING
PA0953		probable thioredoxin	143178022	0	0	COG0526	O	Thiol-disulfide isomerase and thioredoxins	K03673	CELLULAR PROCESSES AND SIGNALING
PA0953		probable thioredoxin	143178022	0	0	COG0526	C	Thiol-disulfide isomerase and thioredoxins	K03673	METABOLISM
PA0953		probable thioredoxin	143178022	0	0	COG0526	C	Thiol-disulfide isomerase and thioredoxins	K03671	METABOLISM
PA0960		hypothetical protein	163460541.9	0	0	COG2900	S	Uncharacterized protein conserved in bacteria	K03745	POORLY CHARACTERIZED
PA0962	dps	"DNA-binding protein from starved cells	196787423.6	706372410.1	3.589520088	COG0783	P	DNA-binding ferritin-like protein (oxidative damage protectant)	K04047	METABOLISM
PA0963	aspS	aspartyl-tRNA synthetase	8487442.272	43895250.7	5.171787835	COG0173	J	Aspartyl-tRNA synthetase	K01876	INFORMATION STORAGE AND PROCESSING
PA0964	pmpR	"pqsR-mediated PQS regulator	278483283.3	148020009.4	0.531522064					
PA0969	tolQ	TolQ protein	160231685.4	26418391.88	0.164876203	COG0811	U	Biopolymer transport proteins	K03561	CELLULAR PROCESSES AND SIGNALING
PA0969	tolQ	TolQ protein	160231685.4	26418391.88	0.164876203	COG0811	U	Biopolymer transport proteins	K03562	CELLULAR PROCESSES AND SIGNALING
PA0970	tolR	TolR protein	1018430398	690888051.6	0.678385143	COG0848	U	Biopolymer transport protein	K03559	CELLULAR PROCESSES AND SIGNALING
PA0970	tolR	TolR protein	1018430398	690888051.6	0.678385143	COG0848	U	Biopolymer transport protein	K03560	CELLULAR PROCESSES AND SIGNALING
PA0973	oprL	Peptidoglycan associated lipoprotein OprL precursor	28924693532	25722102074	0.889278292	COG2885	M	Outer membrane protein and related peptidoglycan-associated (lipo)proteins	K03286	CELLULAR PROCESSES AND SIGNALING
PA0973	oprL	Peptidoglycan associated lipoprotein OprL precursor	28924693532	25722102074	0.889278292	COG2885	M	Outer membrane protein and related peptidoglycan-associated (lipo)proteins	K03640	CELLULAR PROCESSES AND SIGNALING
PA0974		conserved hypothetical protein	942759676.5	669209605.5	0.709841142					
PA1006		Protein PA1006	180401749.5	0	0	COG0425	O	"Predicted redox protein, regulator of disulfide bond formation"	K04085	CELLULAR PROCESSES AND SIGNALING
PA1008	bcp	bacterioferritin comigratory protein	394289287.5	162228276.4	0.411444799	COG1225	O	Peroxiredoxin	K03564	CELLULAR PROCESSES AND SIGNALING
PA1010	dapA	dihydrodipicolinate synthase	1985790317	1542239463	0.776637618	COG0329	M	Dihydrodipicolinate synthase/N-acetylneuraminate lyase	K01639	CELLULAR PROCESSES AND SIGNALING
PA1010	dapA	dihydrodipicolinate synthase	1985790317	1542239463	0.776637618	COG0329	E	Dihydrodipicolinate synthase/N-acetylneuraminate lyase	K01714	METABOLISM
PA1010	dapA	dihydrodipicolinate synthase	1985790317	1542239463	0.776637618	COG0329	M	Dihydrodipicolinate synthase/N-acetylneuraminate lyase	K01714	CELLULAR PROCESSES AND SIGNALING
PA1010	dapA	dihydrodipicolinate synthase	1985790317	1542239463	0.776637618	COG0329	E	Dihydrodipicolinate synthase/N-acetylneuraminate lyase	K01639	METABOLISM
PA1011		hypothetical protein	10282218239	11279759064	1.097016111	COG3317	M	Uncharacterized lipoprotein	K07287	CELLULAR PROCESSES AND SIGNALING
PA1013	purC	phosphoribosylaminoimidazole-succinocarboxamide synthase	119648884.8	0	0	COG0152	F	Phosphoribosylaminoimidazolesuccinocarboxamide (SAICAR) synthase	K01923	METABOLISM
PA1041		probable outer membrane protein precursor	807330346.7	773226310.1	0.957757024	COG2885	M	Outer membrane protein and related peptidoglycan-associated (lipo)proteins	K03640	CELLULAR PROCESSES AND SIGNALING
PA1041		probable outer membrane protein precursor	807330346.7	773226310.1	0.957757024	COG2885	M	Outer membrane protein and related peptidoglycan-associated (lipo)proteins	K03286	CELLULAR PROCESSES AND SIGNALING
PA1049	pdxH	pyridoxine 5'-phosphate oxidase	0	19740164.05	1000	COG0259	H	Pyridoxamine-phosphate oxidase	K00275	METABOLISM
PA1053		conserved hypothetical protein	818598384.8	574546187.5	0.701865772	COG3133	M	Outer membrane lipoprotein	K06077	CELLULAR PROCESSES AND SIGNALING
PA1064		hypothetical protein	379549549.7	208033504.7	0.548106314	COG3650	S	Predicted membrane protein	K08985	POORLY CHARACTERIZED
PA1074	braC	branched-chain amino acid transport protein BraC	592147645.6	228153101.6	0.385297659	COG0683	E	"ABC-type branched-chain amino acid transport systems, periplasmic component"	K01999	METABOLISM
PA1076		hypothetical protein	1914991827	1909932614	0.997358102					
PA1086	flgK	flagellar hook-associated protein 1 FlgK	17998309.28	7202826.196	0.400194601	COG1256	N	Flagellar hook-associated protein	K02396	CELLULAR PROCESSES AND SIGNALING
PA1087	flgL	flagellar hook-associated protein type 3 FlgL	32846575.02	31276336.7	0.952194763	COG1344	N	Flagellin and related hook-associated proteins	K02406	CELLULAR PROCESSES AND SIGNALING
PA1087	flgL	flagellar hook-associated protein type 3 FlgL	32846575.02	31276336.7	0.952194763	COG1344	N	Flagellin and related hook-associated proteins	K02397	CELLULAR PROCESSES AND SIGNALING
PA1092	fliC	flagellin type B	29110582904	48659542166	1.67154132	COG1344	N	Flagellin and related hook-associated proteins	K02397	CELLULAR PROCESSES AND SIGNALING
PA1092	fliC	flagellin type B	29110582904	48659542166	1.67154132	COG1344	N	Flagellin and related hook-associated proteins	K02406	CELLULAR PROCESSES AND SIGNALING
PA1094	fliD	flagellar capping protein FliD	207716472	153140153	0.7372557	COG1345	N	Flagellar capping protein	K02407	CELLULAR PROCESSES AND SIGNALING
PA1096		hypothetical protein	18614809.63	24034410.18	1.29114456					
PA1097	fleQ	transcriptional regulator FleQ	222607004.7	162270637.5	0.728955667	COG2204	T	"Response regulator containing CheY-like receiver, AAA-type ATPase, and DNA-bi"	K07712	CELLULAR PROCESSES AND SIGNALING
PA1097	fleQ	transcriptional regulator FleQ	222607004.7	162270637.5	0.728955667	COG2204	T	"Response regulator containing CheY-like receiver, AAA-type ATPase, and DNA-bi"	K10126	CELLULAR PROCESSES AND SIGNALING
PA1097	fleQ	transcriptional regulator FleQ	222607004.7	162270637.5	0.728955667	COG2204	T	"Response regulator containing CheY-like receiver, AAA-type ATPase, and DNA-bi"	K02481	CELLULAR PROCESSES AND SIGNALING
PA1101	fliF	Flagella M-ring outer membrane protein precursor	15891534.61	0	0	COG1766	N	Flagellar biosynthesis/type III secretory pathway lipoprotein	K02409	CELLULAR PROCESSES AND SIGNALING
PA1101	fliF	Flagella M-ring outer membrane protein precursor	15891534.61	0	0	COG1766	U	Flagellar biosynthesis/type III secretory pathway lipoprotein	K02409	CELLULAR PROCESSES AND SIGNALING
PA1103		probable flagellar assembly protein	92415878.51	70885277.99	0.767024879	COG1317	U	Flagellar biosynthesis/type III secretory pathway protein	K02411	CELLULAR PROCESSES AND SIGNALING
PA1103		probable flagellar assembly protein	92415878.51	70885277.99	0.767024879	COG1317	N	Flagellar biosynthesis/type III secretory pathway protein	K02411	CELLULAR PROCESSES AND SIGNALING
PA1103		probable flagellar assembly protein	92415878.51	70885277.99	0.767024879	COG1317	U	Flagellar biosynthesis/type III secretory pathway protein	K03223	CELLULAR PROCESSES AND SIGNALING
PA1103		probable flagellar assembly protein	92415878.51	70885277.99	0.767024879	COG1317	N	Flagellar biosynthesis/type III secretory pathway protein	K03223	CELLULAR PROCESSES AND SIGNALING
PA1119	yfiB	YfiB	969314447.1	505641839.5	0.521648925	COG2885	M	Outer membrane protein and related peptidoglycan-associated (lipo)proteins	K03640	CELLULAR PROCESSES AND SIGNALING
PA1119	yfiB	YfiB	969314447.1	505641839.5	0.521648925	COG2885	M	Outer membrane protein and related peptidoglycan-associated (lipo)proteins	K03286	CELLULAR PROCESSES AND SIGNALING
PA1123		hypothetical protein	324917030.7	0	0					
PA1127		probable oxidoreductase	84128047.44	0	0					
PA1139		hypothetical protein	18272916.64	0	0					
PA1151	imm2	pyocin S2 immunity protein	3338854030	2539104503	0.760471851					
PA1155	nrdB	"NrdB	47282195.38	160006949.8	3.384084611	COG0208	F	"Ribonucleotide reductase, beta subunit"	K10808	METABOLISM
PA1155	nrdB	"NrdB	47282195.38	160006949.8	3.384084611	COG0208	F	"Ribonucleotide reductase, beta subunit"	K00526	METABOLISM
PA1156	nrdA	"NrdA	239516191.8	20095308.57	8.39E-02	COG0209	F	"Ribonucleotide reductase, alpha subunit"	K00525	METABOLISM
PA1159		probable cold-shock protein	15293315803	12910210918	0.844173434	COG1278	K	Cold shock proteins	K03704	INFORMATION STORAGE AND PROCESSING
PA1203		hypothetical protein	44189156.78	128736541.7	2.913306139	COG1765	O	"Predicted redox protein, regulator of disulfide bond formation"	K06889	CELLULAR PROCESSES AND SIGNALING
PA1203		hypothetical protein	44189156.78	128736541.7	2.913306139	COG1765	O	"Predicted redox protein, regulator of disulfide bond formation"	K07397	CELLULAR PROCESSES AND SIGNALING
PA1244	qslA	QslA	748778951.9	58607930.02	7.83E-02					
PA1272	cobO	cob(I)alamin adenosyltransferase	11527275.7	20036595.94	1.738190051	COG2109	H	ATP:corrinoid adenosyltransferase	K00798	METABOLISM
PA1295		conserved hypothetical protein	55172170.26	31850936.43	0.577300771	COG3100	S	Uncharacterized protein conserved in bacteria	K09902	POORLY CHARACTERIZED
PA1323		hypothetical protein	417437338.6	214890643.5	0.514785391	COG4575	S	Uncharacterized conserved protein	K05594	POORLY CHARACTERIZED
PA1324		hypothetical protein	1087867806	239233916.6	0.219910834					
PA1338	ggt	gamma-glutamyltranspeptidase precursor	20221126.05	0	0	COG0405	E	Gamma-glutamyltransferase	K00681	METABOLISM
PA1342	aatJ	AatJ	895641801.5	91800555.41	0.102496953	COG0834	T	"ABC-type amino acid transport/signal transduction systems, periplasmic compon"	K02030	CELLULAR PROCESSES AND SIGNALING
PA1342	aatJ	AatJ	895641801.5	91800555.41	0.102496953	COG0834	E	"ABC-type amino acid transport/signal transduction systems, periplasmic compon"	K02030	METABOLISM
PA1369		hypothetical protein	20903972764	25944272989	1.241116858					
PA1423	bdlA	BdlA	410781354.9	899531154.1	2.189805217	COG0840	N	Methyl-accepting chemotaxis protein	K03406	CELLULAR PROCESSES AND SIGNALING
PA1423	bdlA	BdlA	410781354.9	899531154.1	2.189805217	COG0840	T	Methyl-accepting chemotaxis protein	K03406	CELLULAR PROCESSES AND SIGNALING
PA1444	fliN	flagellar motor switch protein FliN	0	57258208.72	1000	COG1886	N	Flagellar motor switch/type III secretory pathway protein	K02417	CELLULAR PROCESSES AND SIGNALING
PA1444	fliN	flagellar motor switch protein FliN	0	57258208.72	1000	COG1886	U	Flagellar motor switch/type III secretory pathway protein	K02417	CELLULAR PROCESSES AND SIGNALING
PA1444	fliN	flagellar motor switch protein FliN	0	57258208.72	1000	COG1886	U	Flagellar motor switch/type III secretory pathway protein	K03225	CELLULAR PROCESSES AND SIGNALING
PA1444	fliN	flagellar motor switch protein FliN	0	57258208.72	1000	COG1886	N	Flagellar motor switch/type III secretory pathway protein	K03225	CELLULAR PROCESSES AND SIGNALING
PA1457	cheZ	chemotaxis protein CheZ	1137381664	476975920.2	0.419363117	COG3143	T	Chemotaxis protein	K03414	CELLULAR PROCESSES AND SIGNALING
PA1457	cheZ	chemotaxis protein CheZ	834184401.7	460449269	0.5519754	COG3143	T	Chemotaxis protein	K03414	CELLULAR PROCESSES AND SIGNALING
PA1457	cheZ	chemotaxis protein CheZ	834184401.7	460449269	0.5519754	COG3143	T	Chemotaxis protein	K03414	CELLULAR PROCESSES AND SIGNALING
PA1457	cheZ	chemotaxis protein CheZ	834184401.7	460449269	0.5519754	COG3143	N	Chemotaxis protein	K03414	CELLULAR PROCESSES AND SIGNALING
PA1457	cheZ	chemotaxis protein CheZ	1137381664	476975920.2	0.419363117	COG3143	T	Chemotaxis protein	K03414	CELLULAR PROCESSES AND SIGNALING
PA1457	cheZ	chemotaxis protein CheZ	1137381664	476975920.2	0.419363117	COG3143	N	Chemotaxis protein	K03414	CELLULAR PROCESSES AND SIGNALING
PA1457	cheZ	chemotaxis protein CheZ	834184401.7	460449269	0.5519754	COG3143	N	Chemotaxis protein	K03414	CELLULAR PROCESSES AND SIGNALING
PA1457	cheZ	chemotaxis protein CheZ	1137381664	476975920.2	0.419363117	COG3143	N	Chemotaxis protein	K03414	CELLULAR PROCESSES AND SIGNALING
PA1458		probable two-component sensor	47794573.77	0	0	COG2198	T	FOG: HPt domain	K07647	CELLULAR PROCESSES AND SIGNALING
PA1458		probable two-component sensor	47794573.77	0	0	COG2198	T	FOG: HPt domain	K07676	CELLULAR PROCESSES AND SIGNALING
PA1458		probable two-component sensor	47794573.77	0	0	COG0643	T	Chemotaxis protein histidine kinase and related kinases	K03407	CELLULAR PROCESSES AND SIGNALING
PA1458		probable two-component sensor	47794573.77	0	0	COG0643	T	Chemotaxis protein histidine kinase and related kinases	K02487	CELLULAR PROCESSES AND SIGNALING
PA1458		probable two-component sensor	47794573.77	0	0	COG0643	T	Chemotaxis protein histidine kinase and related kinases	K06596	CELLULAR PROCESSES AND SIGNALING
PA1458		probable two-component sensor	47794573.77	0	0	COG0643	N	Chemotaxis protein histidine kinase and related kinases	K03407	CELLULAR PROCESSES AND SIGNALING
PA1458		probable two-component sensor	47794573.77	0	0	COG2198	T	FOG: HPt domain	K07679	CELLULAR PROCESSES AND SIGNALING
PA1458		probable two-component sensor	47794573.77	0	0	COG2198	T	FOG: HPt domain	K07648	CELLULAR PROCESSES AND SIGNALING
PA1458		probable two-component sensor	47794573.77	0	0	COG0643	N	Chemotaxis protein histidine kinase and related kinases	K06596	CELLULAR PROCESSES AND SIGNALING
PA1458		probable two-component sensor	47794573.77	0	0	COG0643	N	Chemotaxis protein histidine kinase and related kinases	K02487	CELLULAR PROCESSES AND SIGNALING
PA1458		probable two-component sensor	47794573.77	0	0	COG2198	T	FOG: HPt domain	K07678	CELLULAR PROCESSES AND SIGNALING
PA1479	ccmE	cytochrome C-type biogenesis protein CcmE	673192337.4	109014068	0.161935991	COG2332	O	Cytochrome c-type biogenesis protein CcmE	K02197	CELLULAR PROCESSES AND SIGNALING
PA1482	ccmH	cytochrome C-type biogenesis protein CcmH	22443088.94	54615566.3	2.433513785	COG3088	O	Uncharacterized protein involved in biosynthesis of c-type cytochromes	K02200	CELLULAR PROCESSES AND SIGNALING
PA1482	ccmH	cytochrome C-type biogenesis protein CcmH	22443088.94	54615566.3	2.433513785	COG3088	O	Uncharacterized protein involved in biosynthesis of c-type cytochromes	K04017	CELLULAR PROCESSES AND SIGNALING
PA1483	cycH	cytochrome c-type biogenesis protein	0	40577699.81	1000	COG4235	O	Cytochrome c biogenesis factor	K02200	CELLULAR PROCESSES AND SIGNALING
PA1483	cycH	cytochrome c-type biogenesis protein	0	40577699.81	1000	COG4235	O	Cytochrome c biogenesis factor	K04018	CELLULAR PROCESSES AND SIGNALING
PA1516		hypothetical protein	11428055.01	0	0	COG3195	S	Uncharacterized protein conserved in bacteria	K06016	POORLY CHARACTERIZED
PA1516		hypothetical protein	11428055.01	0	0	COG3195	S	Uncharacterized protein conserved in bacteria	K13485	POORLY CHARACTERIZED
PA1528	zipA	cell division protein ZipA	1989656679	2018861768	1.014678456	COG3115	D	Cell division protein	K03528	CELLULAR PROCESSES AND SIGNALING
PA1532	dnaX	DNA polymerase subunits gamma and tau	52340879.6	0	0	COG2812	L	"DNA polymerase III, gamma/tau subunits"	K02343	INFORMATION STORAGE AND PROCESSING
PA1532	dnaX	DNA polymerase subunits gamma and tau	52340879.6	0	0	COG2812	L	"DNA polymerase III, gamma/tau subunits"	K02341	INFORMATION STORAGE AND PROCESSING
PA1533		conserved hypothetical protein	219159726	38951398.01	0.177730638	COG0718	S	Uncharacterized protein conserved in bacteria	K09747	POORLY CHARACTERIZED
PA1550		hypothetical protein	573145004.3	960443124.9	1.675741946	COG3198	S	Uncharacterized protein conserved in bacteria	K09926	POORLY CHARACTERIZED
PA1551		probable ferredoxin	21281728.66	0	0	COG0348	C	Polyferredoxin	K02574	METABOLISM
PA1552	ccoP1	"Cytochrome c oxidase	114454741.3	324759104.7	2.837445622	COG2010	C	"Cytochrome c, mono- and diheme variants"	K02275	METABOLISM
PA1552	ccoP1	"Cytochrome c oxidase	114454741.3	324759104.7	2.837445622	COG2010	C	"Cytochrome c, mono- and diheme variants"	K00406	METABOLISM
PA1552	ccoP1	"Cytochrome c oxidase	114454741.3	324759104.7	2.837445622	COG2010	C	"Cytochrome c, mono- and diheme variants"	K00406	METABOLISM
PA1552	ccoP1	"Cytochrome c oxidase	114454741.3	324759104.7	2.837445622	COG2010	C	"Cytochrome c, mono- and diheme variants"	K02275	METABOLISM
PA1552	ccoP1	"Cytochrome c oxidase	0	43309845.54	1000	COG2010	C	"Cytochrome c, mono- and diheme variants"	K00406	METABOLISM
PA1552	ccoP1	"Cytochrome c oxidase	0	43309845.54	1000	COG2010	C	"Cytochrome c, mono- and diheme variants"	K02275	METABOLISM
PA1552	ccoP1	"Cytochrome c oxidase	0	43309845.54	1000	COG2010	C	"Cytochrome c, mono- and diheme variants"	K02275	METABOLISM
PA1552	ccoP1	"Cytochrome c oxidase	0	43309845.54	1000	COG2010	C	"Cytochrome c, mono- and diheme variants"	K00406	METABOLISM
PA1553	ccoO1	"Cytochrome c oxidase	291321006.4	1384235532	4.751581592	COG2993	C	"Cbb3-type cytochrome oxidase, cytochrome c subunit"	K00405	METABOLISM
PA1555	ccoP2	"Cytochrome c oxidase	0	43309845.54	1000	COG2010	C	"Cytochrome c, mono- and diheme variants"	K02275	METABOLISM
PA1555	ccoP2	"Cytochrome c oxidase	0	140808013	1000	COG2010	C	"Cytochrome c, mono- and diheme variants"	K00406	METABOLISM
PA1555	ccoP2	"Cytochrome c oxidase	0	140808013	1000	COG2010	C	"Cytochrome c, mono- and diheme variants"	K02275	METABOLISM
PA1555	ccoP2	"Cytochrome c oxidase	0	140808013	1000	COG2010	C	"Cytochrome c, mono- and diheme variants"	K00406	METABOLISM
PA1555	ccoP2	"Cytochrome c oxidase	0	43309845.54	1000	COG2010	C	"Cytochrome c, mono- and diheme variants"	K00406	METABOLISM
PA1555	ccoP2	"Cytochrome c oxidase	0	43309845.54	1000	COG2010	C	"Cytochrome c, mono- and diheme variants"	K00406	METABOLISM
PA1555	ccoP2	"Cytochrome c oxidase	0	43309845.54	1000	COG2010	C	"Cytochrome c, mono- and diheme variants"	K02275	METABOLISM
PA1555	ccoP2	"Cytochrome c oxidase	0	140808013	1000	COG2010	C	"Cytochrome c, mono- and diheme variants"	K02275	METABOLISM
PA1556	ccoO2	"Cytochrome c oxidase	25182502.26	895676105.3	35.56739898	COG2993	C	"Cbb3-type cytochrome oxidase, cytochrome c subunit"	K00405	METABOLISM
PA1561	aer	aerotaxis receptor Aer	968672330.4	1308582290	1.350902931	COG0840	T	Methyl-accepting chemotaxis protein	K03406	CELLULAR PROCESSES AND SIGNALING
PA1561	aer	aerotaxis receptor Aer	968672330.4	1308582290	1.350902931	COG0840	N	Methyl-accepting chemotaxis protein	K03406	CELLULAR PROCESSES AND SIGNALING
PA1571		hypothetical protein	213603025.6	43961421.94	0.205808985					
PA1579		hypothetical protein	69741900.8	0	0					
PA1586	sucB	dihydrolipoamide succinyltransferase (E2 subunit)	1246511618	2139969492	1.716766583	COG0508	C	"Pyruvate/2-oxoglutarate dehydrogenase complex, dihydrolipoamide acyltransfera"	K00627	METABOLISM
PA1586	sucB	dihydrolipoamide succinyltransferase (E2 subunit)	1246511618	2139969492	1.716766583	COG0508	C	"Pyruvate/2-oxoglutarate dehydrogenase complex, dihydrolipoamide acyltransfera"	K00658	METABOLISM
PA1586	sucB	dihydrolipoamide succinyltransferase (E2 subunit)	1246511618	2139969492	1.716766583	COG0508	C	"Pyruvate/2-oxoglutarate dehydrogenase complex, dihydrolipoamide acyltransfera"	K09699	METABOLISM
PA1588	sucC	succinyl-CoA synthetase beta chain	558197091	1929493293	3.456652362	COG0045	C	"Succinyl-CoA synthetase, beta subunit"	K01903	METABOLISM
PA1588	sucC	succinyl-CoA synthetase beta chain	558197091	1929493293	3.456652362	COG0045	C	"Succinyl-CoA synthetase, beta subunit"	K01900	METABOLISM
PA1589	sucD	succinyl-CoA synthetase alpha chain	638943779.1	854296891.9	1.33704548	COG0074	C	"Succinyl-CoA synthetase, alpha subunit"	K01902	METABOLISM

PA1596	htpG	heat shock protein HtpG	26337526.76	66200937.07	2.513559366	COG0326	O	"Molecular chaperone, HSP90 family"	K04079	CELLULAR PROCESSES AND SIGNALING
PA1608		probable chemotaxis transducer	410781354.9	899531154.1	2.189805217	COG0840	N	Methyl-accepting chemotaxis protein	K03406	CELLULAR PROCESSES AND SIGNALING
PA1608		probable chemotaxis transducer	410781354.9	899531154.1	2.189805217	COG0840	T	Methyl-accepting chemotaxis protein	K03406	CELLULAR PROCESSES AND SIGNALING
PA1615		probable lipase	333550597.2	456489799.7	1.368577372					
PA1641		hypothetical protein	0	18936738.03	1000					
PA1646		probable chemotaxis transducer	410781354.9	899531154.1	2.189805217	COG0840	N	Methyl-accepting chemotaxis protein	K03406	CELLULAR PROCESSES AND SIGNALING
PA1646		probable chemotaxis transducer	410781354.9	899531154.1	2.189805217	COG0840	T	Methyl-accepting chemotaxis protein	K03406	CELLULAR PROCESSES AND SIGNALING
PA1657	hsiB2	HsiB2	145557781.6	0	0	COG3516	S	Uncharacterized protein conserved in bacteria	K11901	POORLY CHARACTERIZED
PA1673		hypothetical protein	11695062.85	0	0	COG2703	P	Hemerythrin	K07216	METABOLISM
PA1707	pcrH	regulatory protein PcrH	154884185.2	392990265.4	2.53731693					
PA1708	popB	translocator protein PopB	38269643.39	0	0					
PA1709	popD	Translocator outer membrane protein PopD precursor	39813370.51	16160510.01	0.405906604					
PA1722	pscI	type III export protein PscI	0	43171956.9	1000					
PA1746		hypothetical protein	187433183.6	547179691.7	2.919332006					
PA1749		hypothetical protein	912328297.1	615681274.9	0.67484619	COG2388	R	Predicted acetyltransferase	K06975	POORLY CHARACTERIZED
PA1750		phospho-2-dehydro-3-deoxyheptonate aldolase	51681429.16	0	0	COG0722	E	3-deoxy-D-arabino-heptulosonate 7-phosphate (DAHP) synthase	K01626	METABOLISM
PA1772		probable methyltransferase	489430404.9	133779708.7	0.273337552	COG0684	H	Demethylmenaquinone methyltransferase	K02553	METABOLISM
PA1777	oprF	Major porin and structural outer membrane porin OprF precursor	1.09255E+11	1.4122E+11	1.292568171	COG2885	M	Outer membrane protein and related peptidoglycan-associated (lipo)proteins	K03286	CELLULAR PROCESSES AND SIGNALING
PA1777	oprF	Major porin and structural outer membrane porin OprF precursor	1.09255E+11	1.4122E+11	1.292568171	COG2885	M	Outer membrane protein and related peptidoglycan-associated (lipo)proteins	K03640	CELLULAR PROCESSES AND SIGNALING
PA1777	oprF	Major porin and structural outer membrane porin OprF precursor	1.09255E+11	1.4122E+11	1.292568171	COG3047	M	Outer membrane protein W	K07275	CELLULAR PROCESSES AND SIGNALING
PA1787	acnB	aconitate hydratase 2	1262463778	1419968005	1.124759402	COG1049	C	Aconitase B	K01682	METABOLISM
PA1793	ppiB	peptidyl-prolyl cis-trans isomerase B	22223001730	13013589146	0.58559097	COG0652	O	Peptidyl-prolyl cis-trans isomerase (rotamase) - cyclophilin family	K03768	CELLULAR PROCESSES AND SIGNALING
PA1793	ppiB	peptidyl-prolyl cis-trans isomerase B	22223001730	13013589146	0.58559097	COG0652	O	Peptidyl-prolyl cis-trans isomerase (rotamase) - cyclophilin family	K01802	CELLULAR PROCESSES AND SIGNALING
PA1793	ppiB	peptidyl-prolyl cis-trans isomerase B	22223001730	13013589146	0.58559097	COG0652	O	Peptidyl-prolyl cis-trans isomerase (rotamase) - cyclophilin family	K03767	CELLULAR PROCESSES AND SIGNALING
PA1800	tig	trigger factor	11935591909	9277449670	0.777292801	COG0544	O	FKBP-type peptidyl-prolyl cis-trans isomerase (trigger factor)	K03545	CELLULAR PROCESSES AND SIGNALING
PA1801	clpP	ClpP	0	28781910.22	1000	COG0740	O	Protease subunit of ATP-dependent Clp proteases	K01358	CELLULAR PROCESSES AND SIGNALING
PA1801	clpP	ClpP	0	28781910.22	1000	COG0740	U	Protease subunit of ATP-dependent Clp proteases	K01358	CELLULAR PROCESSES AND SIGNALING
PA1802	clpX	ClpX	17792131752	1053963559	5.92E-02	COG1219	O	"ATP-dependent protease Clp, ATPase subunit"	K03544	CELLULAR PROCESSES AND SIGNALING
PA1804	hupB	DNA-binding protein HU	336645224	3431277453	10.1925624	COG0776	L	Bacterial nucleoid DNA-binding protein	K05788	INFORMATION STORAGE AND PROCESSING
PA1804	hupB	DNA-binding protein HU	336645224	3431277453	10.1925624	COG0776	L	Bacterial nucleoid DNA-binding protein	K04764	INFORMATION STORAGE AND PROCESSING
PA1804	hupB	DNA-binding protein HU	336645224	3431277453	10.1925624	COG0776	L	Bacterial nucleoid DNA-binding protein	K05787	INFORMATION STORAGE AND PROCESSING
PA1804	hupB	DNA-binding protein HU	336645224	3431277453	10.1925624	COG0776	L	Bacterial nucleoid DNA-binding protein	K03530	INFORMATION STORAGE AND PROCESSING
PA1805	ppiD	peptidyl-prolyl cis-trans isomerase D	5056673334	3700691004	0.731843004	COG0760	O	Parvulin-like peptidyl-prolyl isomerase	K07533	CELLULAR PROCESSES AND SIGNALING
PA1805	ppiD	peptidyl-prolyl cis-trans isomerase D	5056673334	3700691004	0.731843004	COG0760	O	Parvulin-like peptidyl-prolyl isomerase	K03769	CELLULAR PROCESSES AND SIGNALING
PA1805	ppiD	peptidyl-prolyl cis-trans isomerase D	5056673334	3700691004	0.731843004	COG0760	O	Parvulin-like peptidyl-prolyl isomerase	K03770	CELLULAR PROCESSES AND SIGNALING
PA1805	ppiD	peptidyl-prolyl cis-trans isomerase D	5056673334	3700691004	0.731843004	COG0760	O	Parvulin-like peptidyl-prolyl isomerase	K03771	CELLULAR PROCESSES AND SIGNALING
PA1830		hypothetical protein	543638717	514202505.4	0.945853357					
PA1847	nfuA	NfuA	395844115	0	0	COG0316	S	Uncharacterized conserved protein	K13628	POORLY CHARACTERIZED
PA1847	nfuA	NfuA	395844115	0	0	COG0316	S	Uncharacterized conserved protein	K07400	POORLY CHARACTERIZED
PA1847	nfuA	NfuA	395844115	0	0	COG0694	O	Thioredoxin-like proteins and domains	K07400	CELLULAR PROCESSES AND SIGNALING
PA1847	nfuA	NfuA	395844115	0	0	COG0694	O	Thioredoxin-like proteins and domains	K13819	CELLULAR PROCESSES AND SIGNALING
PA1870		hypothetical protein	105119115.8	0	0					
PA1930		probable chemotaxis transducer	410781354.9	899531154.1	2.189805217	COG2202	T	FOG: PAS/PAC domain	K00936	CELLULAR PROCESSES AND SIGNALING
PA1930		probable chemotaxis transducer	410781354.9	899531154.1	2.189805217	COG0840	N	Methyl-accepting chemotaxis protein	K03406	CELLULAR PROCESSES AND SIGNALING
PA1930		probable chemotaxis transducer	410781354.9	899531154.1	2.189805217	COG0840	T	Methyl-accepting chemotaxis protein	K03406	CELLULAR PROCESSES AND SIGNALING
PA1969		hypothetical protein	5302960100	3888356292	0.733242608					
PA2021		hypothetical protein	196455179.5	217132529.2	1.10525225					
PA2081	kynB	"kynurenine formamidase	0	142184986.9	1000	COG1878	R	Predicted metal-dependent hydrolase	K07130	POORLY CHARACTERIZED
PA2169		hypothetical protein	202028103.5	0	0					
PA2190		conserved hypothetical protein	108970364.5	20501611.5	0.188139331	COG3729	R	General stress protein	K06884	POORLY CHARACTERIZED
PA2204		probable binding protein component of ABC transporter	7061994.416	0	0	COG0834	E	"ABC-type amino acid transport/signal transduction systems, periplasmic compon"	K02030	METABOLISM
PA2204		probable binding protein component of ABC transporter	7061994.416	0	0	COG0834	T	"ABC-type amino acid transport/signal transduction systems, periplasmic compon"	K02030	CELLULAR PROCESSES AND SIGNALING
PA2234	psID	PsID	45178375.13	0	0	COG1596	M	Periplasmic protein involved in polysaccharide export	K01991	CELLULAR PROCESSES AND SIGNALING
PA2247	bkdA1	2-oxoisovalerate dehydrogenase (alpha subunit)	14894375.18	0	0	COG1071	C	"Pyruvate/2-oxoglutarate dehydrogenase complex, dehydrogenase (E1) component,"	K11381	METABOLISM
PA2247	bkdA1	2-oxoisovalerate dehydrogenase (alpha subunit)	14894375.18	0	0	COG1071	C	"Pyruvate/2-oxoglutarate dehydrogenase complex, dehydrogenase (E1) component,"	K00166	METABOLISM
PA2247	bkdA1	2-oxoisovalerate dehydrogenase (alpha subunit)	14894375.18	0	0	COG1071	C	"Pyruvate/2-oxoglutarate dehydrogenase complex, dehydrogenase (E1) component,"	K00161	METABOLISM
PA2264		conserved hypothetical protein	153696610.9	99523657.81	0.647533197					
PA2331		hypothetical protein	1623568184	1533859046	0.944745691	COG2128	S	Uncharacterized conserved protein	K04756	POORLY CHARACTERIZED
PA2381		hypothetical protein	324807563.5	203305703	0.625926628					
PA2385	pvdQ	3-oxo-C12-homoserine lactone acylase PvdQ	620406501.7	594553427.1	0.958328814	COG2366	R	Protein related to penicillin acylase	K07116	POORLY CHARACTERIZED
PA2385	pvdQ	3-oxo-C12-homoserine lactone acylase PvdQ	620406501.7	594553427.1	0.958328814	COG2366	R	Protein related to penicillin acylase	K01434	POORLY CHARACTERIZED
PA2393		putative dipeptidase	450245318.3	1803200880	4.004929772	COG2355	E	"Zn-dependent dipeptidase, microsomal dipeptidase homolog"	K01273	METABOLISM
PA2395	pvdO	PvdO	1045614477	617124133.2	0.590202361					
PA2396	pvdF	pyoverdine synthetase F	38175033.64	198210612.7	5.192152929	COG0299	F	Folate-dependent phosphoribosylglycinamide formyltransferase PurN	K11175	METABOLISM
PA2410	fpvF	FpvF	231531573	148413020.7	0.641005539	COG0803	P	"ABC-type metal ion transport system, periplasmic component/surface adhesin"	K09815	METABOLISM
PA2410	fpvF	FpvF	231531573	148413020.7	0.641005539	COG0803	P	"ABC-type metal ion transport system, periplasmic component/surface adhesin"	K09818	METABOLISM
PA2410	fpvF	FpvF	231531573	148413020.7	0.641005539	COG0803	P	"ABC-type metal ion transport system, periplasmic component/surface adhesin"	K02077	METABOLISM
PA2412		conserved hypothetical protein	1228116512	2076199344	1.69055568	COG3251	S	Uncharacterized protein conserved in bacteria	K05375	POORLY CHARACTERIZED
PA2413	pvdH	"L-2	16904917.73	19946518.89	1.179924044	COG0160	E	4-aminobutyrate aminotransferase and related aminotransferases	K00823	METABOLISM
PA2413	pvdH	"L-2	16904917.73	19946518.89	1.179924044	COG0160	E	4-aminobutyrate aminotransferase and related aminotransferases	K07250	METABOLISM
PA2413	pvdH	"L-2	16904917.73	19946518.89	1.179924044	COG0160	E	4-aminobutyrate aminotransferase and related aminotransferases	K00836	METABOLISM
PA2433		hypothetical protein	218192623.1	54455110.59	0.249573564					
PA2446	gcvH2	glycine cleavage system protein H2	242156555.2	113486555.7	0.46864953	COG0509	E	Glycine cleavage system H protein (lipoate-binding)	K02437	METABOLISM
PA2453		hypothetical protein	45577819.54	143732839.4	3.153569891					
PA2476	dsbG	thiol:disulfide interchange protein DsbG	63173098.12	0	0	COG1651	O	Protein-disulfide isomerase	K03981	CELLULAR PROCESSES AND SIGNALING
PA2476	dsbG	thiol:disulfide interchange protein DsbG	63173098.12	0	0	COG1651	O	Protein-disulfide isomerase	K03805	CELLULAR PROCESSES AND SIGNALING
PA2507	catA	"catechol 1	357811508.3	1794087990	5.014058934	COG3485	Q	"Protocatechuate 3,4-dioxygenase beta subunit"	K00449	METABOLISM
PA2507	catA	"catechol 1	357811508.3	1794087990	5.014058934	COG3485	Q	"Protocatechuate 3,4-dioxygenase beta subunit"	K03381	METABOLISM
PA2507	catA	"catechol 1	357811508.3	1794087990	5.014058934	COG3485	Q	"Protocatechuate 3,4-dioxygenase beta subunit"	K00448	METABOLISM
PA2508	catC	muconolactone delta-isomerase	88074162	39038931.47	0.443250672	COG4829	Q	Muconolactone delta-isomerase	K01828	METABOLISM
PA2508	catC	muconolactone delta-isomerase	88074162	39038931.47	0.443250672	COG4829	Q	Muconolactone delta-isomerase	K03464	METABOLISM
PA2532	tpx	thiol peroxidase	157780541.4	202406558.2	1.282835997	COG2077	O	Peroxiredoxin	K11065	CELLULAR PROCESSES AND SIGNALING
PA2561	ctpH	CtpH	410781354.9	901896680.6	2.19556382	COG0840	T	Methyl-accepting chemotaxis protein	K03406	CELLULAR PROCESSES AND SIGNALING
PA2561	ctpH	CtpH	410781354.9	901896680.6	2.19556382	COG0840	N	Methyl-accepting chemotaxis protein	K03406	CELLULAR PROCESSES AND SIGNALING
PA2573		probable chemotaxis transducer	513541374.9	899531154.1	1.751623527	COG0840	N	Methyl-accepting chemotaxis protein	K03406	CELLULAR PROCESSES AND SIGNALING
PA2573		probable chemotaxis transducer	513541374.9	899531154.1	1.751623527	COG0840	T	Methyl-accepting chemotaxis protein	K03406	CELLULAR PROCESSES AND SIGNALING
PA2575		hypothetical protein	133384290.6	0	0	COG3560	R	Predicted oxidoreductase related to nitroreductase	K07078	POORLY CHARACTERIZED
PA2582		hypothetical protein	1289105645	366235279.9	0.284100284	COG3109	T	Activator of osmoprotectant transporter ProP	K03607	CELLULAR PROCESSES AND SIGNALING
PA2612	serS	seryl-tRNA synthetase	0	30501215.14	1000	COG0172	J	Seryl-tRNA synthetase	K01875	INFORMATION STORAGE AND PROCESSING
PA2614	lolA	lipoprotein localization protein LolA	9509304.408	0	0	COG2834	M	Outer membrane lipoprotein-sorting protein	K03634	CELLULAR PROCESSES AND SIGNALING
PA2615	ftsK	cell division protein FtsK	44163455.03	84175466.77	1.905998222	COG1674	D	DNA segregation ATPase FtsK/SpoIIIE and related proteins	K03466	CELLULAR PROCESSES AND SIGNALING
PA2619	infA	initiation factor	42112267.86	59027142.08	1.401661442	COG0361	J	Translation initiation factor 1 (IF-1)	K03236	INFORMATION STORAGE AND PROCESSING
PA2619	infA	initiation factor	42112267.86	59027142.08	1.401661442	COG0361	J	Translation initiation factor 1 (IF-1)	K02518	INFORMATION STORAGE AND PROCESSING
PA2620	clpA	ATP-binding protease component ClpA	9766321.867	0	0	COG0542	O	"ATPases with chaperone activity, ATP-binding subunit"	K03695	CELLULAR PROCESSES AND SIGNALING
PA2620	clpA	ATP-binding protease component ClpA	9766321.867	0	0	COG0542	O	"ATPases with chaperone activity, ATP-binding subunit"	K03696	CELLULAR PROCESSES AND SIGNALING
PA2620	clpA	ATP-binding protease component ClpA	9766321.867	0	0	COG0542	O	"ATPases with chaperone activity, ATP-binding subunit"	K11907	CELLULAR PROCESSES AND SIGNALING
PA2620	clpA	ATP-binding protease component ClpA	9766321.867	0	0	COG0542	O	"ATPases with chaperone activity, ATP-binding subunit"	K03694	CELLULAR PROCESSES AND SIGNALING
PA2622	cspD	cold-shock protein CspD	479940739.7	297864318.1	0.620627285	COG1278	K	Cold shock proteins	K03704	INFORMATION STORAGE AND PROCESSING
PA2623	icd	isocitrate dehydrogenase	62879449.27	505234771.6	8.034974501	COG0538	C	Isocitrate dehydrogenases	K00030	METABOLISM
PA2623	icd	isocitrate dehydrogenase	62879449.27	505234771.6	8.034974501	COG0538	C	Isocitrate dehydrogenases	K00031	METABOLISM
PA2624	idh	isocitrate dehydrogenase	24100552.69	181139578.7	7.515992727	COG2838	C	Monomeric isocitrate dehydrogenase	K00031	METABOLISM
PA2626	trmU	tRNA methyltransferase	0	65388379.03	1000	COG0482	J	"Predicted tRNA(5-methylaminomethyl-2-thiouridylate) methyltransferase, contai"	K00566	INFORMATION STORAGE AND PROCESSING
PA2638	nuoB	NADH dehydrogenase I chain B	0	38464905.48	1000	COG0377	C	NADH:ubiquinone oxidoreductase 20 kD subunit and related Fe-S oxidoreductases	K00331	METABOLISM
PA2638	nuoB	NADH dehydrogenase I chain B	0	38464905.48	1000	COG0377	C	NADH:ubiquinone oxidoreductase 20 kD subunit and related Fe-S oxidoreductases	K05582	METABOLISM
PA2640	nuoE	NADH dehydrogenase I chain E	673536877.4	913180408	1.355798678	COG1905	C	NADH:ubiquinone oxidoreductase 24 kD subunit	K00334	METABOLISM
PA2640	nuoE	NADH dehydrogenase I chain E	673536877.4	913180408	1.355798678	COG1905	C	NADH:ubiquinone oxidoreductase 24 kD subunit	K00127	METABOLISM
PA2642	nuoG	NADH dehydrogenase I chain G	49130125.29	0	0	COG1034	C	NADH dehydrogenase/NADH:ubiquinone oxidoreductase 75 kD subunit (chain G)	K00336	METABOLISM
PA2652		methyl-accepting chemotaxis protein	513541374.9	899531154.1	1.751623527	COG0840	T	Methyl-accepting chemotaxis protein	K03406	CELLULAR PROCESSES AND SIGNALING
PA2652		methyl-accepting chemotaxis protein	513541374.9	899531154.1	1.751623527	COG0840	N	Methyl-accepting chemotaxis protein	K03406	CELLULAR PROCESSES AND SIGNALING
PA2652		methyl-accepting chemotaxis protein	513541374.9	899531154.1	1.751623527	COG4564	T	Signal transduction histidine kinase	K02480	CELLULAR PROCESSES AND SIGNALING
PA2654	tlpQ	TlpQ	410781354.9	899531154.1	2.189805217	COG0840	N	Methyl-accepting chemotaxis protein	K03406	CELLULAR PROCESSES AND SIGNALING
PA2654	tlpQ	TlpQ	410781354.9	899531154.1	2.189805217	COG0840	T	Methyl-accepting chemotaxis protein	K03406	CELLULAR PROCESSES AND SIGNALING
PA2659		hypothetical protein	4264396099	2451196194	0.574804999					
PA2667	mvaU	MvaU	1499220405	2725444454	1.81790779					
PA2738	himA	"integration host factor	2073230047	1957953622	0.944397668	COG0776	L	Bacterial nucleoid DNA-binding protein	K05788	INFORMATION STORAGE AND PROCESSING
PA2738	himA	"integration host factor	2073230047	1957953622	0.944397668	COG0776	L	Bacterial nucleoid DNA-binding protein	K03530	INFORMATION STORAGE AND PROCESSING
PA2738	himA	"integration host factor	2073230047	1957953622	0.944397668	COG0776	L	Bacterial nucleoid DNA-binding protein	K05787	INFORMATION STORAGE AND PROCESSING
PA2738	himA	"integration host factor	2073230047	1957953622	0.944397668	COG0776	L	Bacterial nucleoid DNA-binding protein	K04764	INFORMATION STORAGE AND PROCESSING
PA2740	pheS	"phenylalanyl-tRNA synthetase	18367270.22	0	0	COG0016	J	Phenylalanyl-tRNA synthetase alpha subunit	K01889	INFORMATION STORAGE AND PROCESSING
PA2742	rpmI	50S ribosomal protein L35	515195337.8	0	0	COG0291	J	Ribosomal protein L35	K02916	INFORMATION STORAGE AND PROCESSING
PA2743	infC	translation initiation factor IF-3	490013177.1	602096279.8	1.228734875	COG0290	J	Translation initiation factor 3 (IF-3)	K02520	INFORMATION STORAGE AND PROCESSING
PA2755	eco	ecotin precursor	540035520.1	690805815.7	1.279185887	COG4574	R	Serine protease inhibitor ecotin	K08276	POORLY CHARACTERIZED
PA2788		probable chemotaxis transducer	7615352150	16695818182	2.192389512	COG3850	T	"Signal transduction histidine kinase, nitrate/nitrite-specific"	K07673	CELLULAR PROCESSES AND SIGNALING
PA2788		probable chemotaxis transducer	7615352150	16695818182	2.192389512	COG3850	T	"Signal transduction histidine kinase, nitrate/nitrite-specific"	K07674	CELLULAR PROCESSES AND SIGNALING
PA2788		probable chemotaxis transducer	7615352150	16695818182	2.192389512	COG0840	T	Methyl-accepting chemotaxis protein	K03406	CELLULAR PROCESSES AND SIGNALING
PA2788		probable chemotaxis transducer	7615352150	16695818182	2.192389512	COG0840	N	Methyl-accepting chemotaxis protein	K03406	CELLULAR PROCESSES AND SIGNALING
PA2796	tal	transaldolase	40952786.11	0	0	COG0176	G	Transaldolase	K00616	METABOLISM
PA2800	vacJ	VacJ	1168233152	431868167.5	0.36967635	COG2853	M	Surface lipoprotein	K04754	CELLULAR PROCESSES AND SIGNALING
PA2805		hypothetical protein	863388245.2	654746311.5	0.758345177					
PA2817		hypothetical protein	631009026.9	457501875.5	0.72503222					
PA2823		conserved hypothetical protein	129436895.2	0	0	COG2607	R	Predicted ATPase (AAA superfamily)	K06923	POORLY CHARACTERIZED
PA2851	efp	translation elongation factor P	617500752.8	614771994	0.995580963	COG0231	J	Translation elongation factor P (EF-P)/translation initiation factor 5A (eIF-	K03263	INFORMATION STORAGE AND PROCESSING
PA2851	efp	translation elongation factor P	617500752.8	614771994	0.995580963	COG0231	J	Translation elongation factor P (EF-P)/translation initiation factor 5A (eIF-	K02356	INFORMATION STORAGE AND PROCESSING
PA2853	oprI	Outer membrane lipoprotein OprI precursor	529215170.5	2021729508	3.820241029					
PA2867		probable chemotaxis transducer	458285268.4	899531154.1	1.962819266	COG0840	T	Methyl-accepting chemotaxis protein	K03406	CELLULAR PROCESSES AND SIGNALING
PA2867		probable chemotaxis transducer	458285268.4	899531154.1	1.962819266	COG0840	N	Methyl-accepting chemotaxis protein	K03406	CELLULAR PROCESSES AND SIGNALING
PA2883		hypothetical protein	317310082.4	433870134.5	1.367338003					
PA2901		hypothetical protein	0	75181149.71	1000					
PA2920		probable chemotaxis transducer	455122929	942703111	2.071315355	COG0840	N	Methyl-accepting chemotaxis protein	K03406	CELLULAR PROCESSES AND SIGNALING
PA2920		probable chemotaxis transducer	455122929	942703111	2.071315355	COG0840	T	Methyl-accepting chemotaxis protein	K03406	CELLULAR PROCESSES AND SIGNALING
PA2945		conserved hypothetical protein	201256538.8	0	0	COG0523	R	Putative GTPases (G3E family)	K02234	POORLY CHARACTERIZED
PA2951	etfA	electron transfer flavoprotein alpha-subunit	118553956.3	661555160.6	5.580203151	COG2025	C	"Electron transfer flavoprotein, alpha subunit"	K03522	METABOLISM
PA2952	etfB	electron transfer flavoprotein beta-subunit	245001251.8	130688402.2	0.533419325	COG2086	C	"Electron transfer flavoprotein, beta subunit"	K03521	METABOLISM
PA2957		probable transcriptional regulator	397558190.9	876946869.6	2.20583273					
PA2966	acpP	acyl carrier protein	1403535623	202582696.1	0.14433741	COG0236	Q	Acyl carrier protein	K02078	METABOLISM
PA2966	acpP	acyl carrier protein	1403535623	202582696.1	0.14433741	COG0236	I	Acyl carrier protein	K14188	METABOLISM

PA2966	acpP	acyl carrier protein	1403535623	202582696.1	0.14433741	COG0236	I	Acyl carrier protein	K02078	METABOLISM
PA2966	acpP	acyl carrier protein	1403535623	202582696.1	0.14433741	COG0236	Q	Acyl carrier protein	K14188	METABOLISM
PA2967	fabG	3-oxoacyl-[acyl-carrier-protein] reductase	1958203207	2342949789	1.196479395	COG1028	I	Dehydrogenases with different specificities (related to short-chain alcohol	K00059	METABOLISM
PA2967	fabG	3-oxoacyl-[acyl-carrier-protein] reductase	1958203207	2342949789	1.196479395	COG1028	Q	Dehydrogenases with different specificities (related to short-chain alcohol	K00059	METABOLISM
PA2967	fabG	3-oxoacyl-[acyl-carrier-protein] reductase	1958203207	2342949789	1.196479395	COG1028	R	Dehydrogenases with different specificities (related to short-chain alcohol	K00059	POORLY CHARACTERIZED
PA2968	fabD	malonyl-CoA-[acyl-carrier-protein] transacylase	342795540.8	181116055.4	0.528350092	COG0331	I	(acyl-carrier-protein) S-malonyltransferase	K11533	METABOLISM
PA2968	fabD	malonyl-CoA-[acyl-carrier-protein] transacylase	342795540.8	181116055.4	0.528350092	COG0331	I	(acyl-carrier-protein) S-malonyltransferase	K00645	METABOLISM
PA2970	rpmF	50S ribosomal protein L32	33332474633	32545839703	0.976400344	COG0333	J	Ribosomal protein L32	K02911	INFORMATION STORAGE AND PROCESSING
PA2976	rne	ribonuclease E	469269648.1	1159662562	2.471207262	COG1530	J	Ribonucleases G and E	K08301	INFORMATION STORAGE AND PROCESSING
PA2976	rne	ribonuclease E	469269648.1	1159662562	2.471207262	COG1530	J	Ribonucleases G and E	K08300	INFORMATION STORAGE AND PROCESSING
PA2980		conserved hypothetical protein	3765283564	4033901477	1.071340686	COG2835	S	Uncharacterized conserved protein	K09791	POORLY CHARACTERIZED
PA3003		hypothetical protein	489839071.2	104745831.3	0.213837232					
PA3009		hypothetical protein	65909266.71	0	0					
PA3021		hypothetical protein	94596940.62	21195644.6	0.224062686					
PA3023		conserved hypothetical protein	39255873.17	21540748.68	0.548726775	COG1597	R	Sphingosine kinase and enzymes related to eukaryotic diacylglycerol kinase	K07029	POORLY CHARACTERIZED
PA3023		conserved hypothetical protein	39255873.17	21540748.68	0.548726775	COG1597	I	Sphingosine kinase and enzymes related to eukaryotic diacylglycerol kinase	K07029	METABOLISM
PA3024		probable carbohydrate kinase	0	92175780.8	1000	COG1070	G	Sugar (pentulose and hexulose) kinases	K00851	METABOLISM
PA3024		probable carbohydrate kinase	0	92175780.8	1000	COG1070	G	Sugar (pentulose and hexulose) kinases	K00848	METABOLISM
PA3024		probable carbohydrate kinase	0	92175780.8	1000	COG1070	G	Sugar (pentulose and hexulose) kinases	K00854	METABOLISM
PA3024		probable carbohydrate kinase	0	92175780.8	1000	COG1070	G	Sugar (pentulose and hexulose) kinases	K00880	METABOLISM
PA3028	moeA2	molybdenum cofactor biosynthesis protein A2	0	25462351.25	1000	COG0303	H	Molybdopterin biosynthesis enzyme	K07219	METABOLISM
PA3028	moeA2	molybdenum cofactor biosynthesis protein A2	0	25462351.25	1000	COG0303	H	Molybdopterin biosynthesis enzyme	K03750	METABOLISM
PA3031		hypothetical protein	38902708.98	339215609.5	8.719588387					
PA3040		conserved hypothetical protein	171872953.8	303478355.4	1.76571327	COG4575	S	Uncharacterized conserved protein	K05594	POORLY CHARACTERIZED
PA3049	rmf	ribosome modulation factor	279346383.7	0	0	COG3130	J	Ribosome modulation factor	K03812	INFORMATION STORAGE AND PROCESSING
PA3062	pelC	PelC	533205516	209325469.6	0.39257934					
PA3074		hypothetical protein	44792336.62	21368148.83	0.477049211	COG2304	R	Uncharacterized protein containing a von Willebrand factor type A (vWA) domai	K07114	POORLY CHARACTERIZED
PA3085		hypothetical protein	74772048.39	0	0	COG3139	S	Uncharacterized protein conserved in bacteria	K09916	POORLY CHARACTERIZED
PA3101	xcpT	general secretion pathway protein G	1506446780	1977706504	1.312828658	COG2165	N	"Type II secretory pathway, pseudopilin PulG"	K02456	CELLULAR PROCESSES AND SIGNALING
PA3101	xcpT	general secretion pathway protein G	1506446780	1977706504	1.312828658	COG2165	U	"Type II secretory pathway, pseudopilin PulG"	K02246	CELLULAR PROCESSES AND SIGNALING
PA3101	xcpT	general secretion pathway protein G	1506446780	1977706504	1.312828658	COG2165	U	"Type II secretory pathway, pseudopilin PulG"	K02679	CELLULAR PROCESSES AND SIGNALING
PA3101	xcpT	general secretion pathway protein G	1506446780	1977706504	1.312828658	COG2165	N	"Type II secretory pathway, pseudopilin PulG"	K02458	CELLULAR PROCESSES AND SIGNALING
PA3101	xcpT	general secretion pathway protein G	1506446780	1977706504	1.312828658	COG2165	U	"Type II secretory pathway, pseudopilin PulG"	K02457	CELLULAR PROCESSES AND SIGNALING
PA3101	xcpT	general secretion pathway protein G	1506446780	1977706504	1.312828658	COG2165	U	"Type II secretory pathway, pseudopilin PulG"	K02456	CELLULAR PROCESSES AND SIGNALING
PA3101	xcpT	general secretion pathway protein G	1506446780	1977706504	1.312828658	COG2165	U	"Type II secretory pathway, pseudopilin PulG"	K02246	CELLULAR PROCESSES AND SIGNALING
PA3101	xcpT	general secretion pathway protein G	1506446780	1977706504	1.312828658	COG2165	N	"Type II secretory pathway, pseudopilin PulG"	K02457	CELLULAR PROCESSES AND SIGNALING
PA3101	xcpT	general secretion pathway protein G	1506446780	1977706504	1.312828658	COG2165	U	"Type II secretory pathway, pseudopilin PulG"	K02679	CELLULAR PROCESSES AND SIGNALING
PA3101	xcpT	general secretion pathway protein G	1506446780	1977706504	1.312828658	COG2165	N	"Type II secretory pathway, pseudopilin PulG"	K02246	CELLULAR PROCESSES AND SIGNALING
PA3101	xcpT	general secretion pathway protein G	1506446780	1977706504	1.312828658	COG2165	U	"Type II secretory pathway, pseudopilin PulG"	K02456	CELLULAR PROCESSES AND SIGNALING
PA3101	xcpT	general secretion pathway protein G	1506446780	1977706504	1.312828658	COG2165	N	"Type II secretory pathway, pseudopilin PulG"	K02679	CELLULAR PROCESSES AND SIGNALING
PA3101	xcpT	general secretion pathway protein G	1506446780	1977706504	1.312828658	COG2165	N	"Type II secretory pathway, pseudopilin PulG"	K02457	CELLULAR PROCESSES AND SIGNALING
PA3101	xcpT	general secretion pathway protein G	1506446780	1977706504	1.312828658	COG2165	U	"Type II secretory pathway, pseudopilin PulG"	K02457	CELLULAR PROCESSES AND SIGNALING
PA3101	xcpT	general secretion pathway protein G	1506446780	1977706504	1.312828658	COG2165	N	"Type II secretory pathway, pseudopilin PulG"	K02458	CELLULAR PROCESSES AND SIGNALING
PA3101	xcpT	general secretion pathway protein G	1506446780	1977706504	1.312828658	COG2165	U	"Type II secretory pathway, pseudopilin PulG"	K02457	CELLULAR PROCESSES AND SIGNALING
PA3101	xcpT	general secretion pathway protein G	1506446780	1977706504	1.312828658	COG2165	N	"Type II secretory pathway, pseudopilin PulG"	K02246	CELLULAR PROCESSES AND SIGNALING
PA3101	xcpT	general secretion pathway protein G	1506446780	1977706504	1.312828658	COG2165	N	"Type II secretory pathway, pseudopilin PulG"	K02456	CELLULAR PROCESSES AND SIGNALING
PA3101	xcpT	general secretion pathway protein G	1506446780	1977706504	1.312828658	COG2165	U	"Type II secretory pathway, pseudopilin PulG"	K02458	CELLULAR PROCESSES AND SIGNALING
PA3101	xcpT	general secretion pathway protein G	1506446780	1977706504	1.312828658	COG2165	U	"Type II secretory pathway, pseudopilin PulG"	K02458	CELLULAR PROCESSES AND SIGNALING
PA3101	xcpT	general secretion pathway protein G	1506446780	1977706504	1.312828658	COG2165	N	"Type II secretory pathway, pseudopilin PulG"	K02457	CELLULAR PROCESSES AND SIGNALING
PA3101	xcpT	general secretion pathway protein G	1506446780	1977706504	1.312828658	COG2165	U	"Type II secretory pathway, pseudopilin PulG"	K02246	CELLULAR PROCESSES AND SIGNALING
PA3101	xcpT	general secretion pathway protein G	1506446780	1977706504	1.312828658	COG2165	N	"Type II secretory pathway, pseudopilin PulG"	K02679	CELLULAR PROCESSES AND SIGNALING
PA3101	xcpT	general secretion pathway protein G	1506446780	1977706504	1.312828658	COG2165	U	"Type II secretory pathway, pseudopilin PulG"	K02458	CELLULAR PROCESSES AND SIGNALING
PA3101	xcpT	general secretion pathway protein G	1506446780	1977706504	1.312828658	COG2165	U	"Type II secretory pathway, pseudopilin PulG"	K02246	CELLULAR PROCESSES AND SIGNALING
PA3101	xcpT	general secretion pathway protein G	1506446780	1977706504	1.312828658	COG2165	U	"Type II secretory pathway, pseudopilin PulG"	K02679	CELLULAR PROCESSES AND SIGNALING
PA3101	xcpT	general secretion pathway protein G	1506446780	1977706504	1.312828658	COG2165	N	"Type II secretory pathway, pseudopilin PulG"	K02679	CELLULAR PROCESSES AND SIGNALING
PA3101	xcpT	general secretion pathway protein G	1506446780	1977706504	1.312828658	COG2165	U	"Type II secretory pathway, pseudopilin PulG"	K02456	CELLULAR PROCESSES AND SIGNALING
PA3101	xcpT	general secretion pathway protein G	1506446780	1977706504	1.312828658	COG2165	U	"Type II secretory pathway, pseudopilin PulG"	K02679	CELLULAR PROCESSES AND SIGNALING
PA3101	xcpT	general secretion pathway protein G	1506446780	1977706504	1.312828658	COG2165	U	"Type II secretory pathway, pseudopilin PulG"	K02458	CELLULAR PROCESSES AND SIGNALING
PA3101	xcpT	general secretion pathway protein G	1506446780	1977706504	1.312828658	COG2165	N	"Type II secretory pathway, pseudopilin PulG"	K02456	CELLULAR PROCESSES AND SIGNALING
PA3101	xcpT	general secretion pathway protein G	1506446780	1977706504	1.312828658	COG2165	N	"Type II secretory pathway, pseudopilin PulG"	K02458	CELLULAR PROCESSES AND SIGNALING
PA3101	xcpT	general secretion pathway protein G	1506446780	1977706504	1.312828658	COG2165	N	"Type II secretory pathway, pseudopilin PulG"	K02246	CELLULAR PROCESSES AND SIGNALING
PA3101	xcpT	general secretion pathway protein G	1506446780	1977706504	1.312828658	COG2165	U	"Type II secretory pathway, pseudopilin PulG"	K02456	CELLULAR PROCESSES AND SIGNALING
PA3101	xcpT	general secretion pathway protein G	1506446780	1977706504	1.312828658	COG2165	N	"Type II secretory pathway, pseudopilin PulG"	K02246	CELLULAR PROCESSES AND SIGNALING
PA3101	xcpT	general secretion pathway protein G	1506446780	1977706504	1.312828658	COG2165	U	"Type II secretory pathway, pseudopilin PulG"	K02457	CELLULAR PROCESSES AND SIGNALING
PA3101	xcpT	general secretion pathway protein G	1506446780	1977706504	1.312828658	COG2165	N	"Type II secretory pathway, pseudopilin PulG"	K02458	CELLULAR PROCESSES AND SIGNALING
PA3101	xcpT	general secretion pathway protein G	1506446780	1977706504	1.312828658	COG2165	N	"Type II secretory pathway, pseudopilin PulG"	K02457	CELLULAR PROCESSES AND SIGNALING
PA3101	xcpT	general secretion pathway protein G	1506446780	1977706504	1.312828658	COG2165	N	"Type II secretory pathway, pseudopilin PulG"	K02456	CELLULAR PROCESSES AND SIGNALING
PA3101	xcpT	general secretion pathway protein G	1506446780	1977706504	1.312828658	COG2165	N	"Type II secretory pathway, pseudopilin PulG"	K02679	CELLULAR PROCESSES AND SIGNALING
PA3104	xcpP	secretion protein XcpP	45359568.17	0	0					
PA3110		hypothetical protein	24996612.89	36986762.24	1.479670962	COG3147	S	Uncharacterized protein conserved in bacteria	K03749	POORLY CHARACTERIZED
PA3112	accD	acetyl-CoA carboxylase beta subunit	0	10522471.23	1000	COG0777	I	Acetyl-CoA carboxylase beta subunit	K01962	METABOLISM
PA3112	accD	acetyl-CoA carboxylase beta subunit	0	10522471.23	1000	COG0777	I	Acetyl-CoA carboxylase beta subunit	K13932	METABOLISM
PA3112	accD	acetyl-CoA carboxylase beta subunit	0	10522471.23	1000	COG0777	I	Acetyl-CoA carboxylase beta subunit	K01963	METABOLISM
PA3115	fimV	Motility protein FimV	4063635578	2796604361	0.688202549	COG3170	N	Tfp pilus assembly protein FimV	K07288	CELLULAR PROCESSES AND SIGNALING
PA3115	fimV	Motility protein FimV	4063635578	2796604361	0.688202549	COG3170	U	Tfp pilus assembly protein FimV	K07288	CELLULAR PROCESSES AND SIGNALING
PA3158	wbpB	"UDP-2-acetamido-2-deoxy-d-glucuronic acid 3-dehydrogenase	83918078.69	136159769.8	1.622532021	COG0673	R	Predicted dehydrogenases and related proteins	K00010	POORLY CHARACTERIZED
PA3161	himD	integration host factor beta subunit	113923031.1	188588051.3	1.65539882	COG0776	L	Bacterial nucleoid DNA-binding protein	K05788	INFORMATION STORAGE AND PROCESSING
PA3161	himD	integration host factor beta subunit	113923031.1	188588051.3	1.65539882	COG0776	L	Bacterial nucleoid DNA-binding protein	K03530	INFORMATION STORAGE AND PROCESSING
PA3161	himD	integration host factor beta subunit	113923031.1	188588051.3	1.65539882	COG0776	L	Bacterial nucleoid DNA-binding protein	K05787	INFORMATION STORAGE AND PROCESSING
PA3161	himD	integration host factor beta subunit	113923031.1	188588051.3	1.65539882	COG0776	L	Bacterial nucleoid DNA-binding protein	K04764	INFORMATION STORAGE AND PROCESSING
PA3162	rpsA	30S ribosomal protein S1	1669032096	3106298224	1.861137501	COG0539	J	Ribosomal protein S1	K02945	INFORMATION STORAGE AND PROCESSING
PA3162	rpsA	30S ribosomal protein S1	1669032096	3106298224	1.861137501	COG1093	J	"Translation initiation factor 2, alpha subunit (eIF-2alpha)"	K03237	INFORMATION STORAGE AND PROCESSING
PA3171	ubiG	3-demethylubiquinone-9 3-methyltransferase	56242677.86	35312878.33	0.627866234	COG2227	H	"2-polyprenyl-3-methyl-5-hydroxy-6-metoxy-1,4-benzoquinol methylase"	K03428	METABOLISM
PA3171	ubiG	3-demethylubiquinone-9 3-methyltransferase	56242677.86	35312878.33	0.627866234	COG2227	H	"2-polyprenyl-3-methyl-5-hydroxy-6-metoxy-1,4-benzoquinol methylase"	K00568	METABOLISM
PA3172		probable hydrolase	10919655.69	0	0	COG0546	R	Predicted phosphatases	K01091	POORLY CHARACTERIZED
PA3190		probable binding protein component of ABC sugar transporter	660606081.5	51128379.88	7.74E-02	COG1653	G	"ABC-type sugar transport system, periplasmic component"	K05813	METABOLISM
PA3190		probable binding protein component of ABC sugar transporter	660606081.5	51128379.88	7.74E-02	COG1653	G	"ABC-type sugar transport system, periplasmic component"	K02027	METABOLISM
PA3197		hypothetical protein	14862952.45	0	0	COG1386	K	Predicted transcriptional regulator containing the HTH domain	K06024	INFORMATION STORAGE AND PROCESSING
PA3202		conserved hypothetical protein	111704398.4	0	0	COG2350	S	Uncharacterized protein conserved in bacteria	K09780	POORLY CHARACTERIZED
PA3213		hypothetical protein	63791989.33	0	0	COG1463	Q	"ABC-type transport system involved in resistance to organic solvents, peripla"	K02067	METABOLISM
PA3214		hypothetical protein	1345242185	0	0					
PA3227	ppiA	peptidyl-prolyl cis-trans isomerase A	225589602.6	0	0	COG0652	O	Peptidyl-prolyl cis-trans isomerase (rotamase) - cyclophilin family	K03768	CELLULAR PROCESSES AND SIGNALING
PA3227	ppiA	peptidyl-prolyl cis-trans isomerase A	225589602.6	0	0	COG0652	O	Peptidyl-prolyl cis-trans isomerase (rotamase) - cyclophilin family	K03767	CELLULAR PROCESSES AND SIGNALING
PA3227	ppiA	peptidyl-prolyl cis-trans isomerase A	225589602.6	0	0	COG0652	O	Peptidyl-prolyl cis-trans isomerase (rotamase) - cyclophilin family	K01802	CELLULAR PROCESSES AND SIGNALING
PA3244	minD	cell division inhibitor MinD	35400269.74	44400523.62	1.254242523	COG2894	D	Septum formation inhibitor-activating ATPase	K03609	CELLULAR PROCESSES AND SIGNALING
PA3245	minE	cell division topological specificity factor MinE	317310680.1	123721296.8	0.38990587	COG0851	D	Septum formation topological specificity factor	K03608	CELLULAR PROCESSES AND SIGNALING
PA3262		"probable peptidyl-prolyl cis-trans isomerase	17912861647	13499374066	0.753613484	COG0545	O	FKBP-type peptidyl-prolyl cis-trans isomerases 1	K09575	CELLULAR PROCESSES AND SIGNALING
PA3262		"probable peptidyl-prolyl cis-trans isomerase	17912861647	13499374066	0.753613484	COG0545	O	FKBP-type peptidyl-prolyl cis-trans isomerases 1	K03773	CELLULAR PROCESSES AND SIGNALING
PA3262		"probable peptidyl-prolyl cis-trans isomerase	17912861647	13499374066	0.753613484	COG0545	O	FKBP-type peptidyl-prolyl cis-trans isomerases 1	K03772	CELLULAR PROCESSES AND SIGNALING
PA3262		"probable peptidyl-prolyl cis-trans isomerase	17912861647	13499374066	0.753613484	COG0545	O	FKBP-type peptidyl-prolyl cis-trans isomerases 1	K01802	CELLULAR PROCESSES AND SIGNALING
PA3266	capB	cold acclimation protein B	11642276072	5058144393	0.434463533	COG1278	K	Cold shock proteins	K03704	INFORMATION STORAGE AND PROCESSING
PA3302		conserved hypothetical protein	161517285	170386655.6	1.054912826					
PA3309		conserved hypothetical protein	8341806446	9236749573	1.107284092	COG0589	T	Universal stress protein UspA and related nucleotide-binding proteins	K06149	CELLULAR PROCESSES AND SIGNALING
PA3313		hypothetical protein	18014447.58	0	0	COG3221	P	"ABC-type phosphate/phosphonate transport system, periplasmic component"	K02044	METABOLISM
PA3326	clpP2	ClpP2	702309597.7	0	0	COG0740	O	Protease subunit of ATP-dependent Clp proteases	K01358	CELLULAR PROCESSES AND SIGNALING
PA3326	clpP2	ClpP2	702309597.7	0	0	COG0740	U	Protease subunit of ATP-dependent Clp proteases	K01358	CELLULAR PROCESSES AND SIGNALING
PA3347	hsbA	HptB-dependent secretion and biofilm anti anti-sigma factor HsbA	86041247.83	68494411.43	0.796064831	COG1366	T	Anti-anti-sigma regulatory factor (antagonist of anti-sigma factor)	K04749	CELLULAR PROCESSES AND SIGNALING
PA3347	hsbA	HptB-dependent secretion and biofilm anti anti-sigma factor HsbA	86041247.83	68494411.43	0.796064831	COG1366	T	Anti-anti-sigma regulatory factor (antagonist of anti-sigma factor)	K06378	CELLULAR PROCESSES AND SIGNALING
PA3351	flgM	FlgM	357557052.5	290665326.4	0.812920132	COG2747	U	Negative regulator of flagellin synthesis (anti-sigma28 factor)	K02398	CELLULAR PROCESSES AND SIGNALING
PA3351	flgM	FlgM	357557052.5	290665326.4	0.812920132	COG2747	N	Negative regulator of flagellin synthesis (anti-sigma28 factor)	K02398	CELLULAR PROCESSES AND SIGNALING
PA3351	flgM	FlgM	357557052.5	290665326.4	0.812920132	COG2747	K	Negative regulator of flagellin synthesis (anti-sigma28 factor)	K02398	INFORMATION STORAGE AND PROCESSING
PA3352		hypothetical protein	1176496989	77519213.16	6.59E-02					
PA3385	amrZ	alginate and motility regulator Z	2112404290	2828314923	1.338907962					
PA3413		conserved hypothetical protein	0	100226966.4	1000	COG3141	S	Uncharacterized protein conserved in bacteria	K09918	POORLY CHARACTERIZED
PA3424		hypothetical protein	766660877.5	0	0	COG0665	E	Glycine/D-amino acid oxidases (deaminating)	K00303	METABOLISM
PA3424		hypothetical protein	766660877.5	0	0	COG0665	E	Glycine/D-amino acid oxidases (deaminating)	K00285	METABOLISM
PA3424		hypothetical protein	766660877.5	0	0	COG0665	E	Glycine/D-amino acid oxidases (deaminating)	K03153	METABOLISM
PA3435		conserved hypothetical protein	254254307.2	0	0	COG0716	C	Flavodoxins	K03839	METABOLISM
PA3435		conserved hypothetical protein	254254307.2	0	0	COG0716	C	Flavodoxins	K03840	METABOLISM
PA3435		conserved hypothetical protein	254254307.2	0	0	COG0716	C	Flavodoxins	K06205	METABOLISM
PA3440		conserved hypothetical protein	1250327602	1054326927	0.843240544	COG3492	S	Uncharacterized protein conserved in bacteria	K09948	POORLY CHARACTERIZED
PA3441		probable molybdopterin-binding protein	0	214685531.8	1000					
PA3452	mqoA	malate:quinone oxidoreductase	0	491514182.8	1000	COG0579	R	Predicted dehydrogenase	K00116	POORLY CHARACTERIZED
PA3452	mqoA	malate:quinone oxidoreductase	0	491514182.8	1000	COG0579	R	Predicted dehydrogenase	K00111	POORLY CHARACTERIZED
PA3453		conserved hypothetical protein	45751242.29	380182304.4	8.309770083	COG3132	S	Uncharacterized protein conserved in bacteria	K03810	POORLY CHARACTERIZED
PA3453		conserved hypothetical protein	45751242.29	380182304.4	8.309770083	COG3132	S	Uncharacterized protein conserved in bacteria	K09915	POORLY CHARACTERIZED
PA3470		hypothetical protein	14915978.31	0	0	COG0494	R	NTP pyrophosphohydrolases including oxidative damage repair enzymes	K01515	POORLY CHARACTERIZED
PA3470		hypothetical protein	14915978.31	0	0	COG0494	L	NTP pyrophosphohydrolases including oxidative damage repair enzymes	K01515	INFORMATION STORAGE AND PROCESSING
PA3470		hypothetical protein	14915978.31	0	0	COG0494	R	NTP pyrophosphohydrolases including oxidative damage repair enzymes	K03574	POORLY CHARACTERIZED
PA3470		hypothetical protein	14915978.31	0	0	COG0494	R	NTP pyrophosphohydrolases including oxidative damage repair enzymes	K08311	POORLY CHARACTERIZED
PA3470		hypothetical protein	14915978.31	0	0	COG0494	L	NTP pyrophosphohydrolases including oxidative damage repair enzymes	K03574	INFORMATION STORAGE AND PROCESSING
PA3470		hypothetical protein	14915978.31	0	0	COG0494	L	NTP pyrophosphohydrolases including oxidative damage repair enzymes	K08311	INFORMATION STORAGE AND PROCESSING
PA3483		hypothetical protein	0	13946450.72	1000					
PA3525	argG	argininosuccinate synthase	23610511.76	30724686.53	1.301313874	COG0137	E	Argininosuccinate synthase	K01940	METABOLISM
PA3529		alkylhydroperoxide reductase C	3146845765	2326512163	0.739315599	COG0450	O	Peroxiredoxin	K03386	CELLULAR PROCESSES AND SIGNALING
PA3531	bfrB	bacterioferritin	2392674570	3649252423	1.525177084	COG2193	P	Bacterioferritin (cytochrome b1)	K03594	METABOLISM
PA3533	grxD	GrxD	189650450.1	250069936.2	1.318583405	COG0278	O	Glutaredoxin-related protein	K07390	CELLULAR PROCESSES AND SIGNALING
PA3562	fruI	"phosphotransferase system transporter enzyme I	25574432.54	0	0	COG4668	G	"Mannitol/fructose-specific phosphotransferase system, IIA domain"	K11183	METABOLISM
PA3562	fruI	"phosphotransferase system transporter enzyme I	25574432.54	0	0	COG1080	G	Phosphoenolpyruvate-protein kinase (PTS system EI component in bacteria)	K02768	METABOLISM
PA3562	fruI	"phosphotransferase system transporter enzyme I	25574432.54	0	0	COG4668	G	"Mannitol/fructose-specific phosphotransferase system, IIA domain"	K02798	METABOLISM
PA3562	fruI	"phosphotransferase system transporter enzyme I	25574432.54	0	0	COG4668	G	"Mannitol/fructose-specific phosphotransferase system, IIA domain"	K02768	METABOLISM
PA3562	fruI	"phosphotransferase system transporter enzyme I	25574432.54	0	0	COG4668	G	"Mannitol/fructose-specific phosphotransferase system, IIA domain"	K02800	METABOLISM
PA3562	fruI	"phosphotransferase system transporter enzyme I	25574432.54	0	0	COG1080	G	Phosphoenolpyruvate-protein kinase (PTS system EI component in bacteria)	K08483	METABOLISM
PA3562	fruI	"phosphotransferase system transporter enzyme I	25574432.54	0	0	COG4668	G	"Mannitol/fructose-specific phosphotransferase system, IIA domain"	K08483	METABOLISM
PA3562	fruI	"phosphotransferase system transporter enzyme I	25574432.54	0	0	COG1080	G	Phosphoenolpyruvate-protein kinase (PTS system EI component in bacteria)	K11183	METABOLISM
PA3562	fruI	"phosphotransferase system transporter enzyme I	25574432.54	0	0	COG1080	G	Phosphoenolpyruvate-protein kinase (PTS system EI component in bacteria)	K11201	METABOLISM

PA3562	fruI	"phosphotransferase system transporter enzyme I	25574432.54	0	0	COG1925	G	"Phosphotransferase system, HPr-related proteins"	K02768	METABOLISM
PA3562	fruI	"phosphotransferase system transporter enzyme I	25574432.54	0	0	COG1925	G	"Phosphotransferase system, HPr-related proteins"	K11183	METABOLISM
PA3562	fruI	"phosphotransferase system transporter enzyme I	25574432.54	0	0	COG1925	G	"Phosphotransferase system, HPr-related proteins"	K08485	METABOLISM
PA3562	fruI	"phosphotransferase system transporter enzyme I	25574432.54	0	0	COG1925	G	"Phosphotransferase system, HPr-related proteins"	K02784	METABOLISM
PA3562	fruI	"phosphotransferase system transporter enzyme I	25574432.54	0	0	COG4668	G	"Mannitol/fructose-specific phosphotransferase system, IIA domain"	K02799	METABOLISM
PA3562	fruI	"phosphotransferase system transporter enzyme I	25574432.54	0	0	COG1925	G	"Phosphotransferase system, HPr-related proteins"	K08483	METABOLISM
PA3562	fruI	"phosphotransferase system transporter enzyme I	25574432.54	0	0	COG1925	G	"Phosphotransferase system, HPr-related proteins"	K11189	METABOLISM
PA3562	fruI	"phosphotransferase system transporter enzyme I	25574432.54	0	0	COG1080	G	Phosphoenolpyruvate-protein kinase (PTS system EI component in bacteria)	K11189	METABOLISM
PA3580		conserved hypothetical protein	18272916.64	0	0	COG2606	S	Uncharacterized conserved protein	K03976	POORLY CHARACTERIZED
PA3611		hypothetical protein	66674427.32	29729536.01	0.445891134					
PA3621	fdxA	ferredoxin I	1599082361	1405891315	0.879186306	COG1146	C	Ferredoxin	K05524	METABOLISM
PA3621	fdxA	ferredoxin I	1599082361	1405891315	0.879186306	COG1146	C	Ferredoxin	K00176	METABOLISM
PA3635	eno	enolase	11065429845	7549911594	0.682297181	COG0148	G	Enolase	K01689	METABOLISM
PA3639	accA	acetyl-coenzyme A carboxylase carboxyl transferase (alpha subunit)	110295498.7	30076743.8	0.272692396	COG0825	I	Acetyl-CoA carboxylase alpha subunit	K01963	METABOLISM
PA3639	accA	acetyl-coenzyme A carboxylase carboxyl transferase (alpha subunit)	110295498.7	30076743.8	0.272692396	COG0825	I	Acetyl-CoA carboxylase alpha subunit	K01962	METABOLISM
PA3647		probable outer membrane protein precursor	7564285087	14060003255	1.85873524	COG2825	M	Outer membrane protein	K06142	CELLULAR PROCESSES AND SIGNALING
PA3653	frr	ribosome recycling factor	10437034968	6072247426	0.58179813	COG0233	J	Ribosome recycling factor	K02838	INFORMATION STORAGE AND PROCESSING
PA3655	tsf	elongation factor Ts	1871173339	1818209895	0.971695063	COG0264	J	Translation elongation factor Ts	K02357	INFORMATION STORAGE AND PROCESSING
PA3656	rpsB	30S ribosomal protein S2	1224185255	1173397877	0.958513323	COG0052	J	Ribosomal protein S2	K02967	INFORMATION STORAGE AND PROCESSING
PA3657	map	methionine aminopeptidase	25761261.18	0	0	COG0024	J	Methionine aminopeptidase	K01265	INFORMATION STORAGE AND PROCESSING
PA3664		conserved hypothetical protein	119051511.4	0	0	COG1393	P	"Arsenate reductase and related proteins, glutaredoxin family"	K00537	METABOLISM
PA3674		hypothetical protein	16090488.32	0	0	COG3126	S	Uncharacterized protein conserved in bacteria	K09914	POORLY CHARACTERIZED
PA3675		hypothetical protein	23330183.42	0	0					
PA3686	adk	adenylate kinase	4659778607	2375662482	0.509823037	COG0563	F	Adenylate kinase and related kinases	K00939	METABOLISM
PA3688		hypothetical protein	813845952.7	1604790415	1.971860165					
PA3691		hypothetical protein	15966658850	8273387072	0.518166459					
PA3692	lptF	"Lipotoxon F	13293976140	9069335364	0.682213904	COG2885	M	Outer membrane protein and related peptidoglycan-associated (lipo)proteins	K03286	CELLULAR PROCESSES AND SIGNALING
PA3692	lptF	"Lipotoxon F	13293976140	9069335364	0.682213904	COG2885	M	Outer membrane protein and related peptidoglycan-associated (lipo)proteins	K03640	CELLULAR PROCESSES AND SIGNALING
PA3694		hypothetical protein	255191012	144981008.9	0.568127411					
PA3708	wspA	probable chemotaxis transducer	640836828	83806074.4	0.130775996	COG0840	N	Methyl-accepting chemotaxis protein	K03406	CELLULAR PROCESSES AND SIGNALING
PA3708	wspA	probable chemotaxis transducer	640836828	83806074.4	0.130775996	COG0840	T	Methyl-accepting chemotaxis protein	K03406	CELLULAR PROCESSES AND SIGNALING
PA3712		hypothetical protein	252833111.7	378057939.7	1.495286504					
PA3716		hypothetical protein	41193067.55	18028508.99	0.437658812					
PA3731		conserved hypothetical protein	823503063.5	287493027.1	0.349109845	COG1842	K	"Phage shock protein A (IM30), suppresses sigma54-dependent transcription"	K03969	INFORMATION STORAGE AND PROCESSING
PA3731		conserved hypothetical protein	823503063.5	287493027.1	0.349109845	COG1842	T	"Phage shock protein A (IM30), suppresses sigma54-dependent transcription"	K03969	CELLULAR PROCESSES AND SIGNALING
PA3745	rpsP	30S ribosomal protein S16	61743449.18	409417372.9	6.630944308	COG0228	J	Ribosomal protein S16	K02959	INFORMATION STORAGE AND PROCESSING
PA3746	ffh	signal recognition particle protein Ffh	641764225.5	878875876.2	1.369468476	COG0541	U	Signal recognition particle GTPase	K03106	CELLULAR PROCESSES AND SIGNALING
PA3753		conserved hypothetical protein	595882425.2	174267522.7	0.292452865	COG0663	R	"Carbonic anhydrases/acetyltransferases, isoleucine patch superfamily"	K08279	POORLY CHARACTERIZED
PA3765		hypothetical protein	19952495.84	0	0	COG3644	S	Uncharacterized protein conserved in bacteria	K09965	POORLY CHARACTERIZED
PA3785		conserved hypothetical protein	6839290069	2024043589	0.295943522	COG2847	S	Uncharacterized protein conserved in bacteria	K09796	POORLY CHARACTERIZED
PA3790	oprC	Putative copper transport outer membrane porin OprC precursor	90488930.68	343979556.7	3.801344033	COG4774	P	Outer membrane receptor for monomeric catechols	K02014	METABOLISM
PA3790	oprC	Putative copper transport outer membrane porin OprC precursor	90488930.68	343979556.7	3.801344033	COG1629	P	"Outer membrane receptor proteins, mostly Fe transport"	K02014	METABOLISM
PA3801		conserved hypothetical protein	1284661206	1983907668	1.544304178					
PA3804		hypothetical protein	322315518.8	859529584	2.666733477					
PA3805	pilF	type 4 fimbrial biogenesis protein PilF	378548974.7	66330458.68	0.175222925	COG3063	U	Tfp pilus assembly protein PilF	K02656	CELLULAR PROCESSES AND SIGNALING
PA3805	pilF	type 4 fimbrial biogenesis protein PilF	378548974.7	66330458.68	0.175222925	COG3063	N	Tfp pilus assembly protein PilF	K02656	CELLULAR PROCESSES AND SIGNALING
PA3807	ndk	nucleoside diphosphate kinase	7359967308	9526455320	1.294361092	COG0105	F	Nucleoside diphosphate kinase	K00940	METABOLISM
PA3808		conserved hypothetical protein	14991631.95	0	0					
PA3809	fdx2	ferredoxin [2Fe-2S]	105816820	80554886.01	0.761267311	COG0633	C	Ferredoxin	K11107	METABOLISM
PA3809	fdx2	ferredoxin [2Fe-2S]	105816820	80554886.01	0.761267311	COG0633	C	Ferredoxin	K04755	METABOLISM
PA3809	fdx2	ferredoxin [2Fe-2S]	105816820	80554886.01	0.761267311	COG0633	C	Ferredoxin	K02639	METABOLISM
PA3813	iscU	probable iron-binding protein IscU	1892922480	3063225715	1.618252066	COG0822	C	NifU homolog involved in Fe-S cluster formation	K13819	METABOLISM
PA3813	iscU	probable iron-binding protein IscU	1892922480	3063225715	1.618252066	COG0822	C	NifU homolog involved in Fe-S cluster formation	K04488	METABOLISM
PA3816	cysE	O-acetylserine synthase	95473020.06	133100975.3	1.394121346	COG1045	E	Serine acetyltransferase	K00640	METABOLISM
PA3819		conserved hypothetical protein	15674307.89	0	0	COG3134	S	Predicted outer membrane lipoprotein	K06077	POORLY CHARACTERIZED
PA3834	valS	valyl-tRNA synthetase	0	60181313.96	1000	COG0525	J	Valyl-tRNA synthetase	K01873	INFORMATION STORAGE AND PROCESSING
PA3836		hypothetical protein	179122297.8	0	0	COG2984	R	"ABC-type uncharacterized transport system, periplasmic component"	K01989	POORLY CHARACTERIZED
PA3902		hypothetical protein	57087334.57	0	0					
PA3910	eddA	"Extracelullar DNA degradation protein	0	442499171	1000	COG3540	P	Phosphodiesterase/alkaline phosphatase D	K01113	METABOLISM
PA3918	moaC	molybdopterin biosynthetic protein C	0	27745354.86	1000	COG0315	H	Molybdenum cofactor biosynthesis enzyme	K03637	METABOLISM
PA3931		conserved hypothetical protein	394284078.8	17694592.82	4.49E-02	COG1464	P	"ABC-type metal ion transport system, periplasmic component/surface antigen"	K02073	METABOLISM
PA3940		probable DNA binding protein	4167004413	4612225737	1.106844457	COG0776	L	Bacterial nucleoid DNA-binding protein	K05787	INFORMATION STORAGE AND PROCESSING
PA3940		probable DNA binding protein	4167004413	4612225737	1.106844457	COG0776	L	Bacterial nucleoid DNA-binding protein	K03530	INFORMATION STORAGE AND PROCESSING
PA3940		probable DNA binding protein	4167004413	4612225737	1.106844457	COG0776	L	Bacterial nucleoid DNA-binding protein	K05788	INFORMATION STORAGE AND PROCESSING
PA3940		probable DNA binding protein	4167004413	4612225737	1.106844457	COG0776	L	Bacterial nucleoid DNA-binding protein	K04764	INFORMATION STORAGE AND PROCESSING
PA3981		conserved hypothetical protein	36186179.61	0	0	COG1702	T	"Phosphate starvation-inducible protein PhoH, predicted ATPase"	K06217	CELLULAR PROCESSES AND SIGNALING
PA3983		conserved hypothetical protein	524192656.6	0	0	COG4535	P	Putative Mg2 and Co2 transporter CorC	K06189	METABOLISM
PA3988	lptE	LPS-assembly lipoprotein LptE	5567686374	4278256016	0.768408227	COG2980	M	Rare lipoprotein B	K03643	CELLULAR PROCESSES AND SIGNALING
PA3999	dacC	D-ala-D-ala-carboxypeptidase	15722381.26	0	0	COG1686	M	D-alanyl-D-alanine carboxypeptidase	K07258	CELLULAR PROCESSES AND SIGNALING
PA3999	dacC	D-ala-D-ala-carboxypeptidase	15722381.26	0	0	COG1686	M	D-alanyl-D-alanine carboxypeptidase	K01286	CELLULAR PROCESSES AND SIGNALING
PA3999	dacC	D-ala-D-ala-carboxypeptidase	15722381.26	0	0	COG1686	M	D-alanyl-D-alanine carboxypeptidase	K07262	CELLULAR PROCESSES AND SIGNALING
PA4005		conserved hypothetical protein	712462214.2	919843527.4	1.291076929	COG0799	S	Uncharacterized homolog of plant Iojap protein	K09710	POORLY CHARACTERIZED
PA4017		conserved hypothetical protein	13486670.92	21547633.55	1.597698474	COG0702	G	Predicted nucleoside-diphosphate-sugar epimerases	K00356	METABOLISM
PA4017		conserved hypothetical protein	13486670.92	21547633.55	1.597698474	COG0702	G	Predicted nucleoside-diphosphate-sugar epimerases	K00329	METABOLISM
PA4017		conserved hypothetical protein	13486670.92	21547633.55	1.597698474	COG0702	M	Predicted nucleoside-diphosphate-sugar epimerases	K00329	CELLULAR PROCESSES AND SIGNALING
PA4017		conserved hypothetical protein	13486670.92	21547633.55	1.597698474	COG0702	M	Predicted nucleoside-diphosphate-sugar epimerases	K00356	CELLULAR PROCESSES AND SIGNALING
PA4031	ppa	inorganic pyrophosphatase	7926878657	8260815491	1.042127154	COG0221	C	Inorganic pyrophosphatase	K01507	METABOLISM
PA4042	xseB	exodeoxyribonuclease VII small subunit	65966561.96	171031920.9	2.592706301	COG1722	L	Exonuclease VII small subunit	K03602	INFORMATION STORAGE AND PROCESSING
PA4052	nusB	NusB protein	60786422.05	0	0	COG0781	K	Transcription termination factor	K03625	INFORMATION STORAGE AND PROCESSING
PA4057	nrdR	NrdR	16637141.4	50863694.9	3.057237639	COG1327	K	"Predicted transcriptional regulator, consists of a Zn-ribbon and ATP-cone dom"	K07738	INFORMATION STORAGE AND PROCESSING
PA4063		hypothetical protein	94522653.18	0	0					
PA4067	oprG	Outer membrane protein OprG precursor	3166437156	8032937784	2.536901062	COG3047	M	Outer membrane protein W	K07275	CELLULAR PROCESSES AND SIGNALING
PA4139		hypothetical protein	1941169183	453966303.7	0.233862307					
PA4164		hypothetical protein	14703106.38	0	0					
PA4175	piv	protease IV	1775818154	594437149.3	0.334739876					
PA4176	ppiC2	peptidyl-prolyl cis-trans isomerase C2	1933971840	2451707778	1.267706038	COG0760	O	Parvulin-like peptidyl-prolyl isomerase	K03769	CELLULAR PROCESSES AND SIGNALING
PA4176	ppiC2	peptidyl-prolyl cis-trans isomerase C2	1933971840	2451707778	1.267706038	COG0760	O	Parvulin-like peptidyl-prolyl isomerase	K03770	CELLULAR PROCESSES AND SIGNALING
PA4176	ppiC2	peptidyl-prolyl cis-trans isomerase C2	1933971840	2451707778	1.267706038	COG0760	O	Parvulin-like peptidyl-prolyl isomerase	K07533	CELLULAR PROCESSES AND SIGNALING
PA4176	ppiC2	peptidyl-prolyl cis-trans isomerase C2	1933971840	2451707778	1.267706038	COG0760	O	Parvulin-like peptidyl-prolyl isomerase	K03771	CELLULAR PROCESSES AND SIGNALING
PA4211	phzB1	probable phenazine biosynthesis protein	0	161895794.6	1000	COG3631	R	Ketosteroid isomerase-related protein	K06893	POORLY CHARACTERIZED
PA4226	pchE	dihydroaeruginoic acid synthetase	39561476.32	0	0	COG3433	Q	Aryl carrier domain	K12239	METABOLISM
PA4226	pchE	dihydroaeruginoic acid synthetase	39561476.32	0	0	COG3433	Q	Aryl carrier domain	K04788	METABOLISM
PA4226	pchE	dihydroaeruginoic acid synthetase	39561476.32	0	0	COG3433	Q	Aryl carrier domain	K04779	METABOLISM
PA4226	pchE	dihydroaeruginoic acid synthetase	39561476.32	0	0	COG3433	Q	Aryl carrier domain	K04784	METABOLISM
PA4226	pchE	dihydroaeruginoic acid synthetase	39561476.32	0	0	COG3433	Q	Aryl carrier domain	K01252	METABOLISM
PA4230	pchB	salicylate biosynthesis protein PchB	937393049.5	707534996.8	0.754790103	COG1605	E	Chorismate mutase	K04516	METABOLISM
PA4230	pchB	salicylate biosynthesis protein PchB	937393049.5	707534996.8	0.754790103	COG1605	E	Chorismate mutase	K04093	METABOLISM
PA4230	pchB	salicylate biosynthesis protein PchB	937393049.5	707534996.8	0.754790103	COG1605	E	Chorismate mutase	K14170	METABOLISM
PA4230	pchB	salicylate biosynthesis protein PchB	937393049.5	707534996.8	0.754790103	COG1605	E	Chorismate mutase	K04092	METABOLISM
PA4230	pchB	salicylate biosynthesis protein PchB	937393049.5	707534996.8	0.754790103	COG1605	E	Chorismate mutase	K14187	METABOLISM
PA4230	pchB	salicylate biosynthesis protein PchB	937393049.5	707534996.8	0.754790103	COG1605	E	Chorismate mutase	K04782	METABOLISM
PA4230	pchB	salicylate biosynthesis protein PchB	937393049.5	707534996.8	0.754790103	COG1605	E	Chorismate mutase	K01850	METABOLISM
PA4230	pchB	salicylate biosynthesis protein PchB	937393049.5	707534996.8	0.754790103	COG1605	E	Chorismate mutase	K13853	METABOLISM
PA4232	ssb	single-stranded DNA-binding protein	223603395.6	380110491.4	1.699931659	COG0629	L	Single-stranded DNA-binding protein	K03111	INFORMATION STORAGE AND PROCESSING
PA4235	ftnA	bacterial ferritin	2953723188	4489920811	1.520088554	COG2193	P	Bacterioferritin (cytochrome b1)	K03594	METABOLISM
PA4236	katA	catalase	219116690.5	50093450.12	0.228615401	COG0753	P	Catalase	K00429	METABOLISM
PA4236	katA	catalase	219116690.5	50093450.12	0.228615401	COG0753	P	Catalase	K03781	METABOLISM
PA4237	rplQ	50S ribosomal protein L17	6546602686	231197743.9	3.53E-02	COG0203	J	Ribosomal protein L17	K02879	INFORMATION STORAGE AND PROCESSING
PA4238	rpoA	DNA-directed RNA polymerase alpha chain	531098570.5	947295701.5	1.783653269	COG0202	K	"DNA-directed RNA polymerase, alpha subunit/40 kD subunit"	K03047	INFORMATION STORAGE AND PROCESSING
PA4238	rpoA	DNA-directed RNA polymerase alpha chain	531098570.5	947295701.5	1.783653269	COG0202	K	"DNA-directed RNA polymerase, alpha subunit/40 kD subunit"	K03040	INFORMATION STORAGE AND PROCESSING
PA4239	rpsD	30S ribosomal protein S4	0	43264042.03	1000	COG0522	J	Ribosomal protein S4 and related proteins	K02986	INFORMATION STORAGE AND PROCESSING
PA4240	rpsK	30S ribosomal protein S11	152807637.9	352315030	2.305611388	COG0100	J	Ribosomal protein S11	K02948	INFORMATION STORAGE AND PROCESSING
PA4244	rplO	50S ribosomal protein L15	1278659294	1160534646	0.907618356	COG0200	J	Ribosomal protein L15	K02876	INFORMATION STORAGE AND PROCESSING
PA4246	rpsE	30S ribosomal protein S5	0	130389006	1000	COG0098	J	Ribosomal protein S5	K02981	INFORMATION STORAGE AND PROCESSING
PA4246	rpsE	30S ribosomal protein S5	0	130389006	1000	COG0098	J	Ribosomal protein S5	K02988	INFORMATION STORAGE AND PROCESSING
PA4247	rplR	50S ribosomal protein L18	24565916.52	53789668.83	2.189605618	COG0256	J	Ribosomal protein L18	K02881	INFORMATION STORAGE AND PROCESSING
PA4248	rplF	50S ribosomal protein L6	40375649.57	68086674.15	1.686330124	COG0097	J	Ribosomal protein L6P/L9E	K02940	INFORMATION STORAGE AND PROCESSING
PA4248	rplF	50S ribosomal protein L6	40375649.57	68086674.15	1.686330124	COG0097	J	Ribosomal protein L6P/L9E	K02933	INFORMATION STORAGE AND PROCESSING
PA4249	rpsH	30S ribosomal protein S8	2897947838	4338363796	1.497046889	COG0096	J	Ribosomal protein S8	K02994	INFORMATION STORAGE AND PROCESSING
PA4250	rpsN	30S ribosomal protein S14	203467315.9	0	0	COG0199	J	Ribosomal protein S14	K02954	INFORMATION STORAGE AND PROCESSING
PA4251	rplE	50S ribosomal protein L5	1242171353	1378803178	1.109994345	COG0094	J	Ribosomal protein L5	K02931	INFORMATION STORAGE AND PROCESSING
PA4252	rplX	50S ribosomal protein L24	13932702949	17891729582	1.284153523	COG0198	J	Ribosomal protein L24	K02895	INFORMATION STORAGE AND PROCESSING
PA4254	rpsQ	30S ribosomal protein S17	64356232.31	16508674.04	0.256520207	COG0186	J	Ribosomal protein S17	K02961	INFORMATION STORAGE AND PROCESSING
PA4255	rpmC	50S ribosomal protein L29	29593408557	27857004493	0.941324634	COG0255	J	Ribosomal protein L29	K02904	INFORMATION STORAGE AND PROCESSING
PA4255	rpmC	50S ribosomal protein L29	29593408557	27857004493	0.941324634	COG0255	J	Ribosomal protein L29	K02918	INFORMATION STORAGE AND PROCESSING
PA4257	rpsC	30S ribosomal protein S3	130109751.6	296903305	2.281945061	COG0092	J	Ribosomal protein S3	K02982	INFORMATION STORAGE AND PROCESSING
PA4258	rplV	50S ribosomal protein L22	637819135.6	95164473.97	0.149202915	COG0091	J	Ribosomal protein L22	K02890	INFORMATION STORAGE AND PROCESSING
PA4259	rpsS	30S ribosomal protein S19	267154022	208131518.5	0.779069381	COG0185	J	Ribosomal protein S19	K02965	INFORMATION STORAGE AND PROCESSING
PA4260	rplB	50S ribosomal protein L2	0	26669498.37	1000	COG0090	J	Ribosomal protein L2	K02886	INFORMATION STORAGE AND PROCESSING
PA4264	rpsJ	30S ribosomal protein S10	48783706.74	105752552.3	2.167784273	COG0051	J	Ribosomal protein S10	K02946	INFORMATION STORAGE AND PROCESSING
PA4266	fusA1	elongation factor G	13281740.06	61063533.49	4.597555231	COG0480	J	Translation elongation factors (GTPases)	K02355	INFORMATION STORAGE AND PROCESSING
PA4266	fusA1	elongation factor G	13281740.06	61063533.49	4.597555231	COG0480	J	Translation elongation factors (GTPases)	K03234	INFORMATION STORAGE AND PROCESSING
PA4267	rpsG	30S ribosomal protein S7	2525004406	1225258157	0.485249908	COG0049	J	Ribosomal protein S7	K02992	INFORMATION STORAGE AND PROCESSING
PA4268	rpsL	30S ribosomal protein S12	0	21658173.96	1000	COG0048	J	Ribosomal protein S12	K02950	INFORMATION STORAGE AND PROCESSING
PA4269	rpoC	DNA-directed RNA polymerase beta* chain	13120869.33	35221845.06	2.684413979	COG0086	K	"DNA-directed RNA polymerase, beta' subunit/160 kD subunit"	K03042	INFORMATION STORAGE AND PROCESSING
PA4269	rpoC	DNA-directed RNA polymerase beta* chain	13120869.33	35221845.06	2.684413979	COG0086	K	"DNA-directed RNA polymerase, beta' subunit/160 kD subunit"	K03046	INFORMATION STORAGE AND PROCESSING
PA4269	rpoC	DNA-directed RNA polymerase beta* chain	13120869.33	35221845.06	2.684413979	COG0086	K	"DNA-directed RNA polymerase, beta' subunit/160 kD subunit"	K03041	INFORMATION STORAGE AND PROCESSING
PA4271	rplL	50S ribosomal protein L7 / L12	7177794870	58801308795	8.192113296	COG0222	J	Ribosomal protein L7/L12	K02935	INFORMATION STORAGE AND PROCESSING
PA4272	rplJ	50S ribosomal protein L10	2073773114	2646321047	1.276089958	COG0244	J	Ribosomal protein L10	K02864	INFORMATION STORAGE AND PROCESSING
PA4273	rplA	50S ribosomal protein L1	1421773616	578717271	0.407038972	COG0081	J	Ribosomal protein L1	K02863	INFORMATION STORAGE AND PROCESSING
PA4274	rplK	50S ribosomal protein L11	0	138654578.8	1000	COG0080	J	Ribosomal protein L11	K02867	INFORMATION STORAGE AND PROCESSING
PA4275	nusG	transcription antitermination protein NusG	0	11748838.61	1000	COG0250	K	Transcription antiterminator	K05785	INFORMATION STORAGE AND PROCESSING
PA4275	nusG	transcription antitermination protein NusG	0	11748838.61	1000	COG0250	K	Transcription antiterminator	K02601	INFORMATION STORAGE AND PROCESSING
PA4277	tufB	elongation factor Tu	2401406332	4956715922	2.064088803	COG0050	J	GTPases - translation elongation factors	K02358	INFORMATION STORAGE AND PROCESSING
PA4307	pctC	chemotactic transducer PctC	536096151.2	1021093088	1.904682744	COG0840	T	Methyl-accepting chemotaxis protein	K03406	CELLULAR PROCESSES AND SIGNALING
PA4307	pctC	chemotactic transducer PctC	536096151.2	1021093088	1.904682744	COG0840	N	Methyl-accepting chemotaxis protein	K03406	CELLULAR PROCESSES AND SIGNALING

PA4308		conserved hypothetical protein	25461976.73	0	0	COG0433	R	Predicted ATPase	K06915	POORLY CHARACTERIZED
PA4309	pctA	chemotactic transducer PctA	699699034.6	1021093088	1.459331852	COG0840	T	Methyl-accepting chemotaxis protein	K03406	CELLULAR PROCESSES AND SIGNALING
PA4309	pctA	chemotactic transducer PctA	699699034.6	1021093088	1.459331852	COG0840	N	Methyl-accepting chemotaxis protein	K03406	CELLULAR PROCESSES AND SIGNALING
PA4310	pctB	chemotactic transducer PctB	649591303.7	1021093088	1.571900797	COG0840	N	Methyl-accepting chemotaxis protein	K03406	CELLULAR PROCESSES AND SIGNALING
PA4310	pctB	chemotactic transducer PctB	649591303.7	1021093088	1.571900797	COG0840	T	Methyl-accepting chemotaxis protein	K03406	CELLULAR PROCESSES AND SIGNALING
PA4315	mvaT	"transcriptional regulator MvaT	43639694437	39897073087	0.91423814					
PA4336		conserved hypothetical protein	89483061.69	328373852.4	3.669676095	COG0693	R	Putative intracellular protease/amidase	K03152	POORLY CHARACTERIZED
PA4336		conserved hypothetical protein	89483061.69	328373852.4	3.669676095	COG0693	R	Putative intracellular protease/amidase	K05520	POORLY CHARACTERIZED
PA4352		conserved hypothetical protein	841424609.1	242201104.1	0.287846471	COG0589	T	Universal stress protein UspA and related nucleotide-binding proteins	K06149	CELLULAR PROCESSES AND SIGNALING
PA4356	xenB	xenobiotic reductase	161771570	333754569.2	2.06312252	COG1902	C	"NADH:flavin oxidoreductases, Old Yellow Enzyme family"	K00219	METABOLISM
PA4356	xenB	xenobiotic reductase	161771570	333754569.2	2.06312252	COG1902	C	"NADH:flavin oxidoreductases, Old Yellow Enzyme family"	K00540	METABOLISM
PA4356	xenB	xenobiotic reductase	161771570	333754569.2	2.06312252	COG1902	C	"NADH:flavin oxidoreductases, Old Yellow Enzyme family"	K10680	METABOLISM
PA4366	sodB	superoxide dismutase	1798839575	100229643.8	5.57E-02	COG0605	P	Superoxide dismutase	K04564	METABOLISM
PA4369		hypothetical protein	78336274.29	50162681.31	0.640350614	COG1704	S	Uncharacterized conserved protein	K03744	POORLY CHARACTERIZED
PA4370	icmP	Insulin-cleaving metalloproteinase outer membrane protein precursor	61028906495	64698888037	1.060135135	COG3487	P	Uncharacterized iron-regulated protein	K07231	METABOLISM
PA4372		hypothetical protein	398580198.2	1045511425	2.62308923	COG3489	R	Predicted periplasmic lipoprotein	K07338	POORLY CHARACTERIZED
PA4385	groEL	GroEL protein	15550333261	17627035035	1.133547091	COG0459	O	Chaperonin GroEL (HSP60 family)	K04077	CELLULAR PROCESSES AND SIGNALING
PA4386	groES	GroES protein	79909699305	66733051428	0.835105776	COG0234	O	Co-chaperonin GroES (HSP10)	K04078	CELLULAR PROCESSES AND SIGNALING
PA4387		conserved hypothetical protein	7612942.505	0	0	COG3030	R	Protein affecting phage T7 exclusion by the F plasmid	K07113	POORLY CHARACTERIZED
PA4395		conserved hypothetical protein	2404375268	1446681298	0.601686982	COG1666	S	Uncharacterized protein conserved in bacteria	K09767	POORLY CHARACTERIZED
PA4403	secA	secretion protein SecA	64116633.97	147280555.3	2.297072479	COG0653	U	"Preprotein translocase subunit SecA (ATPase, RNA helicase)"	K03070	CELLULAR PROCESSES AND SIGNALING
PA4406	lpxC	UDP-3-O-acyl-N-acetylglucosamine deacetylase	625163800.9	96415330.46	0.154224109	COG0774	M	UDP-3-O-acyl-N-acetylglucosamine deacetylase	K02535	CELLULAR PROCESSES AND SIGNALING
PA4407	ftsZ	cell division protein FtsZ	284532415.4	471897755.2	1.658502616	COG0206	D	Cell division GTPase	K03531	CELLULAR PROCESSES AND SIGNALING
PA4408	ftsA	cell division protein FtsA	15083850.84	0	0	COG0849	D	Actin-like ATPase involved in cell division	K03590	CELLULAR PROCESSES AND SIGNALING
PA4423		conserved hypothetical protein	528535483.1	874582490.7	1.654728053	COG3107	R	Putative lipoprotein	K07121	POORLY CHARACTERIZED
PA4426		conserved hypothetical protein	28374642.02	0	0	COG2823	R	Predicted periplasmic or secreted lipoprotein	K04065	POORLY CHARACTERIZED
PA4427	sspB	stringent starvation protein B	0	8533767.1	1000	COG2969	R	Stringent starvation protein B	K03600	POORLY CHARACTERIZED
PA4429		probable cytochrome c1 precursor	2650919052	3984907981	1.503217527	COG2857	C	Cytochrome c1	K00413	METABOLISM
PA4429		probable cytochrome c1 precursor	2650919052	3984907981	1.503217527	COG2857	C	Cytochrome c1	K02275	METABOLISM
PA4431		probable iron-sulfur protein	2288020647	1798232299	0.785933598	COG0723	C	Rieske Fe-S protein	K03890	METABOLISM
PA4431		probable iron-sulfur protein	2288020647	1798232299	0.785933598	COG0723	C	Rieske Fe-S protein	K02636	METABOLISM
PA4431		probable iron-sulfur protein	2288020647	1798232299	0.785933598	COG0723	C	Rieske Fe-S protein	K00411	METABOLISM
PA4433	rplM	50S ribosomal protein L13	284392891.7	1418039285	4.986198061	COG0102	J	Ribosomal protein L13	K02871	INFORMATION STORAGE AND PROCESSING
PA4441		hypothetical protein	5961188642	6402636059	1.07405359	COG3105	S	Uncharacterized protein conserved in bacteria	K09908	POORLY CHARACTERIZED
PA4453		conserved hypothetical protein	85842550.28	0	0	COG2854	Q	"ABC-type transport system involved in resistance to organic solvents, auxilia"	K07323	METABOLISM
PA4457		arabinose-5-phosphate isomerase KdsD	17914799.95	0	0	COG0517	R	FOG: CBS domain	K06041	POORLY CHARACTERIZED
PA4457		arabinose-5-phosphate isomerase KdsD	17914799.95	0	0	COG0794	M	Predicted sugar phosphate isomerase involved in capsule formation	K02467	CELLULAR PROCESSES AND SIGNALING
PA4457		arabinose-5-phosphate isomerase KdsD	17914799.95	0	0	COG0517	R	FOG: CBS domain	K00088	POORLY CHARACTERIZED
PA4457		arabinose-5-phosphate isomerase KdsD	17914799.95	0	0	COG0794	M	Predicted sugar phosphate isomerase involved in capsule formation	K06041	CELLULAR PROCESSES AND SIGNALING
PA4457		arabinose-5-phosphate isomerase KdsD	17914799.95	0	0	COG0794	M	Predicted sugar phosphate isomerase involved in capsule formation	K08094	CELLULAR PROCESSES AND SIGNALING
PA4459	lptC	Lipopolysaccharide export system protein LptC	601604521.9	929746169.1	1.545444117	COG3117	S	Uncharacterized protein conserved in bacteria	K11719	POORLY CHARACTERIZED
PA4460	lptH	LptH	556758390.9	131698183.1	0.236544586	COG1934	S	Uncharacterized protein conserved in bacteria	K09774	POORLY CHARACTERIZED
PA4463		conserved hypothetical protein	31825250.82	38929156.05	1.223216001	COG1544	J	Ribosome-associated protein Y (PSrp-1)	K05808	INFORMATION STORAGE AND PROCESSING
PA4463		conserved hypothetical protein	31825250.82	38929156.05	1.223216001	COG1544	J	Ribosome-associated protein Y (PSrp-1)	K05809	INFORMATION STORAGE AND PROCESSING
PA4464	ptsN	nitrogen regulatory IIA protein	283991397.9	86680029.31	0.305220616	COG1762	T	Phosphotransferase system mannitol/fructose-specific IIA domain (Ntr-type)	K02770	CELLULAR PROCESSES AND SIGNALING
PA4464	ptsN	nitrogen regulatory IIA protein	283991397.9	86680029.31	0.305220616	COG1762	T	Phosphotransferase system mannitol/fructose-specific IIA domain (Ntr-type)	K02821	CELLULAR PROCESSES AND SIGNALING
PA4464	ptsN	nitrogen regulatory IIA protein	283991397.9	86680029.31	0.305220616	COG1762	T	Phosphotransferase system mannitol/fructose-specific IIA domain (Ntr-type)	K02769	CELLULAR PROCESSES AND SIGNALING
PA4464	ptsN	nitrogen regulatory IIA protein	283991397.9	86680029.31	0.305220616	COG1762	T	Phosphotransferase system mannitol/fructose-specific IIA domain (Ntr-type)	K02768	CELLULAR PROCESSES AND SIGNALING
PA4464	ptsN	nitrogen regulatory IIA protein	283991397.9	86680029.31	0.305220616	COG1762	T	Phosphotransferase system mannitol/fructose-specific IIA domain (Ntr-type)	K02806	CELLULAR PROCESSES AND SIGNALING
PA4464	ptsN	nitrogen regulatory IIA protein	283991397.9	86680029.31	0.305220616	COG1762	T	Phosphotransferase system mannitol/fructose-specific IIA domain (Ntr-type)	K02773	CELLULAR PROCESSES AND SIGNALING
PA4464	ptsN	nitrogen regulatory IIA protein	283991397.9	86680029.31	0.305220616	COG1762	G	Phosphotransferase system mannitol/fructose-specific IIA domain (Ntr-type)	K02769	METABOLISM
PA4464	ptsN	nitrogen regulatory IIA protein	283991397.9	86680029.31	0.305220616	COG1762	G	Phosphotransferase system mannitol/fructose-specific IIA domain (Ntr-type)	K02770	METABOLISM
PA4464	ptsN	nitrogen regulatory IIA protein	283991397.9	86680029.31	0.305220616	COG1762	G	Phosphotransferase system mannitol/fructose-specific IIA domain (Ntr-type)	K02806	METABOLISM
PA4464	ptsN	nitrogen regulatory IIA protein	283991397.9	86680029.31	0.305220616	COG1762	G	Phosphotransferase system mannitol/fructose-specific IIA domain (Ntr-type)	K02821	METABOLISM
PA4464	ptsN	nitrogen regulatory IIA protein	283991397.9	86680029.31	0.305220616	COG1762	G	Phosphotransferase system mannitol/fructose-specific IIA domain (Ntr-type)	K02773	METABOLISM
PA4464	ptsN	nitrogen regulatory IIA protein	283991397.9	86680029.31	0.305220616	COG1762	G	Phosphotransferase system mannitol/fructose-specific IIA domain (Ntr-type)	K02768	METABOLISM
PA4468	sodM	superoxide dismutase	25043746.99	48946067.49	1.954422695	COG0605	P	Superoxide dismutase	K04564	METABOLISM
PA4470	fumC1	fumarate hydratase	563408312	664602097.9	1.179610034	COG0114	C	Fumarase	K01679	METABOLISM
PA4473		hypothetical protein	1657209293	199507263.1	0.120387488	COG3028	S	Uncharacterized protein conserved in bacteria	K09889	POORLY CHARACTERIZED
PA4473		hypothetical protein	1657209293	199507263.1	0.120387488	COG3028	S	Uncharacterized protein conserved in bacteria	K09889	POORLY CHARACTERIZED
PA4473		hypothetical protein	1657209293	199507263.1	0.120387488	COG3028	S	Uncharacterized protein conserved in bacteria	K09889	POORLY CHARACTERIZED
PA4473		hypothetical protein	1657209293	199507263.1	0.120387488	COG3028	S	Uncharacterized protein conserved in bacteria	K09889	POORLY CHARACTERIZED
PA4481	mreB	rod shape-determining protein MreB	45530600.06	0	0	COG1077	D	Actin-like ATPase involved in cell morphogenesis	K03569	CELLULAR PROCESSES AND SIGNALING
PA4482	gatC	Glu-tRNA(Gln) amidotransferase subunit C	2951101781	4720759968	1.599660167	COG0721	J	Asp-tRNAAsn/Glu-tRNAGln amidotransferase C subunit	K02435	INFORMATION STORAGE AND PROCESSING
PA4484	gatB	Glu-tRNA(Gln) amidotransferase subunit B	51180970.91	45517976.2	0.889353511	COG0064	J	Asp-tRNAAsn/Glu-tRNAGln amidotransferase B subunit (PET112 homolog)	K02434	INFORMATION STORAGE AND PROCESSING
PA4487	magF	MagF	259733646.2	0	0					
PA4495		hypothetical protein	2982099282	1427489724	0.478686184					
PA4520		probable chemotaxis transducer	410781354.9	899531154.1	2.189805217	COG0840	N	Methyl-accepting chemotaxis protein	K03406	CELLULAR PROCESSES AND SIGNALING
PA4520		probable chemotaxis transducer	410781354.9	899531154.1	2.189805217	COG0840	T	Methyl-accepting chemotaxis protein	K03406	CELLULAR PROCESSES AND SIGNALING
PA4525	pilA	type 4 fimbrial precursor PilA	512468476.5	11134536129	21.72726058	COG4969	U	"Tfp pilus assembly protein, major pilin PilA"	K02655	CELLULAR PROCESSES AND SIGNALING
PA4525	pilA	type 4 fimbrial precursor PilA	512468476.5	11134536129	21.72726058	COG4969	N	"Tfp pilus assembly protein, major pilin PilA"	K02650	CELLULAR PROCESSES AND SIGNALING
PA4525	pilA	type 4 fimbrial precursor PilA	512468476.5	11134536129	21.72726058	COG4969	N	"Tfp pilus assembly protein, major pilin PilA"	K02682	CELLULAR PROCESSES AND SIGNALING
PA4525	pilA	type 4 fimbrial precursor PilA	512468476.5	11134536129	21.72726058	COG4969	U	"Tfp pilus assembly protein, major pilin PilA"	K02650	CELLULAR PROCESSES AND SIGNALING
PA4525	pilA	type 4 fimbrial precursor PilA	512468476.5	11134536129	21.72726058	COG4969	U	"Tfp pilus assembly protein, major pilin PilA"	K02682	CELLULAR PROCESSES AND SIGNALING
PA4525	pilA	type 4 fimbrial precursor PilA	512468476.5	11134536129	21.72726058	COG4969	N	"Tfp pilus assembly protein, major pilin PilA"	K02655	CELLULAR PROCESSES AND SIGNALING
PA4529	coaE	dephosphocoenzyme A kinase	73508700.85	0	0	COG0237	H	Dephospho-CoA kinase	K00859	METABOLISM
PA4542	clpB	ClpB protein	240026042.7	183949944.2	0.76637494	COG0542	O	"ATPases with chaperone activity, ATP-binding subunit"	K03694	CELLULAR PROCESSES AND SIGNALING
PA4542	clpB	ClpB protein	240026042.7	183949944.2	0.76637494	COG0542	O	"ATPases with chaperone activity, ATP-binding subunit"	K03696	CELLULAR PROCESSES AND SIGNALING
PA4542	clpB	ClpB protein	240026042.7	183949944.2	0.76637494	COG0542	O	"ATPases with chaperone activity, ATP-binding subunit"	K03695	CELLULAR PROCESSES AND SIGNALING
PA4542	clpB	ClpB protein	240026042.7	183949944.2	0.76637494	COG0542	O	"ATPases with chaperone activity, ATP-binding subunit"	K11907	CELLULAR PROCESSES AND SIGNALING
PA4550	fimU	type 4 fimbrial biogenesis protein FimU	946891595.1	0	0	COG4970	N	Tfp pilus assembly protein FimT	K08084	CELLULAR PROCESSES AND SIGNALING
PA4550	fimU	type 4 fimbrial biogenesis protein FimU	946891595.1	0	0	COG4970	U	Tfp pilus assembly protein FimT	K02457	CELLULAR PROCESSES AND SIGNALING
PA4550	fimU	type 4 fimbrial biogenesis protein FimU	946891595.1	0	0	COG4970	N	Tfp pilus assembly protein FimT	K02457	CELLULAR PROCESSES AND SIGNALING
PA4550	fimU	type 4 fimbrial biogenesis protein FimU	946891595.1	0	0	COG4970	U	Tfp pilus assembly protein FimT	K08084	CELLULAR PROCESSES AND SIGNALING
PA4550	fimU	type 4 fimbrial biogenesis protein FimU	946891595.1	0	0	COG4970	N	Tfp pilus assembly protein FimT	K08085	CELLULAR PROCESSES AND SIGNALING
PA4550	fimU	type 4 fimbrial biogenesis protein FimU	946891595.1	0	0	COG4970	U	Tfp pilus assembly protein FimT	K08085	CELLULAR PROCESSES AND SIGNALING
PA4553	pilX	type 4 fimbrial biogenesis protein PilX	23065481.05	0	0	COG4726	N	Tfp pilus assembly protein PilX	K02673	CELLULAR PROCESSES AND SIGNALING
PA4553	pilX	type 4 fimbrial biogenesis protein PilX	23065481.05	0	0	COG4726	U	Tfp pilus assembly protein PilX	K02673	CELLULAR PROCESSES AND SIGNALING
PA4557	lytB	LytB protein	18416624.4	0	0	COG0761	I	Penicillin tolerance protein	K03527	METABOLISM
PA4557	lytB	LytB protein	18416624.4	0	0	COG0761	M	Penicillin tolerance protein	K03527	CELLULAR PROCESSES AND SIGNALING
PA4558		"probable peptidyl-prolyl cis-trans isomerase	813887707.4	674300010.3	0.828492683	COG1047	O	FKBP-type peptidyl-prolyl cis-trans isomerases 2	K01802	CELLULAR PROCESSES AND SIGNALING
PA4558		"probable peptidyl-prolyl cis-trans isomerase	813887707.4	674300010.3	0.828492683	COG1047	O	FKBP-type peptidyl-prolyl cis-trans isomerases 2	K03775	CELLULAR PROCESSES AND SIGNALING
PA4558		"probable peptidyl-prolyl cis-trans isomerase	813887707.4	674300010.3	0.828492683	COG1047	O	FKBP-type peptidyl-prolyl cis-trans isomerases 2	K03774	CELLULAR PROCESSES AND SIGNALING
PA4563	rpsT	30S ribosomal protein S20	2554798792	865178523.9	0.3386484	COG0268	J	Ribosomal protein S20	K02968	INFORMATION STORAGE AND PROCESSING
PA4572	fklB	peptidyl-prolyl cis-trans isomerase FklB	968295769.9	730326688	0.754239263	COG0545	O	FKBP-type peptidyl-prolyl cis-trans isomerases 1	K03773	CELLULAR PROCESSES AND SIGNALING
PA4572	fklB	peptidyl-prolyl cis-trans isomerase FklB	968295769.9	730326688	0.754239263	COG0545	O	FKBP-type peptidyl-prolyl cis-trans isomerases 1	K01802	CELLULAR PROCESSES AND SIGNALING
PA4572	fklB	peptidyl-prolyl cis-trans isomerase FklB	968295769.9	730326688	0.754239263	COG0545	O	FKBP-type peptidyl-prolyl cis-trans isomerases 1	K09575	CELLULAR PROCESSES AND SIGNALING
PA4572	fklB	peptidyl-prolyl cis-trans isomerase FklB	968295769.9	730326688	0.754239263	COG0545	O	FKBP-type peptidyl-prolyl cis-trans isomerases 1	K03772	CELLULAR PROCESSES AND SIGNALING
PA4577		hypothetical protein	1145403000	674577317.6	0.588943208	COG1734	T	DnaK suppressor protein	K06204	CELLULAR PROCESSES AND SIGNALING
PA4587	ccpR	cytochrome c551 peroxidase precursor	207967256.1	0	0	COG1858	P	Cytochrome c peroxidase	K00428	METABOLISM
PA4595		probable ATP-binding component of ABC transporter	22562802.72	2817651981	124.8804068	COG0488	R	ATPase components of ABC transporters with duplicated ATPase domains	K06158	POORLY CHARACTERIZED
PA4611		hypothetical protein	16118623624	16066914037	0.996791935	COG2841	S	Uncharacterized protein conserved in bacteria	K09794	POORLY CHARACTERIZED
PA4626	hprA	glycerate dehydrogenase	9012347.064	0	0	COG1052	R	Lactate dehydrogenase and related dehydrogenases	K00090	POORLY CHARACTERIZED
PA4626	hprA	glycerate dehydrogenase	9012347.064	0	0	COG1052	R	Lactate dehydrogenase and related dehydrogenases	K00018	POORLY CHARACTERIZED
PA4626	hprA	glycerate dehydrogenase	9012347.064	0	0	COG1052	R	Lactate dehydrogenase and related dehydrogenases	K03778	POORLY CHARACTERIZED
PA4626	hprA	glycerate dehydrogenase	9012347.064	0	0	COG1052	H	Lactate dehydrogenase and related dehydrogenases	K00015	METABOLISM
PA4626	hprA	glycerate dehydrogenase	9012347.064	0	0	COG1052	H	Lactate dehydrogenase and related dehydrogenases	K00090	METABOLISM
PA4626	hprA	glycerate dehydrogenase	9012347.064	0	0	COG1052	H	Lactate dehydrogenase and related dehydrogenases	K00018	METABOLISM
PA4626	hprA	glycerate dehydrogenase	9012347.064	0	0	COG1052	C	Lactate dehydrogenase and related dehydrogenases	K03778	METABOLISM
PA4626	hprA	glycerate dehydrogenase	9012347.064	0	0	COG1052	C	Lactate dehydrogenase and related dehydrogenases	K00018	METABOLISM
PA4626	hprA	glycerate dehydrogenase	9012347.064	0	0	COG1052	C	Lactate dehydrogenase and related dehydrogenases	K00015	METABOLISM
PA4626	hprA	glycerate dehydrogenase	9012347.064	0	0	COG1052	R	Lactate dehydrogenase and related dehydrogenases	K00015	POORLY CHARACTERIZED
PA4626	hprA	glycerate dehydrogenase	9012347.064	0	0	COG1052	C	Lactate dehydrogenase and related dehydrogenases	K00090	METABOLISM
PA4626	hprA	glycerate dehydrogenase	9012347.064	0	0	COG1052	H	Lactate dehydrogenase and related dehydrogenases	K03778	METABOLISM
PA4632		hypothetical protein	349696416.9	911286495.1	2.605936038	COG0501	O	Zn-dependent protease with chaperone function	K03799	CELLULAR PROCESSES AND SIGNALING
PA4632		hypothetical protein	349696416.9	911286495.1	2.605936038	COG0501	O	Zn-dependent protease with chaperone function	K07387	CELLULAR PROCESSES AND SIGNALING
PA4632		hypothetical protein	349696416.9	911286495.1	2.605936038	COG0501	O	Zn-dependent protease with chaperone function	K06013	CELLULAR PROCESSES AND SIGNALING
PA4633		probable chemotaxis transducer	469964192.9	899531154.1	1.914041894	COG0840	T	Methyl-accepting chemotaxis protein	K03406	CELLULAR PROCESSES AND SIGNALING
PA4633		probable chemotaxis transducer	469964192.9	899531154.1	1.914041894	COG0840	N	Methyl-accepting chemotaxis protein	K03406	CELLULAR PROCESSES AND SIGNALING
PA4639		hypothetical protein	55225708.45	0	0					
PA4642		hypothetical protein	2819741951	1537606519	0.545300437					
PA4659		probable transcriptional regulator	1020820404	808223440.7	0.791739112					
PA4661	pagL	Lipid A 3-O-deacylase	1866080807	1914513921	1.025954457					
PA4665	prfA	peptide chain release factor 1	16494629.07	0	0	COG0216	J	Protein chain release factor A	K02835	INFORMATION STORAGE AND PROCESSING
PA4671		probable ribosomal protein L25	523624400.4	425859302	0.813291553	COG1825	J	Ribosomal protein L25 (general stress protein Ctc)	K02897	INFORMATION STORAGE AND PROCESSING
PA4687	hitA	ferric iron-binding periplasmic protein HitA	87699309.29	0	0	COG1840	P	"ABC-type Fe3 transport system, periplasmic component"	K02012	METABOLISM
PA4687	hitA	ferric iron-binding periplasmic protein HitA	87699309.29	0	0	COG1840	P	"ABC-type Fe3 transport system, periplasmic component"	K02055	METABOLISM
PA4694	ilvC	ketol-acid reductoisomerase	635578558.2	464937152.1	0.731517994	COG0059	H	Ketol-acid reductoisomerase	K00053	METABOLISM
PA4694	ilvC	ketol-acid reductoisomerase	635578558.2	464937152.1	0.731517994	COG0059	E	Ketol-acid reductoisomerase	K00053	METABOLISM
PA4699		hypothetical protein	1050323618	1362591223	1.297306088	COG3071	H	Uncharacterized enzyme of heme biosynthesis	K02498	METABOLISM
PA4702		hypothetical protein	1022852635	0	0					
PA4708	phuT	"Heme-transport protein	198123145.9	130708674.4	0.659734499	COG4558	P	"ABC-type hemin transport system, periplasmic component"	K02016	METABOLISM
PA4723	dksA	suppressor protein DksA	4643828155	4349171128	0.93654868	COG1734	T	DnaK suppressor protein	K06204	CELLULAR PROCESSES AND SIGNALING
PA4738		conserved hypothetical protein	2890091301	2726954344	0.943553009					
PA4739		conserved hypothetical protein	8439976015	2823043217	0.334484744	COG2823	R	Predicted periplasmic or secreted lipoprotein	K04065	POORLY CHARACTERIZED
PA4741	rpsO	30S ribosomal protein S15	8941270209	6154902198	0.688370003	COG0184	J	Ribosomal protein S15P/S13E	K02956	INFORMATION STORAGE AND PROCESSING
PA4744	infB	translation initiation factor IF-2	272637288.8	658033645.5	2.413586374	COG0532	J	Translation initiation factor 2 (IF-2; GTPase)	K03243	INFORMATION STORAGE AND PROCESSING
PA4744	infB	translation initiation factor IF-2	272637288.8	658033645.5	2.413586374	COG0532	J	Translation initiation factor 2 (IF-2; GTPase)	K02519	INFORMATION STORAGE AND PROCESSING
PA4745	nusA	N utilization substance protein A	253194729.3	100184414	0.395681278	COG0195	K	Transcription elongation factor	K02600	INFORMATION STORAGE AND PROCESSING
PA4747	secG	secretion protein SecG	78695074.08	245610740.1	3.121043382	COG1314	U	Preprotein translocase subunit SecG	K03075	CELLULAR PROCESSES AND SIGNALING
PA4748	tpiA	triosephosphate isomerase	119034092.2	0	0	COG0149	G	Triosephosphate isomerase	K01803	METABOLISM
PA4751	ftsH	cell division protein FtsH	3011787787	1134288949	0.376616491	COG0465	O	ATP-dependent Zn proteases	K03798	CELLULAR PROCESSES AND SIGNALING
PA4753		conserved hypothetical protein	260791601.8	30839051.86	0.118251706	COG1534	J	"Predicted RNA-binding protein containing KH domain, possibly ribosomal protei"	K07574	INFORMATION STORAGE AND PROCESSING
PA4755	greA	transcription elongation factor GreA	6965899773	5498479612	0.789342338	COG0782	K	Transcription elongation factor	K04760	INFORMATION STORAGE AND PROCESSING
PA4755	greA	transcription elongation factor GreA	6965899773	5498479612	0.789342338	COG0782	K	Transcription elongation factor	K06140	INFORMATION STORAGE AND PROCESSING
PA4755	greA	transcription elongation factor GreA	6965899773	5498479612	0.789342338	COG0782	K	Transcription elongation factor	K03624	INFORMATION STORAGE AND PROCESSING

PA4758	carA	carbamoyl-phosphate synthase small chain	181327695.4	0	0	COG0505	E	Carbamoylphosphate synthase small subunit	K01956	METABOLISM
PA4758	carA	carbamoyl-phosphate synthase small chain	181327695.4	0	0	COG0505	F	Carbamoylphosphate synthase small subunit	K01956	METABOLISM
PA4760	dnaJ	DnaJ protein	10079268.37	0	0	COG0484	O	DnaJ-class molecular chaperone with C-terminal Zn finger domain	K03686	CELLULAR PROCESSES AND SIGNALING
PA4761	dnaK	DnaK protein	12599055581	12425363105	0.98621385	COG0443	O	Molecular chaperone	K04046	CELLULAR PROCESSES AND SIGNALING
PA4761	dnaK	DnaK protein	12599055581	12425363105	0.98621385	COG0443	O	Molecular chaperone	K04044	CELLULAR PROCESSES AND SIGNALING
PA4761	dnaK	DnaK protein	12599055581	12425363105	0.98621385	COG0443	O	Molecular chaperone	K04043	CELLULAR PROCESSES AND SIGNALING
PA4761	dnaK	DnaK protein	12599055581	12425363105	0.98621385	COG0443	O	Molecular chaperone	K03283	CELLULAR PROCESSES AND SIGNALING
PA4762	grpE	heat shock protein GrpE	19872974127	11218072546	0.564488862	COG0576	O	Molecular chaperone GrpE (heat shock protein)	K03687	CELLULAR PROCESSES AND SIGNALING
PA4764	fur	ferric uptake regulation protein	1529074.563	4900917.879	3.205152972	COG0735	P	Fe2? uptake regulation proteins	K09825	METABOLISM
PA4764	fur	ferric uptake regulation protein	1529074.563	4900917.879	3.205152972	COG0735	P	Fe2? uptake regulation proteins	K09823	METABOLISM
PA4764	fur	ferric uptake regulation protein	1529074.563	4900917.879	3.205152972	COG0735	P	Fe2? uptake regulation proteins	K03711	METABOLISM
PA4765	omlA	Outer membrane lipoprotein OmlA precursor	291153987.8	571419309.3	1.962601693	COG2913	J	Small protein A (tmRNA-binding)	K06186	INFORMATION STORAGE AND PROCESSING
PA4778	cueR	CueR	458879482.5	313527396.2	0.683245619					
PA4845	dipZ	thiol:disulfide interchange protein DipZ	45381854.4	53595553.75	1.180990827	COG4232	C	Thiol:disulfide interchange protein	K04084	METABOLISM
PA4845	dipZ	thiol:disulfide interchange protein DipZ	45381854.4	53595553.75	1.180990827	COG4232	O	Thiol:disulfide interchange protein	K04084	CELLULAR PROCESSES AND SIGNALING
PA4847	accB	biotin carboxyl carrier protein (BCCP)	258228855.9	507762666.2	1.966328141	COG0511	I	Biotin carboxyl carrier protein	K01571	METABOLISM
PA4847	accB	biotin carboxyl carrier protein (BCCP)	258228855.9	507762666.2	1.966328141	COG0511	I	Biotin carboxyl carrier protein	K02160	METABOLISM
PA4847	accB	biotin carboxyl carrier protein (BCCP)	258228855.9	507762666.2	1.966328141	COG0511	I	Biotin carboxyl carrier protein	K01960	METABOLISM
PA4851		hypothetical protein	387171526.2	380429490.3	0.982586437					
PA4853	fis	DNA-binding protein Fis	807967852.4	696818888.6	0.862433928	COG2901	L	"Factor for inversion stimulation Fis, transcriptional activator"	K03557	INFORMATION STORAGE AND PROCESSING
PA4853	fis	DNA-binding protein Fis	807967852.4	696818888.6	0.862433928	COG2901	K	"Factor for inversion stimulation Fis, transcriptional activator"	K03557	INFORMATION STORAGE AND PROCESSING
PA4853	fis	DNA-binding protein Fis	807967852.4	696818888.6	0.862433928	COG2901	L	"Factor for inversion stimulation Fis, transcriptional activator"	K03557	INFORMATION STORAGE AND PROCESSING
PA4853	fis	DNA-binding protein Fis	807967852.4	696818888.6	0.862433928	COG2901	K	"Factor for inversion stimulation Fis, transcriptional activator"	K03557	INFORMATION STORAGE AND PROCESSING
PA4853	fis	DNA-binding protein Fis	807967852.4	696818888.6	0.862433928	COG2901	K	"Factor for inversion stimulation Fis, transcriptional activator"	K03557	INFORMATION STORAGE AND PROCESSING
PA4853	fis	DNA-binding protein Fis	807967852.4	696818888.6	0.862433928	COG2901	L	"Factor for inversion stimulation Fis, transcriptional activator"	K03557	INFORMATION STORAGE AND PROCESSING
PA4853	fis	DNA-binding protein Fis	807967852.4	696818888.6	0.862433928	COG2901	L	"Factor for inversion stimulation Fis, transcriptional activator"	K03557	INFORMATION STORAGE AND PROCESSING
PA4853	fis	DNA-binding protein Fis	807967852.4	696818888.6	0.862433928	COG2901	K	"Factor for inversion stimulation Fis, transcriptional activator"	K03557	INFORMATION STORAGE AND PROCESSING
PA4865	ureA	urease gamma subunit	68639167.82	74798389.22	1.089733334	COG0831	E	Urea amidohydrolase (urease) gamma subunit	K14048	METABOLISM
PA4865	ureA	urease gamma subunit	68639167.82	74798389.22	1.089733334	COG0831	E	Urea amidohydrolase (urease) gamma subunit	K01430	METABOLISM
PA4867	ureB	urease beta subunit	217561692.2	143707308	0.660535899	COG0832	E	Urea amidohydrolase (urease) beta subunit	K14048	METABOLISM
PA4867	ureB	urease beta subunit	217561692.2	143707308	0.660535899	COG0832	E	Urea amidohydrolase (urease) beta subunit	K01429	METABOLISM
PA4874		conserved hypothetical protein	38926873.75	122769654.8	3.153853443					
PA4876	osmE	osmotically inducible lipoprotein OsmE	2782410379	4383889039	1.575572415	COG2913	J	Small protein A (tmRNA-binding)	K06186	INFORMATION STORAGE AND PROCESSING
PA4880		probable bacterioferritin	1444430431	1971686068	1.365026675	COG2193	P	Bacterioferritin (cytochrome b1)	K03594	METABOLISM
PA4885	irlR	two-component response regulator	84135988.51	294473519.8	3.499971	COG0745	K	Response regulators consisting of a CheY-like receiver domain and a winged-he	K02483	INFORMATION STORAGE AND PROCESSING
PA4885	irlR	two-component response regulator	84135988.51	294473519.8	3.499971	COG0745	T	Response regulators consisting of a CheY-like receiver domain and a winged-he	K02483	CELLULAR PROCESSES AND SIGNALING
PA4915		probable chemotaxis transducer	540493626	913040320	1.689271207	COG0840	T	Methyl-accepting chemotaxis protein	K03406	CELLULAR PROCESSES AND SIGNALING
PA4915		probable chemotaxis transducer	540493626	913040320	1.689271207	COG0840	N	Methyl-accepting chemotaxis protein	K03406	CELLULAR PROCESSES AND SIGNALING
PA4920	nadE	NH3-dependent NAD synthetase	1533873361	0	0	COG0171	H	NAD synthase	K01916	METABOLISM
PA4920	nadE	NH3-dependent NAD synthetase	1533873361	0	0	COG0171	H	NAD synthase	K01950	METABOLISM
PA4922	azu	azurin precursor	26407664353	8092252848	0.306435766					
PA4932	rplI	50S ribosomal protein L9	2098305900	3664315944	1.746321136	COG0359	J	Ribosomal protein L9	K02939	INFORMATION STORAGE AND PROCESSING
PA4934	rpsR	30S ribosomal protein S18	59198600.64	0	0	COG0238	J	Ribosomal protein S18	K02963	INFORMATION STORAGE AND PROCESSING
PA4935	rpsF	30S ribosomal protein S6	28047410121	24409960688	0.870310684	COG0360	J	Ribosomal protein S6	K02990	INFORMATION STORAGE AND PROCESSING
PA4942	hflK	protease subunit HflK	13385554620	11620961724	0.86817185	COG0330	O	"Membrane protease subunits, stomatin/prohibitin homologs"	K04087	CELLULAR PROCESSES AND SIGNALING
PA4942	hflK	protease subunit HflK	13385554620	11620961724	0.86817185	COG0330	O	"Membrane protease subunits, stomatin/prohibitin homologs"	K04088	CELLULAR PROCESSES AND SIGNALING
PA4944	hfq	Hfq	4203082492	5599127798	1.332147967	COG1923	R	Uncharacterized host factor I protein	K03666	POORLY CHARACTERIZED
PA4951	orn	oligoribonuclease	9243918.94	0	0	COG1949	A	Oligoribonuclease (3'->5' exoribonuclease)	K13288	INFORMATION STORAGE AND PROCESSING
PA4953	motB	chemotaxis protein MotB	122227342	0	0	COG1360	N	Flagellar motor protein	K11892	CELLULAR PROCESSES AND SIGNALING
PA4953	motB	chemotaxis protein MotB	122227342	0	0	COG1360	N	Flagellar motor protein	K02557	CELLULAR PROCESSES AND SIGNALING
PA4956	rhdA	thiosulfate:cyanide sulfurtransferase	26775839.73	0	0	COG2897	P	Rhodanese-related sulfurtransferase	K01011	METABOLISM
PA4963		hypothetical protein	93720007.3	109561032.6	1.169025011	COG3009	S	Uncharacterized protein conserved in bacteria	K09857	POORLY CHARACTERIZED
PA4974		probable outer membrane protein precursor	3554237222	4024989396	1.132448158	COG1538	U	Outer membrane protein	K12340	CELLULAR PROCESSES AND SIGNALING
PA4974		probable outer membrane protein precursor	3554237222	4024989396	1.132448158	COG1538	M	Outer membrane protein	K03287	CELLULAR PROCESSES AND SIGNALING
PA4974		probable outer membrane protein precursor	3554237222	4024989396	1.132448158	COG1538	M	Outer membrane protein	K12340	CELLULAR PROCESSES AND SIGNALING
PA4974		probable outer membrane protein precursor	3554237222	4024989396	1.132448158	COG1538	U	Outer membrane protein	K03287	CELLULAR PROCESSES AND SIGNALING
PA5015	aceE	pyruvate dehydrogenase	14428157.47	71420385.35	4.950069717	COG2609	C	"Pyruvate dehydrogenase complex, dehydrogenase (E1) component"	K00163	METABOLISM
PA5016	aceF	dihydrolipoamide acetyltransferase	850274121.5	686101537.3	0.806918051	COG0508	C	"Pyruvate/2-oxoglutarate dehydrogenase complex, dihydrolipoamide acyltransfera"	K00658	METABOLISM
PA5016	aceF	dihydrolipoamide acetyltransferase	850274121.5	686101537.3	0.806918051	COG0508	C	"Pyruvate/2-oxoglutarate dehydrogenase complex, dihydrolipoamide acyltransfera"	K09699	METABOLISM
PA5016	aceF	dihydrolipoamide acetyltransferase	850274121.5	686101537.3	0.806918051	COG0508	C	"Pyruvate/2-oxoglutarate dehydrogenase complex, dihydrolipoamide acyltransfera"	K00627	METABOLISM
PA5027		hypothetical protein	53572941.03	116178348.5	2.168601279	COG0589	T	Universal stress protein UspA and related nucleotide-binding proteins	K06149	CELLULAR PROCESSES AND SIGNALING
PA5041	pilP	type 4 fimbrial biogenesis protein PilP	43940763.49	57426601.16	1.306909498	COG3168	N	Tfp pilus assembly protein PilP	K02665	CELLULAR PROCESSES AND SIGNALING
PA5041	pilP	type 4 fimbrial biogenesis protein PilP	43940763.49	57426601.16	1.306909498	COG3168	U	Tfp pilus assembly protein PilP	K02665	CELLULAR PROCESSES AND SIGNALING
PA5042	pilO	type 4 fimbrial biogenesis protein PilO	32348422.24	0	0	COG3167	U	Tfp pilus assembly protein PilO	K02664	CELLULAR PROCESSES AND SIGNALING
PA5042	pilO	type 4 fimbrial biogenesis protein PilO	32348422.24	0	0	COG3167	N	Tfp pilus assembly protein PilO	K02664	CELLULAR PROCESSES AND SIGNALING
PA5046		malic enzyme	44073712.39	288574525.3	6.547542961	COG0281	C	Malic enzyme	K00027	METABOLISM
PA5046		malic enzyme	44073712.39	288574525.3	6.547542961	COG0281	C	Malic enzyme	K00029	METABOLISM
PA5049	rpmE	50S ribosomal protein L31	2870801330	3839830393	1.337546542	COG0254	J	Ribosomal protein L31	K02909	INFORMATION STORAGE AND PROCESSING
PA5054	hslU	heat shock protein HslU	136227194	245249380.1	1.80029679	COG1220	O	"ATP-dependent protease HslVU (ClpYQ), ATPase subunit"	K03667	CELLULAR PROCESSES AND SIGNALING
PA5060	phaF	polyhydroxyalkanoate synthesis protein PhaF	3700764497	8469842999	2.288673869					
PA5061		conserved hypothetical protein	62176109.47	0	0					
PA5062		conserved hypothetical protein	2854319.765	0	0					
PA5063	ubiE	ubiquinone biosynthesis methyltransferase UbiE	268663935.5	425147578.6	1.582451242	COG2226	H	Methylase involved in ubiquinone/menaquinone biosynthesis	K03183	METABOLISM
PA5064		hypothetical protein	1040575739	77033562.11	7.40E-02	COG3165	S	Uncharacterized protein conserved in bacteria	K03690	POORLY CHARACTERIZED
PA5065	ubiB	ubiquinone biosynthetic protein UbiB	548058563.4	688481216	1.256218335	COG0661	R	Predicted unusual protein kinase	K03688	POORLY CHARACTERIZED
PA5065	ubiB	ubiquinone biosynthetic protein UbiB	548058563.4	688481216	1.256218335	COG0661	R	Predicted unusual protein kinase	K08869	POORLY CHARACTERIZED
PA5067	hisE	phosphoribosyl-ATP pyrophosphohydrolase	309061017.4	0	0	COG0140	E	Phosphoribosyl-ATP pyrophosphohydrolase	K01523	METABOLISM
PA5067	hisE	phosphoribosyl-ATP pyrophosphohydrolase	309061017.4	0	0	COG0140	E	Phosphoribosyl-ATP pyrophosphohydrolase	K11755	METABOLISM
PA5068	tatA	translocation protein TatA	907872758.6	864774037.8	0.952527796	COG1826	U	Sec-independent protein secretion pathway components	K03425	CELLULAR PROCESSES AND SIGNALING
PA5068	tatA	translocation protein TatA	907872758.6	864774037.8	0.952527796	COG1826	U	Sec-independent protein secretion pathway components	K03116	CELLULAR PROCESSES AND SIGNALING
PA5068	tatA	translocation protein TatA	907872758.6	864774037.8	0.952527796	COG1826	U	Sec-independent protein secretion pathway components	K03117	CELLULAR PROCESSES AND SIGNALING
PA5069	tatB	translocation protein TatB	16049416.76	0	0					
PA5072	mcpK	McpK	410781354.9	899531154.1	2.189805217	COG0840	N	Methyl-accepting chemotaxis protein	K03406	CELLULAR PROCESSES AND SIGNALING
PA5072	mcpK	McpK	410781354.9	899531154.1	2.189805217	COG0840	T	Methyl-accepting chemotaxis protein	K03406	CELLULAR PROCESSES AND SIGNALING
PA5077	opgH	OpgH	11979575.2	9826430.042	0.820265317	COG2943	M	Membrane glycosyltransferase	K03669	CELLULAR PROCESSES AND SIGNALING
PA5082	dguC	DguC	89074053.84	0	0	COG0834	T	"ABC-type amino acid transport/signal transduction systems, periplasmic compon"	K02030	CELLULAR PROCESSES AND SIGNALING
PA5082	dguC	DguC	89074053.84	0	0	COG0834	E	"ABC-type amino acid transport/signal transduction systems, periplasmic compon"	K02030	METABOLISM
PA5108		hypothetical protein	348924681.4	523297896.2	1.49974457					
PA5110	fbp	"fructose-1	38382696.91	0	0	COG0158	G	"Fructose-1,6-bisphosphatase"	K03841	METABOLISM
PA5128	secB	secretion protein SecB	60596007.12	79574843.95	1.313202763	COG1952	U	Preprotein translocase subunit SecB	K03071	CELLULAR PROCESSES AND SIGNALING
PA5130		conserved hypothetical protein	6857295807	1535635725	0.223941882	COG0607	P	Rhodanese-related sulfurtransferase	K03151	METABOLISM
PA5133		conserved hypothetical protein	90665256.61	31602794.26	0.348565652					
PA5134	ctpA	"carboxyl-terminal processing protease	7341784.817	42650428.04	5.809272418	COG0793	M	Periplasmic protease	K03797	CELLULAR PROCESSES AND SIGNALING
PA5146		hypothetical protein	22584832.79	0	0	COG2982	M	Uncharacterized protein involved in outer membrane biogenesis	K07290	CELLULAR PROCESSES AND SIGNALING
PA5146		hypothetical protein	22584832.79	0	0	COG2982	M	Uncharacterized protein involved in outer membrane biogenesis	K07289	CELLULAR PROCESSES AND SIGNALING
PA5148		conserved hypothetical protein	2698901052	1288292006	0.477339473					
PA5171	arcA	arginine deiminase	8725529.242	0	0	COG2235	E	Arginine deiminase	K01478	METABOLISM
PA5172	arcB	"ornithine carbamoyltransferase	417450146.7	6207264.499	1.49E-02	COG0078	E	Ornithine carbamoyltransferase	K00611	METABOLISM
PA5173	arcC	carbamate kinase	15569707.77	116995735.5	7.514318009	COG0549	E	Carbamate kinase	K00926	METABOLISM
PA5178		conserved hypothetical protein	22374334634	21115780112	0.943750081					
PA5200	amgR	AmgR	29555043.79	0	0	COG0745	K	Response regulators consisting of a CheY-like receiver domain and a winged-he	K02483	INFORMATION STORAGE AND PROCESSING
PA5200	amgR	AmgR	29555043.79	0	0	COG0745	T	Response regulators consisting of a CheY-like receiver domain and a winged-he	K02483	CELLULAR PROCESSES AND SIGNALING
PA5201		conserved hypothetical protein	43006193.37	102016841.2	2.372143015	COG2183	K	Transcriptional accessory protein	K06959	INFORMATION STORAGE AND PROCESSING
PA5201		conserved hypothetical protein	43006193.37	102016841.2	2.372143015	COG2183	K	Transcriptional accessory protein	K11292	INFORMATION STORAGE AND PROCESSING
PA5207		probable phosphate transporter	0	2324226769	1000	COG0306	P	Phosphate/sulphate permeases	K03306	METABOLISM
PA5210		probable secretion pathway ATPase	108838781.8	0	0	COG2804	U	"Type II secretory pathway, ATPase PulE/Tfp pilus assembly pathway, ATPase PilI"	K02504	CELLULAR PROCESSES AND SIGNALING
PA5210		probable secretion pathway ATPase	108838781.8	0	0	COG2804	N	"Type II secretory pathway, ATPase PulE/Tfp pilus assembly pathway, ATPase PilI"	K02504	CELLULAR PROCESSES AND SIGNALING
PA5210		probable secretion pathway ATPase	108838781.8	0	0	COG2804	N	"Type II secretory pathway, ATPase PulE/Tfp pilus assembly pathway, ATPase PilI"	K02243	CELLULAR PROCESSES AND SIGNALING
PA5210		probable secretion pathway ATPase	108838781.8	0	0	COG2804	N	"Type II secretory pathway, ATPase PulE/Tfp pilus assembly pathway, ATPase PilI"	K02454	CELLULAR PROCESSES AND SIGNALING
PA5210		probable secretion pathway ATPase	108838781.8	0	0	COG2804	U	"Type II secretory pathway, ATPase PulE/Tfp pilus assembly pathway, ATPase PilI"	K02243	CELLULAR PROCESSES AND SIGNALING
PA5210		probable secretion pathway ATPase	108838781.8	0	0	COG2804	U	"Type II secretory pathway, ATPase PulE/Tfp pilus assembly pathway, ATPase PilI"	K02652	CELLULAR PROCESSES AND SIGNALING
PA5210		probable secretion pathway ATPase	108838781.8	0	0	COG2804	U	"Type II secretory pathway, ATPase PulE/Tfp pilus assembly pathway, ATPase PilI"	K02454	CELLULAR PROCESSES AND SIGNALING
PA5210		probable secretion pathway ATPase	108838781.8	0	0	COG2804	N	"Type II secretory pathway, ATPase PulE/Tfp pilus assembly pathway, ATPase PilI"	K02652	CELLULAR PROCESSES AND SIGNALING
PA5214	gcvH1	glycine cleavage system protein H1	1343011854	132181845.2	9.84E-02	COG0509	E	Glycine cleavage system H protein (lipoate-binding)	K02437	METABOLISM
PA5225		hypothetical protein	47151773.97	49941055.44	1.059155388	COG3079	S	Uncharacterized protein conserved in bacteria	K09895	POORLY CHARACTERIZED
PA5226		hypothetical protein	69559597.72	396842923.7	5.7050779					
PA5227		conserved hypothetical protein	1517226997	515780957.4	0.339949762	COG3027	S	Uncharacterized protein conserved in bacteria	K09888	POORLY CHARACTERIZED
PA5233		hypothetical protein	688740185.9	19887615.01	2.89E-02	COG1580	N	Flagellar basal body-associated protein	K02415	CELLULAR PROCESSES AND SIGNALING
PA5239	rho	transcription termination factor Rho	354158786.4	27544641.79	0.077774837	COG1158	K	Transcription termination factor	K03628	INFORMATION STORAGE AND PROCESSING
PA5240	trxA	thioredoxin	11125474588	10823789455	0.972883392	COG3118	O	Thioredoxin domain-containing protein	K05838	CELLULAR PROCESSES AND SIGNALING
PA5245		conserved hypothetical protein	134419703.8	93205738.15	0.69339342					
PA5253	algP	alginate regulatory protein AlgP	2808442576	3609146145	1.285105908					
PA5258		hypothetical protein	992761099.5	899286741.3	0.905844056	COG2959	H	Uncharacterized enzyme of heme biosynthesis	K13543	METABOLISM
PA5258		hypothetical protein	992761099.5	899286741.3	0.905844056	COG2959	H	Uncharacterized enzyme of heme biosynthesis	K02496	METABOLISM
PA5269		hypothetical protein	418361747.5	230794214	0.551661846					
PA5271		hypothetical protein	34554417.6	78099282.54	2.260182285					
PA5274	rnk	nucleoside diphosphate kinase regulator	62501659.23	86356536.07	1.381667897	COG0782	K	Transcription elongation factor	K06140	INFORMATION STORAGE AND PROCESSING
PA5274	rnk	nucleoside diphosphate kinase regulator	62501659.23	86356536.07	1.381667897	COG0782	K	Transcription elongation factor	K03624	INFORMATION STORAGE AND PROCESSING
PA5274	rnk	nucleoside diphosphate kinase regulator	62501659.23	86356536.07	1.381667897	COG0782	K	Transcription elongation factor	K04760	INFORMATION STORAGE AND PROCESSING
PA5275		conserved hypothetical protein	97882665.47	15768741.81	0.16109841	COG1965	P	"Protein implicated in iron transport, frataxin homolog"	K06202	METABOLISM
PA5275		conserved hypothetical protein	97882665.47	15768741.81	0.16109841	COG1965	P	"Protein implicated in iron transport, frataxin homolog"	K06202	METABOLISM
PA5275		conserved hypothetical protein	97882665.47	15768741.81	0.16109841	COG1965	P	"Protein implicated in iron transport, frataxin homolog"	K06202	METABOLISM
PA5275		conserved hypothetical protein	97882665.47	15768741.81	0.16109841	COG1965	P	"Protein implicated in iron transport, frataxin homolog"	K06202	METABOLISM
PA5285	sutA	SutA	3167755544	4462582142	1.408752058					
PA5288	glnK	nitrogen regulatory protein P-II 2	3778376939	3823340174	1.011900145	COG0347	E	Nitrogen regulatory protein PII	K04751	METABOLISM
PA5288	glnK	nitrogen regulatory protein P-II 2	3778376939	3823340174	1.011900145	COG0347	E	Nitrogen regulatory protein PII	K04752	METABOLISM
PA5289		hypothetical protein	0	2517709771	1000	COG2960	S	Uncharacterized protein conserved in bacteria	K09806	POORLY CHARACTERIZED
PA5300	cycB	cytochrome c5	14965093408	10327342550	0.690095429					
PA5303		conserved hypothetical protein	0	75953115.71	1000	COG0251	J	"Putative translation initiation inhibitor, yjgF family"	K07567	INFORMATION STORAGE AND PROCESSING
PA5305		conserved hypothetical protein	135368789.9	1122788348	8.294292574	COG3784	S	Uncharacterized protein conserved in bacteria	K09978	POORLY CHARACTERIZED
PA5312	pauC	Aldehyde dehydrogenase	0	11469523.28	1000	COG1012	C	NAD-dependent aldehyde dehydrogenases	K00140	METABOLISM
PA5312	pauC	Aldehyde dehydrogenase	0	11469523.28	1000	COG1012	C	NAD-dependent aldehyde dehydrogenases	K00128	METABOLISM
PA5312	pauC	Aldehyde dehydrogenase	0	11469523.28	1000	COG1012	C	NAD-dependent aldehyde dehydrogenases	K00130	METABOLISM
PA5312	pauC	Aldehyde dehydrogenase	0	11469523.28	1000	COG1012	C	NAD-dependent aldehyde dehydrogenases	K00135	METABOLISM
PA5315	rpmG	50S ribosomal protein L33	10980648239	11707032155	1.066151278	COG0267	J	Ribosomal protein L33	K02913	INFORMATION STORAGE AND PROCESSING
PA5323	argB	acetylglutamate kinase	531319725.1	341931308	0.643550939	COG0548	E	Acetylglutamate kinase	K00930	METABOLISM

PA5323	argB	acetylglutamate kinase	531319725.1	341931308	0.643550939	COG0548	E	Acetylglutamate kinase	K00619	METABOLISM
PA5325	sphA	SphA	30621452.77	0	0					
PA5330		hypothetical protein	31687434.82	28903447.29	0.912142225					
PA5335		conserved hypothetical protein	41358668.76	74018754.24	1.789679321					
PA5337	rpoZ	RNA polymerase omega subunit	4582450507	5651858249	1.233370276	COG1758	K	"DNA-directed RNA polymerase, subunit K/omega"	K03014	INFORMATION STORAGE AND PROCESSING
PA5337	rpoZ	RNA polymerase omega subunit	4582450507	5651858249	1.233370276	COG1758	K	"DNA-directed RNA polymerase, subunit K/omega"	K03055	INFORMATION STORAGE AND PROCESSING
PA5337	rpoZ	RNA polymerase omega subunit	4582450507	5651858249	1.233370276	COG1758	K	"DNA-directed RNA polymerase, subunit K/omega"	K03060	INFORMATION STORAGE AND PROCESSING
PA5339		conserved hypothetical protein	2502326244	2893611217	1.156368489	COG0251	J	"Putative translation initiation inhibitor, yjgF family"	K07567	INFORMATION STORAGE AND PROCESSING
PA5340		hypothetical protein	304502842.7	53595553.75	0.176010028					
PA5348		probable DNA-binding protein	425038565.9	1089569809	2.563461052	COG0776	L	Bacterial nucleoid DNA-binding protein	K04764	INFORMATION STORAGE AND PROCESSING
PA5348		probable DNA-binding protein	425038565.9	1089569809	2.563461052	COG0776	L	Bacterial nucleoid DNA-binding protein	K05787	INFORMATION STORAGE AND PROCESSING
PA5348		probable DNA-binding protein	425038565.9	1089569809	2.563461052	COG0776	L	Bacterial nucleoid DNA-binding protein	K03530	INFORMATION STORAGE AND PROCESSING
PA5348		probable DNA-binding protein	425038565.9	1089569809	2.563461052	COG0776	L	Bacterial nucleoid DNA-binding protein	K05788	INFORMATION STORAGE AND PROCESSING
PA5355	glcD	glycolate oxidase subunit GlcD	0	137079664.9	1000	COG0277	C	FAD/FMN-containing dehydrogenases	K11472	METABOLISM
PA5355	glcD	glycolate oxidase subunit GlcD	0	137079664.9	1000	COG0277	C	FAD/FMN-containing dehydrogenases	K06911	METABOLISM
PA5355	glcD	glycolate oxidase subunit GlcD	0	137079664.9	1000	COG0277	C	FAD/FMN-containing dehydrogenases	K00104	METABOLISM
PA5355	glcD	glycolate oxidase subunit GlcD	0	137079664.9	1000	COG0277	C	FAD/FMN-containing dehydrogenases	K00102	METABOLISM
PA5359		hypothetical protein	23037303.06	0	0					
PA5373	betB	betaine aldehyde dehydrogenase	0	25943335.88	1000	COG1012	C	NAD-dependent aldehyde dehydrogenases	K00135	METABOLISM
PA5373	betB	betaine aldehyde dehydrogenase	0	25943335.88	1000	COG1012	C	NAD-dependent aldehyde dehydrogenases	K00140	METABOLISM
PA5373	betB	betaine aldehyde dehydrogenase	0	25943335.88	1000	COG1012	C	NAD-dependent aldehyde dehydrogenases	K00128	METABOLISM
PA5373	betB	betaine aldehyde dehydrogenase	0	25943335.88	1000	COG1012	C	NAD-dependent aldehyde dehydrogenases	K00130	METABOLISM
PA5381		hypothetical protein	0	23994152.82	1000					
PA5406		hypothetical protein	115291030	110741787.7	0.960541229					
PA5407		hypothetical protein	25698074.16	0	0	COG3027	S	Uncharacterized protein conserved in bacteria	K09888	POORLY CHARACTERIZED
PA5414		hypothetical protein	147881953.8	83353681.1	0.563650121					
PA5427	adhA	alcohol dehydrogenase	181968360.5	0	0	COG1064	R	Zn-dependent alcohol dehydrogenases	K13979	POORLY CHARACTERIZED
PA5427	adhA	alcohol dehydrogenase	181968360.5	0	0	COG1064	R	Zn-dependent alcohol dehydrogenases	K13953	POORLY CHARACTERIZED
PA5427	adhA	alcohol dehydrogenase	181968360.5	0	0	COG1064	R	Zn-dependent alcohol dehydrogenases	K00001	POORLY CHARACTERIZED
PA5435		probable transcarboxylase subunit	0	10761146.7	1000	COG0511	I	Biotin carboxyl carrier protein	K01571	METABOLISM
PA5435		probable transcarboxylase subunit	0	10761146.7	1000	COG5016	C	Pyruvate/oxaloacetate carboxyltransferase	K01571	METABOLISM
PA5435		probable transcarboxylase subunit	0	10761146.7	1000	COG0511	I	Biotin carboxyl carrier protein	K02160	METABOLISM
PA5435		probable transcarboxylase subunit	0	10761146.7	1000	COG5016	C	Pyruvate/oxaloacetate carboxyltransferase	K01960	METABOLISM
PA5435		probable transcarboxylase subunit	0	10761146.7	1000	COG0511	I	Biotin carboxyl carrier protein	K01960	METABOLISM
PA5441		hypothetical protein	14656399.22	0	0					
PA5472		hypothetical protein	293785043.9	82929974.75	0.282281132	COG0834	T	"ABC-type amino acid transport/signal transduction systems, periplasmic compon"	K02030	CELLULAR PROCESSES AND SIGNALING
PA5472		hypothetical protein	293785043.9	82929974.75	0.282281132	COG0834	E	"ABC-type amino acid transport/signal transduction systems, periplasmic compon"	K02030	METABOLISM
PA5481		hypothetical protein	93942015.73	0	0					
PA5487		hypothetical protein	13745139.98	97861726.8	7.119733009	COG3706	T	Response regulator containing a CheY-like receiver domain and a GGDEF domain	K02488	CELLULAR PROCESSES AND SIGNALING
PA5489	dsbA	thiol:disulfide interchange protein DsbA	41898371.27	19502922.93	0.465481648	COG1651	O	Protein-disulfide isomerase	K03805	CELLULAR PROCESSES AND SIGNALING
PA5489	dsbA	thiol:disulfide interchange protein DsbA	41898371.27	19502922.93	0.465481648	COG1651	O	Protein-disulfide isomerase	K03981	CELLULAR PROCESSES AND SIGNALING
PA5490	cc4	cytochrome c4 precursor	1498475823	2531687969	1.689508719					
PA5494		hypothetical protein	315548189.3	95249014.43	0.301852515					
PA5496	nrdJb	"class II (cobalamin-dependent) ribonucleotide-diphosphate reductase subunit	235186173.4	521118356.8	2.215769529					
PA5502		hypothetical protein	0	129359048.7	1000					
PA5505		probable TonB-dependent receptor	2422369052	59199072.57	2.44E-02	COG1464	P	"ABC-type metal ion transport system, periplasmic component/surface antigen"	K02073	METABOLISM
PA5528		hypothetical protein	620756591.9	197783463.9	0.318616776					
PA5553	atpC	ATP synthase epsilon chain	99153236.82	536871894.6	5.414567509	COG0355	C	"F0F1-type ATP synthase, epsilon subunit (mitochondrial delta subunit)"	K02114	METABOLISM
PA5554	atpD	ATP synthase beta chain	119162088.7	206556795.8	1.733410333	COG0055	C	"F0F1-type ATP synthase, beta subunit"	K02112	METABOLISM
PA5554	atpD	ATP synthase beta chain	119162088.7	206556795.8	1.733410333	COG0055	C	"F0F1-type ATP synthase, beta subunit"	K02133	METABOLISM
PA5555	atpG	ATP synthase gamma chain	0	29571566.5	1000	COG0224	C	"F0F1-type ATP synthase, gamma subunit"	K02115	METABOLISM
PA5556	atpA	ATP synthase alpha chain	673309745.7	1743107444	2.588864122	COG0056	C	"F0F1-type ATP synthase, alpha subunit"	K02111	METABOLISM
PA5557	atpH	ATP synthase delta chain	5496053645	5101779167	0.928262258	COG0712	C	"F0F1-type ATP synthase, delta subunit (mitochondrial oligomycin sensitivity p"	K02113	METABOLISM
PA5558	atpF	ATP synthase B chain	13625972678	19048425913	1.39794981	COG0711	C	"F0F1-type ATP synthase, subunit b"	K02109	METABOLISM
PA5562	spoOJ	chromosome partitioning protein Spo0J	77251797.23	0	0	COG1475	K	Predicted transcriptional regulators	K03497	INFORMATION STORAGE AND PROCESSING
PA5568		conserved hypothetical protein	0	93119964.15	1000	COG0706	U	Preprotein translocase subunit YidC	K03217	CELLULAR PROCESSES AND SIGNALING
PA5570	rpmH	50S ribosomal protein L34	7186532610	1740227273	0.242151169	COG0230	J	Ribosomal protein L34	K02914	INFORMATION STORAGE AND PROCESSING

Table. S4. KEGG pathways of up-regulated proteins from the planktonic cells grown in control and antibiotic-impregnated catheters

General description	Functional category	KEGG pathway	Count	Count (%)
Metabolism	Amino acid metabolism	pae00280 Valine, leucine and isoleucine degradation	31	2.35
		pae00310 Lysine degradation	19	1.44
		pae00380 Tryptophan metabolism	18	1.36
		pae00220 Arginine biosynthesis	16	1.21
		pae00260 Glycine, serine and threonine metabolism	15	1.14
		pae00250 Alanine, aspartate and glutamate metabolism	14	1.06
		pae00270 Cysteine and methionine metabolism	12	0.91
		pae00360 Phenylalanine metabolism	10	0.76
		pae00350 Tyrosine metabolism	6	0.45
		pae00400 Phenylalanine, tyrosine and tryptophan biosynthesis	5	0.38
		pae00330 Arginine and proline metabolism	5	0.38
		pae00300 Lysine biosynthesis	5	0.38
		pae00340 Histidine metabolism	3	0.23
		pae00290 Valine, leucine and isoleucine biosynthesis	3	0.23
	Biosynthesis of other secondary metabolites	pae00405 Phenazine biosynthesis	2	0.15
		pae00261 Monobactam biosynthesis	2	0.15
		pae00401 Novobiocin biosynthesis	2	0.15
		pae00332 Carbapenem biosynthesis	1	0.08
		pae00521 Streptomycin biosynthesis	1	0.08
		pae00525 Acarbose and validamycin biosynthesis	1	0.08
	Carbohydrate metabolism	pae00620 Pyruvate metabolism	34	2.58
		pae00640 Propanoate metabolism	31	2.35
		pae00630 Glyoxylate and dicarboxylate metabolism	30	2.27
		pae00020 Citrate cycle (TCA cycle)	21	1.59
		pae00650 Butanoate metabolism	19	1.44
		pae00010 Glycolysis/Gluconeogenesis	17	1.29

	pae00030 Pentose phosphate pathway	6	0.45
	pae00051 Fructose and mannose metabolism	3	0.23
	pae00660 C5-Branched dibasic acid metabolism	3	0.23
	pae00562 Inositol phosphate metabolism	3	0.23
	pae00053 Ascorbate and aldarate metabolism	2	0.15
	pae00520 Amino sugar and nucleotide sugar metabolism	2	0.15
	pae00040 Pentose and glucuronate interconversions	1	0.08
Energy metabolism	pae00910 Nitrogen metabolism	16	1.21
	pae00680 Methane metabolism	11	0.83
	pae00190 Oxidative phosphorylation	10	0.76
Global and overview map	pae01100 Metabolic pathways	205	15.53
	pae01110 Biosynthesis of secondary metabolites	117	8.86
	pae01120 Microbial metabolism in diverse environments	91	6.89
	pae01200 Carbon metabolism	65	4.92
	pae01230 Biosynthesis of amino acids	48	3.64
	pae01212 Fatty acid metabolism	40	3.03
	pae01220 Degradation of aromatic compounds	2	0.15
	pae01210 2-Oxocarboxylic acid metabolism	8	0.61
Glycan biosynthesis and metabolism	pae00540 Lipopolysaccharide biosynthesis	2	0.15
	pae00550 Peptidoglycan biosynthesis	1	0.08
Lipid metabolism	pae00071 Fatty acid degradation	24	1.82
	pae00061 Fatty acid biosynthesis	19	1.44
	pae00072 Synthesis and degradation of ketone bodies	6	0.45
	pae00592 alpha-Linolenic acid metabolism	3	0.23
	pae00564 Glycerophospholipid metabolism	2	0.15
	pae00561 Glycerolipid metabolism	2	0.15
	pae01040 Biosynthesis of unsaturated fatty acids	1	0.08
Metabolism of cofactors and vitamins	pae00780 Biotin metabolism	13	0.98
	pae00750 Vitamin B6 metabolism	4	0.30
	pae00760 Nicotinate and nicotinamide metabolism	3	0.23

		pae00670 One carbon pool by folate	3	0.23
		pae00770 Pantothenate and CoA biosynthesis	3	0.23
		pae00790 Folate biosynthesis	2	0.15
		pae00130 Ubiquinone and other terpenoid-quinone biosynthesis	2	0.15
		pae00730 Thiamine metabolism	2	0.15
		pae00740 Riboflavin metabolism	1	0.08
	Metabolism of other amino acids	pae00410 beta-Alanine metabolism	11	0.83
		pae00480 Glutathione metabolism	7	0.53
		pae00460 Cyanoamino acid metabolism	7	0.53
		pae00430 Taurine and hypotaurine metabolism	3	0.23
		pae00450 Selenocompound metabolism	2	0.15
		pae00440 Phosphonate and phosphinate metabolism	1	0.08
	Metabolism of terpenoids and polyketides	pae00281 Geraniol degradation	9	0.68
		pae00903 Limonene and pinene degradation	8	0.61
		pae00900 Terpenoid backbone biosynthesis	7	0.53
		pae00523 Polyketide sugar unit biosynthesis	1	0.08
		pae01053 Biosynthesis of siderophore group nonribosomal peptides	1	0.08
	Nucleotide metabolism	pae00230 Purine metabolism	12	0.91
		pae00240 Pyrimidine metabolism	4	0.30
	Xenobiotics biodegradation and metabolism	pae00362 Benzoate degradation	18	1.36
		pae00930 Caprolactam degradation	6	0.45
		pae00627 Aminobenzoate degradation	4	0.30
		pae00625 Chloroalkane and chloroalkene degradation	3	0.23
		pae00626 Naphthalene degradation	1	0.08
Cellular Processes	Cell motility	pae02030 Bacterial chemotaxis	23	1.74
		pae02040 Flagellar assembly	4	0.30
	Cellular community - prokaryotes	pae02024 Quorum sensing	8	0.61
	Signal transduction	pae02020 Two-component system	43	3.26
	Membrane transport	pae03070 Bacterial secretion system	15	1.14

Environmental Information Processing		pae02010 ABC transporters	2	0.15
		pae02060 Phosphotransferase system (PTS)	1	0.08
Genetic Information Processing	Translation	pae03010 Ribosome	24	1.82
		pae00970 Aminoacyl-tRNA biosynthesis	11	0.83
	Folding, sorting and degradation	pae03018 RNA degradation	6	0.45
		pae03060 Protein export	5	0.38
		pae00920 Sulfur metabolism	4	0.30
		pae04122 Sulfur relay system	4	0.30
	Transcription	pae03020 RNA polymerase	3	0.23
	Replication and repair	pae03430 Mismatch repair	1	0.08
		pae03030 DNA replication	1	0.08
		pae03440 Homologous recombination	1	0.08
Human Diseases	Drug resistance: antimicrobial	pae01503 Cationic antimicrobial peptide (CAMP) resistance	6	0.45
		pae01501 beta-Lactam resistance	4	0.30

Table. S5. KEGG pathways of down-regulated proteins from the planktonic cells grown in control and antibiotic-impregnated catheters

General description	Functional category	KEGG pathway	Count	Count (%)
Metabolism	Amino acid metabolism	pae00380 Tryptophan metabolism	8	1.99
		pae00360 Phenylalanine metabolism	7	1.74
		pae00330 Arginine and proline metabolism	6	1.49
		pae00400 Phenylalanine, tyrosine and tryptophan biosynthesis	5	1.24
		pae00260 Glycine, serine and threonine metabolism	4	0.99
		pae00250 Alanine, aspartate and glutamate metabolism	3	0.74
		pae00270 Cysteine and methionine metabolism	3	0.74
		pae00220 Arginine biosynthesis	2	0.50
		pae00290 Valine, leucine and isoleucine biosynthesis	1	0.25
		pae00280 Valine, leucine and isoleucine degradation	1	0.25
		pae00300 Lysine biosynthesis	1	0.25
	Biosynthesis of other secondary metabolites	pae00261 Monobactam biosynthesis	1	0.25
	Carbohydrate metabolism	pae00630 Glyoxylate and dicarboxylate metabolism	9	2.23
		pae00010 Glycolysis / Gluconeogenesis	3	0.74
		pae00020 Citrate cycle (TCA cycle)	2	0.50
		pae00030 Pentose phosphate pathway	1	0.25
		pae00640 Propanoate metabolism	1	0.25
		pae00620 Pyruvate metabolism	1	0.25
		pae00051 Fructose and mannose metabolism	1	0.25
		pae00562 Inositol phosphate metabolism	1	0.25
	Energy metabolism	pae00190 Oxidative phosphorylation	3	0.74
		pae00920 Sulfur metabolism	3	0.74
		pae00680 Methane metabolism	3	0.74
	Global and overview map	pae01100 Metabolic pathways	64	15.88
		pae01110 Biosynthesis of secondary metabolites	35	8.68

		pae01120 Microbial metabolism in diverse environments	19	4.71
		pae01230 Biosynthesis of amino acids	14	3.47
		pae01200 Carbon metabolism	12	2.98
		pae01212 Fatty acid metabolism	10	2.48
		pae01220 Degradation of aromatic compounds	3	0.74
		pae01210 2-Oxocarboxylic acid metabolism	4	0.99
	Lipid metabolism	pae00061 Fatty acid biosynthesis	10	2.48
		pae00071 Fatty acid degradation	2	0.50
	Metabolism of cofactors and vitamins	pae00780 Biotin metabolism	8	1.99
		pae00860 Porphyrin and chlorophyll metabolism	3	0.74
		pae00770 Pantothenate and CoA biosynthesis	2	0.50
		pae00760 Nicotinate and nicotinamide metabolism	2	0.50
		pae00730 Thiamine metabolism	2	0.50
	Metabolism of other amino acids	pae00480 Glutathione metabolism	1	0.25
		pae00410 beta-Alanine metabolism	1	0.25
	Metabolism of terpenoids and polyketides	pae01053 Biosynthesis of siderophore group nonribosomal peptides	1	0.25
	Nucleotide metabolism	pae00230 Purine metabolism	3	0.74
	Xenobiotics biodegradation and metabolism	pae00627 Aminobenzoate degradation	5	1.24
		pae00643 Styrene degradation	5	1.24
		pae00362 Benzoate degradation	3	0.74
		pae00623 Toluene degradation	1	0.25
		pae00364 Fluorobenzoate degradation	1	0.25
		pae00361 Chlorocyclohexane and chlorobenzene degradation	1	0.25
Cellular Processes	Cell motility	pae02030 Bacterial chemotaxis	20	4.96
		pae02040 Flagellar assembly	9	2.23
	Cellular community - prokaryotes	pae02024 Quorum sensing	14	3.47
		pae02025 Biofilm formation	4	0.99
	Signal transduction	pae02020 Two-component system	28	6.95
	Membrane transport	pae02010 ABC transporters	16	3.97

Environmental Information Processing		pae03070 Bacterial secretion system	15	3.72
		pae02060 Phosphotransferase system (PTS)	1	0.25
Genetic Information Processing	Translation	pae03010 Ribosome	3	0.74
		pae00970 Aminoacyl-tRNA biosynthesis	2	0.50
	Folding, sorting and degradation	pae03060 Protein export	2	0.50
		pae03018 RNA degradation	1	0.25
	Replication and repair	pae03030 DNA replication	1	0.25
		pae03440 Homologous recombination	1	0.25
Human Diseases	Drug resistance: antimicrobial	pae01501 beta-Lactam resistance	2	0.50
		pae01503 Cationic antimicrobial peptide (CAMP) resistance	2	0.50

Table. S6. KEGG pathways of up-regulated proteins from the biofilm cells grown in control and antibiotic-impregnated catheters

General description	Functional category	KEGG pathway	Count	Count (%)
Metabolism	Amino acid metabolism	pae00270 Cysteine and methionine metabolism	5	1.42
		pae00280 Valine, leucine and isoleucine degradation	5	1.42
		pae00310 Lysine degradation	3	0.85
		pae00260 Glycine, serine and threonine metabolism	3	0.85
		pae00380 Tryptophan metabolism	3	0.85
		pae00340 Histidine metabolism	2	0.57
		pae00330 Arginine and proline metabolism	2	0.57
		pae00350 Tyrosine metabolism	1	0.28
		pae00250 Alanine, aspartate and glutamate metabolism	1	0.28
		pae00220 Arginine biosynthesis	1	0.28
	Carbohydrate metabolism	pae00620 Pyruvate metabolism	11	3.13
		pae00020 Citrate cycle (TCA cycle)	7	1.99
		pae00640 Propanoate metabolism	7	1.99
		pae00010 Glycolysis / Gluconeogenesis	4	1.14
		pae00630 Glyoxylate and dicarboxylate metabolism	3	0.85
		pae00562 Inositol phosphate metabolism	3	0.85
		pae00053 Ascorbate and aldarate metabolism	2	0.57
		pae00030 Pentose phosphate pathway	2	0.57
		pae00660 C5-Branched dibasic acid metabolism	1	0.28
		pae00650 Butanoate metabolism	1	0.28
		pae00040 Pentose and glucuronate interconversions	1	0.28
		pae00051 Fructose and mannose metabolism	1	0.28
	Energy metabolism	pae00190 Oxidative phosphorylation	9	2.56
		pae00680 Methane metabolism	1	0.28
		pae00910 Nitrogen metabolism	1	0.28
	Global and overview map	pae01100 Metabolic pathways	52	14.77

		pae01120 Microbial metabolism in diverse environments	28	7.95
		pae01110 Biosynthesis of secondary metabolites	23	6.53
		pae01200 Carbon metabolism	21	5.97
		pae01230 Biosynthesis of amino acids	9	2.56
		pae01212 Fatty acid metabolism	3	0.85
		pae01210 2-Oxocarboxylic acid metabolism	2	0.57
		pae01220 Degradation of aromatic compounds	3	0.85
	Glycan biosynthesis and metabolism	pae00540 Lipopolysaccharide biosynthesis	1	0.28
	Lipid metabolism	pae00061 Fatty acid biosynthesis	3	0.85
		pae00564 Glycerophospholipid metabolism	2	0.57
		pae00071 Fatty acid degradation	2	0.57
		pae00561 Glycerolipid metabolism	2	0.57
	Metabolism of cofactors and vitamins	pae00790 Folate biosynthesis	4	1.14
		pae00670 One carbon pool by folate	1	0.28
		pae00750 Vitamin B6 metabolism	1	0.28
		pae00760 Nicotinate and nicotinamide metabolism	1	0.28
	Metabolism of other amino acids	pae00410 beta-Alanine metabolism	5	1.42
		pae00480 Glutathione metabolism	2	0.57
	Metabolism of terpenoids and polyketides	pae00903 Limonene and pinene degradation	2	0.57
	Nucleotide metabolism	pae00230 Purine metabolism	4	1.14
		pae00240 Pyrimidine metabolism	1	0.28
	Xenobiotics biodegradation and metabolism	pae00362 Benzoate degradation	3	0.85
		pae00625 Chloroalkane and chloroalkene degradation	2	0.57
		pae00633 Nitrotoluene degradation	2	0.57
		pae00623 Toluene degradation	1	0.28
		pae00361 Chlorocyclohexane and chlorobenzene degradation	1	0.28
		pae00364 Fluorobenzoate degradation	1	0.28
Cellular Processes	Cell motility	pae02030 Bacterial chemotaxis	20	5.68
		pae02040 Flagellar assembly	1	0.28

	Cellular community - prokaryotes	pae02024 Quorum sensing	4	1.14
Environmental Information Processing	Signal transduction	pae02020 Two-component system	29	8.24
	Membrane transport	pae03070 Bacterial secretion system	6	1.70
Genetic Information Processing	Translation	pae03010 Ribosome	12	3.41
		pae00970 Aminoacyl-tRNA biosynthesis	3	0.85
	Folding, sorting and degradation	pae00920 Sulfur metabolism	3	0.85
		pae03060 Protein export	3	0.85
		pae04122 Sulfur relay system	2	0.57
		pae03018 RNA degradation	1	0.28
	Transcription	pae03020 RNA polymerase	1	0.28
	Replication and repair	pae03430 Mismatch repair	1	0.28
Human Diseases	Drug resistance: antimicrobial	pae01501 beta-Lactam resistance	2	0.57
		pae01503 Cationic antimicrobial peptide (CAMP) resistance	2	0.57

Table. S7. KEGG pathways of down-regulated proteins from the biofilm cells grown in control and antibiotic-impregnated catheters

General description	Functional category	KEGG pathway	Count	Count (%)
Metabolism	Amino acid metabolism	pae00260 Glycine, serine and threonine metabolism	5	1.24
		pae00400 Phenylalanine, tyrosine and tryptophan biosynthesis	5	1.24
		pae00350 Tyrosine metabolism	4	0.99
		pae00380 Tryptophan metabolism	3	0.74
		pae00270 Cysteine and methionine metabolism	3	0.74
		pae00220 Arginine biosynthesis	3	0.74
		pae00360 Phenylalanine metabolism	2	0.50
		pae00280 Valine, leucine and isoleucine degradation	1	0.25
		pae00250 Alanine, aspartate and glutamate metabolism	1	0.25
		pae00340 Histidine metabolism	1	0.25
	Carbohydrate metabolism	pae00630 Glyoxylate and dicarboxylate metabolism	10	2.48
		pae00620 Pyruvate metabolism	5	1.24
		pae00010 Glycolysis/Gluconeogenesis	5	1.24
		pae00030 Pentose phosphate pathway	4	0.99
		pae00051 Fructose and mannose metabolism	3	0.74
		pae00640 Propanoate metabolism	3	0.74
		pae00020 Citrate cycle (TCA cycle)	1	0.25
		pae00562 Inositol phosphate metabolism	1	0.25
	Energy metabolism	pae00680 Methane metabolism	2	0.50
		pae00920 Sulfur metabolism	2	0.50
		pae00190 Oxidative phosphorylation	1	0.25
	Global and overview map	pae01100 Metabolic pathways	65	16.09
		pae01110 Biosynthesis of secondary metabolites	37	9.16
		pae01120 Microbial metabolism in diverse environments	19	4.70
		pae01200 Carbon metabolism	14	3.47
		pae01230 Biosynthesis of amino acids	13	3.22
		pae01220 Degradation of aromatic compounds	4	0.99

		pae01212 Fatty acid metabolism	2	0.50
	Glycan biosynthesis and metabolism	pae00540 Lipopolysaccharide biosynthesis	2	0.50
		pae00550 Peptidoglycan biosynthesis	1	0.25
	Lipid metabolism	pae00061 Fatty acid biosynthesis	2	0.50
		pae00071 Fatty acid degradation	1	0.25
		pae01040 Biosynthesis of unsaturated fatty acids	1	0.25
	Metabolism of cofactors and vitamins	pae00730 Thiamine metabolism	3	0.74
		pae00790 Folate biosynthesis	1	0.25
		pae00760 Nicotinate and nicotinamide metabolism	1	0.25
		pae00770 Pantothenate and CoA biosynthesis	1	0.25
	Metabolism of other amino acids	pae00460 Cyanoamino acid metabolism	3	0.74
		pae00480 Glutathione metabolism	3	0.74
		pae00430 Taurine and hypotaurine metabolism	3	0.74
	Metabolism of terpenoids and polyketides	pae01053 Biosynthesis of siderophore group nonribosomal peptides	1	0.25
		pae00900 Terpenoid backbone biosynthesis	1	0.25
	Nucleotide metabolism	pae00230 Purine metabolism	7	1.73
		pae00240 Pyrimidine metabolism	4	0.99
	Xenobiotics biodegradation and metabolism	pae00362 Benzoate degradation	1	0.25
		pae00626 Naphthalene degradation	1	0.25
		pae00643 Styrene degradation	1	0.25
		pae00625 Chloroalkane and chloroalkene degradation	1	0.25
Cellular Processes	Cell motility	pae02030 Bacterial chemotaxis	26	6.44
		pae02040 Flagellar assembly	4	0.99
	Cellular community - prokaryotes	pae02024 Quorum sensing	15	3.71
		pae02025 Biofilm formation	8	1.98
Environmental Information Processing	Signal transduction	pae02020 Two-component system	28	6.93
	Membrane transport	pae02010 ABC transporters	22	5.45
		pae03070 Bacterial secretion system	13	3.22
		pae02060 Phosphotransferase system (PTS)	3	0.74
	Translation	pae03010 Ribosome	11	2.72

Genetic Information Processing		pae00970 Aminoacyl-tRNA biosynthesis	1	0.25
	Folding, sorting and degradation	pae04122 Sulfur relay system	4	0.99
		pae03018 RNA degradation	2	0.50
		pae03060 Protein export	1	0.25
	Replication and repair	pae03430 Mismatch repair	2	0.50
		pae03440 Homologous recombination	2	0.50
		pae03030 DNA replication	2	0.50
Human Diseases	Drug resistance: antimicrobial	pae01503 Cationic antimicrobial peptide (CAMP) resistance	2	0.50

Fig. S1. Top 25 KEGG pathways of up-regulated proteins from the planktonic cells grown in control and antibiotic-impregnated catheters

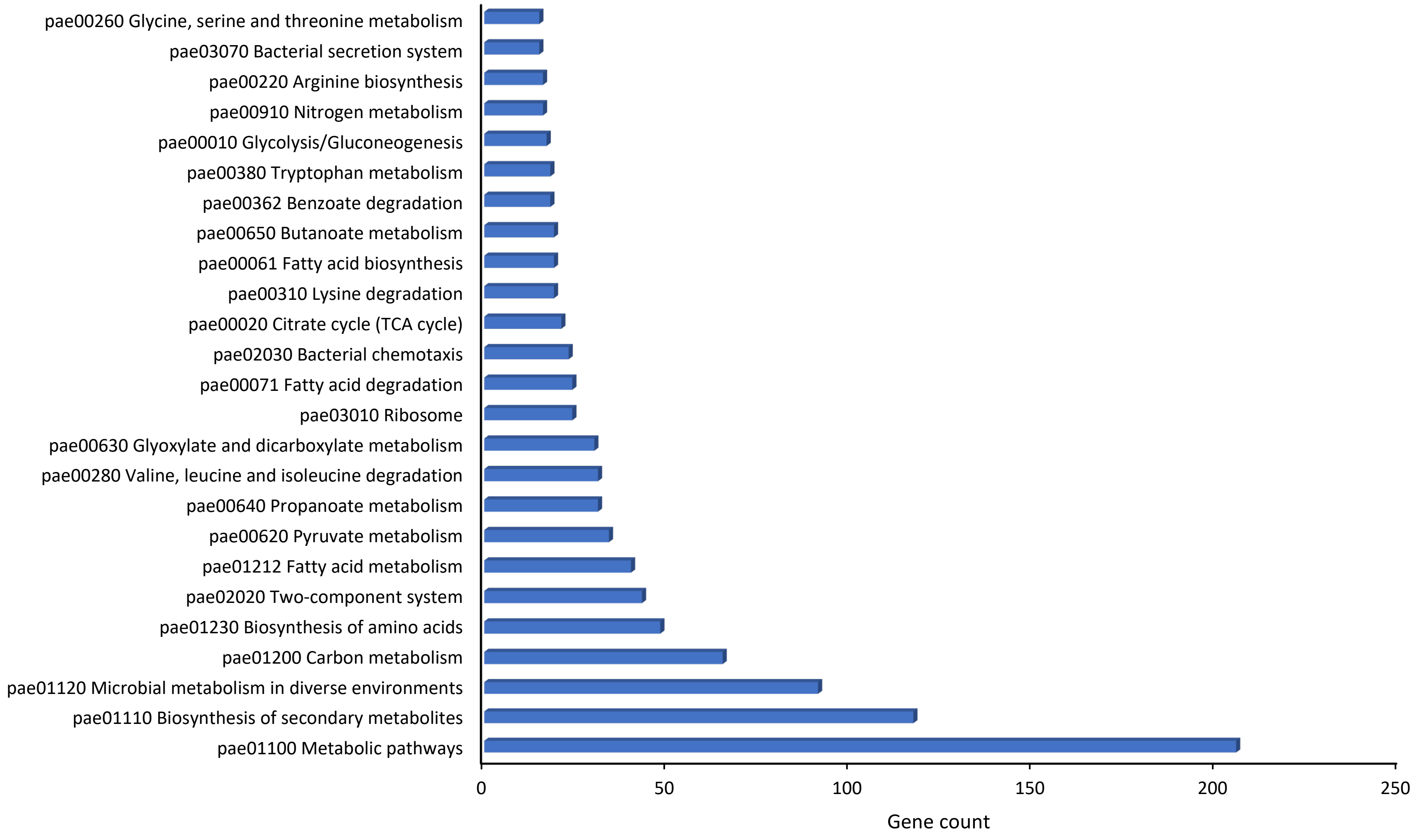

Fig. S2. Top 25 KEGG pathways of down-regulated proteins from the planktonic cells grown in control and antibiotic-impregnated catheter

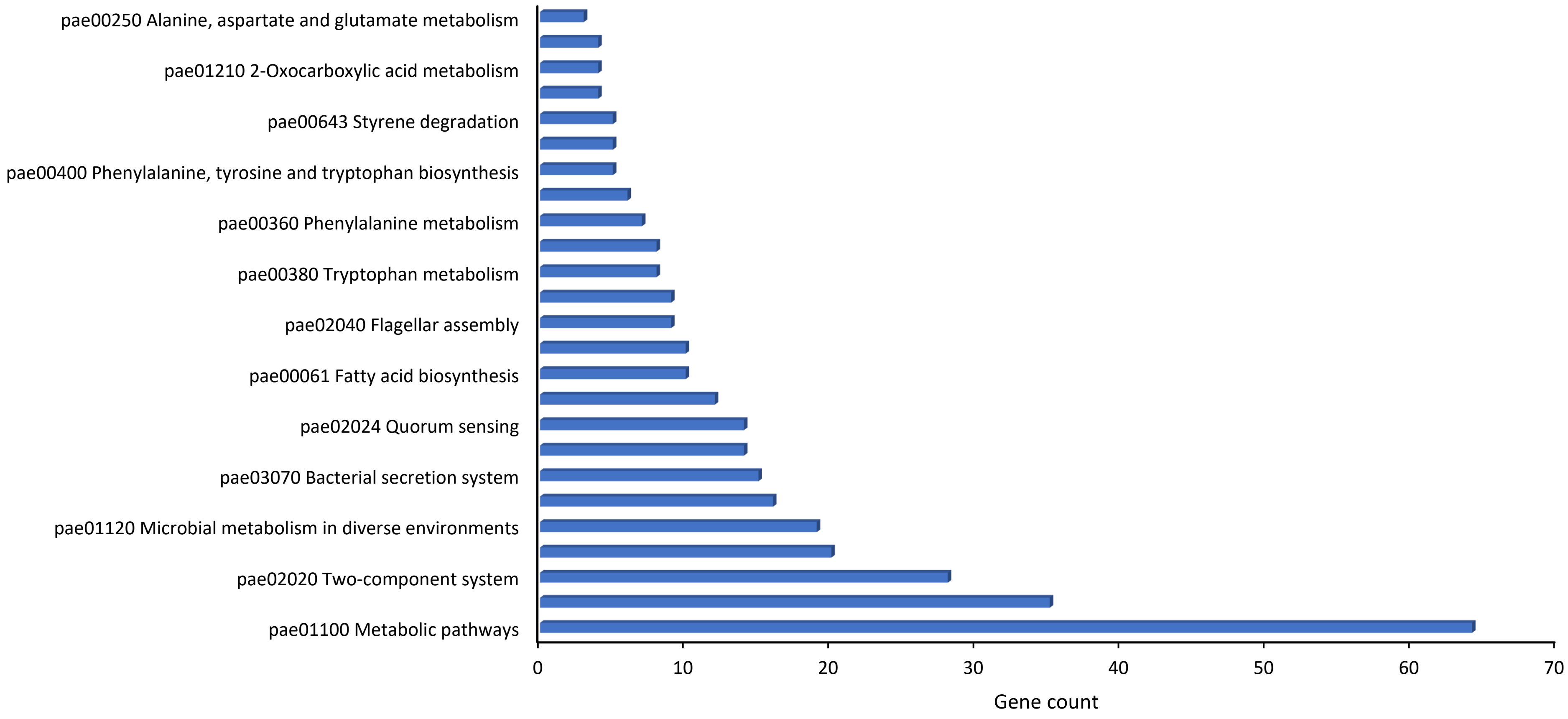

Fig. S3. Top 25 KEGG pathways of up-regulated proteins from the biofilm cells grown in control and antibiotic-impregnated catheters

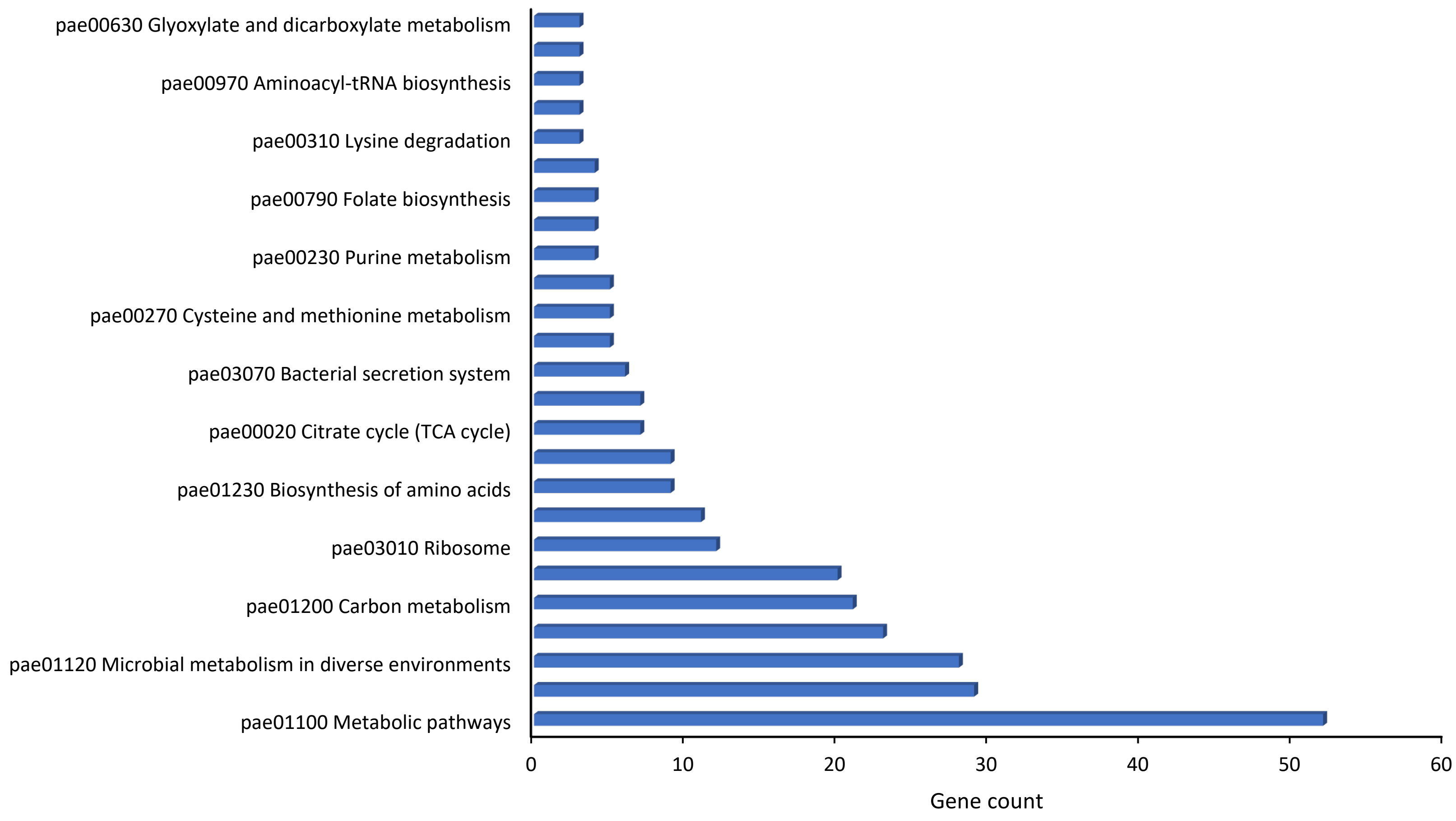

Fig. S4. Top 25 KEGG pathways of down-regulated proteins from the biofilm cells grown in control and antibiotic-impregnated catheters

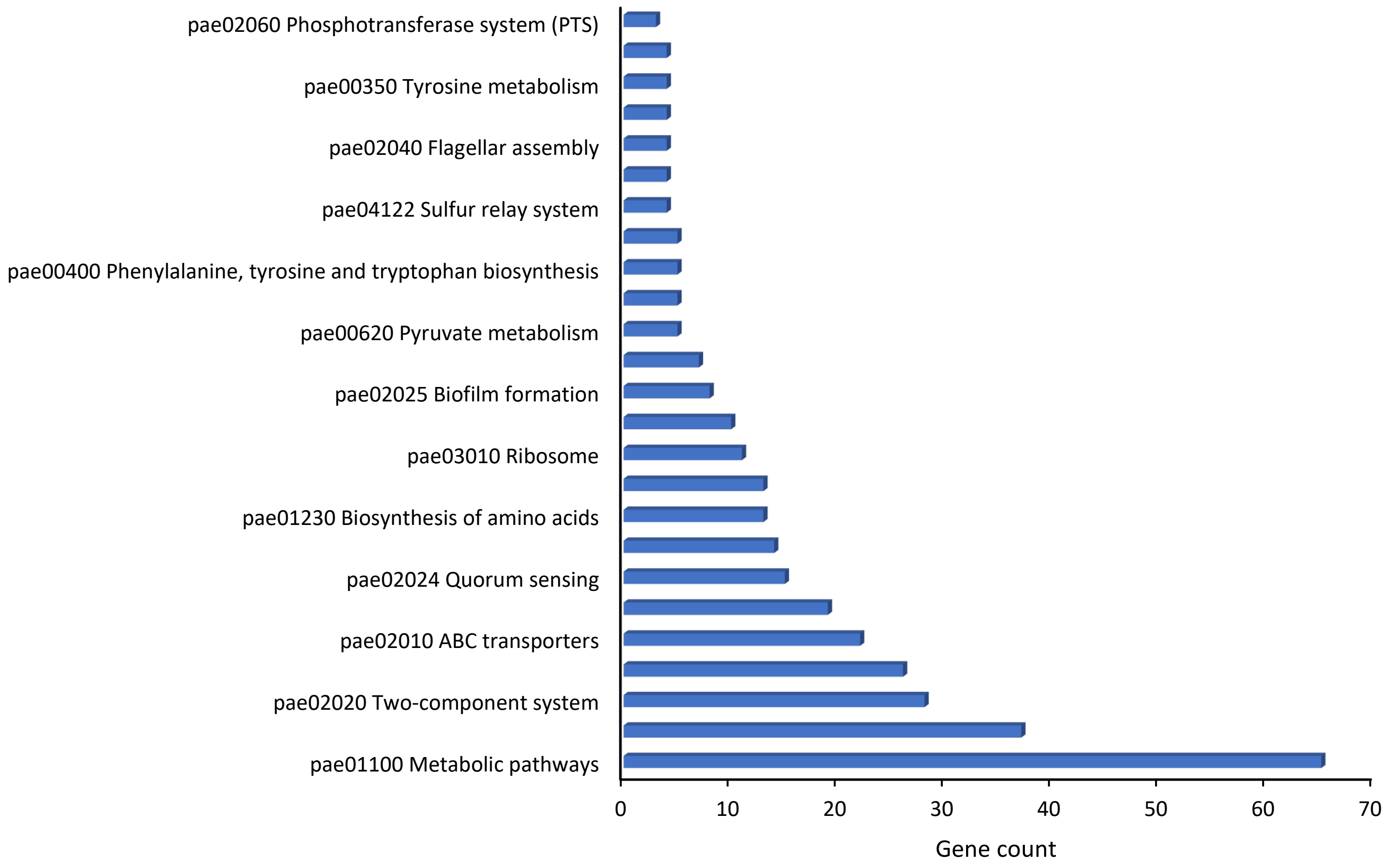

Supplement: Supplementary file 1 [file antibiotics-10-00752-s001.zip › antibiotics-1255778-supplementary.pdf]
